# Substituted Dihydropyridine Synthesis by Dearomatization of Pyridines

**DOI:** 10.1002/anie.202104115

**Published:** 2021-05-10

**Authors:** Arne Heusler, Julian Fliege, Tobias Wagener, Frank Glorius

**Affiliations:** ^1^ Organisch-Chemisches Institut Westfälische Wilhelms-Universität Münster Corrensstrasse 40 48149 Münster Germany

**Keywords:** boranes, chemoselectivity, nitrogen heterocycles, reduction, synthetic methods

## Abstract

Dearomatization is an effective method to transform readily available N‐heterocycles into partially saturated motifs. Manipulation of dihydro‐derivatives holds great potential and provides access to a variety of semi‐saturated N‐heterocyclic building blocks. However, current strategies are limited in scope and the use of sensitive reagents restricts the applicability in synthetic laboratories. Herein, we report the synthesis of a broad variety of N‐substituted 1,4‐ and 1,2‐dihydropyridines by very mild and selective reduction with amine borane for the first time.

## Graphical abstract

Metal‐free dearomatization of N‐heteroarenes is a well‐known and powerful method to yield partially saturated N‐heterocyclic building blocks. Yet, the use of sensitive reagents restricts the applicability in synthetic laboratories. Herein, we present a very mild and selective reduction of N‐heteroarenes by amine borane to synthesize a broad variety of N‐substituted 1,4‐ and 1,2‐dihydropyridines.

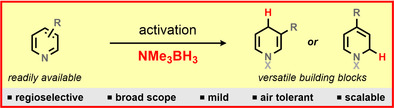

Dihydropyridines (DHPs) are potent motifs in natural product synthesis, and constitute backbones of a variety of active ingredients in the pharmaceutical industry.^[1]^ Especially 1,4‐DHPs offer a wide range of interesting applications in synthetic chemistry, medicinal chemistry, and drug discovery due to their structural similarity to nicotinamide adenine dinucleotide (NADH, Scheme 1 A).^[2]^ The well‐known Hantzsch esters are analogues of NADH modeled on nature and are nowadays widely used as a hydride source in numerous electron transfer reactions.^[3]^ The use of 1,4‐DHPs in medical applications was significantly influenced by the pioneering work of Bossert and Vater in the development of Nifepidine.^[4]^

**Scheme 1:**
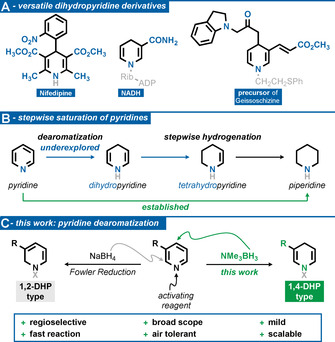
Regioselective synthesis of versatile N‐substituted dihydropyridine‐type motifs by dearomatization of pyridines.

Without doubt, there is high demand for this highly reactive class of substrates.^[5]^ Besides the widespread synthesis approaches by condensation in the Hantzsch ester synthesis, access to DHP motifs with a more versatile substitution pattern is challenging.^[6]^ An alternative synthetic strategy is the direct formation from the unsaturated precursors by dearomatization.^[7]^ The common use and ready accessibility of pyridines makes this method a powerful and flexible tool for accessing DHPs. However, this approach is complicated by the necessity of breaking the aromaticity of the substrates, but at the same time preventing the commonly known full reduction to the piperidine (Scheme 1 B).[^8^, ^9^] In 1972, Fowler described a method in which N‐substituted 1,2‐DHPs were afforded with low selectivity by reduction of activated pyridines with sodium borohydride (Scheme 1 C).^[10]^ Despite the selectivity issue and a limited substrate scope due to the reactive borohydride species, the Fowler Reduction is a widely used method for accessing N‐substituted 1,2‐DHPs in very recent studies that exploited the potential of these interesting motifs.^[11]^

Besides the dearomatization using metal‐containing hydrides, hydroborylation is a common strategy for dearomatization of N‐heteroarenes. Several metal‐catalyzed and metal‐free hydroboration protocols for the dearomatization of N‐heterocycles by established hydridic boron reagents have been reported.^[12]^ Mainly, all these reactions were carried out under strict inert conditions and are limited by their tedious handling. Recently, our group reported a rhodium‐catalyzed transfer hydrogenation of various (hetero‐)arenes utilizing ammonia borane as hydrogen donor resulting in very mild reducing conditions.^[9]^ Moreover, the group of Berke reported a concerted double hydrogen transfer of ammonia borane for the reduction of imines and olefins, demonstrating the catalyst‐free reducing potential of amine borane adducts.^[13]^

We envisioned that amine boranes would be suitable mild reducing agents to give access to DHPs when reacted with pyridines (Scheme 1 C). Since pyridine and amine borane were unreactive, we investigated in situ activated pyridines. Pleasingly, we found that sufficient activation of the model substrate methyl nicotinate for the subsequent reduction step can be achieved by phenyl chloroformate in acetonitrile within 10 minutes (Table S8, entry 9). While reasonable selectivity was achieved for the model substrate, the regioisomeric ratios dropped significantly when the method was expanded to most other substrates. Thus, we investigated the role of the activating reagent further. To our delight, we found that triflic anhydride enables very high regioselectivity for the reduction (Table S8, entry 1). In contrast, other common anhydrides gave only traces or even no conversion to the desired product (Table S8, entries 6–8). Omission of amine borane or triflic anhydride prevented reaction (Table S8, entries 2 and 3). Investigating the influence of the temperature revealed that addition of amine borane to the reaction mixture at −20 °C led to the highest regioselectivity (Table S8, entry 10). With the optimized reaction conditions in hand, we conducted a reaction‐condition‐based sensitivity screen and investigated the scope of the presented method (Table 1, see the Supporting Information for more details).^[14]^ Due to the inherent instability and tendency to decompose under re‐aromatization to the starting material, the isolation of the DHPs is challenging. Therefore, purification was carried out by flash column chromatography over de‐activated neutral alumina (see the Supporting Information for further details). A broad range of 3‐substituted pyridines could be converted to the corresponding 1,4‐DHP‐type products in high yields and very good selectivities (**1**–**14**). The tolerance of carbonyl groups was shown by the synthesis of DHPs **3** and **4**. When scaled up to 20 mmol, DHP **4** was obtained in 87 % isolated yield, proving the notable practicality of this protocol. Fluorine, chlorine, bromine, and iodine substituents were competent in the reaction (**7**–**10**) and the trifluoromethyl group (**5**) could also be tolerated. Sensitive nitrile (**6**) and boronic ester (**11**) groups were compatible and not reduced. When 2‐methylpyridine was used, the product was obtained with very good selectivity but only in low yield (**13**). Other activation methods, including preformed pyridinium species, were unsuccessful. Furthermore, 4‐trifluoromethyl pyridine could be converted to the DHP with excellent selectivity (**14**). Interestingly, it was found that substituents in the 4‐position completely inverted the selectivity to exclusively yield the 1,2‐DHP. Multi‐substituted pyridines could also be converted to DHPs. In the case of 3,5‐disubstitution, 1,4‐DHPs (**15**–**19**) and in the case of 3,4‐disubstitution, the corresponding 1,2‐DHPs (**20**–**23**) were obtained. Owing to the better stability of the higher substituted DHPs, the tolerance of aryl (**18**, **23**), aldehyde (**19**), sulfide (**22**) and silyl (**20**) groups could be demonstrated in great yields and very high selectivities. The reaction could also be applied to other N‐heterocycles, resulting in dihydroquinolines (**24**–**29**) and dihydroisoquinolines (**30**–**34**), among others. The tolerance of functional groups is consistent with those discussed for the pyridine dearomatization. Further, nitrile (**28**, **32**) and nitro groups (**33**) were compatible and not reduced. Unlike for pyridines, variation of the substitution pattern did not result in inverted selectivity (**29**). When quinazoline and pyrimidine were used, the corresponding tetrahydro‐derivatives **35** and **36** were obtained in excellent yields. In contrast, only the dihydro‐derivative **37** was obtained, when the conditions were applied to phthalazine. In almost all cases, the major regioisomer could be separated in the purification process, providing access to the pure major DHP. To explain the observed selectivity, we propose a two‐step mechanism for the reaction of pyridine, triflic anhydride and amine borane: In the first step, triflic anhydride reacts with the pyridine and forms an activated pyridinium species. In the second step, a hydride from the amine borane adds to the most accessible electrophilic carbon, which reduces the pyridine. The attack takes place in the 4‐position but is shifted to the 2‐position, in case this position is blocked by a substituent. The regioselectivity of the dearomatization was confirmed by X‐ray diffraction analysis of product **S1**, when phenyl chloroformate was used as an activating reagent. Furthermore, the regioisomeric outcome of the dearomatization was confirmed by NMR analysis.[^15^, ^16^]

**Table 1:** Substrate scope for the pyridine dearomatization by amine borane and reaction‐condition‐based sensitivity screen.^[a]^

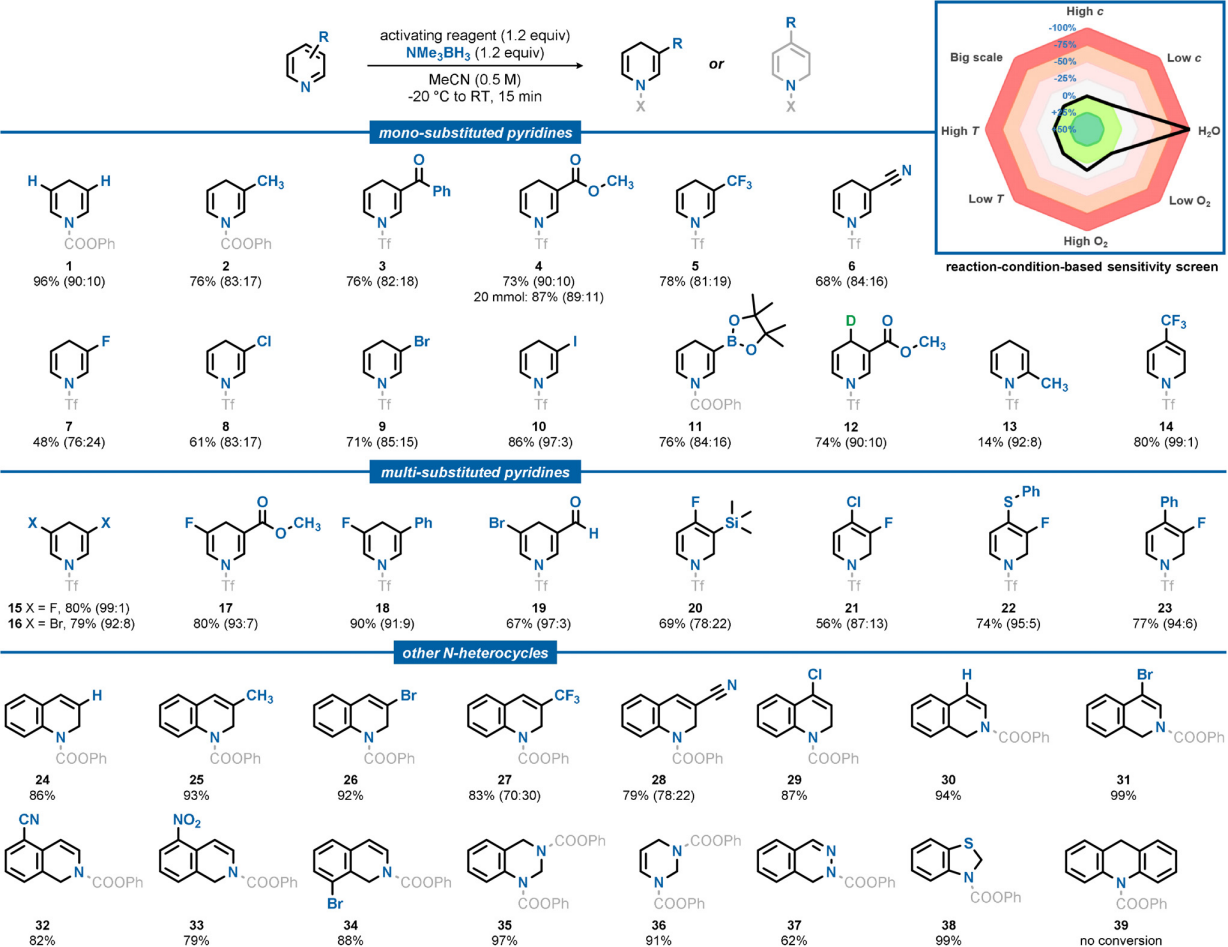

[a] Standard reaction conditions: N‐heterocycle (0.5 mmol), triflic anhydride (1.2 equiv) or phenyl chloroformate (1.2 equiv) and trimethylamine borane (1.2 equiv) in MeCN (0.5 m) at −20 °C warming up to room temperature. Isomeric ratios were determined by GC–FID analysis. Isolated yields reported. See Supporting Information for more details.

To demonstrate the synthetic utility of our method, we sought to showcase exemplary follow‐up functionalization of our in situ obtained DHP‐type products.^[17]^ Subsequent palladium‐catalyzed transfer hydrogenation furnished the corresponding tetrahydropyridines (THP) **40**–**43** selectively in high yields (Scheme 2 A).^[18]^ The position of the remaining C=C‐double bond was confirmed by X‐ray diffraction analysis of product **40** (see Supporting Information for details).[^16^, ^19^] The synthesis of fluorinated piperidine derivatives has been the focus of various studies due to their application as diverse building blocks.^[20]^ Herein, we present a hydrofluorination and difluorination of the synthesized dihydro‐ or tetrahydro‐N‐heterocycles with selectfluor, where the reaction outcome can be controlled by simply varying the equivalents of the reagents (Scheme 2 B,C). The procedures presented provide access to stereoisomeric mixtures of sought‐after fluorinated compounds **44**–**51** and, as it were, illustrate the high relevance of partially saturated N‐heterocycles. To further show the potential of the developed method, a stepwise saturation of pyridine methyl nicotinate was carried out. The last step of each saturation sequence was performed as a deuterium labeling experiment to illustrate the regioselectivity of the respective process (Scheme 2 D). Following the methods presented earlier, the corresponding DHP‐*d* **12** and THP‐*d*_2_ **52** were obtained by applying deuterated amine borane. The remaining THP was successfully deuterated with [Rh(COD)Cl]_2_ in deuterated methanol to give the corresponding piperidine‐*d*_2_ **53**.

**Scheme 2:**
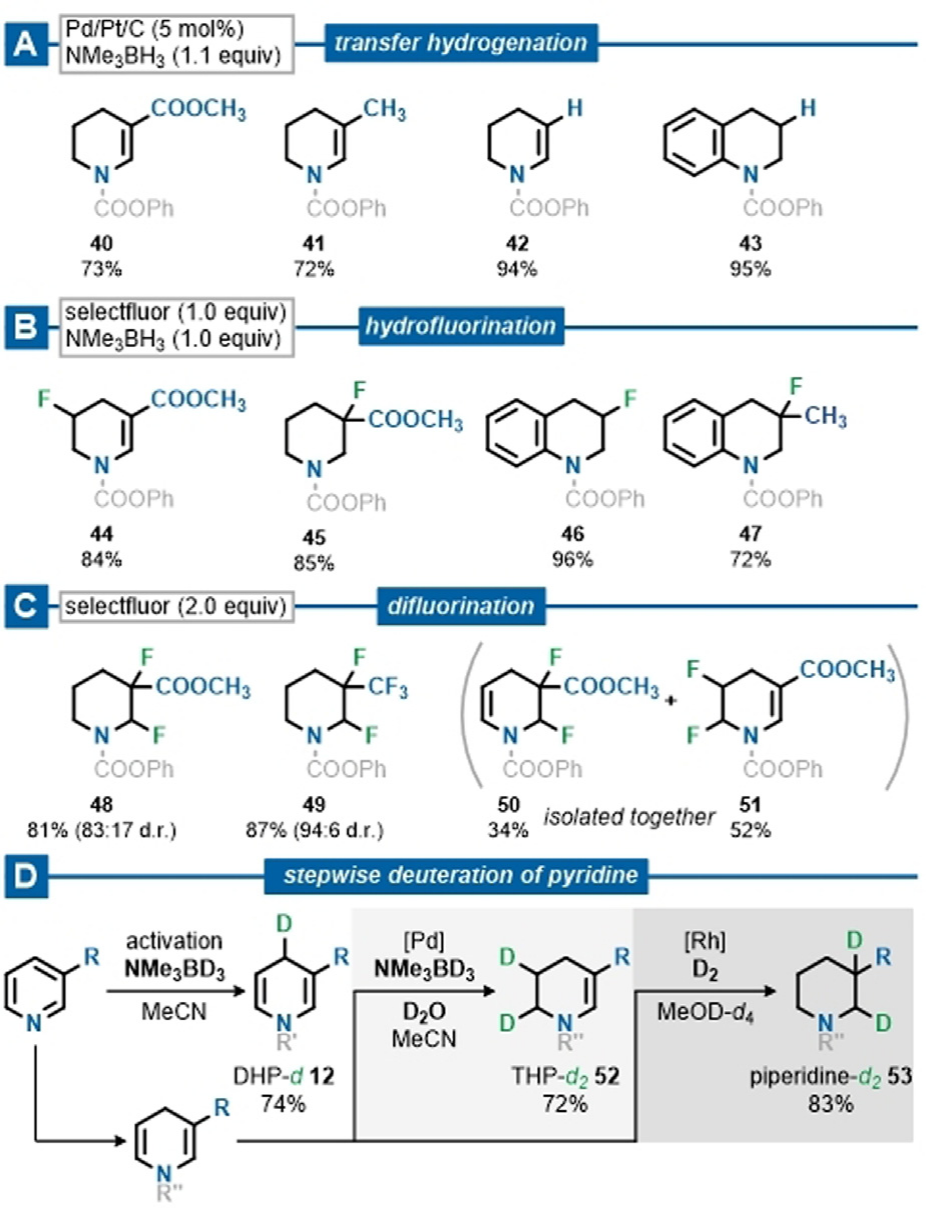
Regioselective functionalization of dihydro‐ and tetrahydro‐type N‐heterocycles. Isolated yields reported. R=COOCH_3_, R′=Tf, R′′=COOPh. Transfer hydrogenation (A) was carried out in situ after the dearomatization. For hydrofluorination (B) and difluorination (C) the isolated dihydro‐ or tetrahydro‐N‐heterocycle was used. In the stepwise saturation of pyridine (D), deuterated reagents were used in the last step of each sequence.

In conclusion, we have developed a straightforward method to access a variety of synthetically highly useful 1,4‐dihydropyridine‐ and 1,2‐dihydropyridine‐type motifs from readily available pyridines through direct dearomatization by amine borane. The setup is simple and can be carried out without the use of anhydrous solvent, oxygen exclusion or special equipment. The valuable building blocks were obtained in high yield and with high regioselectivity, and their synthetic utility was highlighted by exemplary stepwise hydrogenation and (hydro‐)fluorination with complete control of regioselectivity. We envision that this protocol will greatly simplify access to dihydropyridines and further advance their use in a variety of applications.

## Conflict of interest

The authors declare no conflict of interest.

## Supporting information

As a service to our authors and readers, this journal provides supporting information supplied by the authors. Such materials are peer reviewed and may be re‐organized for online delivery, but are not copy‐edited or typeset. Technical support issues arising from supporting information (other than missing files) should be addressed to the authors.

Supplementary
