# Supplementary material for: Substituted Dihydropyridine Synthesis by Dearomatization of Pyridines
Source: Angew Chem Int Ed Engl. 2021 May 10;60(25):13793–7. doi: 10.1002/anie.202104115 (PMC8252501; doi:10.1002/anie.202104115)
Supplement: Supplementary file 1 — Supplementary [file ANIE-60-13793-s001.pdf]

Angewandte Chemie
Eine Zeitschrift der Gesellschaft Deutscher Chemiker

Supporting Information

Substituted Dihydropyridine Synthesis by Dearomatization of Pyridines

*Arne Heusler[+], Julian Fliege[+], Tobias Wagener, and Frank Glorius**

anie_202104115_sm_miscellaneous_information.pdf

Table of Content

1. General Information ... S3
2. Investigation of the Dearomatization of Pyridines ... S4
   2.1. Optimization Studies ... S4
   2.2. Sensitivity Assessment ... S8
   2.3. Starting Material Synthesis ... S10
   2.4. Substrate Scope and Characterization Data ... S10
      General procedure A (GP-A) ... S10
      General Procedure B (GP-B) ... S11
   2.5. Stepwise Hydrogenation ... S25
      General procedure C (GP-C) ... S25
   2.6. Fluorination ... S27
      General procedure D (GP-D) ... S27
      General procedure E (GP-E) ... S27
   2.7. Deuterium Labeling ... S30
      Synthesis of NMe_3BD_3 ... S30
      Synthesis of piperidine derivative ... S31
3. Analytical Data ... S33
   3.1. X-Ray Data ... S33
   3.2. NMR Data ... S35
4. References ... S104

1. General Information

Unless otherwise noted, all reactions were carried out in oven dried glassware under an atmosphere of air without the use of Schlenk techniques. Liquid reagents were added with syringes or pipettes. Solids were weighted under air. Reaction temperatures are reported as the temperature of the heat transfer medium surrounding the vessel.

The following solvents were used from a solvent purification system (HPLC grade, dried via an alumina column under a positive argon pressure): *n*-hexane, THF, Et_2O, DMF and CH_2Cl_2. Additional anhydrous solvents (<50 ppm water) were purchased from *Acros Organics*, *Sigma-Aldrich* or *Carl Roth* and stored over molecular sieves under an argon atmosphere. The solvents (*n*-pentane, ethyl acetate, CH_2Cl_2, Et_2O) used for flash column chromatography were purified by distillation. The benzene-d_6 used for NMR analysis was degassed and stored over molecular sieves under an argon atmosphere.

All hydrogenation reactions (>1 bar) were carried out in *Berghof* High Pressure Reactors using hydrogen gas. All other hydrogenation reactions (at 1 bar) were carried out in 4 mL vials which were put in Schlenk tube which was connected to a balloon filled with hydrogen gas. Commercially available chemicals were purchased from *Acros Organics*, *Sigma-Aldrich*, *Alfa Aesar*, *ABCR*, *TCI Europe*, *Fluorochem UK* and *Combi-Blocks* and used as received unless otherwise stated.

Analytical thin layer chromatography (TLC) was performed on silica geld 60 F254 aluminum plates (*Merck*). TLC plates were visualized by exposure to short wave ultraviolet light (254 nm, 366 nm) and/or $KMnO_4$ (1 g $KMnO_4$, 6 g K_2CO_3 and 0.1 g KOH in 100 mL water) staining solution followed by heating. Flash column chromatography was performed either on Merck silica gel (40-63 µm mesh) or *EcoChrom MP* Alumina (Basic or Neutral) with a positive pressure of argon.

^{1}H-, ^{13}C- and ^{19}F-NMR spectra were recorded at room temperature on a *Bruker* Avance II 300 MHz or Avance II 400 MHz, *Agilent* DD2 500 MHz or *Agilent* DD2 600 MHz. Chemical shifts (δ) are given in ppm. The residual solvent signals were used as references and the chemical shifts were converted to the TMS scale ($CDCl_3$: δ_H = 7.26 ppm, δ_C = 77.16 ppm; C_6D_6: δ_H = 7.16 ppm, δ_C = 128.06 ppm). ^{19}F-NMR spectra were not calibrated by an internal reference. The multiplicities of the signals are reported as s (singlet), bs (broad singlet), d (doublet), t (triplet), q (quartet), p (pentet), hept (heptet) and m (multiplet). Coupling constants (*J*) are quoted in Hz.

GC-MS spectra were recorded on an *Agilent Technologies* 7890A GC-system with an *Agilent* 5975C VL MSD or an *Agilent* 5975 inert Mass Selective Detector (EI) and a HP-5MS column (0.25 mm · 30 m, film: 0.25 µm). Method: Initial temperature 50 °C, hold 3 min, increment 40 °C/min until 280 °C, hold 3 min.

GC-FID analysis was undertaken on an *Agilent Technologies* 6890A equipped with an HP-5 quartz column (0.32 mm · 30 m, film: 0.25 µm) using flame ionization detection. Method: Initial temperature 50 °C, hold 3 min, increment 15 °C/min until 280 °C.

ESI mass spectra were recorded on a *Thermo Fisher Scientific* Orbitrap LTQ XL or a *Thermo Fisher Scientific* Orbitrap Velos Pro. EI mass spectra were measured on a Trace 1310 with GC Exactive Orbitrap from *Thermo Fisher Scientific*.

X-Ray diffraction: Data sets for compounds **S1** and **40** were collected with a Bruker D8 Venture PHOTON III diffractometer. Programs used: data collection: APEX3 V2016.1-0[1]; cell refinement: SAINT V8.37A; data reduction: SAINT V8.37A; absorption correction, SADABS V2014/7; structure solution *SHELXT-2015*[2]; structure refinement *SHELXL-2015*[3] and graphics, *XP*[4]. *R*-values are given for observed reflections, and wR^2 values are given for all reflections.

2. Investigation of the Dearomatization of Pyridines

2.1. Optimization Studies

Methyl nicotinate was used as the model substrate for the investigation of dearomatization of activated pyridines by amine boranes. It was found that phenyl chloroformate (PhOCOCl) is a sufficient activating reagent for the subsequent reduction to dihydropyridine (DHP) **S1** by amine borane (Table S1). The major isomer was identified as 1,4-DHP by X-ray crystal analysis and NMR experiments.

Table S1: Selected optimization reactions with phenyl chloroformate as the activating reagent.

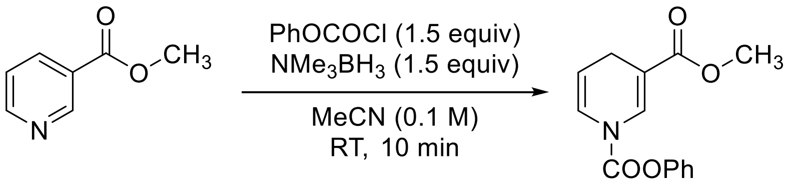

S1

entry	variation	yield [%]	ratio of DHPs
1	none	61	68:16:16
2	*n*-pentane instead of MeCN	44	62:12:25
3	–40 °C → RT	85	78:10:12
4	NH_3BH_3 instead of NMe_3BH_3	38	39:31:30
5	without NMe_3BH_3 or PhOCOCl	0	-

Reaction conditions: pyridine (0.1 mmol, 1.0 equiv), NMe_3BH_3 (0.15 mmol, 1.5 equiv), PhOCOCl (0.15 mmol, 1.5 equiv), MeCN (0.1 M) at room temperature for 10 min. Yields and ratios were determined by GC-FID analysis with mesitylene as internal standard. The first mentioned isomer is the 1,4-DHP. The other isomers were not determined.

Since those reaction conditions resulted in bad DHP ratios for other pyridines, other activating reagents were investigated. Among several reagents, triflic anhydride gave the best yield and ratio for the model substrate methyl nicotinate:

Table S2: Screening of different solvents.

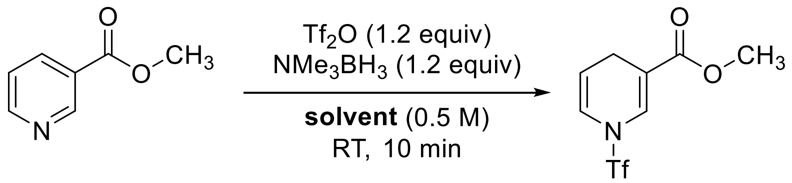

entry	solvent	yield [%]	ratio of DHPs
1	MeCN	98	84:16
2	MeOH	0	-
3	Aceton	72	80:20
4	THF	37	85:15
5	Et_2O	49	86:14

Reaction conditions: pyridine (0.2 mmol, 1.0 equiv), NMe_3BH_3 (0.24 mmol, 1.5 equiv), Tf_2O (0.24 mmol, 1.5 equiv), **solvent** (0.4 mL, 0.5 M) at room temperature for 10 min. Yields and ratios were determined by GC-FID analysis with mesitylene as internal standard. The first mentioned isomer is the 1,4-DHP. The other isomers were not determined.

Table S3: Screening of equivalents of triflic anhydride.

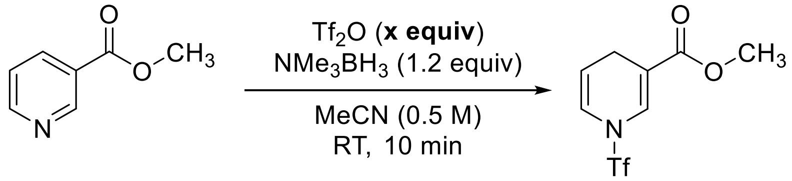

entry	equivalents Tf_2O	yield [%]	ratio of DHPs
1	0.5	44	84:16
2	1.0	91	85:15
3	1.2	98	84:16
4	2.0	62	92:8

Reaction conditions: pyridine (0.2 mmol, 1.0 equiv), NMe_3BH_3 (0.24 mmol, 1.5 equiv), Tf_2O (**x equiv**), MeCN (0.4 mL, 0.5 M) at room temperature for 10 min. Yields and ratios were determined by GC-FID analysis with mesitylene as internal standard. The first mentioned isomer is the 1,4-DHP. The other isomers were not determined.

Table S4: Screening of equivalents of amine borane.

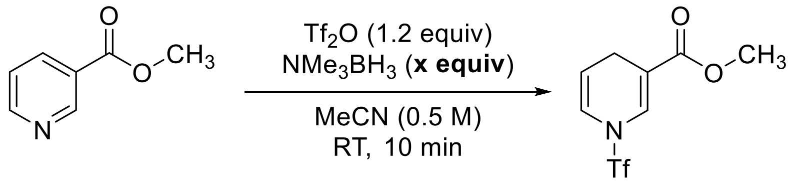

entry	equivalents NMe_3BH_3	yield [%]	ratio of DHPs
1	0.5	38	93:7

2	1.0	88	87:13
3	1.2	98	84:16
4	2.0	76	85:15

Reaction conditions: pyridine (0.2 mmol, 1.0 equiv), NMe_3BH_3 (**x equiv**), Tf_2O (0.24 mmol, 1.5 equiv), MeCN (0.4 mL, 0.5 M) at room temperature for 10 min. Yields and ratios were determined by GC-FID analysis with mesitylene as internal standard. The first mentioned isomer is the 1,4-DHP. The other isomers were not determined.

Table S5: Screening solvent concentration.

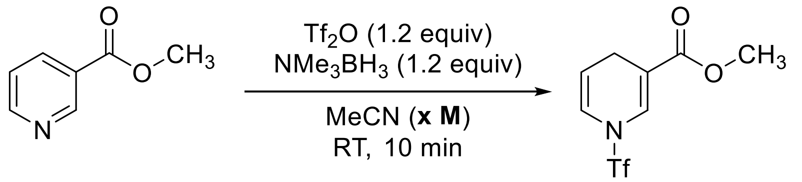

entry	conventration	yield [%]	ratio of DHPs
1	0.1	100	86:14
2	0.5	98	84:16
3	1.0	97	84:16

Reaction conditions: pyridine (0.2 mmol, 1.0 equiv), NMe_3BH_3 (0.24 mmol, 1.5 equiv), Tf_2O (0.24 mmol, 1.5 equiv), MeCN (**x M**) at room temperature for 10 min. Yields and ratios were determined by GC-FID analysis with mesitylene as internal standard. The first mentioned isomer is the 1,4-DHP. The other isomers were not determined.

Table S6: Screening of different amine boranes.

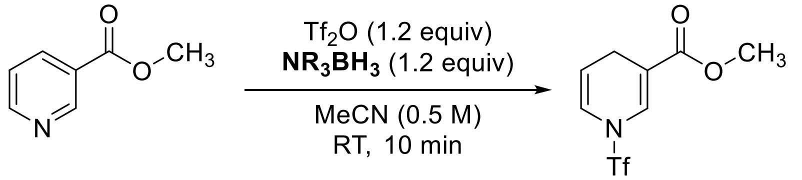

entry	amine borane	yield [%]	ratio of DHPs
1	NMe_3BH_3	98	84:16
2	NH_3BH_3	71	72:28
3	NEt_3BH_3	100	88:12
4	NMe_2HBH_3	84	83:17
5	Morpholine-BH_3	84	79:21

Reaction conditions: pyridine (0.2 mmol, 1.0 equiv), **amine borane** (0.24 mmol, 1.5 equiv), Tf_2O (0.24 mmol, 1.5 equiv), MeCN (0.4 mL, 0.5 M) at room temperature for 10 min. Yields and ratios were determined by GC-FID analysis with mesitylene as internal standard. The first mentioned isomer is the 1,4-DHP. The other isomers were not determined.

Table S7: Screening of the reaction temperature.

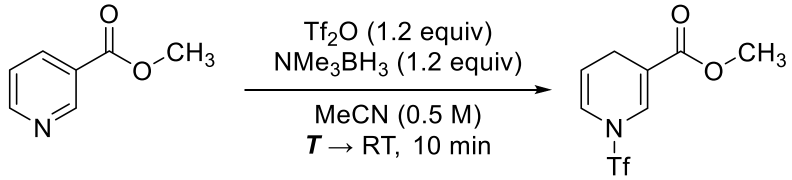

entry	temperature	yield [%]	ratio of DHPs
1	RT	98	84:16
2	0 °C	100	87:13
3	–20 °C	100	90:10
4	–40 °C	76	92:8

Reaction conditions: pyridine (0.2 mmol, 1.0 equiv), NMe_3BH_3 (0.24 mmol, 1.5 equiv), Tf_2O (0.24 mmol, 1.5 equiv), MeCN (0.4 mL, 0.5 M) at ***T*** for 5 min, followed by 5 min at room temperature. Yields and ratios were determined by GC-FID analysis with mesitylene as internal standard. The first mentioned isomer is the 1,4-DHP. The other isomers were not determined.

Table S8: Selected examples of the evaluation of reaction conditions.

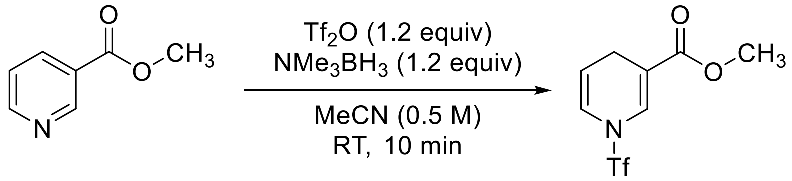

entry	variation	yield [%]	ratio of DHPs
1	none	98%	84:16
2	without NMe_3BH_3	-	n/a
3	without Tf_2O	-	n/a
4	NMe_3BH_3 (0.5 equiv)	38%	93:7
5	NH_3BH_3 (1.2 equiv)	71%	72:28
6	Ac_2O instead of Tf_2O	traces	80:20
7	Boc_2O instead of Tf_2O	-	n/a
8	TFA instead of Tf_2O	-	n/a
9	PhOCOCl instead of Tf_2O	91%	68:32
**10**	**-20 °C to RT, 15 min**	**99%**	**90:10**

Reaction conditions: pyridine (0.5 mmol, 1.0 equiv), NMe_3BH_3 (1.2 equiv), Tf_2O (1.2 equiv), MeCN (1.0 mL, 0.5 M) at RT for 5 min, followed by 5 min at room temperature. Yields and ratios were determined by GC-FID analysis with mesitylene as internal standard. The first mentioned isomer is the 1,4-DHP. The other isomers were not determined.

2.2. Sensitivity Assessment

The sensitivity screen of general procedure A was performed following a literature procedure by Glorius and coworkers.[5] A description of the experiments included in the assessment is given in Table S9. Volume changes due to solvation of starting materials were neglected. All reactions were carried out using Schlenk tubes with an argon flow. Both stock solutions were cooled to –20 °C. Additives were added to sock solution I if needed. Tf_2O was added to stock solution I and then stock solution II was added to stock solution I. For the big scale reaction, substrate and amine borane (NMe_3BH_3) were cooled to –20 °C in 4.0 mL MeCN each. Tf_2O was added to the substrate and the amine borane in MeCN was also added to the substrate. The reactions were stirred at –20 °C for 5 minutes and then for 5 minutes at room temperature. The reactions were analyzed by GC-FID using mesitylene as internal standard. The deviation from the yield (and the regioisomeric ratio) of the 'standard' experiment was calculated for each experiment (Table S10 and Table S11). The deviation values are plotted in a separate radar diagram (Figure S1 and Figure S2).

Standard conditions: n = 0.2 mmol, c = 0.5 M, V(MeCN) = 0.4 mL, T = –20 °C, then RT

Stock solution I (SL I): Substrate (5.55 mmol, 762 mg, 1.0 eq.), mesitylene (5.55 mmol, 668 mg, 1.0 eq.), MeCN (5.0 mL).

Stock solution II (SL II): NMe_3BH_3 (6.66 mmol, 486 mg, 1.2 eq), MeCN (5.0 mL).

Big scale: Substrate (4.0 mmol, 549 mg, 1.0 eq.), NMe_3BH_3 (4.8 mmol, 350 mg, 1.2 eq.), Tf_2O (4.8 mmol, 0.81 mL, 1.2 eq), MeCN (8.0 mL).

Table S9: Conditions for the Sensitivity Screen (Stock Solution = SL).

Entry	Experiment	Preparation
0	Standard	180 µL of SL I + 40 µL Tf_2O + 180 µL SL II + 40 µL MeCN
1	Low c	180 µL of SL I + 40 µL Tf_2O + 180 µL SL II + 80 µL MeCN
2	High c	180 µL of SL I + 40 µL Tf_2O + 180 µL SL II
3	Low T	180 µL of SL I + 40 µL Tf_2O + 180 µL SL II + 40 µL MeCN + T = –30 °C
4	High T	180 µL of SL I + 40 µL Tf_2O + 180 µL SL II + 40 µL MeCN + T = –10 °C
5	Low O_2	180 µL of SL I + 40 µL Tf_2O + 180 µL SL II + 40 µL MeCN + degassed stock solutions
6	High O_2	180 µL of SL I + 40 µL Tf_2O + 180 µL SL II + 40 µL MeCN + no argon flow
7	High H_2O	180 µL of SL I + 40 µL Tf_2O + 180 µL SL II + 40 µL MeCN + 4 µL H_2O
8	Big scale	Big Scale conditions

Table S10: Results of the Sensitivity Screen with respect to the yield.

Entry	Experiment	Yield [%]	Deviation [%]
0	Standard	94	-
1	Low *c*	93	-1
2	High *c*	95	1
3	Low *T*	94	0
4	High *T*	95	1
5	Low O_2	94	0
6	High O_2	84	-11
7	High H_2O	0	-100
8	Big scale	95	1

Table S11: Results of the Sensitivity Screen with respect to the ratio of the isomers.

Entry	Experiment	Share of main isomer [%]	Deviation [%]
0	Standard	90	-
1	Low *c*	90	0
2	High *c*	90	0
3	Low *T*	91	1
4	High *T*	88	-2
5	Low O_2	90	0
6	High O_2	89	-1
7	High H_2O	0	-100
8	Big scale	90	0

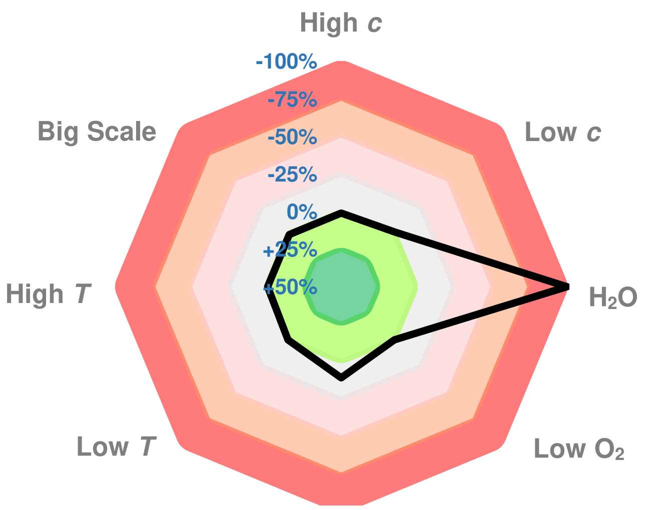

Figure S1: Results of the sensitivity assessment with respect to the yield.

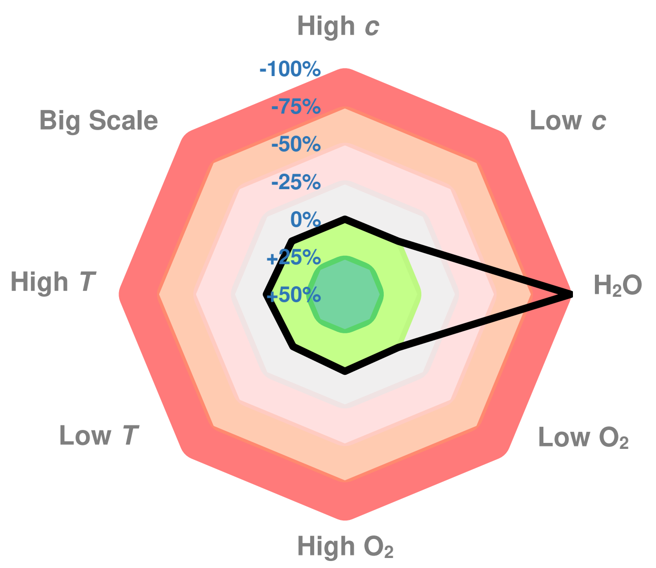

Figure S2: Results of the sensitivity assessment with respect to the ratio of the isomers.

2.3. Starting Material Synthesis

Unless otherwise stated, starting materials are commercially available and were purchased. The following substrates were synthesized by following literature procedures:

Methyl 5-fluoronicotinate was synthesized by following a literature procedure.[6] The analytical data was in agreement with the data reported in the literature.

3-Fluoro-5-phenylpyridine was synthesized by following a literature procedure.[6] The analytical data was in agreement with the data reported in the literature.

4-Fluoro-3-(trimethylsilyl)pyridine was synthesized by following a literature procedure.[7] The analytical data was in agreement with the data reported in the literature.

3-Fluoro-4-(phenylthio)pyridine was synthesized by following a literature procedure.[8] The analytical data was in agreement with the data reported in the literature.

3-Fluoro-4-phenylpyridine was synthesized by following a literature procedure.[6] The analytical data was in agreement with the data reported in the literature.

2.4. Substrate Scope and Characterization Data

General procedure A (GP-A)

Pyridine derivative (0.5 mmol, 1.0 eq.) was dissolved in MeCN (0.5 mL) under a stream of argon and cooled to –20 °C using an ice/NaCl-cooling bath. NMe_3BH_3 (44 mg, 0.60 mmol, 1.2 eq.) was also dissolved in MeCN (0.5 mL) under a stream of argon and cooled to –20 °C. After 5 minutes of cooling, Tf_2O (101 µL, 0.60 mmol, 1.2 eq.) was added to the pyridine and directly afterwards, the NMe_3BH_3-solution was added to the

pyridine. The solution was stirred at –20 °C for 5 minutes and afterwards at room temperature for an additional 5 minutes. The product was directly purified by column chromatography (neutral alumina, activity grade **III**).

General Procedure B (GP-B)

The N-heterocycle (0.5 mmol, 1.0 eq.) was dissolved in MeCN (0.5 mL) and PhOCOCl (75 µL, 0.60 mmol, 1.2 eq) was added. The mixture was cooled to –40 °C and stirred for 5 minutes. NMe_3BH_3 (40 mg, 0.55 mmol, 1.1 eq.) was dissolved in MeCN (0.5 mL) and the mixture was transferred to the activated N-heterocycle. The reaction was stirred for 5 minutes at –40 °C and for additional 5 minutes at room temperature. The product was directly purified by column chromatography on silica gel.

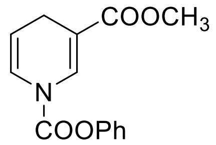

3-Methyl 1-phenyl pyridine-1,3(4H)-dicarboxylate (**S1**): The title compound was synthesized from methyl nicotinate (0.50 mmol, 69 mg, 1.0 equiv) using GP-B. The product was purified by column chromatography (neutral alumina, activity grade **III**, pentane/ethyl acetate 95:5 to 90:10) to afford **S1** as a white solid (115 mg, 88% yield, 79:21 ratio of 1,4-DHP vs. other DHPs).

$\mathbf{R_f}$ (pentane/ethyl acetate 90:10) 0.28. **^{1}H NMR** (599 MHz, benzene-d_6, T = 338 K) δ 8.11 (s, 1H), 7.03 – 6.98 (m, 2H), 6.96 – 6.91 (m, 2H), 6.91 – 6.86 (m, 1H), 6.71 (s, 1H), 4.86 – 4.61 (m, 1H), 3.42 (s, 3H), 3.08 – 2.89 (m, 2H). **^{13}C{^{1}H} NMR** (151 MHz, benzene-d_6, T = 338 K) δ 166.7, 151.3, 149.5, 132.8, 129.5, 128.3, 126.1, 122.7, 121.6, 108.7, 51.1, 22.8.

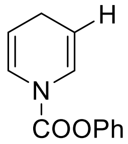

Phenyl pyridine-1(4H)-carboxylate (**1**): The title compound was synthesized from pyridine (0.50 mmol, 40 µL, 1.0 equiv) using GP-B. The product was purified by column chromatography (neutral alumina, activity grade **III**, pentane/ethyl acetate 95:5) to afford **1** as a white solid (97 mg, 96% yield, 90:10 ratio of 1,4-DHP vs. 1,2-DHP).

$\mathbf{R_f}$ (pentane/ethyl acetate 90:10) 0.61. **^{1}H NMR** (400 MHz, benzene-d_6) δ 7.04 – 6.99 (m, 3H), 6.98 – 6.95 (m, 2H), 6.91 – 6.85 (m, 1H), 6.77 – 6.73 (m, 1H), 4.68 – 4.50 (m, 2H), 2.54 – 2.42 (m, 2H). **^{13}C{^{1}H} NMR** (101 MHz, benzene-d_6) δ 151.4, 149.8, 129.4, 125.8, 124.0, 123.8, 122.0, 22.6. **HRMS** (ESI) m/z calc. for $C_{12}H_{11}NO_2Na$ $[M+Na]^+$ 224.06820, found 224.06786.

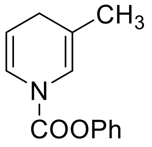

Phenyl 3-methylpyridine-1(4H)-carboxylate (**2**): The title compound was synthesized from 3-methyl pyridine (0.50 mmol, 49 µL, 1.0 equiv) using GP-B. The

product was purified by column chromatography (neutral alumina, activity grade III, pentane/ethyl acetate 99:1) to afford **2** as a colorless oil (81 mg, 76% yield, 83:17 ratio of 1,4-DHP vs. other DHPs).

(Two rotamers are observed due to the dynamic phenyl carboxylate group.)

R_f (pentane/ethyl acetate 90:10) 0.21. **^{1}H NMR** (400 MHz, benzene-*d*$_6$) δ 7.14 – 6.98 (m, 5H), 6.96 – 6.86 (m, 1H), 6.85 – 6.69 (m, 1H), 4.75 – 4.59 (m, 1H), 2.44 – 2.37 (m, 2H), 1.33 – 1.25 (m, 3H). **^{13}C{^{1}H} NMR** (101 MHz, benzene-*d*$_6$) δ 151.6, 149.9, 129.8, 125.7, 123.4, 122.0, 118.7, 115.4, 106.3, 28.1, 20.4. **HRMS** (ESI) m/z calc. for $C_{13}H_{13}NO_2Na$ [M+Na]$^+$ 238.08385, found 238.08342.

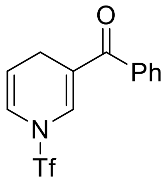

Phenyl(1-((trifluoromethyl)sulfonyl)-1,4-dihydropyridin-3-yl)methanone (**3**): The title compound was synthesized from phenyl(pyridin-3-yl)methanone (0.50 mmol, 92 mg, 1.0 equiv) using GP-A. The product was purified by column chromatography (pentane) to afford **3** as a white solid (121 mg, 76% yield, 82:18 ratio of 1,4-DHP vs. other DHPs).

(Product is mixture of both isomers. Signals are reported for main isomer.)

R_f (pentane/ethyl acetate 80:20) 0.59. **^{1}H NMR** (400 MHz, benzene-*d*$_6$) δ 7.50 – 7.44 (m, 2H), 7.14 – 7.10 (m, 1H), 7.05 – 7.01 (m, 1H), 7.01 – 6.95 (m, 2H), 6.08 – 5.78 (m, 1H), 4.52 (dt, J = 8.3, 3.6 Hz, 1H), 2.93 – 2.68 (m, 2H). **^{13}C{^{1}H} NMR** (101 MHz, benzene-*d*$_6$) δ 193.4, 137.6, 133.8, 132.1, 129.0, 128.7, 123.6, 120.3, 120.0 (q, J = 324.7 Hz), 111.7, 21.7. **^{19}F{^{1}H} NMR** (377 MHz, benzene-*d*$_6$) δ -75.3. **GC** (EI) m/z calc. for $C_{13}H_{10}F_3NO_3S$ [M]$^+$ 317.0, found 316.9.

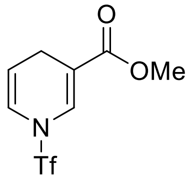

Methyl 1-((trifluoromethyl)sulfonyl)-1,4-dihydropyridine-3-carboxylate (**4**): The title compound was synthesized from methyl nicotinate (0.50 mmol, 69 mg, 1.0 equiv) using GP-A. The product was purified by column chromatography (pentane/diethylether 90:10) to afford **4** as a colorless oil (99 mg, 73% yield, 90:10 ratio of 1,4-DHP vs. other DHPs).

R_f (pentane/diethylether 80:20) 0.54. **^{1}H NMR** (400 MHz, benzene-*d*$_6$) δ 7.47 (s, 1H), 6.10 – 5.90 (m, 1H), 4.55 – 4.35 (m, 1H), 3.24 (s, 3H), 2.68 – 2.51 (m, 2H). **^{13}C{^{1}H} NMR** (101 MHz, benzene-*d*$_6$) δ 165.2, 130.3, 120.5, 120.0 (q, J = 324.5 Hz), 112.7, 111.0, 51.5, 21.8. **^{19}F{^{1}H} NMR** (377 MHz, benzene-*d*$_6$) δ -75.3. **HRMS** (EI) m/z calc. for $C_8H_7F_3NO_4S$ [M]$^+$ 270.0042, found 270.0051.

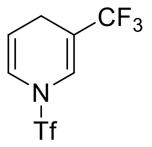

3-(Trifluoromethyl)-1-((trifluoromethyl)sulfonyl)-1,4-dihydropyridine (**5**): The title compound was synthesized from 3-(trifluoromethyl)pyridine (0.50 mmol, 58 µL, 1.0 equiv) using GP-A. The product was purified by column chromatography (pentane) to afford **5** as a colorless oil (111 mg, 78% yield, 81:19 ratio of 1,4-DHP vs. other DHPs).

R$_f$ (pentane/diethylether 80:20) 0.44. **^{1}H NMR** (400 MHz, benzene-*d*$_6$) δ 6.83 (s, 1H), 5.85 (dd, *J* = 8.2, 2.3 Hz, 1H), 4.29 – 4.07 (m, 1H), 2.35 – 2.01 (m, 3H). **^{13}C{^{1}H} NMR** (101 MHz, benzene-*d*$_6$) δ 124.8, 124.4 (q, *J* = 7.4 Hz), 122.1, 120.8, 112.0, 119.9 (q, *J* = 324.5 Hz), 108.9, 19.6. **^{19}F{^{1}H} NMR** (377 MHz, benzene-*d*$_6$) δ -70.4, -75.3. **HRMS** (EI) m/z calc. for $C_7H_5F_6NO_2S$ [M]$^+$ 280.99397, found 280.99395.

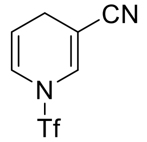

1-((Trifluoromethyl)sulfonyl)-1,4-dihydropyridine-3-carbonitrile (**6**): The title compound was synthesized from 3-pyridinecarbonitrile (0.50 mmol, 52 mg, 1.0 equiv) using GP-A. The product was purified by column chromatography (pentane/diethylether 80:20) to afford **6** as a colorless oil (81 mg, 67% yield, 84:16 ratio of 1,4-DHP vs. other DHP).

R$_f$ (pentane/diethylether 80:20) 0.34. **^{1}H NMR** (599 MHz, benzene-*d*$_6$) δ 6.52 (d, J = 2.2 Hz, 1H), 5.71 (dd, J = 8.5, 2.1 Hz, 1H), 4.01 (dt, J = 8.5, 3.6 Hz, 1H), 1.97 – 1.83 (m, 3H). **^{13}C{^{1}H} NMR** (151 MHz, benzene-*d*$_6$) δ 132.9, 120.3, 119.7 (q, *J* = 324.5 Hz), 116.6, 108.7, 95.0, 22.9. **^{19}F{^{1}H} NMR** (564 MHz, benzene-*d*$_6$) δ -75.3. **HRMS** (EI) m/z calc. for $C_7H_5F_3N_2O_2S$ [M]$^+$ 238.0018, found 238.0013.

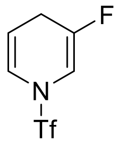

3-Fluoro-1-((trifluoromethyl)sulfonyl)-1,4-dihydropyridine (**7**): The title compound was synthesized from 3-fluoropyridine (0.50 mmol, 43 µL, 1.0 equiv) using GP-A. The product was purified by column chromatography (pentane) to afford **7** as a colorless oil (55 mg, 48% yield, 79:24 ratio of 1,4 DHP vs. other DHPs).

(Slow decomposition during the measurement of the NMR sample was observed.)

R$_f$ (pentane) 0.52. **^{1}H NMR** (400 MHz, benzene-*d*$_6$) δ 6.33 (m, 1H), 5.99 – 5.90 (m, 1H), 4.26 – 4.13 (m, 1H), 2.27 – 2.17 (m, 2H). **^{13}C{^{1}H} NMR** (101 MHz, benzene-*d*$_6$) δ 120.9 (d, *J* = 2.3 Hz), 119.8 (q, *J* = 325.8 Hz), 119.6 (d, *J* = 4.4 Hz), 107.9 (d, *J* = 14.1 Hz), 106.9 (d, *J* = 45.4 Hz), 23.7 (d, *J* = 25.2 Hz). **^{19}F{^{1}H} NMR** (377 MHz, benzene-

d_6) δ -70.5 – -77.3 (m), -123.3 – -130.3 (m). **HRMS** (EI) m/z calc. for $C_6H_5F_4NO_2S$ $[M]^+$ 230.99716, found 230.99728.

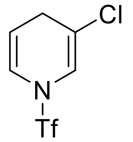

3-Chloro-1-((trifluoromethyl)sulfonyl)-1,4-dihydropyridine (**8**): The title compound was synthesized from 3-chloropyridine (0.50 mmol, 57 mg, 1.0 equiv) using GP-A. The product was purified by column chromatography (pentane) to afford **8** as a colorless oil (76 mg, 61% yield, 83:17 ratio of 1,4 DHP vs. other DHPs).

(One signal in $^{13}C\{^1H\}$ NMR is missing.)

R_f (pentane) 0.50. **^{1}H NMR** (400 MHz, benzene-d_6) δ 6.51 – 6.46 (m, 1H), 5.94 (d, J = 8.3 Hz, 1H), 4.13 (dt, J = 8.3, 3.6 Hz, 1H), 2.41 – 2.15 (m, 2H). **^{13}C{^{1}H} NMR** (101 MHz, benzene-d_6) δ 120.8, 120.1 (q, J = 324.2 Hz), 119.4, 108.5, 29.5. **^{19}F{^{1}H} NMR** (377 MHz, benzene-d_6) δ -74.9. **HRMS** (EI) m/z calc. for $C_6H_5F_3ClNO_2S$ $[M]^+$ 246.9676, found 246.9675.

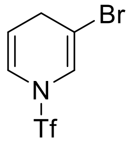

3-Bromo-1-((trifluoromethyl)sulfonyl)-1,4-dihydropyridine (**9**): The title compound was synthesized from 3-bromopyridine (0.50 mmol, 79 mg, 1.0 equiv) using GP-A. The product was purified by column chromatography (pentane) to afford **9** as a colorless oil (76 mg, 66% yield, 82:18 ratio of 1,4-DHP vs. other DHPs).

R_f (pentane) 0.42. **^{1}H NMR** (400 MHz, benzene-d_6) δ 6.70 – 6.59 (m, 1H), 6.02 – 5.92 (m, 1H), 4.13 – 4.03 (m, 1H), 2.45 – 2.34 (m, 2H). **^{13}C{^{1}H} NMR** (101 MHz, benzene-d_6) δ 121.5, 120.7, 120.1 (q, J = 325.2 Hz), 108.7, 107.6, 31.2. **^{19}F{^{1}H} NMR** (377 MHz, benzene-d_6) δ -75.0. **HRMS** (EI) m/z calc. for $C_6H_5F_3BrNO_2S$ $[M]^+$ 290.9171, found 290.9175.

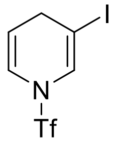

3-Iodo-1-((trifluoromethyl)sulfonyl)-1,4-dihydropyridine (**10**): The title compound was synthesized from 3-iodopyridine (0.50 mmol, 103 mg, 1.0 equiv) using GP-A. The product was purified by column chromatography (pentane) to afford **10** as a yellow oil (146 mg, 86% yield, 93:7 ratio of 1,4-DHP vs. other DHPs).

(Slow decomposition during the measurement of the NMR sample was observed.)

R_f (pentane) 0.35. **^{1}H NMR** (400 MHz, benzene-d_6) δ 6.76 (s, 1H), 6.03 (dd, J = 8.5, 2.0 Hz, 1H), 3.97 (dt, J = 8.3, 3.5 Hz, 1H), 2.52 – 2.38 (m, 2H). **^{13}C{^{1}H} NMR** (101 MHz, benzene-d_6) δ 126.32 123.6, 120.5, 120.0 (q, J = 325.1 Hz), 108.9, 34.5. **^{19}F{^{1}H} NMR**

(377 MHz, benzene-d_6) δ -75.0. **HRMS** (EI) m/z calc. for $C_6H_5F_3NO_2SI$ $[M]^+$ 338.90323, found 338.90299.

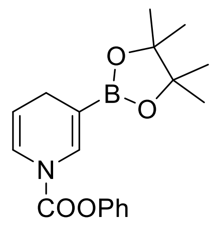

Phenyl 3-(4,4,5,5-tetramethyl-1,3,2-dioxaborolan-2-yl)pyridine-1(4H)-carboxylate (**11**): The title compound was synthesized from 3-pyridineboronic acid pinacol ester (0.50 mmol, 103 mg, 1.0 equiv) using GP-B. The product was purified by column chromatography (neutral alumina, activity grade **III**, pentane/ethyl acetate 99:1) to afford **11** as a colorless oil (124 mg, 76% yield, 84:16 ratio of 1,4-DHP vs. other DHPs).

R_f (pentane/ethyl acetate 90:10) 0.31. **^{1}H NMR** (400 MHz, benzene-d_6) δ 8.08 – 7.88 (m, 1H), 7.05 – 6.72 (m, 6H), 4.81 – 4.65 (m, 1H), 3.12 – 2.98 (m, 2H), 1.10 – 1.00 (m, 12H). **^{11}B NMR** (128 MHz, benzene-d_6) δ 30.9. **^{13}C{^{1}H} NMR** (101 MHz, benzene-d_6) δ 151.3, 149.8, 134.6, 129.3, 125.7, 123.5, 122.0, 108.9, 108.0, 83.5, 24.9, 23.6. **HRMS** (ESI) m/z calc. for $C_{18}H_{22}NO_4BNa$ $[M+Na]^+$ 350.15374, found 350.15323.

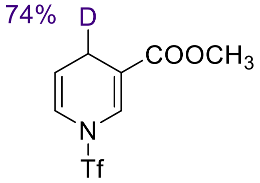

Methyl 1-((trifluoromethyl)sulfonyl)-1,4-dihydropyridine-3-carboxylate-4-d (**12**): The title compound was synthesized from methyl nicotinate (0.30 mmol, 41 mg, 1.0 equiv) using GP-A with NMe_3BD_3 (27 mg, 0.36 mmol, 1.2 equiv). The product was purified by column chromatography (pentane/diethylether 90:10) to afford **12** as a colorless oil (57 mg, 70% yield, 90:10 ratio of 1,4 DHP vs. other DHP).

(*Degree of Deuteration was assigned through ^{1}H NMR and ^{2}H NMR)*

R_f (pentane/diethylether 80:20) 0.54. **^{1}H NMR** (400 MHz, benzene-d_6) δ 7.60 – 7.39 (m, 1H), 6.10 – 5.88 (m, 1H), 4.54 – 4.13 (m, 1H), 3.23 (s, 3H), 2.68 – 2.25 (m, 1H). **^{13}C{^{1}H} NMR** (101 MHz, benzene-d_6) δ 165.2, 130.3, 120.5, 120.0 (q, J = 324.7 Hz), 112.6, 110.9, 51.5, 21.8. **^{19}F{^{1}H} NMR** (377 MHz, benzene-d_6) δ -75.3. **HRMS** (ESI) m/z calc. for $C_8H_8NO_4SDF_3$ $[M+H]^+$ 273.0262, found 273.0262.

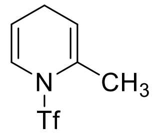

6-Methyl-1-((trifluoromethyl)sulfonyl)-1,4-dihydropyridine (**13**): The title compound was synthesized from 2-methylpyridine (0.50 mmol, 49 µL, 1.0 equiv) using GP-A. The product was purified by column chromatography (pentane) to afford **13** as a colorless oil (16 mg, 14% yield, 96:4 ratio of 1,4-DHP vs. other DHPs).

R$_f$ (pentane) 0.16. **^{1}H NMR** (400 MHz, benzene-*d*$_6$) δ 6.34 – 6.29 (m, 1H), 4.67 – 4.60 (m, 1H), 4.41 – 4.35 (m, 1H), 2.07 – 2.01 (m, 2H), 1.86 – 1.81 (m, 3H). **^{13}C{^{1}H} NMR** (101 MHz, benzene-*d*$_6$) δ 132.4, 125.6, 120.8 (q, *J* = 326.7 Hz), 113.6, 113.3, 22.7, 21.1. **^{19}F{^{1}H} NMR** (377 MHz, benzene-*d*$_6$) δ -74.2. **HRMS** (EI) m/z calc. for $C_7H_8NO_2SF_3$ [M]$^+$ 227.02224, found 227.02218.

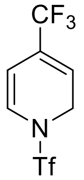

4-(Trifluoromethyl)-1-((trifluoromethyl)sulfonyl)-1,2-dihydropyridine (**14**): The title compound was synthesized from 4-(trifluoromethyl)pyridine (0.50 mmol, 58 µL, 1.0 equiv) using GP-A. The product was purified by column chromatography (pentane) to afford **14** as a colorless oil (113 mg, 80% yield, 99:1 ratio of 1,2-DHP vs. 1,4-DHP).

R$_f$ (pentane) 0.31. **^{1}H NMR** (400 MHz, benzene-*d*$_6$) δ 6.17 – 5.81 (m, 1H), 5.11 – 4.87 (m, 2H), 3.64 – 3.44 (m, 2H). **^{13}C{^{1}H} NMR** (101 MHz, benzene-*d*$_6$) δ 126.7, 126.4 – 126.0 (m), 122.5 (q, *J* = 271.5 Hz), 120.2 (q, *J* = 324.0 Hz), 119.8 (q, *J* = 5.8 Hz), 105.93 – 105.27 (m), 44.1. **^{19}F{^{1}H} NMR** (377 MHz, benzene-*d*$_6$) δ -68.7 – -69.0 (m), -75.5 – -76.2 (m). **HRMS** (EI) m/z calc. for $C_7H_5F_6NO_2S$ [M]$^+$ 280.99397, found 280.99463.

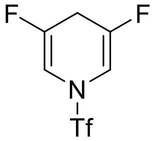

3,5-Difluoro-1-((trifluoromethyl)sulfonyl)-1,4-dihydropyridine (**15**): The title compound was synthesized from 3,5-difluoropyridine (0.50 mmol, 46 µL, 1.0 equiv) using GP-A. The product was purified by column chromatography (pentane) to afford **15** as a colorless oil (100 mg, 80% yield, 99:1 ratio of 1,4-DHP vs. 1,2-DHP).

R$_f$ (pentane) 0.51. **^{1}H NMR** (400 MHz, benzene-*d*$_6$) δ 6.36 – 5.95 (m, 2H), 2.52 – 2.23 (m, 2H). **^{13}C{^{1}H} NMR** (101 MHz, benzene-*d*$_6$) δ 148.7 (dd, *J* = 256.1, 22.9 Hz), 120.0 (q, *J* = 326.1 Hz), 107.8 – 106.5 (m), 26.9 (t, *J* = 28.1 Hz). **^{19}F{^{1}H} NMR** (377 MHz, benzene-*d*$_6$) δ -74.0, -129.2. **GC** (EI) m/z calc. for $C_6H_4F_5NO_2S$ [M]$^+$ 249.0, found 248.9.

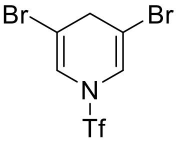

5-Dibromo-1-((trifluoromethyl)sulfonyl)-1,4-dihydropyridine (**16**): The title compound was synthesized from 3,5-dibromopyridine (0.50 mmol, 118 mg, 1.0 equiv) using GP-A. The product was purified by column chromatography (pentane) to afford **16** as a colorless oil (147 mg, 79% yield, 92:8 ratio of 1,4-DHP vs. 1,2-DHP).

R$_f$ (pentane) 0.55. **^{1}H NMR** (400 MHz, benzene-*d*$_6$) δ 6.49 (m, 2H), 2.63 (m, 2H). **^{13}C{^{1}H} NMR** (101 MHz, benzene-*d*$_6$) δ 120.5, 119.7 (q, *J* = 325.1 Hz), 105.5, 39.7.

$^{19}F\{^1H\}$ NMR (377 MHz, benzene-d_6) δ -74.9. **HRMS** (EI) m/z calc. for $C_6H_4F_3Br_2NO_2S$ $[M]^+$ 370.82554, found 370.82560.

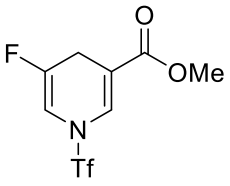

Methyl 5-fluoro-1-((trifluoromethyl)sulfonyl)-1,4-dihydropyridine-3-carboxylate (**17**): The title compound was synthesized from methyl 5-fluoronicotinate (0.50 mmol, 78 mg, 1.0 equiv) using GP-A. The product was purified by column chromatography (pentane/diethylether 90:10) to afford **17** as a colorless oil (116 mg, 80% yield, 93:7 ratio of 1,4-DHP vs. other DHPs).

(Product is mixture of both isomers. Signals are reported for main isomer.)

R_f (pentane/diethylether 80:20) 0.77. **^1H NMR** (400 MHz, benzene-d_6) δ 7.37 – 7.29 (m, 1H), 6.28 – 6.21 (m, 1H), 3.24 (s, 3H), 2.80 – 2.71 (m, 2H). **$^{13}C\{^1H\}$ NMR** (101 MHz, benzene-d_6) δ **$^{13}C\{^1H\}$ NMR** (126 MHz, benzene-d_6) 164.4, 151.5 (d, J = 256.0 Hz), 129.5, 119.8 (q, J = 324.7 Hz), 112.4 (d, J = 14.8 Hz), 106.3 (d, J = 45.8 H.z), 51.7, 24.6 (d, J = 26.9 Hz). **$^{19}F\{^1H\}$ NMR** (377 MHz, benzene-d_6) δ -75.0, -126.3. **HRMS** (EI) m/z calc. for $C_8H_7F_4NO_4S$ $[M]^+$ 289.0026, found 289.0028.

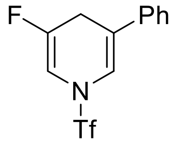

3-Fluoro-5-phenyl-1-((trifluoromethyl)sulfonyl)-1,4-dihydropyridine (**18**): The title compound was synthesized from 3-fluoro-5-phenylpyridine (0.3 mmol, 52 mg, 1.0 equiv) using GP-A. The product was purified by column chromatography (pentane) to afford **18** as a yellow oil (66 mg, 90% yield, 91:9 ratio of 1,4-DHP vs. other DHPs).

(Product is mixture of both isomers. Signals are reported for main isomer.)

R_f (pentane/ethyl acetate 90:10) 0.62. **^1H NMR** (400 MHz, benzene-d_6) δ 7.08 – 6.94 (m, 3H), 6.83 – 6.76 (m, 2H), 6.74 – 6.71 (m, 1H), 6.52 – 6.40 (m, 1H), 2.85 – 2.62 (m, 2H). **$^{13}C\{^1H\}$ NMR** (101 MHz, benzene-d_6) δ 150.6 (d, J = 253.2 Hz), 135.9, 128.9, 128.5, 124.9, 120.4 (d, J = 14.2 Hz), 120.3 (q, J = 325.9 Hz), 117.3, 106.5 (d, J = 44.9 Hz), 26.9 (d, J = 25.0 Hz). **$^{19}F\{^1H\}$ NMR** (377 MHz, benzene-d_6) δ -68.3 – -79.3 (m), -128.0. **HRMS** (EI) m/z calc. for $C_{12}H_9F_4NO_2S$ $[M]^+$ 307.02846, found 307.02850.

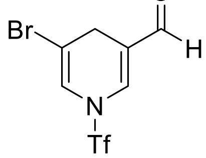

5-Bromo-1-((trifluoromethyl)sulfonyl)-1,4-dihydropyridine-3-carbaldehyde (**19**): The title compound was synthesized from 5-bromonicotinaldehyde (0.5 mmol, 93 mg, 1.0 equiv) using GP-A. The product was purified by column chromatography

(pentane/diethylether 90:10) to afford **19** as a yellow oil (107 mg, 67% yield, 93:7 ratio of 1,4-DHP vs. other DHPs).

R$_f$ (pentane/ethyl acetate 80:20) 0.42. **^{1}H NMR** (400 MHz, benzene-*d*$_6$) δ 8.43 (s, 1H), 6.49 – 6.37 (m, 2H), 2.70 (s, 2H). **^{13}C{^{1}H} NMR** (101 MHz, benzene-*d*$_6$) δ 187.6, 135.7, 122.0, 120.0, 119.6 (q, *J* = 324.4 Hz), 109.9, 29.1. **^{19}F{^{1}H} NMR** (377 MHz, benzene-*d*$_6$) δ -75.2. **GC** (EI) m/z calc. for $C_7H_5F_3BrNO_3S$ [M]$^+$ 318.91, found 318.93.

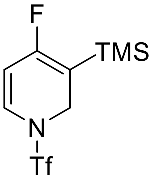

4-Fluoro-1-((trifluoromethyl)sulfonyl)-3-(trimethylsilyl)-1,2-dihydropyridine (**20**): The title compound was synthesized from 4-fluoro-3-(trimethylsilyl)pyridine (0.50 mmol, 85 mg, 1.0 equiv) using GP-A. The product was purified by column chromatography (pentane) to afford **20** as an orange oil (104 mg, 69% yield, 78:22 ratio of 1,4-DHP vs. other DHPs).

R$_f$ (pentane/ethyl acetate 80:20) 0.68. **^{1}H NMR** (400 MHz, benzene-*d*$_6$) δ 6.43 (d, *J* = 4.9 Hz, 1H), 4.37 – 4.22 (m, 1H), 3.87 – 3.72 (m, 2H), 0.02 (s, 9H).**^{13}C{^{1}H} NMR** (101 MHz, benzene-*d*$_6$) δ 158.7 (d, *J* = 246.0 Hz), 132.2 (d, *J* = 13.0 Hz), 120.4 (q, *J* = 324.2 Hz), 116.9 (d, *J* = 47.1 Hz), 93.9 (d, *J* = 24.8 Hz), 45.0 (d, *J* = 8.7 Hz), -1.7. **^{19}F{^{1}H} NMR** (377 MHz, benzene-*d*$_6$) δ -75.8, -111.9. **GC** (EI) m/z calc. for $C_9H_{13}NO_2F_4SSi$ [M]$^+$ 303.0, found 303.1.

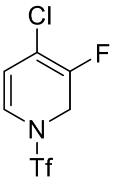

4-Chloro-3-fluoro-1-((trifluoromethyl)sulfonyl)-1,2-dihydropyridine (**21**): The title compound was synthesized from 4-chloro-3-fluoropyridine (0.50 mmol, 49 µL, 1.0 equiv) using GP-A. The product was purified by column chromatography (pentane) to afford **21** as a colorless oil (135 mg, 74% yield, 95:5 ratio of 1,4-DHP vs. other DHPs).

R$_f$ (pentane/ethyl acetate 80:20) 0.62. **^{1}H NMR** (400 MHz, benzene-*d*$_6$) δ 5.65 (d, J = 7.7 Hz, 1H), 4.82 – 4.52 (m, 1H), 3.76 – 3.48 (m, 2H).**^{13}C{^{1}H} NMR** (101 MHz, benzene-*d*$_6$) δ 145.3 (d, *J* = 274.7 Hz), 121.7 (d, *J* = 5.2 Hz), 120.1 (q, *J* = 325.4 Hz), 110.5, 105.6 (d, *J* = 15.8 Hz), 45.6 (d, *J* = 36.5 Hz). **^{19}F{^{1}H} NMR** (377 MHz, benzene-*d*$_6$) δ -75.6, -111.4. **GC** (EI) m/z calc. for $C_6H_4NO_2SF_4Cl$ [M]$^+$ 265.0, found 264.9.

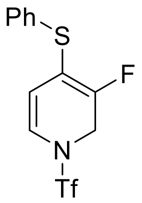

3-Fluoro-4-(phenylthio)-1-((trifluoromethyl)sulfonyl)-1,2-dihydropyridine (**22**): The title compound was synthesized from 3-fluoro-4-(phenylthio)pyridine (0.50 mmol, 103 mg, 1.0 equiv) using GP-A. The product was purified by column chromatography (pentane/diethylether 80:20) to afford **22** as a colorless oil (135 mg, 74% yield, 95:5 ratio of 1,4-DHP vs. other DHPs).

R$_f$ (pentane/diethylether 80:20) 0.77. **^{1}H NMR** (400 MHz, benzene-*d*$_6$) δ 7.15 – 7.09 (m, 2H), 7.00 – 6.86 (m, 3H), 5.78 (d, *J* = 7.5 Hz, 1H), 4.83 (t, *J* = 7.1 Hz, 1H), 3.89 – 3.79 (m, 2H). **^{13}C{^{1}H} NMR** (101 MHz, benzene-*d*$_6$) δ 149.9 (d, *J* = 274.9 Hz), 132.7, 130.9, 129.5, 127.6, 121.3 (d, *J* = 4.7 Hz), 120.3 (q, *J* = 324.7 Hz), 112.6, 106.5 (d, *J* = 14.8 Hz), 45.6 (d, *J* = 39.2 Hz). **^{19}F{^{1}H} NMR** (377 MHz, benzene-*d*$_6$) δ -75.6, -101.3. **HRMS** (EI) m/z calc. for $C_{12}H_9NO_2S_2F_4$ $[M]^+$ 339.0005, found 339.0008.

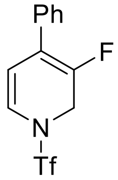

3-Fluoro-4-phenyl-1-((trifluoromethyl)sulfonyl)-1,2-dihydropyridine (**23**): The title compound was synthesized from 3-fluoro-4-phenylpyridine (0.30 mmol, 52 mg, 1.0 equiv) using GP-A. The product was purified by column chromatography (pentane) to afford **23** as a colorless oil (71 mg, 77% yield, 94:6 ratio of 1,4-DHP vs. other DHPs).

R$_f$ (pentane/ethyl acetate 80:20) 0.73. **^{1}H NMR** (400 MHz, benzene-*d*$_6$) δ 7.19 – 7.14 (m, 2H), 7.13 – 7.08 (m, 2H), 7.07 – 7.00 (m, 1H), 6.01 (d, *J* = 7.5 Hz, 1H), 5.21 – 5.10 (m, 1H), 3.97 – 3.86 (m, 2H). **^{13}C{^{1}H} NMR** (101 MHz, benzene-*d*$_6$) δ 146.4 (d, *J* = 276.4 Hz), 132.6 (d, *J* = 2.3 Hz), 128.8, 128.4, 128.0, 128.0, 120.9 (d, *J* = 4.6 Hz), 120.4 (q, *J* = 324.4 Hz), 112.6 (d, *J* = 2.5 Hz), 45.2 (d, *J* = 40.5 Hz). **^{19}F{^{1}H} NMR** (377 MHz, benzene-*d*$_6$) δ -75.5, -112.9. **HRMS** (EI) m/z calc. for $C_{12}H_9NO_2SF_4$ $[M]^+$ 307.02846, found 307.02848.

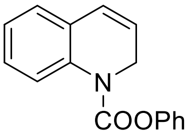

Phenyl quinoline-1(2H)-carboxylate (**24**): The title compound was synthesized from quinoline (0.50 mmol, 59 µL, 1.0 equiv) using GP-B. The product was purified by column chromatography (pentane/ethyl acetate 90:10) to afford **24** as a colorless oil (108 mg, 86% yield).

(One signal in ^{13}C{^{1}H} NMR is missing.)

R_f (pentane/ethyl acetate 98:2) 0.53. **^{1}H NMR** (400 MHz, chloroform-*d*$_3$) δ 7.78 – 7.67 (m, 1H), 7.46 – 7.35 (m, 2H), 7.29 – 7.23 (m, 2H), 7.23 – 7.19 (m, 2H), 7.17 – 7.12 (m, 2H), 6.59 (dt, *J* = 9.6, 1.9 Hz, 1H), 6.10 (dt, *J* = 9.6, 4.1 Hz, 1H), 4.58 (dd, *J* = 4.1, 1.9 Hz, 2H). **^{13}C{^{1}H} NMR** (101 MHz, chloroform-*d*$_3$) δ 152.8, 151.2, 136.0, 129.5, 128.3, 127.7, 126.6, 126.5, 125.7, 125.0, 123.8, 121.7, 43.9. **HRMS** (ESI) m/z calc. for $C_{16}H_{13}NO_2Na$ [M+Na]$^+$ 274.08495, found 274.08358.

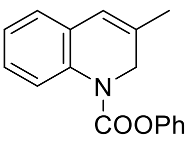

Phenyl 3-methylquinoline-1(2H)-carboxylate (**25**): The title compound was synthesized from 3-methylquinoline (2.0 mmol, 268 µL, 1.0 equiv) using GP-B. The product was purified by column chromatography (pentane/ethyl acetate 95:5 to 90:10) to afford **25** as a white solid (496 mg, 93% yield).

R_f (pentane/ethyl acetate 90:10) 0.47. **^{1}H NMR** (400 MHz, chloroform-*d*$_3$) δ 7.70 (s, 1H), 7.44 – 7.35 (m, 2H), 7.27 – 7.15 (m, 4H), 7.12 – 7.05 (m, 2H), 6.33 – 6.27 (m, 1H), 4.42 (s, 2H), 1.96 (s, 3H). **^{13}C{^{1}H} NMR** (101 MHz, chloroform-*d*$_3$) δ 152.9, 151.2, 134.6, 129.5, 129.0, 126.7, 125.8, 125.7, 125.0, 123.5, 121.8, 121.6, 115.4, 48.2, 20.8. **HRMS** (ESI) m/z calc. for $C_{17}H_{15}NO_2Na$ [M+Na]$^+$ 288.09950, found 288.09924.

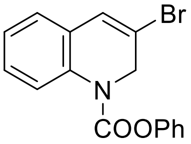

Phenyl 3-bromoquinoline-1(2H)-carboxylate (**26**): The title compound was synthesized from 3-bromoquinoline (0.5 mmol, 68 µL, 1.0 equiv) using GP-B. The product was purified by column chromatography (pentane/ethyl acetate 99:1 to 98:2) to afford **26** as a colorless oil (152 mg, 92% yield).

R_f (pentane/ethyl acetate 95:5) 0.29. **^{1}H NMR** (400 MHz, chloroform-*d*$_3$) δ 7.72 – 7.66 (m, 1H), 7.45 – 7.35 (m, 2H), 7.31 – 7.21 (m, 2H), 7.20 – 7.17 (m, 2H), 7.17 – 7.12 (m, 1H), 7.11 – 7.07 (m, 1H), 6.89 (s, 1H), 4.75 (s, 2H). **^{13}C{^{1}H} NMR** (101 MHz, chloroform-*d*$_3$) δ 152.5, 151.0, 134.3, 129.6, 128.1, 126.1, 126.0, 125.5, 124.1, 121.7, 50.8. **HRMS** (ESI) m/z calc. for $C_{16}H_{12}NO_2BrNa$ [M+Na]$^+$ 351.99436, found 351.99439.

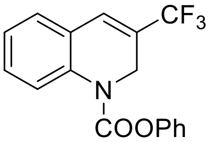

Phenyl 3-(trifluoromethyl)quinoline-1(2H)-carboxylate (**27**): The title compound was synthesized from 3-(trifluoromethyl) quinoline (0.50 mmol, 99 mg, 1.0 equiv) using GP-B. The reaction was stirred for additional two hours at 50 °C. The product was purified by column chromatography (pentane/ethyl acetate 98:2) to afford **27** as a colorless oil (108 mg, 86% yield). Both isomers (70:30 ratio of 1,2-dihydroquinoline vs. other dihydroquinoline) could be isolated. NMR data is given for major isomer.

(One signal in ^{13}C{^{1}H} NMR is missing.)

R$_f$ (pentane/ethyl acetate 98:2) 0.14. **^{1}H NMR** (400 MHz, benzene-*d*$_6$) δ 8.01 – 7.81 (m, 1H), 7.34 – 7.30 (m, 1H), 7.20 – 7.13 (m, 4H), 7.08 – 7.02 (m, 1H), 6.97 – 6.90 (m, 1H), 6.83 – 6.78 (m, 1H), 6.77 – 6.72 (m, 1H), 4.62 – 4.56 (m, 2H). **^{13}C{^{1}H} NMR** (101 MHz, benzene-*d*$_6$) δ 151.9, 151.5, 137.1, 130.1, 129.5, 128.5, 125.8, 125.6, 125.0, 124.6, 124.3, 121.9, 119.2, 41.4. **^{19}F{^{1}H} NMR** (377 MHz, benzene-*d*$_6$) δ -68.1. **HRMS** (ESI) m/z calc. for $C_{17}H_{12}NO_2F_3Na$ [M+Na]$^+$ 342.07123, found 342.07100.

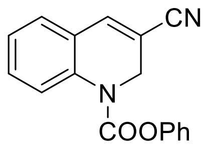

Phenyl 3-cyanoquinoline-1(2H)-carboxylate (**28**): The title compound was synthesized from 3-cyanoquinoline (0.50 mmol, 77 mg, 1.0 equiv) using GP-B. The reaction was stirred for additional two hours at 50 °C. The product was purified by column chromatography (pentane/ethyl acetate 80:20) to afford **28** as a colorless oil (109 mg, 79% yield). Both isomers (78:22 ratio of 1,2-dihydroquinoline vs. other dihydroquinoline) could be isolated. NMR data is given for major isomer.

(One signal in ^{13}C{^{1}H} NMR is missing.)

R$_f$ (pentane/ethyl acetate 98:2) 0.20. **^{1}H NMR** (400 MHz, benzene-*d*$_6$) δ 7.65 (m, 1H), 7.06 – 6.86 (m, 6H), 6.76 – 6.66 (m, 1H), 6.55 – 6.49 (m, 1H), 6.31 – 6.26 (m, 1H), 4.14 – 4.08 (m, 2H). **^{13}C{^{1}H} NMR** (101 MHz, benzene-*d*$_6$) δ 151.7, 151.3, 139.4, 137.1, 130.7, 129.5, 128.2, 125.9, 125.7, 125.0, 124.3, 121.9, 116.9, 43.8. **HRMS** (ESI) m/z calc. for $C_{17}H_{12}N_2O_2Na$ [M+Na]$^+$ 299.07910, found 299.07898.

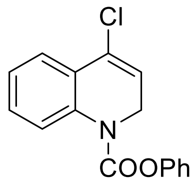

Phenyl 4-chloroquinoline-1(2H)-carboxylate (**29**): The title compound was synthesized from 4-chloroquinoline (0.50 mmol, 82 mg, 1.0 equiv) using GP-B. The reaction was stirred for additional two hours at 50 °C. The product was purified by column chromatography (pentane/ethyl acetate 80:20) to afford **29** as a colorless oil (124 mg, 87% yield).

R$_f$ (pentane/ethyl acetate 95:5) 0.25. **^{1}H NMR** (400 MHz, chloroform-*d*$_3$) δ 7.64 – 7.55 (m, 1H), 7.55 – 7.46 (m, 1H), 7.30 – 7.24 (m, 2H), 7.23 – 7.18 (m, 1H), 7.15 – 7.03 (m, 4H), 6.08 (t, *J* = 4.6 Hz, 1H), 4.46 (d, *J* = 4.6 Hz, 2H). **^{13}C{^{1}H} NMR** (101 MHz, chloroform-*d*$_3$) δ 152.4, 151.0, 136.4, 129.5, 129.0, 126.6, 125.8, 125.2, 124.7, 123.9, 121.6, 44.4. **HRMS** (ESI) m/z calc. for $C_{16}H_{12}NO_2ClNa$ [M+Na]$^+$ 308.04488, found 308.04474.

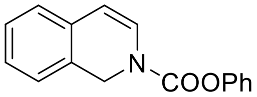

Phenyl isoquinoline-2(1H)-carboxylate (**30**): The title compound was synthesized from isoquinoline (0.50 mmol, 59 µL, 1.0 equiv) using GP-B. The product was purified

by column chromatography (pentane/ethyl acetate 90:10) to afford **30** as a white solid (118 mg, 94% yield).

(Two rotamers are observed due to the dynamic phenyl carboxylate group.)

R$_f$ (pentane/ethyl acetate 90:10) 0.58. **^{1}H NMR** (400 MHz, chloroform-*d*$_3$) δ 7.44 – 7.36 (m, 2H), 7.29 – 7.14 (m, 5H), 7.13 – 7.00 (m, 3H), 5.96 – 5.85 (m, 1H), 5.13 – 4.90 (m, 2H). **^{13}C{^{1}H} NMR** (101 MHz, chloroform-*d*$_3$) δ 151.4, 151.0, 130.9, 129.6, 128.8, 128.1, 127.4, 126.4, 125.9, 125.8, 125.0, 121.7, 109.6, 46.6. **HRMS** (ESI) m/z calc. for $C_{16}H_{13}NO_2Na$ [M+Na]$^+$ 274.08495, found 274.08372.

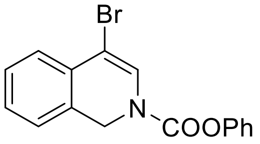

Phenyl 4-bromoisoquinoline-2(1H)-carboxylate (**31**): The title compound was synthesized from 4-bromoisoquinoline (0.50 mmol, 104 mg, 1.0 equiv) using GP-B. The product was purified by column chromatography (pentane to pentane/ethyl acetate 95:5) to afford **31** as a colorless oil (165 mg, 99% yield).

R$_f$ (pentane/ethyl acetate 95:5) 0.20. **^{1}H NMR** (599 MHz, toluene-*d*$_8$, *T* = 363 K) δ 7.42 – 7.36 (m, 1H), 7.36 – 7.30 (m, 1H), 7.10 – 7.03 (m, 2H), 7.03 – 6.95 (m, 3H), 6.94 – 6.85 (m, 2H), 6.63 – 6.55 (m, 1H), 4.64 (s, 2H). **^{13}C{^{1}H} NMR** (151 MHz, toluene-*d*$_8$, *T* = 363 K) δ 151.8, 150.4, 130.9, 129.5, 129.5, 128.6, 128.4, 127.6, 125.8, 125.7, 125.4, 121.7, 104.8, 46.8. **HRMS** (ESI) m/z calc. for $C_{16}H_{12}NO_2BrNa$ [M+Na]$^+$ 351.99436, found 351.99436.

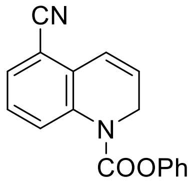

Phenyl 5-cyanoquinoline-1(2H)-carboxylate (**32**): The title compound was synthesized from quinoline-5-carbonitrile (0.50 mmol, 77 mg, 1.0 equiv) using GP-B. The product was purified by column chromatography (pentane to pentane/ethyl acetate 95:5) to afford **32** as a white solid (113 mg, 82% yield). Traces of amine borane NMe_3BH_3 could not be removed and the yield was corrected for this.

(Two rotamers are observed due to the dynamic phenyl carboxylate group.)

R$_f$ (pentane/ethyl acetate 95:5) 0.38. **^{1}H NMR** (400 MHz, chloroform-*d*$_3$) δ 7.93 – 7.83 (m, 1H), 7.47 – 7.33 (m, 3H), 7.32 – 7.21 (m, 3H), 7.20 – 7.12 (m, 2H), 6.71 – 6.60 (m, 1H), 5.16 – 4.95 (m, 2H). **HRMS** (ESI) m/z calc. for $C_{17}H_{12}N_2O_2Na$ [M+Na]$^+$ 299.07910, found 299.07889.

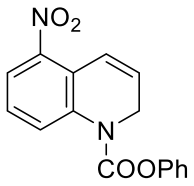

Phenyl 5-nitroquinoline-1(2H)-carboxylate (**33**): The title compound was synthesized from 5-nitroquinoline (0.50 mmol, 87 mg, 1.0 equiv) using GP-B. The product was purified by column chromatography (pentane/ethyl acetate 90:10 to 80:20) to afford **33** as a yellow solid (117 mg, 79% yield).

(Two rotamers are observed due to the dynamic phenyl carboxylate group.)

R$_f$ (pentane/ethyl acetate 90:10) 0.45. **^{1}H NMR** (400 MHz, chloroform-*d*$_3$) δ 7.92 – 7.86 (m, 1H), 7.45 – 7.38 (m, 2H), 7.38 – 7.33 (m, 1H), 7.32 – 7.22 (m, 3H), 7.21 – 7.13 (m, 2H), 6.72 – 6.53 (m, 1H), 5.14 – 4.94 (m, 2H). **^{13}C{^{1}H} NMR** (101 MHz, chloroform-*d*$_3$) δ 151.5, 150.9, 144.8, 131.4, 131.2, 130.7, 130.4, 129.7, 127.1, 126.2, 124.5, 121.6, 103.8, 46.4. **HRMS** (ESI) m/z calc. for $C_{16}H_{12}N_2O_4Na$ [M+Na]$^+$ 319.06893, found 319.06893.

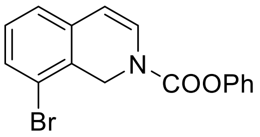

Phenyl 8-bromoisoquinoline-2(1H)-carboxylate (**34**): The title compound was synthesized from 8-bromoisoquinoline (0.50 mmol, 104 mg, 1.0 equiv) using GP-B. The product was purified by column chromatography (neutral alumina, activity grade III, pentane/ethyl acetate 95:5) to afford **34** as a white solid (145 mg, 88% yield).

(Two rotamers are observed due to the dynamic phenyl carboxylate group.)

R$_f$ (pentane/ethyl acetate 95:5) 0.40. **^{1}H NMR** (400 MHz, benzene-*d*$_6$) δ 7.10 – 6.73 (m, 7H), 6.61 – 6.55 (m, 1H), 6.54 – 6.45 (m, 1H), 5.35 – 5.27 (m, 1H), 4.94 – 4.83 (m, 2H). **^{13}C{^{1}H} NMR** (101 MHz, benzene-*d*$_6$) δ 151.6, 151.1, 133.5, 130.9, 129.5, 129.1, 128.7, 126.9, 125.8, 124.0, 122.2, 121.9, 107.8, 47.0. **HRMS** (ESI) m/z calc. for $C_{16}H_{12}NO_2BrNa$ [M+Na]$^+$ 351.99436, found 351.99432.

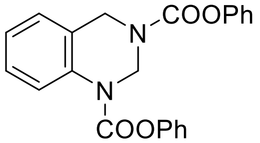

Diphenyl quinazoline-1,3(2H,4H)-dicarboxylate (**35**): The title compound was synthesized from quinazoline (0.50 mmol, 65 mg, 1.0 equiv) and phenyl chloroformate (2.2 equiv) using GP-B. The product was purified by column chromatography (pentane/ethyl acetate 90:10 to 80:20) to afford **35** as a colorless oil (181 mg, 97% yield).

(Two rotamers are observed due to the dynamic phenyl carboxylate group.)

R$_f$ (pentane/ethyl acetate 80:20) 0.24. **^{1}H NMR** (400 MHz, benzene-*d*$_6$) δ 8.15 – 7.78 (m, 1H), 7.28 – 6.96 (m, 9H), 6.94 – 6.80 (m, 3H), 6.69 – 6.61 (m, 1H), 5.32 – 5.08 (m, 2H), 4.65 – 4.24 (m, 2H). **^{13}C{^{1}H} NMR** (101 MHz, benzene-*d*$_6$) δ 153.0, 152.4, 152.0,

151.8, 151.6, 137.4, 129.5, 129.5, 127.2, 126.7, 125.8, 125.6, 125.1, 124.5, 122.1, 122.0, 56.5, 45.8. **HRMS** (ESI) m/z calc. for $C_{22}H_{18}N_2O_4Na$ $[M+Na]^+$ 397.11588, found 397.11554.

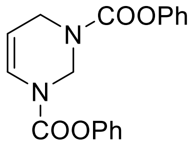

Diphenyl pyrimidine-1,3(2H,4H)-dicarboxylate (**36**): The title compound was synthesized from pyrimidine (0.50 mmol, 39 µL, 1.0 equiv) and phenyl chloroformate (2.2 equiv) using GP-B. The product was purified by column chromatography (pentane/ethyl acetate 70:30) to afford **36** as a colorless oil (148 mg, 91% yield).

(Two rotamers are observed due to the dynamic phenyl carboxylate group.)

R_f (pentane/ethyl acetate 70:30) 0.43. **^{1}H NMR** (400 MHz, benzene-d_6) δ 7.14 – 6.96 (m, 8H), 6.94 – 6.70 (m, 3H), 5.15 – 4.97 (m, 2H), 4.58 – 4.37 (m, 1H), 3.84 – 3.64 (m, 2H). **^{13}C{^{1}H} NMR** (101 MHz, benzene-d_6) δ 151.8, 151.4, 151.3, 150.4, 129.5, 125.8, 125.7, 125.5, 122.0, 122.0, 106.7, 105.9, 55.3, 42.4. **HRMS** (ESI) m/z calc. for $C_{18}H_{16}N_2O_4Na$ $[M+Na]^+$ 347.10023, found 347.09985.

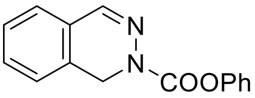

Phenyl phthalazine-2(1H)-carboxylate (**37**): The title compound was synthesized from phthalazine (0.50 mmol, 65 mg, 1.0 equiv) using GP-B. The product was purified by column chromatography (pentane/ethyl acetate 90:10 to 80:20) to afford **37** as a white solid (79 mg, 62% yield).

R_f (pentane/ethyl acetate 80:20) 0.37. **^{1}H NMR** (400 MHz, benzene-d_6) δ 7.31 – 7.25 (m, 3H), 7.09 – 7.02 (m, 2H), 6.93 – 6.79 (m, 3H), 6.58 – 6.54 (m, 1H), 6.44 – 6.40 (m, 1H), 4.68 (s, 2H). **^{13}C{^{1}H} NMR** (101 MHz, benzene-d_6) δ 153.3, 152.3, 142.3, 131.3, 130.3, 129.8, 129.5, 125.9, 125.8, 125.6, 124.6, 122.2, 44.1. **HRMS** (ESI) m/z calc. for $C_{15}H_{12}N_2O_2Na$ $[M+Na]^+$ 275.07910, found 275.07885.

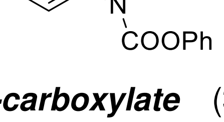

Phenyl benzo[d]thiazole-3(2H)-carboxylate (**38**): The title compound was synthesized from benzothiazole (0.50 mmol, 55 µL, 1.0 equiv) using GP-B. The product was purified by column chromatography (pentane/ethyl acetate 98:2 to 95:5) to afford **38** as a white solid (130 mg, 99% yield).

(One signal in ^{13}C{^{1}H} NMR is missing.)

R_f (pentane/ethyl acetate 90:10) 0.67. **^{1}H NMR** (400 MHz, chloroform-d_3) δ 7.93 – 7.81 (m, 1H), 7.45 – 7.37 (m, 2H), 7.29 – 7.23 (m, 1H), 7.22 – 7.14 (m, 3H), 7.12 – 7.05 (m, 1H), 7.03 – 6.97 (m, 1H), 5.40 (s, 2H). **^{13}C{^{1}H} NMR** (101 MHz, chloroform-d_3) δ 154.1, 150.6, 138.4, 129.7, 126.2, 125.6, 124.6, 122.4, 121.8, 117.1, 51.2. **HRMS** (ESI) m/z calc. for $C_{14}H_{11}NO_2SNa$ $[M+Na]^+$ 280.04027, found 280.04018.

2.5. Stepwise Hydrogenation

General procedure C (GP-C)

In a 4 mL screw-cap vial, equipped with magnetic stir bar, N-heterocycle (0.5 mmol, 1.0 equiv) was dissolved in MeCN (1.0 mL) and PhOCOCl (75 µL, 0.60 mmol, 1.2 equiv) was added. After stirring for 5 minutes at 25 °C, NMe_3BH_3 (40 mg, 0.55 mmol, 1.1 equiv) was added and stirring for additional 5 minutes followed. $Pd_{2.5\%}/Pt_{2.5\%}/C$ (98 mg, 5 mol%) and NMe_3BH_3 (40 mg, 0.55 mmol, 1.1 equiv) were added and the vial was tightly closed. The mixture was stirred for 16 h at room temperature. Afterwards, the mixture was filtered over celite and the product was purified by column chromatography on silica gel.

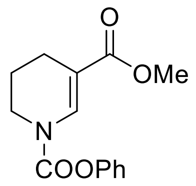

3-Methyl 1-phenyl 5,6-dihydropyridine-1,3(4H)-dicarboxylate (**40**): The title compound was synthesized according to GP-C from methyl nicotinate (69 mg, 0.50 mmol, 1.0 equiv). After column chromatography (pentane/ethyl acetate 85:15) the product was obtained as a white solid (96 mg, 73% yield).

(Two rotamers are observed due to the dynamic phenyl carboxylate group.)

R_f (pentane/ethyl acetate 80:20) 0.43. **^{1}H NMR** (400 MHz, chloroform-*d*$_3$) δ 8.28 – 7.99 (m, 1H), 7.44 – 7.36 (m, 2H), 7.30 – 7.23 (m, 1H), 7.19 – 7.11 (m, 2H), 3.76 (d, *J* = 40.5 Hz, 2H), 3.76 (s, 3H), 2.38 (td, *J* = 6.3, 1.6 Hz, 2H), 1.93 (p, *J* = 6.1 Hz, 2H). **^{13}C{^{1}H} NMR** (101 MHz, chloroform-*d*$_3$) δ 167.9, 151.6, 150.9, 135.1, 129.6, 126.2, 121.7, 109.7, 51.6, 42.7, 29.8, 20.9. **HRMS** (ESI) m/z calc. for $C_{14}H_{15}NO_4Na$ $[M+Na]^+$ 284.0893, found 284.0892.

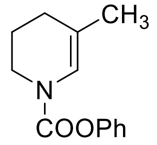

Phenyl 5-methyl-3,4-dihydropyridine-1(2H)-carboxylate (**41**): The title compound was synthesized according to GP-C from 3-methylpyridine (49 µL, 0.50 mmol, 1.0 equiv). After column chromatography (pentane to pentane/ethyl acetate 95:5) the product was obtained as a white solid (79 mg, 72% yield).

(Two rotamers are observed due to the dynamic phenyl carboxylate group.)

R_f (pentane/ethyl acetate 90:10) 0.55. **^{1}H NMR** (400 MHz, chloroform-*d*$_3$) δ 7.40 – 7.33 (m, 2H), 7.24 – 7.17 (m, 1H), 7.15 – 7.10 (m, 2H), 6.81 – 6.64 (m, 1H), 3.75 – 3.69 (m, 1H), 3.68 – 3.61 (m, 1H), 2.11 – 1.98 (m, 2H), 1.98 – 1.84 (m, 2H), 1.72 (s, 3H). **^{13}C{^{1}H} NMR** (101 MHz, chloroform-*d*$_3$) δ 152.1, 151.7, 151.4, 151.3, 129.4, 125.5, 125.5, 121.9, 121.8, 120.1, 119.8, 117.0, 116.5, 42.5, 42.0, 27.1, 26.9, 22.0, 21.8, 21.1, 21.1. **HRMS** (ESI) m/z calc. for $C_{13}H_{15}NO_2Na$ $[M+Na]^+$ 240.0995, found 240.0990.

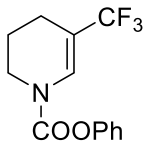

Phenyl 5-(trifluoromethyl)-3,4-dihydropyridine-1(2H)-carboxylate (**S2**): The title compound was synthesized according to GP-C from 3-(trifluormethyl)pyridine (58 µL, 0.50 mmol, 1.0 equiv). After column chromatography (pentane/ethyl acetate, 85:15) the product was obtained as a colorless oil (75 mg, 55% yield).

(Two rotamers are observed due to the dynamic phenyl carboxylate group. Not all signals could be assigned in $^{13}C\{^1H\}$ NMR due to coupling to fluor.)

R_f (pentane/ethyl acetate 80:20) 0.63. **^{1}H NMR** (400 MHz, benzene-*d*6) δ 7.57 – 7.48 (m, 1H), 7.37 – 7.28 (m, 2H), 7.22 – 7.13 (m, 1H), 7.11 – 7.03 (m, 2H), 3.81 – 3.55 (m, 2H), 2.25 – 2.14 (m, 2H), 1.98 – 1.86 (m, 2H). **^{13}C{^{1}H} NMR** (101 MHz, benzene-*d*6) δ 150.8, 129.7, 126.2, 121.7, 121.6, 42.3, 20.4, 19.6. **HRMS** (ESI) m/z calc. for $C_{13}H_{12}NO_2F_3Na$ $[M+Na]^+$ 294.0712, found 294.0710.

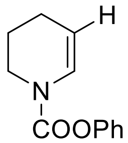

Phenyl 3,4-dihydropyridine-1(2H)-carboxylate (**42**): The title compound was synthesized according to GP-C from pyridine (20 µL, 0.25 mmol, 1.0 equiv). After column chromatography (pentane to pentane/ethyl acetate 95:5) the product was obtained as a white solid (49 mg, 94% yield).

(Two rotamers are observed due to the dynamic phenyl carboxylate group.)

R_f (pentane/ethyl acetate 90:10) 0.52. **^{1}H NMR** (400 MHz, chloroform-*d*3) δ 7.46 – 7.33 (m, 3H), 7.32 – 7.27 (m, 1H), 7.25 – 7.18 (m, 1H), 7.16 – 7.12 (m, 2H), 7.00 – 6.88 (m, 1H), 5.19 – 4.88 (m, 1H), 3.99 – 3.60 (m, 2H), 2.16 – 2.07 (m, 2H), 1.97 – 1.86 (m, 2H). **^{13}C{^{1}H} NMR** (101 MHz, chloroform-*d*3) δ 152.1, 151.2, 129.6, 126.0, 125.2, 121.8, 121.0, 107.9, 42.9, 21.8, 21.5. **HRMS** (ESI) m/z calc. for $C_{12}H_{13}NO_2Na$ $[M+Na]^+$ 226.08385, found 226.08347.

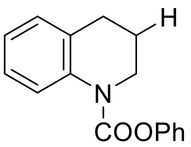

Phenyl 3,4-dihydroquinoline-1(2H)-carboxylate (**43**): The title compound was synthesized according to GP-C from quinoline (29 µL, 0.25 mmol, 1.0 equiv). After column chromatography (pentane to pentane/ethyl acetate 95:5) the product was obtained as a yellow oil (60 mg, 95% yield).

(Two rotamers are observed due to the dynamic phenyl carboxylate group.)

R_f (pentane/ethyl acetate 90:10) 0.47. **^{1}H NMR** (400 MHz, chloroform-*d*3) δ 7.84 – 7.75 (m, 1H), 7.45 – 7.35 (m, 2H), 7.28 – 7.12 (m, 5H), 7.09 – 7.02 (m, 1H), 3.94 (t, *J* = 6.1 Hz, 2H), 2.86 (t, *J* = 6.6 Hz, 2H), 2.05 (p, *J* = 6.5 Hz, 2H). **^{13}C{^{1}H} NMR** (101 MHz,

chloroform-d_3) δ 153.4, 151.3, 137.9, 129.5, 128.8, 126.3, 125.6, 124.3, 124.1, 121.9, 115.4, 45.5, 27.4, 23.6. **HRMS** (ESI) m/z calc. for $C_{16}H_{15}NO_2Na$ $[M+Na]^+$ 276.09950, found 276.09912.

2.6. Fluorination

General procedure D (GP-D)

In a 4 mL screw-cap vial, equipped with magnetic stir bar, the dihydro N-heterocycle (1.0 equiv) was dissolved in MeCN (1.0 mL). Selectfluor (1.0 equiv) and NMe_3BH_3 (1.0 equiv) were added and the mixture was stirred for 16 h at room temperature. Afterwards, the raw product was purified by column chromatography on silica gel.

General procedure E (GP-E)

In a 4 mL screw-cap vial, equipped with magnetic stir bar, the dihydro N-heterocycle (1.0 equiv) was dissolved in MeCN (1.0 mL). Selectfluor (2.0 equiv) was added and the mixture was stirred for 16 h at room temperature. Afterwards, the raw product was purified by column chromatography on silica gel.

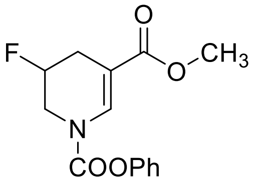

3-Methyl 1-phenyl 5-fluoro-5,6-dihydropyridine-1,3(4H)-dicarboxylate (**44**): The title compound was synthesized according to GP-D from dihydropyridine **S1** (39 mg, 0.15 mmol, 1.0 equiv). After column chromatography (pentane/ethyl acetate 80:20) the product was obtained as a white solid (35 mg, 84% yield) with traces of two other isomers.

(Two rotamers are observed due to the dynamic phenyl carboxylate group.)

R_f (pentane/ethyl acetate 80:20) 0.24. **^{1}H NMR** (400 MHz, chloroform-d_3) δ 8.30 – 8.17 (m, 1H), 7.44 – 7.38 (m, 2H), 7.30 – 7.25 (m, 1H), 7.19 – 7.14 (m, 2H), 5.34 – 5.08 (m, 1H), 4.48 – 4.27 (m, 1H), 3.78 (s, 3H), 3.70 – 3.35 (m, 1H), 2.92 – 2.79 (m, 1H), 2.64 – 2.46 (m, 1H). **^{13}C{^{1}H} NMR** (101 MHz, chloroform-d_3) δ 167.3, 150.7, 134.7, 129.5, 126.4, 121.6, 82.8 (d, J = 175.1 Hz), 51.9, 45.7 (d, J = 22.7 Hz), 27.2. **^{19}F{^{1}H} NMR** (376 MHz, chloroform-d_3) δ -182.6. **HRMS** (ESI) m/z calc. for $C_{14}H_{14}NO_4FNa$ $[M+Na]^+$ 302.07991, found 302.07997.

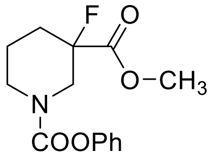

3-Methyl 1-phenyl 3-fluoropiperidine-1,3-dicarboxylate (**45**): The title compound was synthesized according to GP-D from tetrahydropyridine **40** (52 mg, 0.20 mmol,

1.0 equiv). After column chromatography (pentane/ethyl acetate 80:20) the product was obtained as a white solid (48 mg, 85% yield) with traces of another isomer.

(Two rotamers are observed due to the dynamic phenyl carboxylate group.)

R$_f$ (pentane/ethyl acetate 80:20) 0.22. **^{1}H NMR** (400 MHz, chloroform-*d$_3$*) δ 7.40 – 7.29 (m, 2H), 7.23 – 7.15 (m, 1H), 7.15 – 7.03 (m, 3H), 4.54 – 4.35 (m, 1H), 4.33 – 4.14 (m, 1H), 3.83 (s, 3H), 3.60 – 3.31 (m, 1H), 3.21 – 2.89 (m, 1H), 2.25 – 2.13 (m, 1H), 2.04 – 1.86 (m, 1H), 1.81 – 1.66 (m, 1H). **^{13}C{^{1}H} NMR** (101 MHz, chloroform-*d$_3$*) δ 170.5 (d, *J* = 23.1 Hz), 154.1, 151.3, 129.4, 125.5, 121.8, 91.0 (d, *J* = 191.5 Hz), 53.0, 49.3 (d, *J* = 23.6 Hz), 44.0, 31.5 (d, *J* = 23.4 Hz), 20.1. **^{19}F{^{1}H} NMR** (376 MHz, chloroform-*d$_3$*) δ -168.5. **HRMS** (ESI) m/z calc. for $C_{14}H_{16}NO_4FNa$ [M+Na]$^+$ 304.09556, found 304.09513.

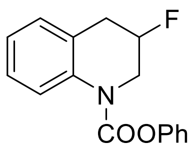

Phenyl 3-fluoro-3,4-dihydroquinoline-1(2H)-carboxylate (**46**): The title compound was synthesized according to GP-D from phenyl quinoline-1(2*H*)-carboxylate **24** (50 mg, 0.20 mmol, 1.0 equiv). After column chromatography (pentane/ethyl acetate 80:20) the product was obtained as a white solid (52 mg, 96% yield).

(Two rotamers are observed due to the dynamic phenyl carboxylate group.)

R$_f$ (pentane/ethyl acetate 80:20) 0.42. **^{1}H NMR** (500 MHz, toluene-*d$_8$*) δ 7.91 – 7.78 (m, 1H), 7.09 – 7.00 (m, 5H), 6.92 – 6.88 (m, 1H), 6.87 – 6.82 (m, 1H), 6.76 – 6.71 (m, 1H), 4.55 – 4.39 (m, 1H), 4.13 – 4.03 (m, 1H), 3.35 – 3.23 (m, 1H), 2.69 – 2.58 (m, 1H), 2.47 – 2.34 (m, 1H). **^{13}C{^{1}H} NMR** (126 MHz, toluene-*d$_8$*) δ 151.9, 138.0, 129.3, 128.2, 126.8, 125.4, 124.7, 122.0, 48.6, 33.3. **^{19}F{^{1}H} NMR** (470 MHz, toluene-*d$_8$*) δ -77.8. **HRMS** (ESI) m/z calc. for $C_{16}H_{14}NO_2FNa$ [M+Na]$^+$ 294.09063, found 294.08988.

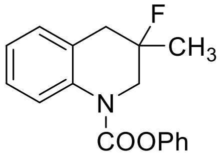

Phenyl 3-fluoro-3-methyl-3,4-dihydroquinoline-1(2H)-carboxylate (**47**): The title compound was synthesized according to GP-D from phenyl 3-methylquinoline-1(2*H*)-carboxylate **25** (80 mg, 0.30 mmol, 1.0 equiv). After column chromatography (pentane/ethyl acetate 95:5 to 90:10) the product was obtained as a white solid (62 mg, 72% yield).

(Two rotamers are observed due to the dynamic phenyl carboxylate group.)

R$_f$ (pentane/ethyl acetate 80:20) 0.49. **^{1}H NMR** (500 MHz, toluene-*d$_8$*) δ 7.95 – 7.82 (m, 1H), 7.11 – 7.00 (m, 5H), 6.93 – 6.84 (m, 2H), 6.81 – 6.74 (m, 1H), 4.30 – 4.20 (m, 1H), 3.12 – 3.00 (m, 1H), 2.78 – 2.66 (m, 1H), 2.41 – 2.26 (m, 1H), 1.12 – 1.04 (m, 3H). **^{13}C{^{1}H} NMR** (126 MHz, toluene-*d$_8$*) δ 153.2, 151.9, 137.6, 129.3, 126.8, 126.6, 125.4, 124.6, 124.6, 122.1, 53.4, 52.9, 39.1, 24.2. **^{19}F{^{1}H} NMR** (470 MHz, toluene-*d$_8$*)

δ -75.7. **HRMS** (ESI) m/z calc. for $C_{17}H_{16}NO_2FNa$ $[M+Na]^+$ 308.10573, found 308.10529.

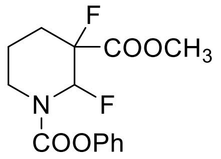

3-Methyl 1-phenyl 2,3-difluoropiperidine-1,3-dicarboxylate (**48**): The title compound was synthesized according to GP-E from tetrahydropyridne **40** (52 mg, 0.20 mmol, 1.0 equiv). After column chromatography (pentane/ethyl acetate 95:5 to 90:10) the product was obtained as a white solid (48 mg, 81% yield).

(Two rotamers of two conformers can be distinguished by ^{19}F NMR. The major signals are reported.)

R$_f$ (pentane/ethyl acetate 80:20) 0.35. **^{1}H NMR** (400 MHz, chloroform-*d*$_3$) δ 7.42 – 7.35 (m, 2H), 7.27 – 7.22 (m, 1H), 7.17 – 7.11 (m, 2H), 6.55 (dm, *J* = 47.7 Hz, 1H), 4.26 – 4.08 (m, 1H), 3.87 (s, 3H), 3.39 – 3.04 (m, 1H), 2.41 – 2.28 (m, 1H), 2.28 – 2.19 (m, 1H), 2.00 – 1.88 (m, 1H), 1.87 – 1.71 (m, 1H). **^{13}C{^{1}H} NMR** (101 MHz, chloroform-*d*$_3$) δ 167.9 (d, *J* = 22.6 Hz), 153.6, 150.8, 129.6, 126.1, 121.7, 92.4 – 88.0 (m), 53.3, 39.2 (d, *J* = 72.8 Hz), 25.2 (d, *J* = 20.8 Hz), 18.9. **^{19}F{^{1}H} NMR** (376 MHz, chloroform-*d*$_3$) -154.3, -165.1. **HRMS** (ESI) m/z calc. for $C_{14}H_{15}NO_4F_2Na$ $[M+Na]^+$ 322.08614, found 322.08590.

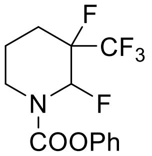

Phenyl 2,3-difluoro-3-(trifluoromethyl)piperidine-1-carboxylate (**49**): The title compound was synthesized according to GP-E from tetrahydropyridne **S2** (81 mg, 0.3 mmol, 1.0 equiv). After column chromatography (pentane/ethyl acetate 95:5 to 90:10) the product was obtained as a yellow oil (81 mg, 87% yield).

(Two rotamers of two conformers can be distinguished by ^{19}F NMR. The major signals are reported.)

R$_f$ (pentane/ethyl acetate 80:20) 0.38. **^{1}H NMR** (500 MHz, chloroform-*d*$_3$) δ 7.45 – 7.38 (m, 2H), 7.31 – 7.24 (m, 1H), 7.20 – 7.12 (m, 2H), 6.61 – 6.46 (m, 1H), 4.30 – 4.13 (m, 1H), 3.29 (d, J = 66.7 Hz, 1H), 2.32 – 1.81 (m, 4H). **^{13}C{^{1}H, ^{19}F} NMR** (126 MHz, chloroform-*d*$_3$) δ 153.2, 150.7, 129.7, 126.3, 122.6, 121.6, 88.8, 88.5, 39.0, 22.7, 18.4.**^{19}F{^{1}H} NMR** (470 MHz, chloroform-*d*$_3$) -77.0 – -85.6 (m), -157.1, -176.7. **HRMS** (ESI) m/z calc. for $C_{13}H_{12}NO_2F_5Na$ $[M+Na]^+$ 332.06804, found 332.06804.

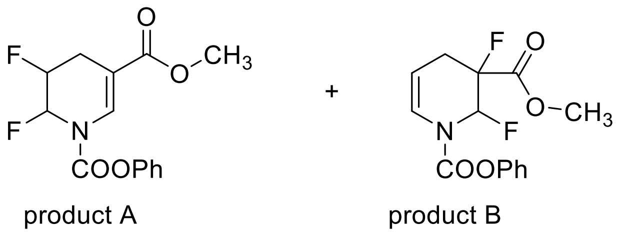

The title compounds were synthesized according to GP-E from dihydropyridine **S1** (39 mg, 0.15 mmol, 1.0 equiv). After preparative thin layer chromatography (pentane/ethyl acetate 80:20) product A (23 mg, 52% yield) and product B (15 mg, 34% yield) were obtained as white solids.

(A)*3-Methyl 1-phenyl 5,6-difluoro-5,6-dihydropyridine-1,3(4H)-dicarboxylate* (**51**):

(Two rotamers are observed due to the dynamic phenyl carboxylate group. Two rotamers of two conformers can be distinguished by ^{19}F NMR. The major signals are reported.)

R_f (pentane/ethyl acetate 80:20) 0.34. **^{1}H NMR** (400 MHz, chloroform-*d_3*) δ 8.27 – 8.07 (m, 1H), 7.49 – 7.38 (m, 2H), 7.34 – 7.28 (m, 1H), 7.24 – 7.16 (m, 2H), 6.69 – 6.38 (m, 1H), 5.28 – 5.05 (m, 1H), 3.87 (s, 3H), 3.07 – 2.77 (m, 1H), 2.74 – 2.48 (m, 1H). **^{13}C{^{1}H} NMR** (101 MHz, chloroform-*d_3*) δ 166.7, 150.3, 131.6, 129.9, 126.9, 121.4, 53.9, 52.1, 47.5, 22.8. **^{19}F{^{1}H} NMR** (376 MHz, chloroform-*d_3*) δ -148.9, -194.8. **HRMS** (ESI) m/z calc. for $C_{14}H_{13}NO_4F_2Na$ [M+Na]$^+$ 320.07049, found 320.07054.

(B)*3-Methyl 1-phenyl 2,3-difluoro-3,4-dihydropyridine-1,3(2H)-dicarboxylate* (**50**):

R_f (pentane/ethyl acetate 80:20) 0.29. **^{1}H NMR** (400 MHz, chloroform-*d_3*) δ 8.07 – 7.90 (m, 1H), 7.46 – 7.40 (m, 2H), 7.34 – 7.28 (m, 1H), 7.22 – 7.16 (m, 2H), 6.64 – 6.39 (m, 1H), 4.99 – 4.66 (m, 1H), 3.80 (s, 3H), 3.09 – 2.92 (m, 1H), 2.86 – 2.60 (m, 1H). **^{19}F{^{1}H} NMR** (376 MHz, chloroform-*d_3*) δ -160.5, -194.3. **HRMS** (ESI) m/z calc. for $C_{14}H_{13}NO_4F_2Na$ [M+Na]$^+$ 320.07049, found 320.07061.

2.7. Deuterium Labeling

Synthesis of NMe_3BD_3

NMe_3BD_3 was synthesized after a modified procedure by Atkinson *et al.*[9] NMe_3BH_3 (500 mg) was dissolved in CH_2Cl_2 (1 mL). D_2O (2 mL) and D_2SO_4 (55 µL) were added afterwards and the resulting mixture was stirred overnight with rigorous stirring. The layers were separated, and the aqueous phase was washed with CH_2Cl_2 (3x). The combined organic layer was evaporated, and the resulting white solid was again dissolved as described above. This procedure was repeated two times to obtain NMe_3BD_3 as a white solid. The degree of deuteration was determined through ^{1}H NMR and was above 90%.

^{1}H NMR (400 MHz, chloroform-*d_3*) δ 2.65 (s, 9H).

Synthesis of piperidine derivative

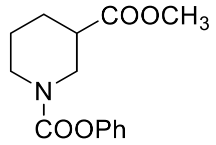

3-Methyl 1-phenyl piperidine-1,3-dicarboxylate **(S3):** A 4 mL screw-cap vial, equipped with a magnetic stir bar, was charged with methyl nicotinate (52 mg, 0.2 mmol, 1.0 equiv) and Pd/$C_{10\%}$ (10 mg). MeOH (1.0 mL) was added and the vial tightly closed. The mixture was stirred at 40 °C overnight under a H_2-atmosphere (5 bar). The product was purified by column chromatography (silica gel, pentane/ethyl acetate 80:20) to obtain the title compound as colorless oil (50 mg, 95% yield).

R_f (pentane/ethyl acetate 80:20) 0.38. **^{1}H NMR** (500 MHz, toluene-*d*$_8$) δ 7.11 – 7.03 (m, 4H), 6.93 – 6.88 (m, 1H), 4.18 – 4.04 (m, 1H), 3.80 – 3.69 (m, 1H), 3.35 (s, 3H), 3.19 – 3.03 (m, 1H), 2.84 – 2.70 (m, 1H), 2.32 – 2.20 (m, 1H), 1.77 – 1.64 (m, 1H), 1.50 – 1.14 (m, 3H). **^{13}C{^{1}H} NMR** (126 MHz, toluene-*d*$_8$) δ 172.9, 153.4, 152.8, 129.3, 125.1, 122.1, 51.2, 46.8, 45.0, 41.6, 27.5, 24.4. **HRMS** (ESI) m/z calculated for $C_{14}H_{17}NO_4Na$ $[M+Na]^+$ 286.1061, found 286.1045.

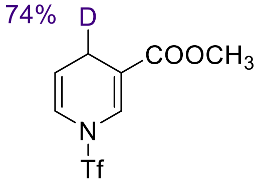

Methyl 1-((trifluoromethyl)sulfonyl)-1,4-dihydropyridine-3-carboxylate-4-d (**12**): The title compound was synthesized from methyl nicotinate (0.30 mmol, 41 mg, 1.0 equiv) using GP-A with NMe_3BD_3 (27 mg, 0.36 mmol, 1.2 equiv). The product was purified by column chromatography (pentane/diethylether 90:10) to afford **12** as a colorless oil (57 mg, 70% yield, 90:10 ratio of 1,4 DHP vs. other DHP).

(*Degree of Deuteration was assigned through ^{1}H NMR and ^{2}H NMR.)*

R_f (pentane/diethylether 80:20) 0.54. **^{1}H NMR** (400 MHz, benzene-*d*$_6$) δ 7.60 – 7.39 (m, 1H), 6.10 – 5.88 (m, 1H), 4.54 – 4.13 (m, 1H), 3.23 (s, 3H), 2.68 – 2.25 (m, 1H). **^{13}C{^{1}H} NMR** (101 MHz, benzene-*d*$_6$) δ 165.2, 130.3, 120.5, 120.0 (q, J = 324.7 Hz), 112.6, 110.9, 51.5, 21.8. **^{19}F{^{1}H} NMR** (377 MHz, benzene-*d*$_6$) δ -75.3. **HRMS** (ESI) m/z calc. for $C_8H_8NO_4SDF_3$ $[M+H]^+$ 273.0262, found 273.0262.

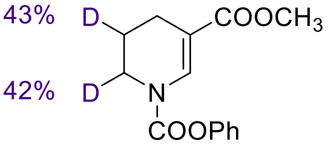

3-Methyl 1-phenyl 5,6-dihydropyridine-1,3(4H)-dicarboxylate-5-d (**52**): In a 4 mL screw-cap vial, equipped with magnetic stir bar, dihydropyridine **S1** (42 mg, 0.16 mmol, 1.0 equiv) and NMe_3BH_3 (13 mg, 1.0 equiv) were dissolved in MeCN (1.0 mL). $Pd_{2.5\%}/Pt_{2.5\%}/C$ (25 mg, 5 mol%, dried overnight at 110 °C) and D_2O (25 µL) were added and the vial tightly closed. The mixture was stirred for 16 h at room

temperature. The product was purified by column chromatography (silica gel, pentane/ethyl acetate 90:10) to obtain **52** as a white solid (30 mg, 72% yield).

(Degree of Deuteration was assigned through ^{1}H NMR and ^{2}H NMR.)

R$_f$ (pentane/ethyl acetate 90:10) 0.24. **^{1}H NMR** (500 MHz, toluene-d_8, T = 368 K) 8.29 – 8.01 (m, 1H), 7.08 – 6.95 (m, 4H), 6.90 (tq, J = 8.4, 1.5 Hz, 1H), 3.49 (s, 3H), 3.36 – 3.24 (m, 1H), 2.15 (d, J = 6.3 Hz, 2H), 1.43 – 1.35 (m, 1H). **^{13}C{^{1}H} NMR** (126 MHz, toluene-d_8, T = 368 K) δ 167.1, 152.0, 151.5, 135.3, 129.5, 125.8, 121.8, 110.3, 50.8, 43.0, 30.4, 21.4. **HRMS** (ESI) m/z calc. for $C_{14}H_{13}NO_4D_2Na$ [M+Na]$^+$ 286.10188, found 286.10144.

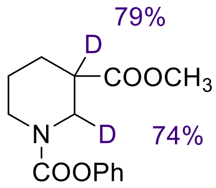

3-Methyl 1-phenyl piperidine-1,3-dicarboxylate-2,3-d$_2$ (**53**): A 4 mL screw-cap vial, equipped with a magnetic stir bar, was charged with tetrahydropyridine **40** (0.20 mmol, 52 mg, 1.0 equiv) and Pd/C$_{10\%}$ (12 mg, 5 mol%, dried overnight at 110 °C). Methanol-d_4 (1.0 mL) and D_2O (10 µL) were added and the vial tightly closed. The mixture was stirred at 40 °C overnight under a D_2-atmosphere (5 bar). The product was purified by column chromatography (silica gel, pentane to pentane/ethyl acetate 95:5) to obtain **53** as a white solid (44 mg, 83% yield).

(*Degree of Deuteration was assigned through ^{1}H NMR and ^{2}H NMR.)*

R$_f$ (pentane/diethylether 80:20) 0.36. **^{1}H NMR** (500 MHz, toluene-d_8) δ 7.16 – 7.04 (m, 4H), 6.95 – 6.86 (m, 1H), 3.89 – 3.70 (m, 1H), 3.30 (s, 3H), 3.13 – 2.87 (m, 1H), 2.81 – 2.42 (m, 1H), 1.76 – 1.59 (m, 1H), 1.46 – 1.25 (m, 2H), 1.20 – 1.06 (m, 1H). **^{13}C{^{1}H} NMR** (126 MHz, toluene-d_8) δ 172.8, 153.2, 152.3, 137.5, 125.1, 122.1, 51.1, 46.2, 44.6, 40.7, 27.2, 24.3. **HRMS** (ESI) m/z calc. for $C_{14}H_{15}D_2NO_4Na$ [M+Na]$^+$ 288.11808, found 288.11624.

3. Analytical Data

3.1. X-Ray Data

X-ray crystal structure analysis of S1: A colorless plate-like specimen of $C_{14}H_{13}NO_4$, approximate dimensions 0.058 mm x 0.085 mm x 0.158 mm, was used for the X-ray crystallographic analysis. The X-ray intensity data were measured on a Bruker D8 Venture PHOTON III Diffractometer system equipped with a micro focus tube Cu Ims (CuK_α, λ = 1.54178 Å) and a MX mirror monochromator. A total of 1895 frames were collected. The total exposure time was 26.03 hours. The frames were integrated with the Bruker SAINT software package using a wide-frame algorithm. The integration of the data using a monoclinic unit cell yielded a total of 2087 reflections to a maximum θ angle of 66.73° (0.84 Å resolution), of which 2087 were independent (average redundancy 1.000, completeness = 97.6%, R_{int} = 4.75%, R_{sig} = 2.81%) and 1957 (93.77%) were greater than $2\sigma(F^2)$. The final cell constants of a = 6.8124(3) Å, b = 7.3253(3) Å, c = 24.3352(11) Å, β = 95.633(2)°, volume = 1208.53(9) $Å^3$, are based upon the refinement of the XYZ-centroids of 9956 reflections above 20 σ(I) with 14.13° < 2θ < 133.4°. Data were corrected for absorption effects using the Multi-Scan method (SADABS). The ratio of minimum to maximum apparent transmission was 0.860. The calculated minimum and maximum transmission coefficients (based on crystal size) are 0.8740 and 0.9510. The structure was solved and refined using the Bruker SHELXTL Software Package, using the space group $P2_1/n$, with Z = 4 for the formula unit, $C_{14}H_{13}NO_4$. The final anisotropic full-matrix least-squares refinement on F^2 with 174 variables converged at R1 = 9.29%, for the observed data and wR2 = 24.53% for all data. The goodness-of-fit was 1.206. The largest peak in the final difference electron density synthesis was 0.473 $e^-/Å^3$ and the largest hole was -0.523 $e^-/Å^3$ with an RMS deviation of 0.114 $e^-/Å^3$. On the basis of the final model, the calculated density was 1.425 g/cm^3 and F(000), 544 e^-. CCDC Nr.: 2063435.

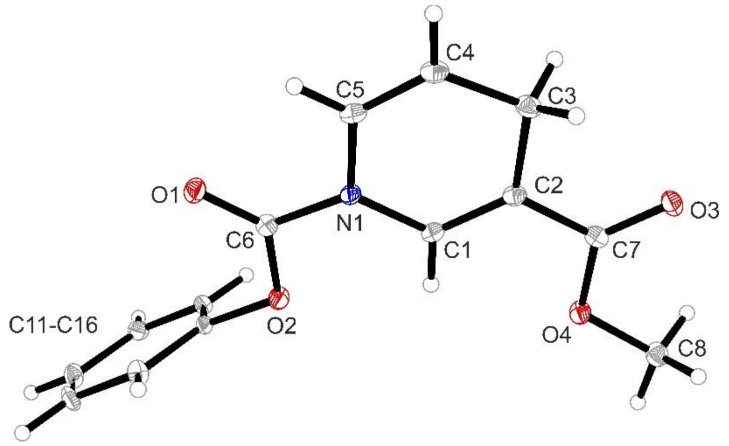

Figure S1: Crystal structure of compound S**1**.
Thermal ellipsoids are shown at 30% probability.

X-ray crystal structure analysis of 40: A colorless plate-like specimen of $C_{14}H_{15}NO_4$, approximate dimensions 0.046 mm x 0.177 mm x 0.202 mm, was used for the X-ray crystallographic analysis. The X-ray intensity data were measured on a Bruker D8 Venture PHOTON III Diffractometer system equipped with a micro focus tube Cu Ims (CuK_α, λ = 1.54178 Å) and a MX mirror monochromator. A total of 1316 frames were

collected. The total exposure time was 17.34 hours. The frames were integrated with the Bruker SAINT software package using a wide-frame algorithm. The integration of the data using a monoclinic unit cell yielded a total of 2204 reflections to a maximum θ angle of 66.82° (0.84 Å resolution), of which 2204 were independent (average redundancy 1.000, completeness = 98.3%, R_{int} = 9.53%, R_{sig} = 5.03%) and 1833 (83.17%) were greater than 2σ(F^2). The final cell constants of a = 6.9893(3) Å, b = 7.4629(3) Å, c = 24.1834(9) Å, β = 94.544(2)°, volume = 1257.45(9) Å^3, are based upon the refinement of the XYZ-centroids of 8336 reflections above 20 σ(I) with 7.334° < 2θ < 133.6°. Data were corrected for absorption effects using the Multi-Scan method (SADABS). The ratio of minimum to maximum apparent transmission was 0.759. The calculated minimum and maximum transmission coefficients (based on crystal size) are 0.8480 and 0.9620. The structure was solved and refined using the Bruker SHELXTL Software Package, using the space group $P2_1/c$, with Z = 4 for the formula unit, $C_{14}H_{15}NO_4$. The final anisotropic full-matrix least-squares refinement on F^2 with 175 variables converged at R1 = 9.50%, for the observed data and wR2 = 24.94% for all data. The goodness-of-fit was 1.134. The largest peak in the final difference electron density synthesis was 0.442 e$^-$/Å^3 and the largest hole was -0.372 e$^-$/Å^3 with an RMS deviation of 0.094 e$^-$/Å^3. On the basis of the final model, the calculated density was 1.380 g/cm^3 and F(000), 552 e$^-$. CCDC Nr.: 2063436.

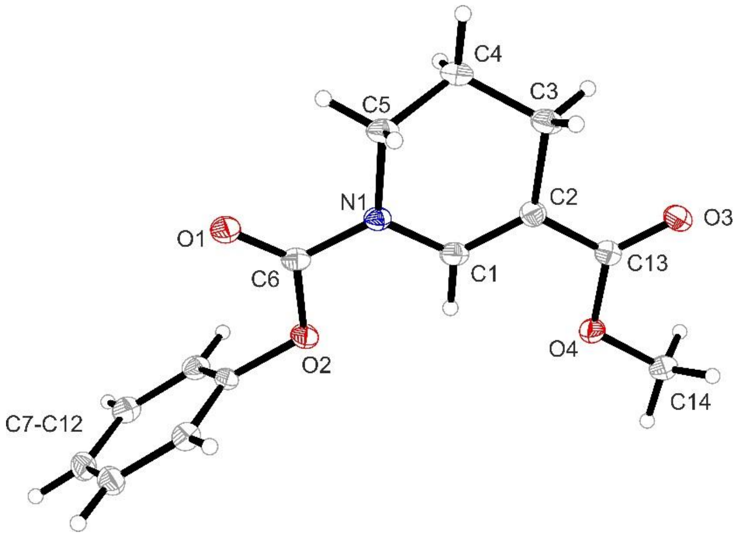

Figure S2: Crystal structure of compound **40**.
Thermal ellipsoids are shown at 30% probability.

3.2. NMR Data

^{1}H NMR (599 MHz, benzene-d_6, T = 338 K) – compound **S1**

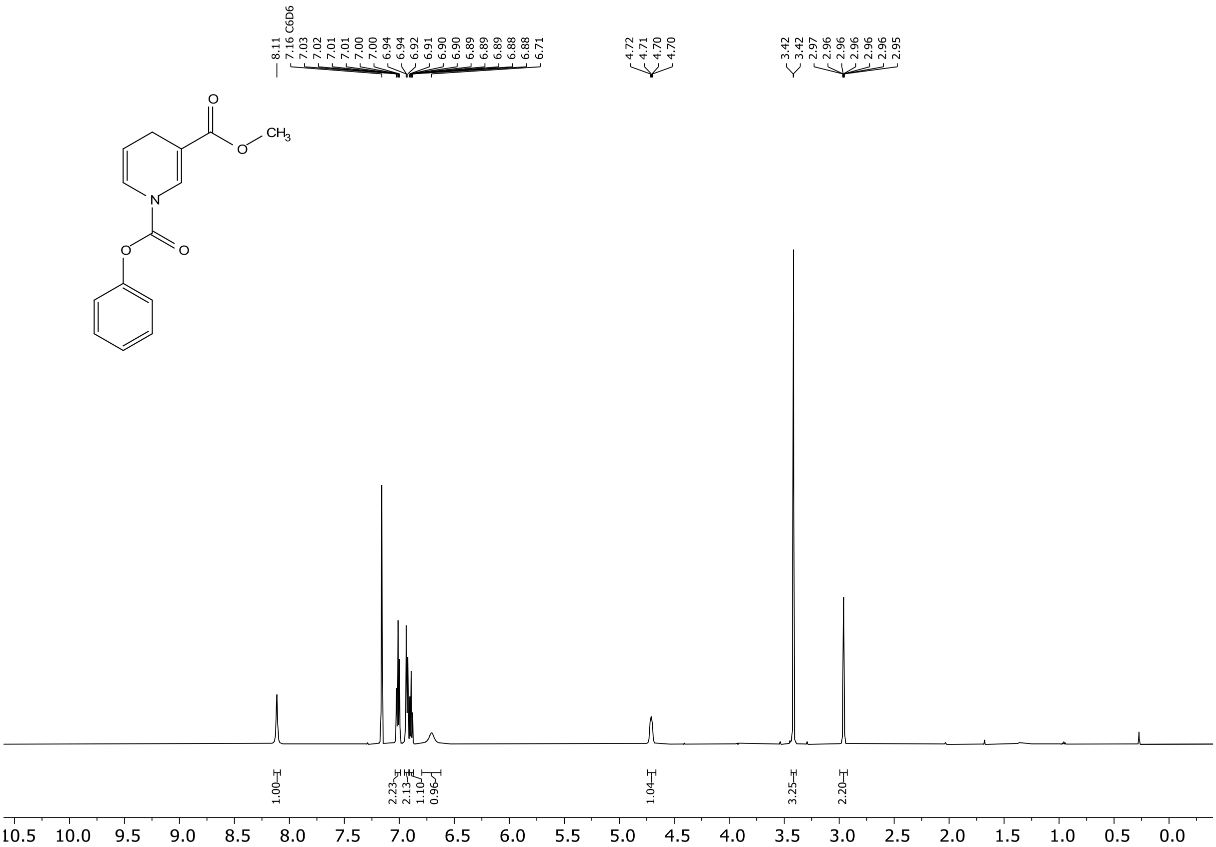

$^{13}C\{^{1}H\}$ NMR (151 MHz, benzene-d_6, T = 338 K) – compound **S1**

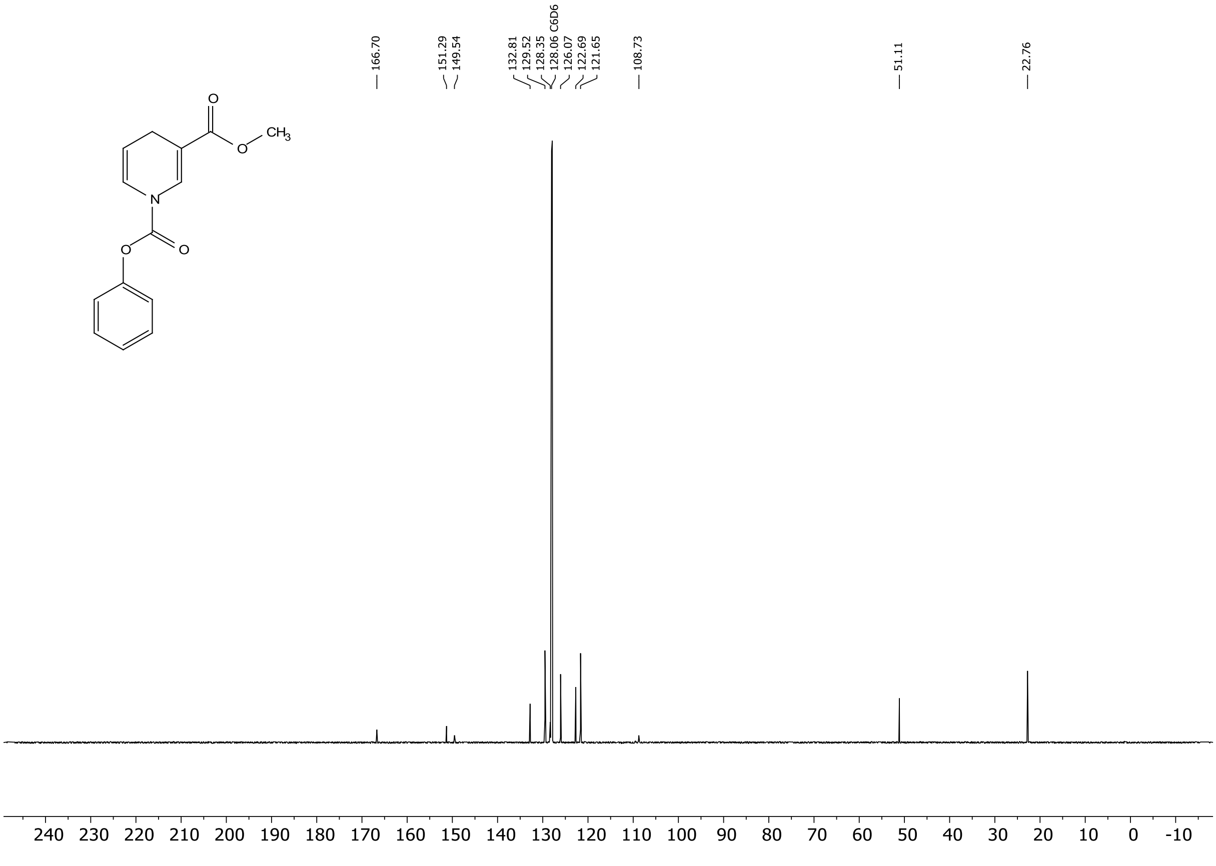

^{1}**H NMR** (400 MHz, benzene-d_6) – compound **1**

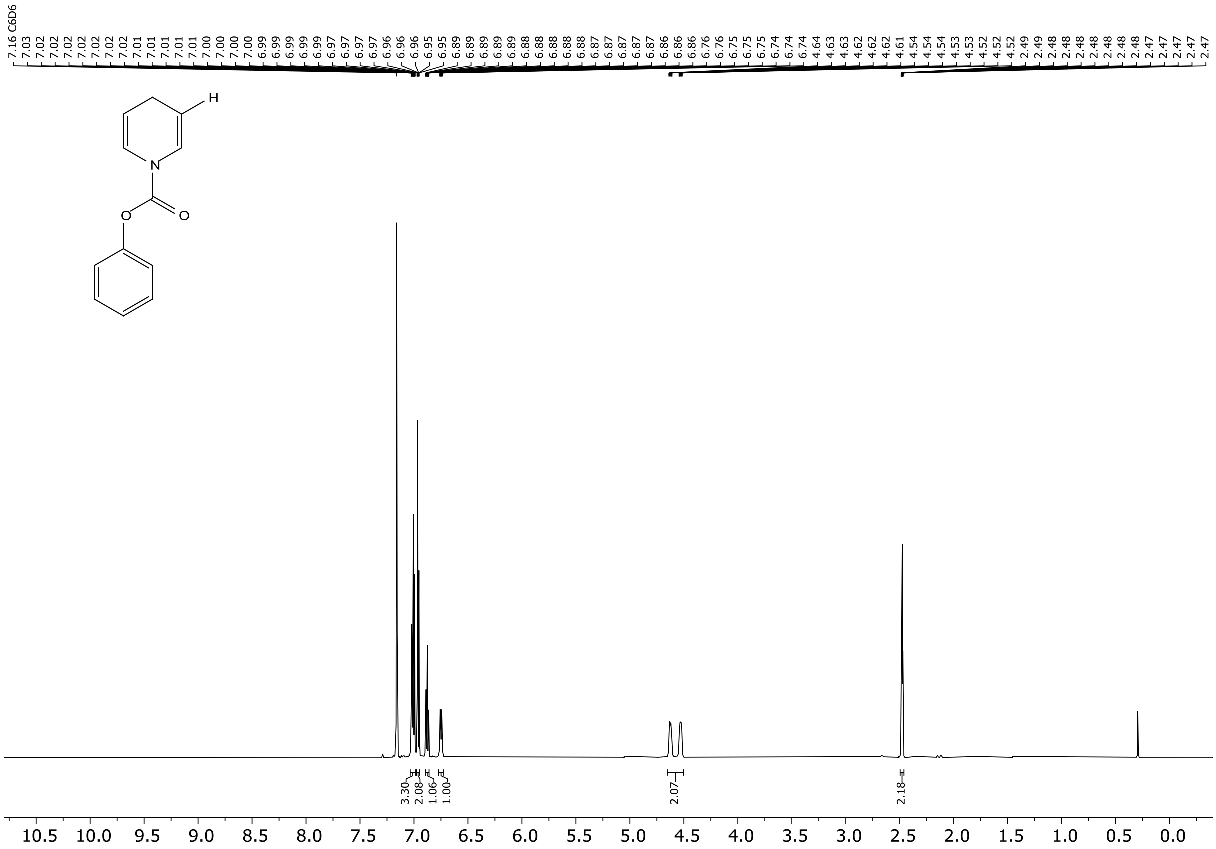

^{13}**C{^{1}H} NMR** (101 MHz, benzene-d_6) – compound **1**

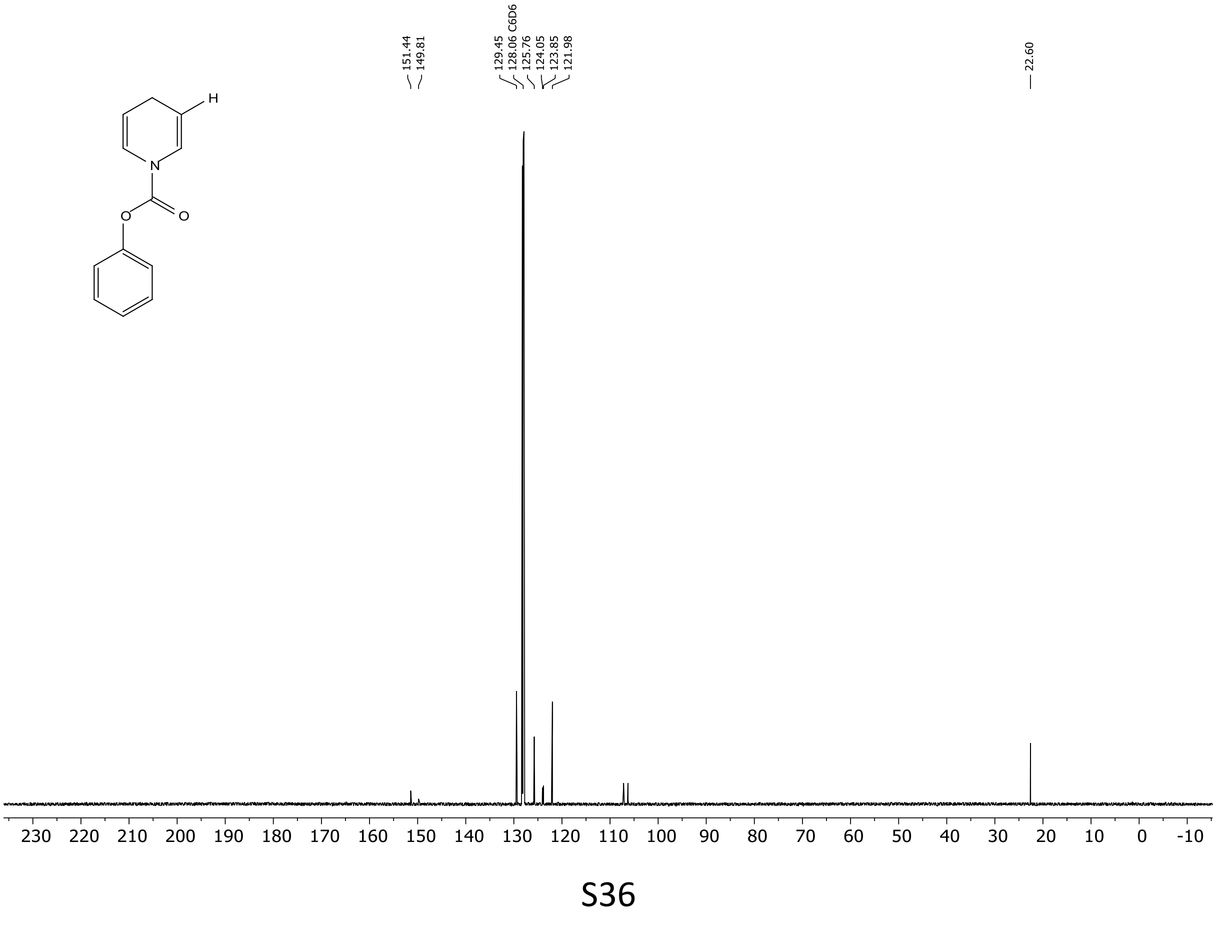

^{1}H NMR (400 MHz, benzene-d_6) – compound **2**

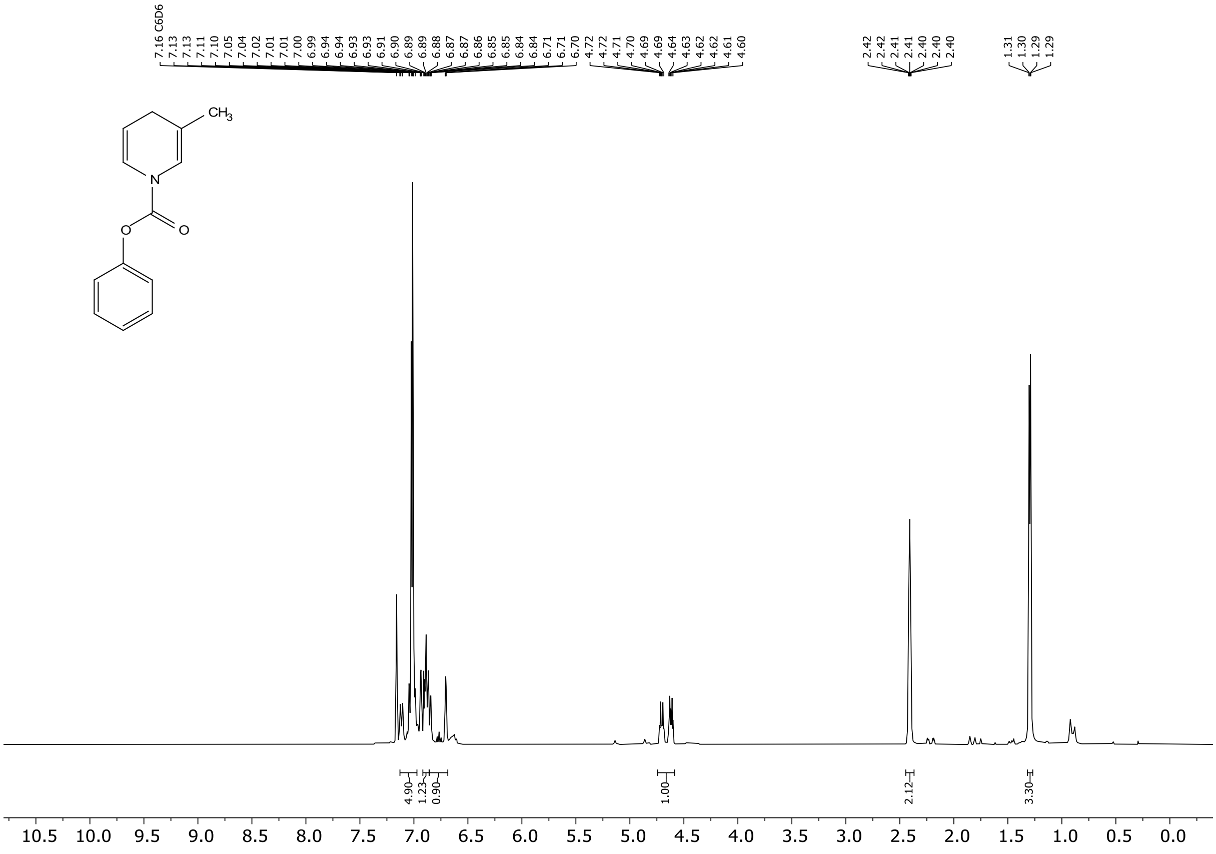

^{13}C{^{1}H} NMR (101 MHz, benzene-d_6) – compound **2**

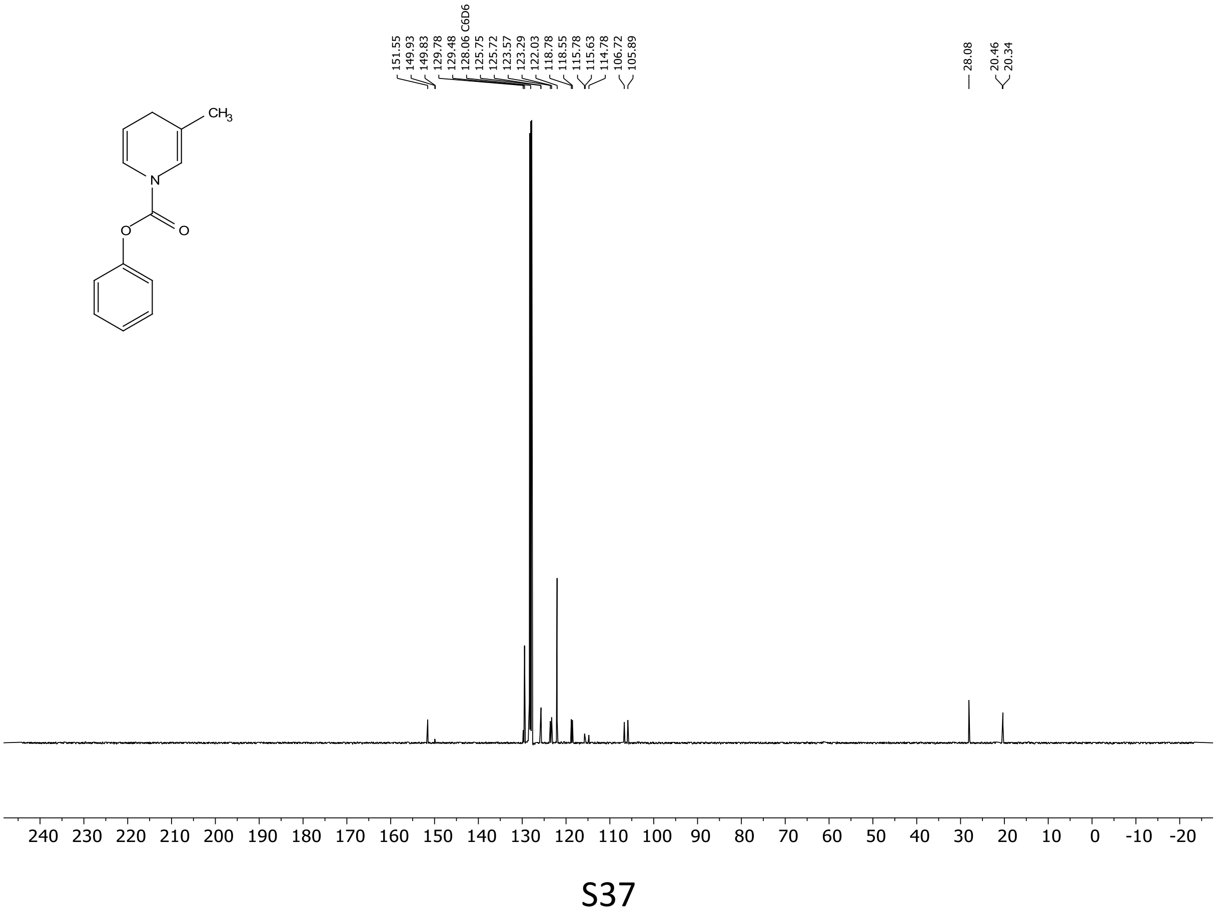

^{1}H NMR (400 MHz, benzene-d_6) – compound **3**

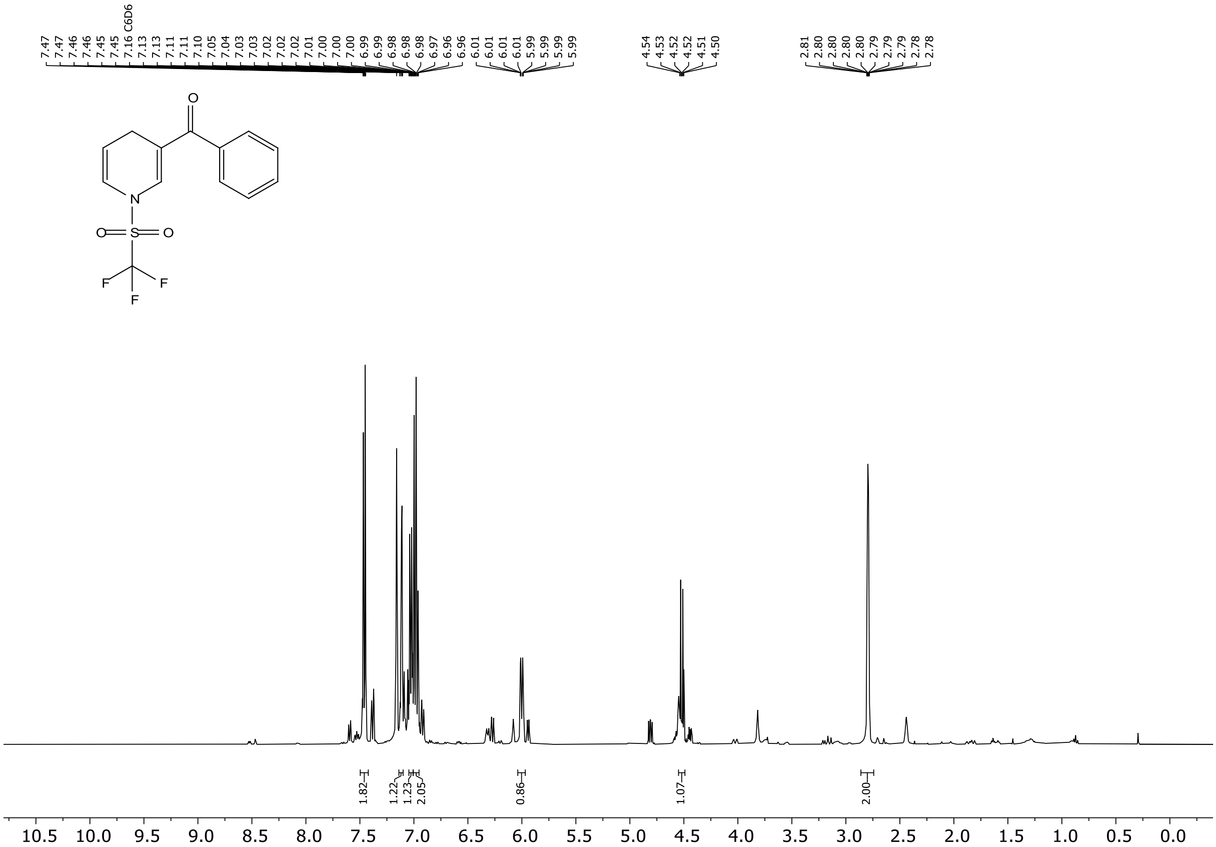

^{13}C{^{1}H} NMR (101 MHz, benzene-d_6) – compound **3**

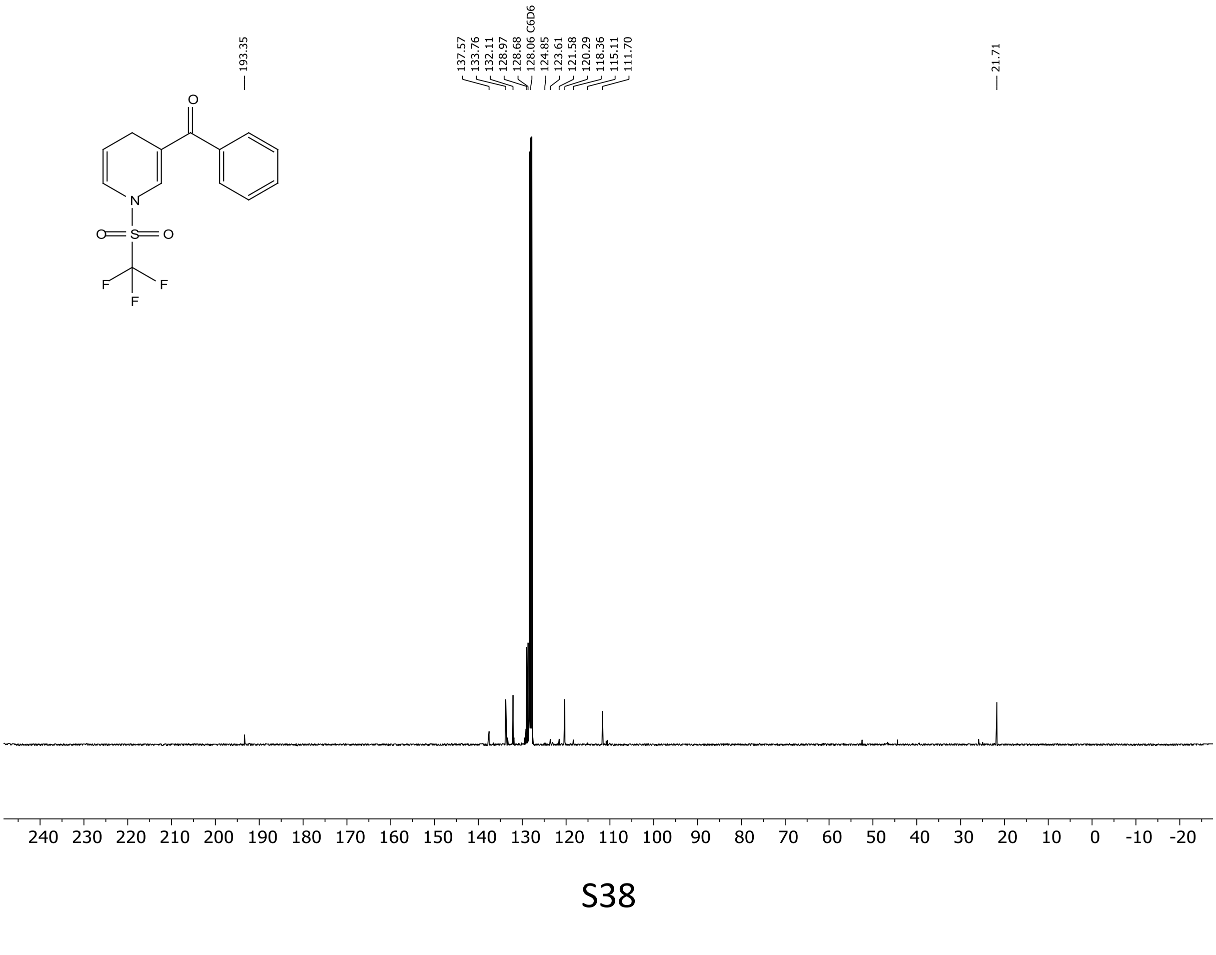

$^{19}F\{^{1}H\}$ NMR (377 MHz, benzene-d_6) – compound **3**

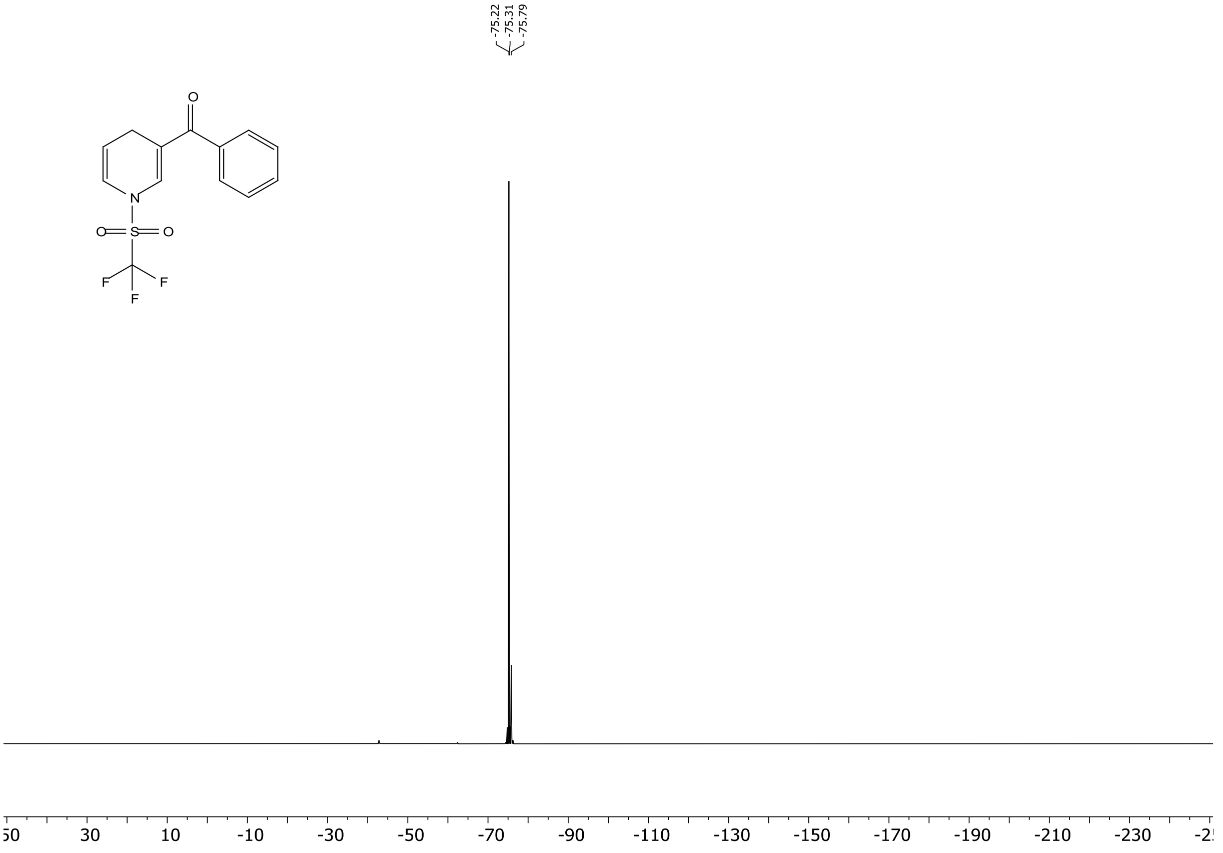

^{1}H NMR (400 MHz, benzene-d_6) – compound **4**

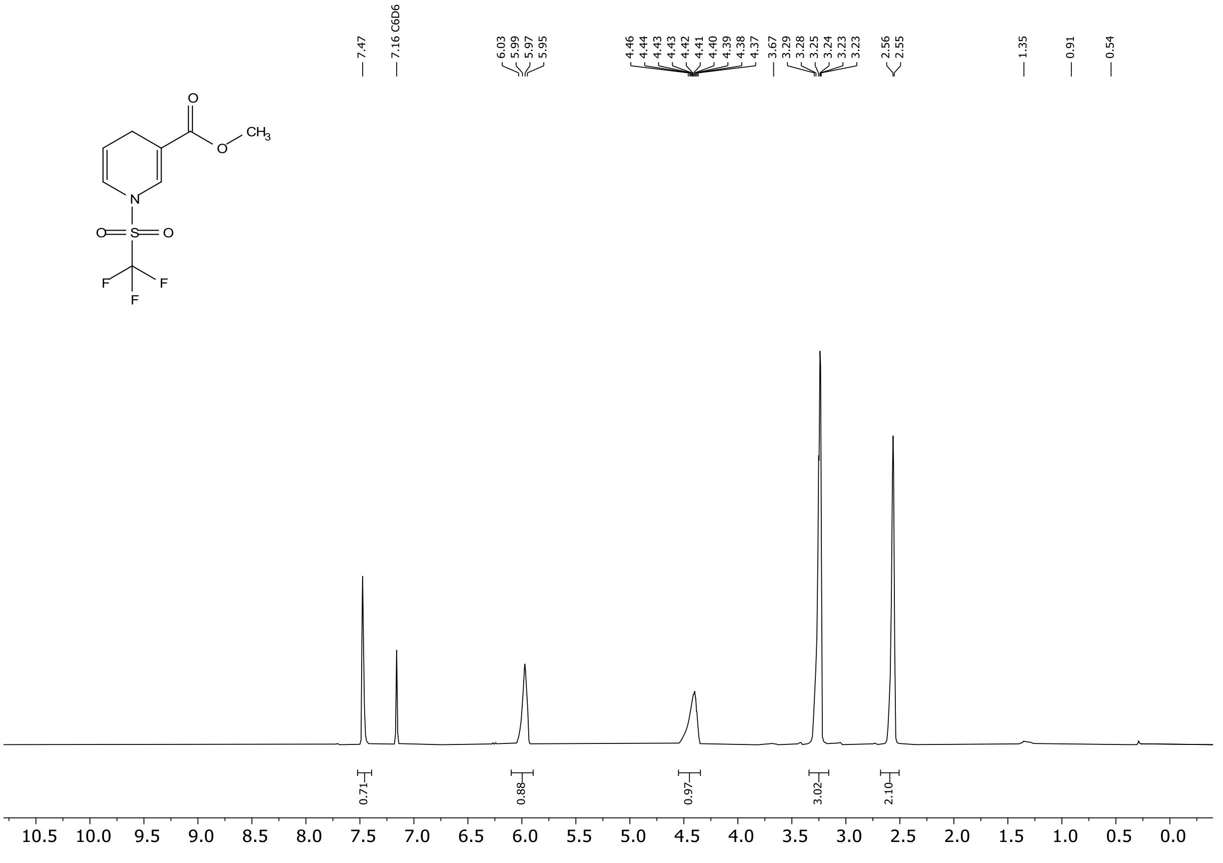

^{13}C{^{1}H} NMR (101 MHz, benzene-d_6) – compound **4**

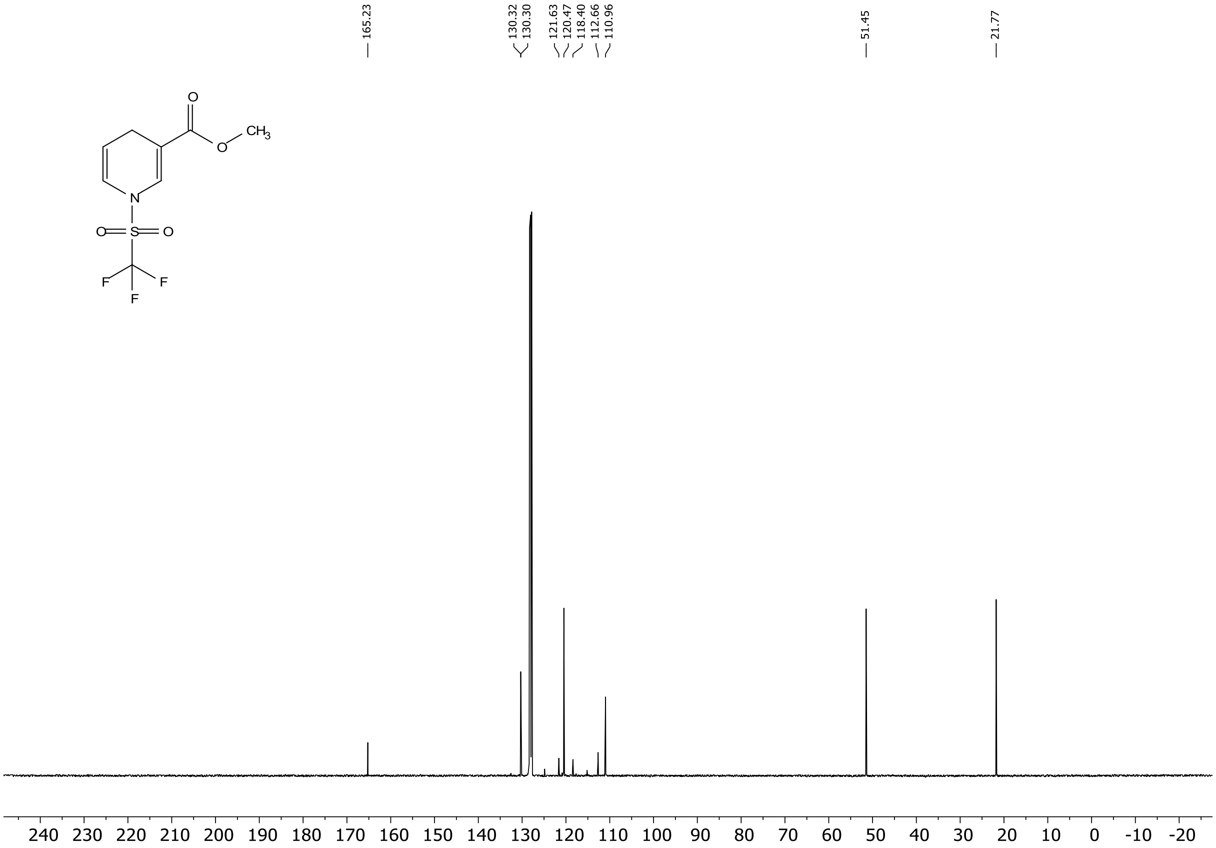

^{19}F{^{1}H} NMR (377 MHz, benzene-d_6) – compound **4**

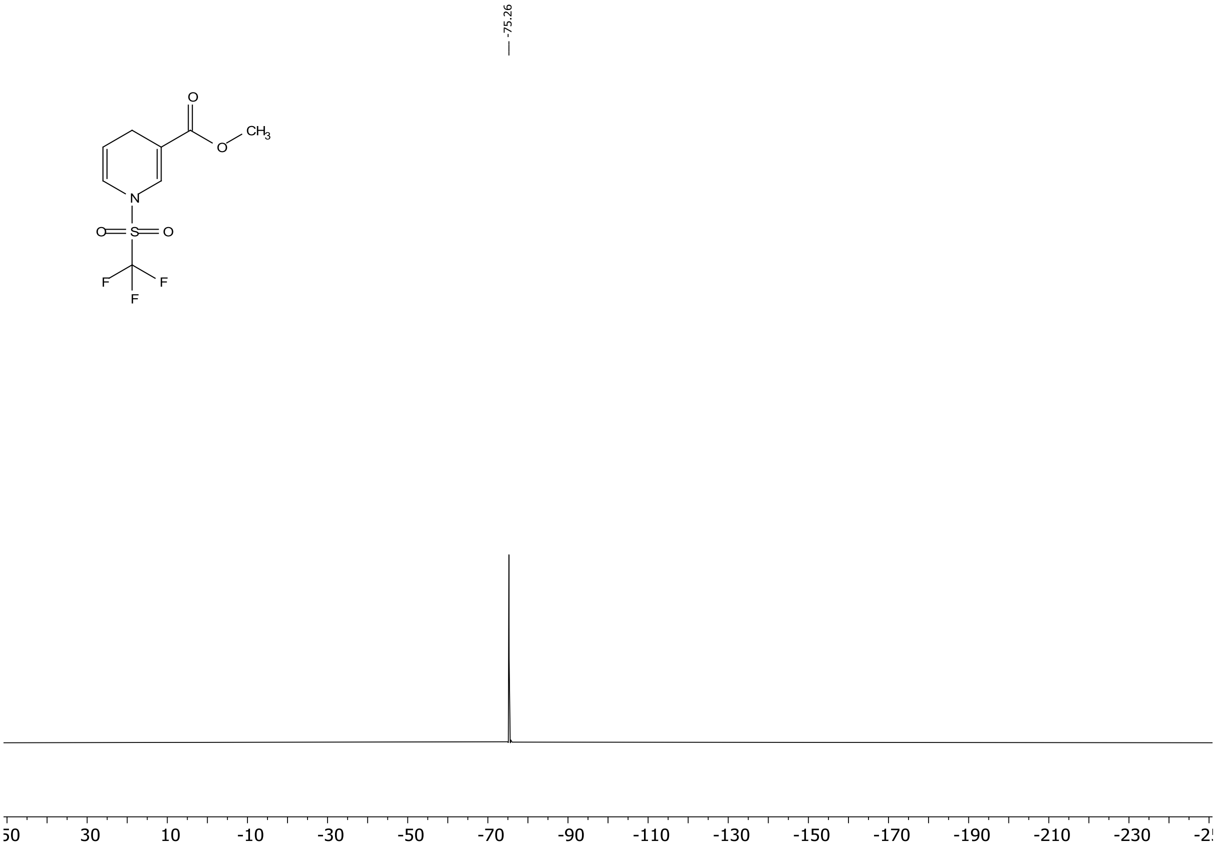

^{1}H NMR (400 MHz, benzene-d_6) – compound **5**

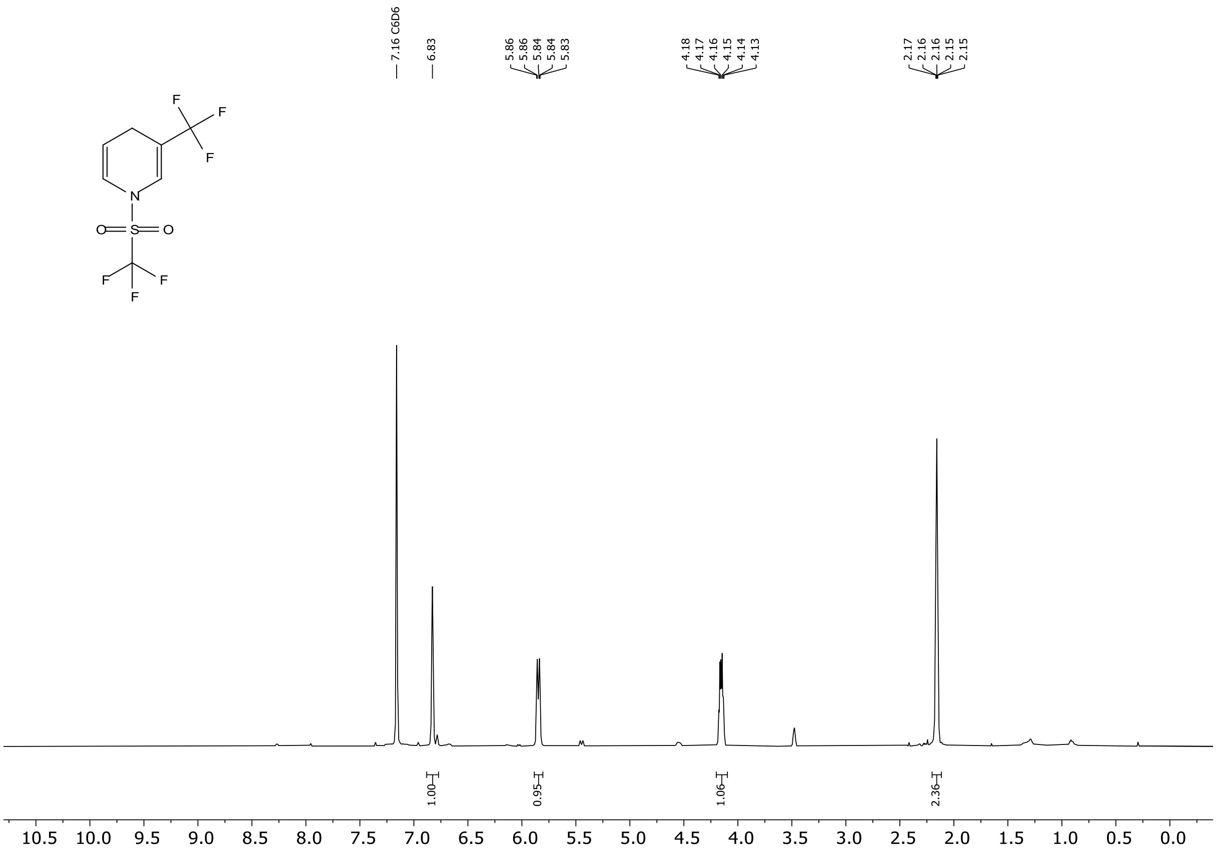

^{13}C{^{1}H} NMR (101 MHz, benzene-d_6) – compound **5**

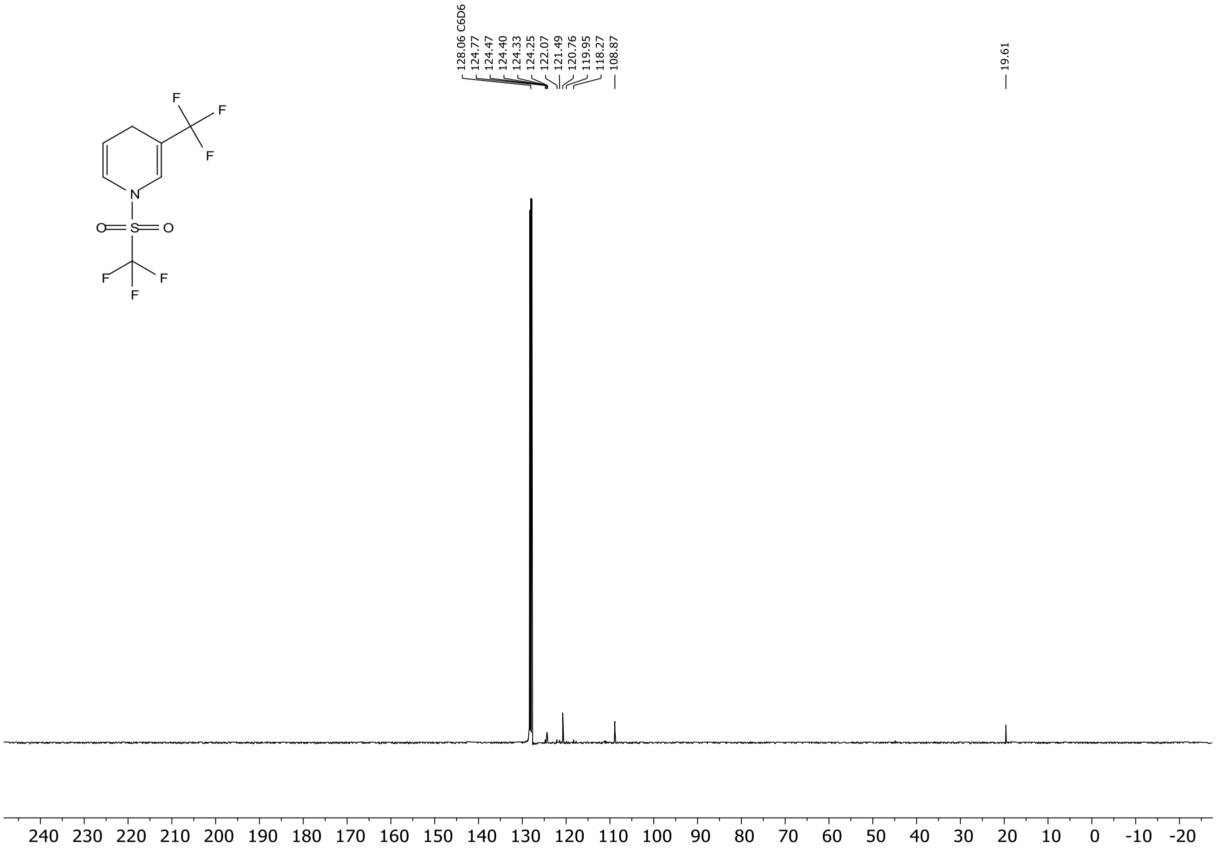

^{19}F{^{1}H} NMR (377 MHz, benzene-d_6) – compound **5**

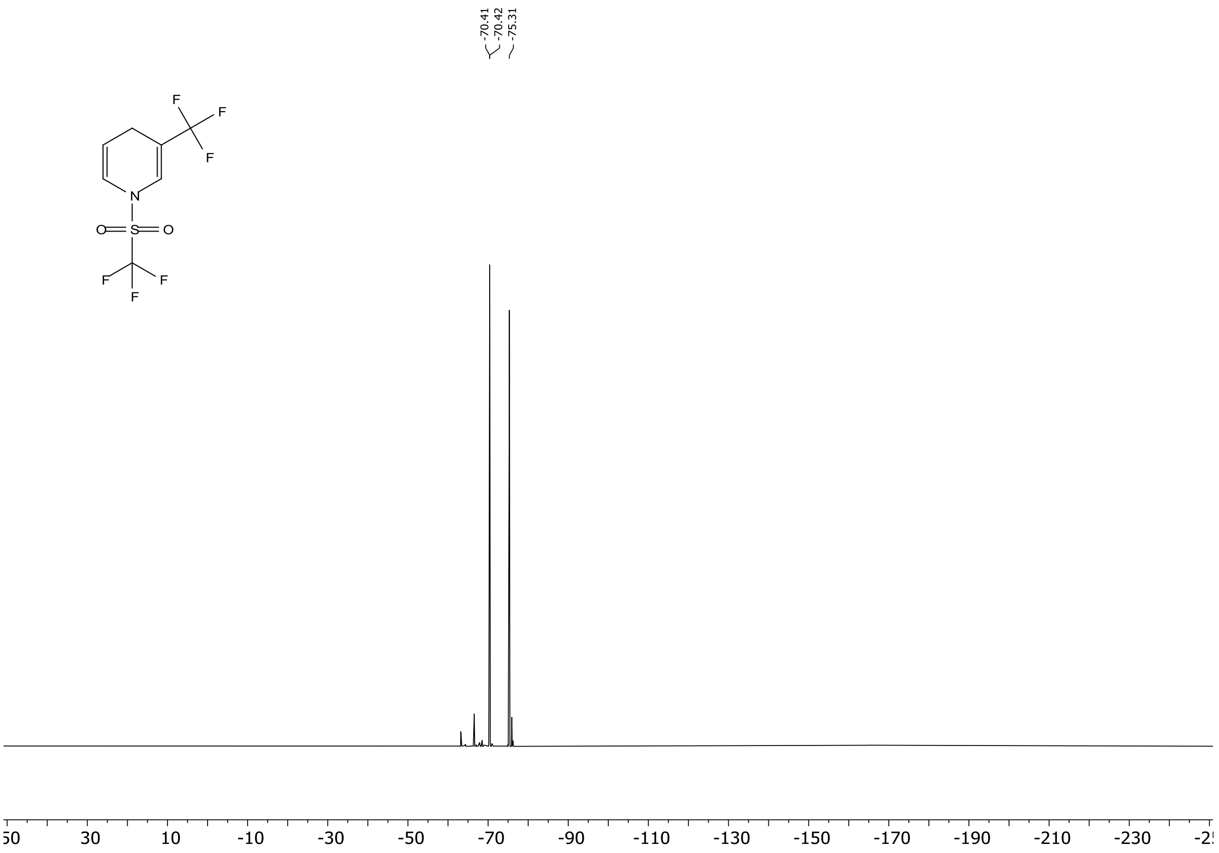

^{1}H NMR (599 MHz, benzene-d_6) – compound **6**

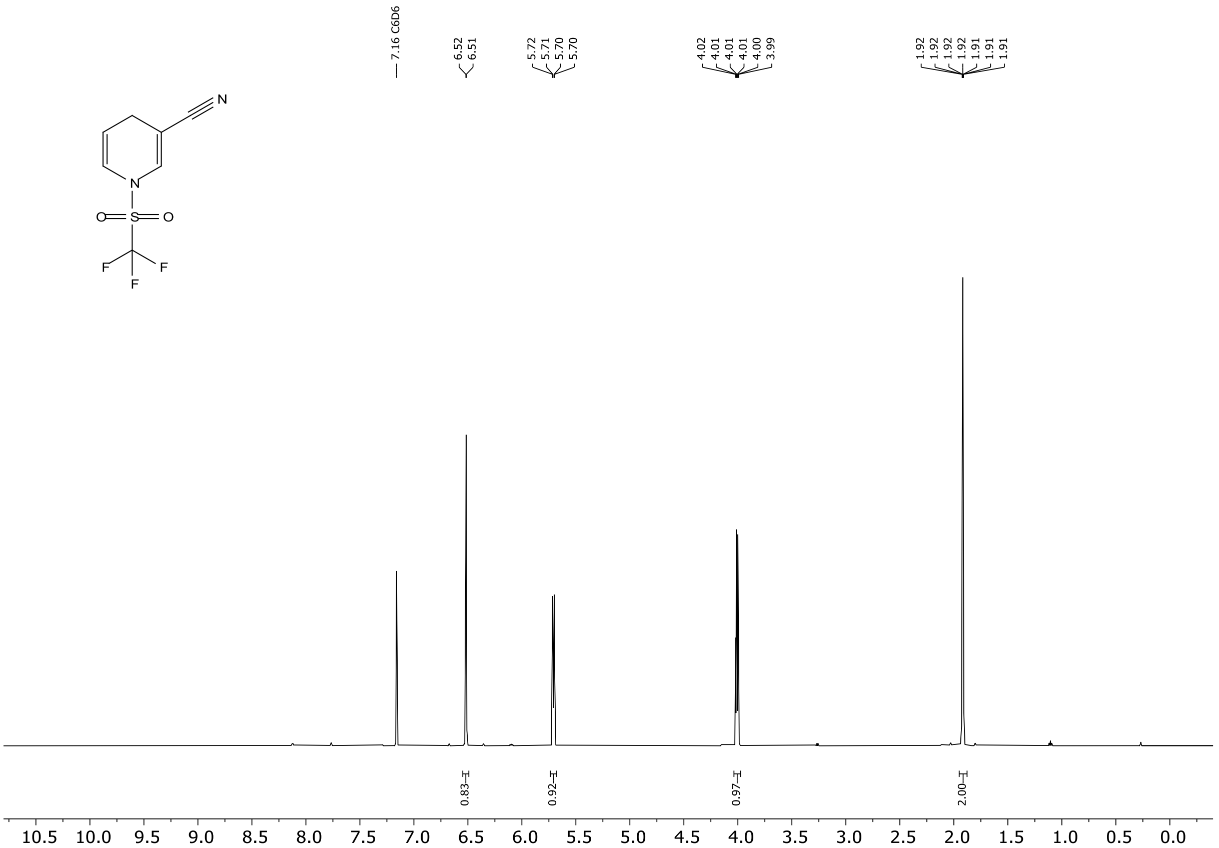

$^{13}C\{^1H\}$ NMR (151 MHz, benzene-d_6) – compound **6**

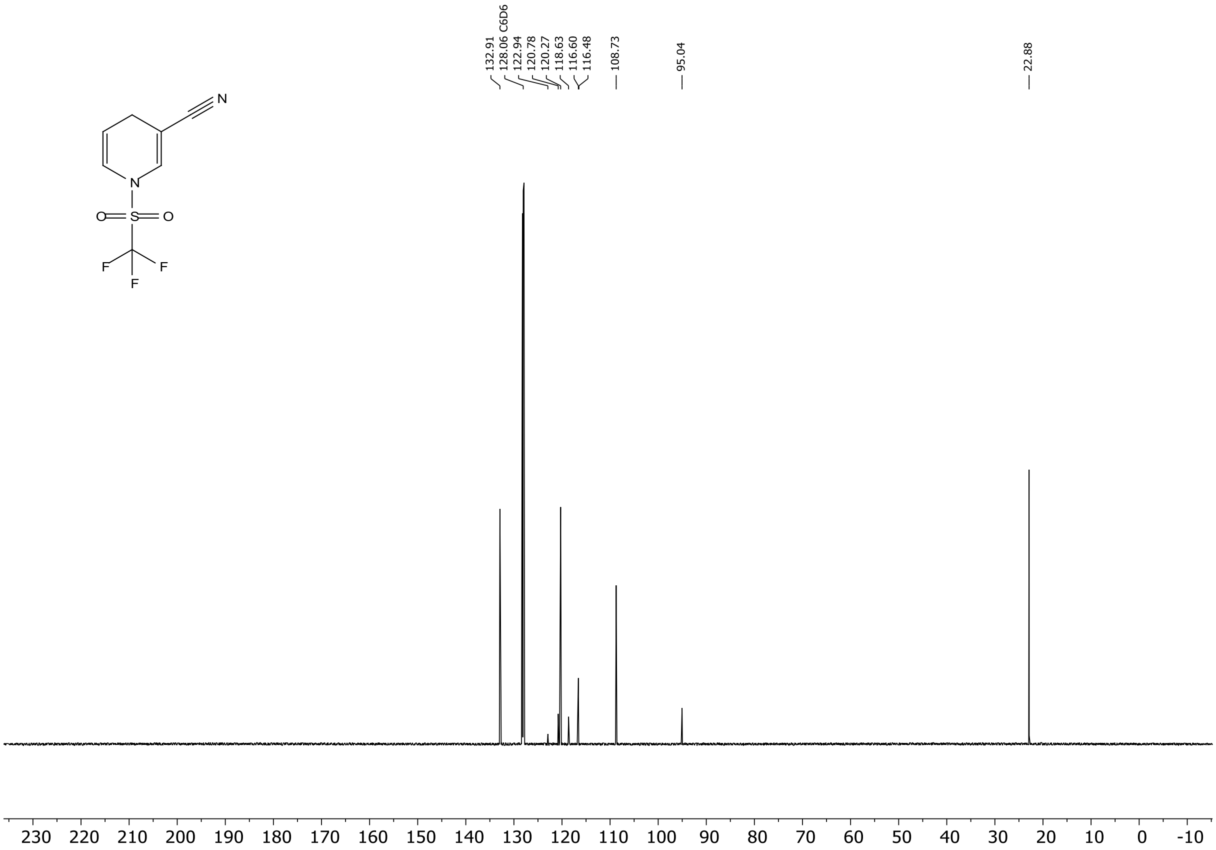

$^{19}F\{^1H\}$ NMR (564 MHz, benzene-d_6) – compound **6**

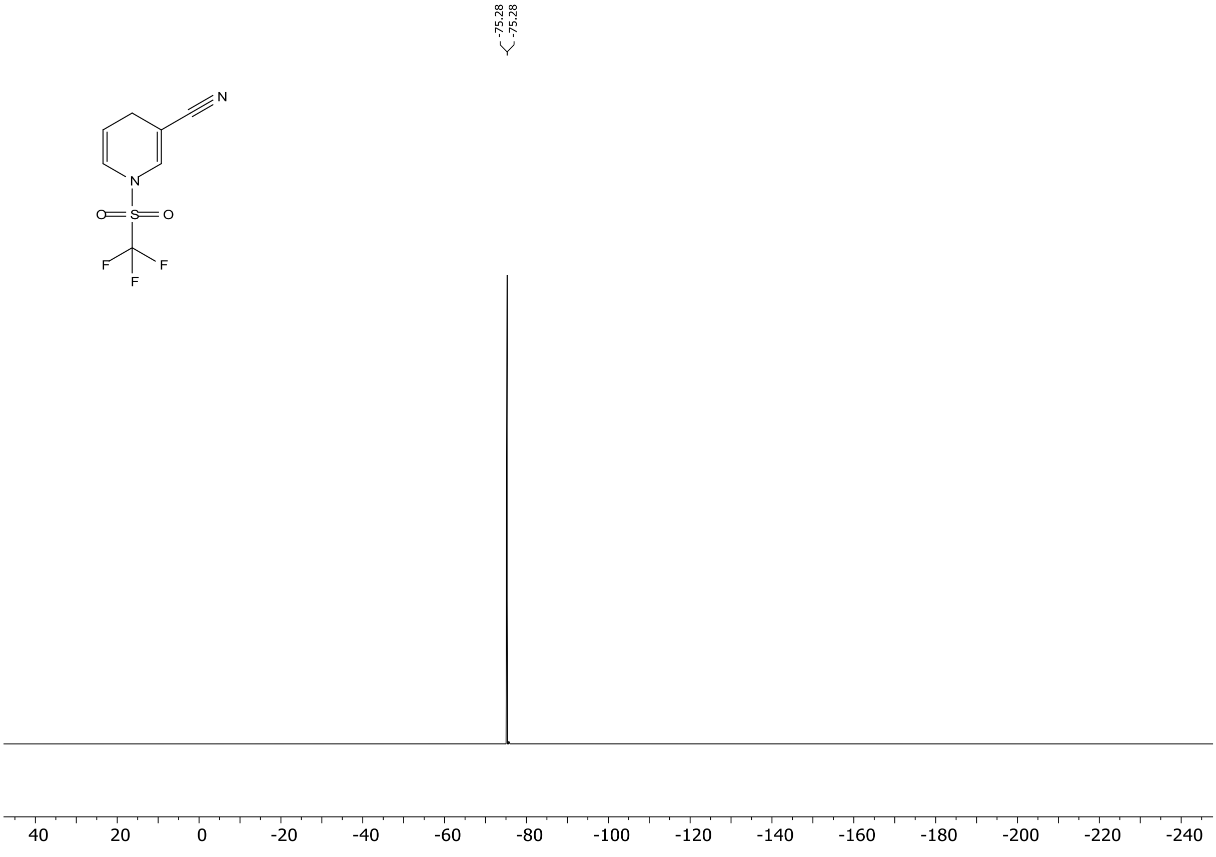

^{1}H NMR (400 MHz, benzene-d_6) – compound **7**

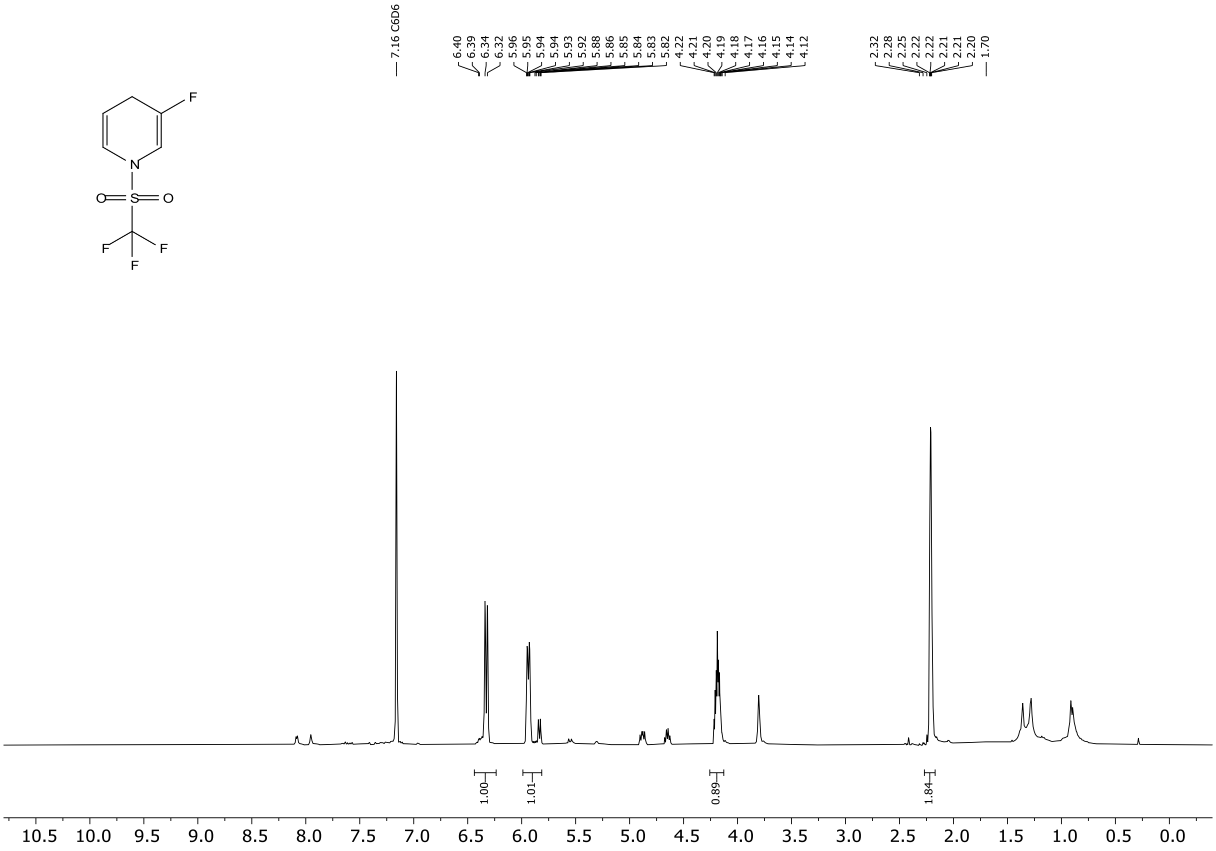

^{13}C{^{1}H} NMR (101 MHz, benzene-d_6) – compound **7**

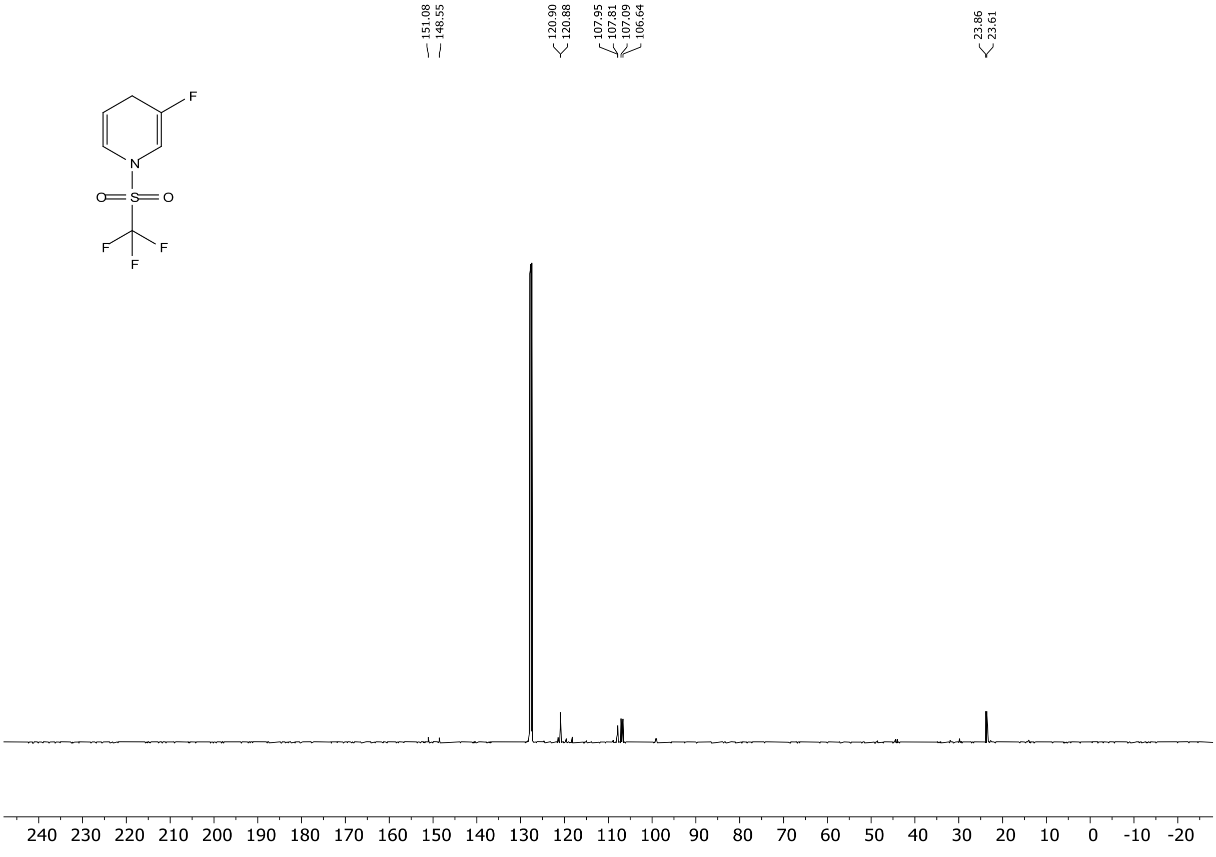

$^{19}F\{^{1}H\}$ NMR (377 MHz, benzene-d_6) – compound **7**

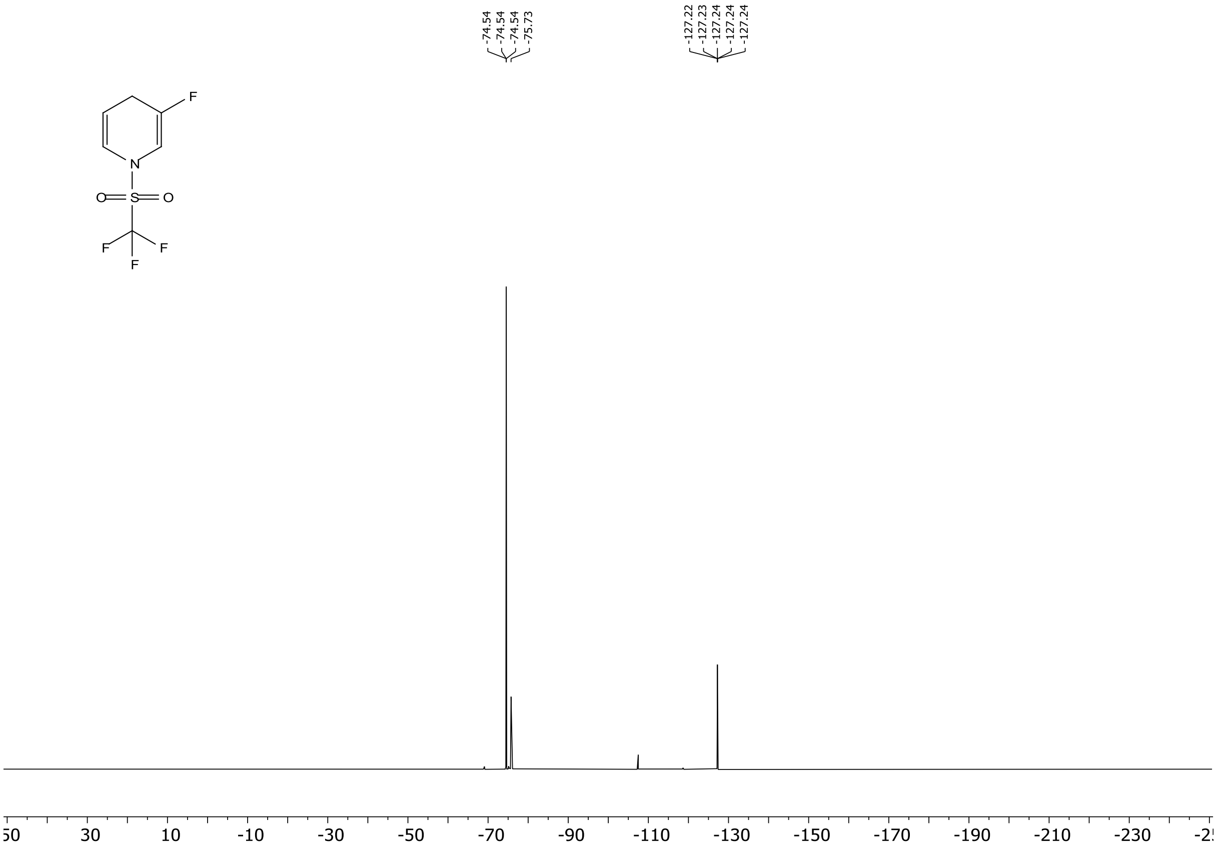

^{1}H NMR (400 MHz, benzene-d_6) – compound **8**

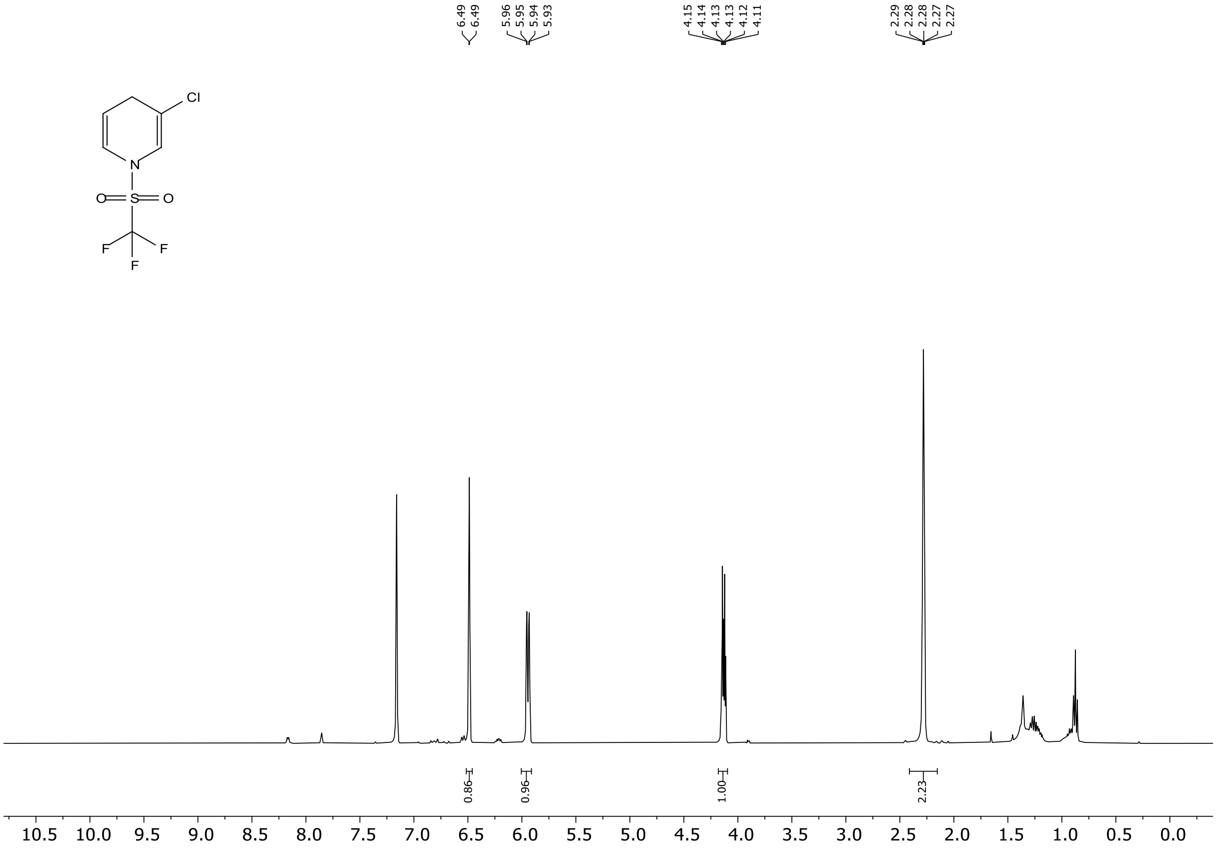

^{13}C{^{1}H} NMR (101 MHz, benzene-d_6) – compound **8**

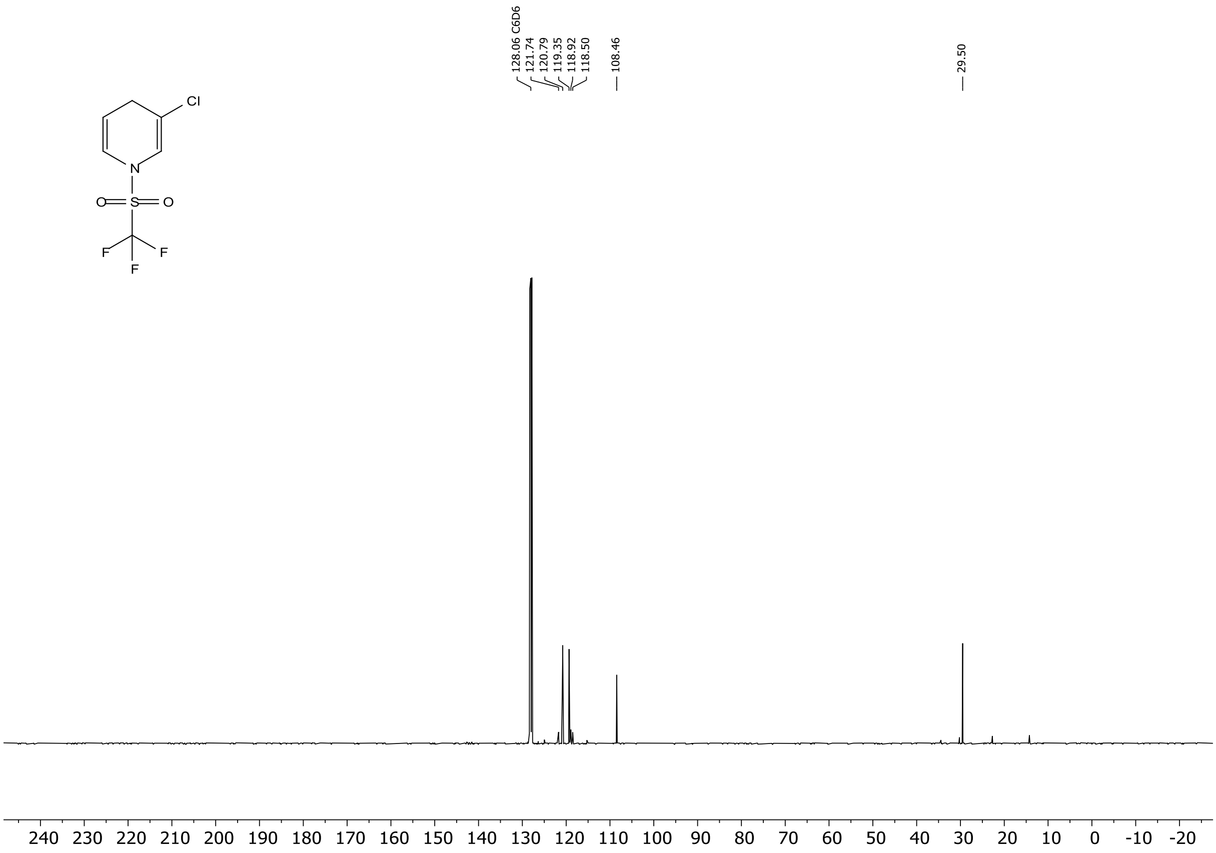

^{19}F{^{1}H} NMR (377 MHz, benzene-d_6) – compound **8**

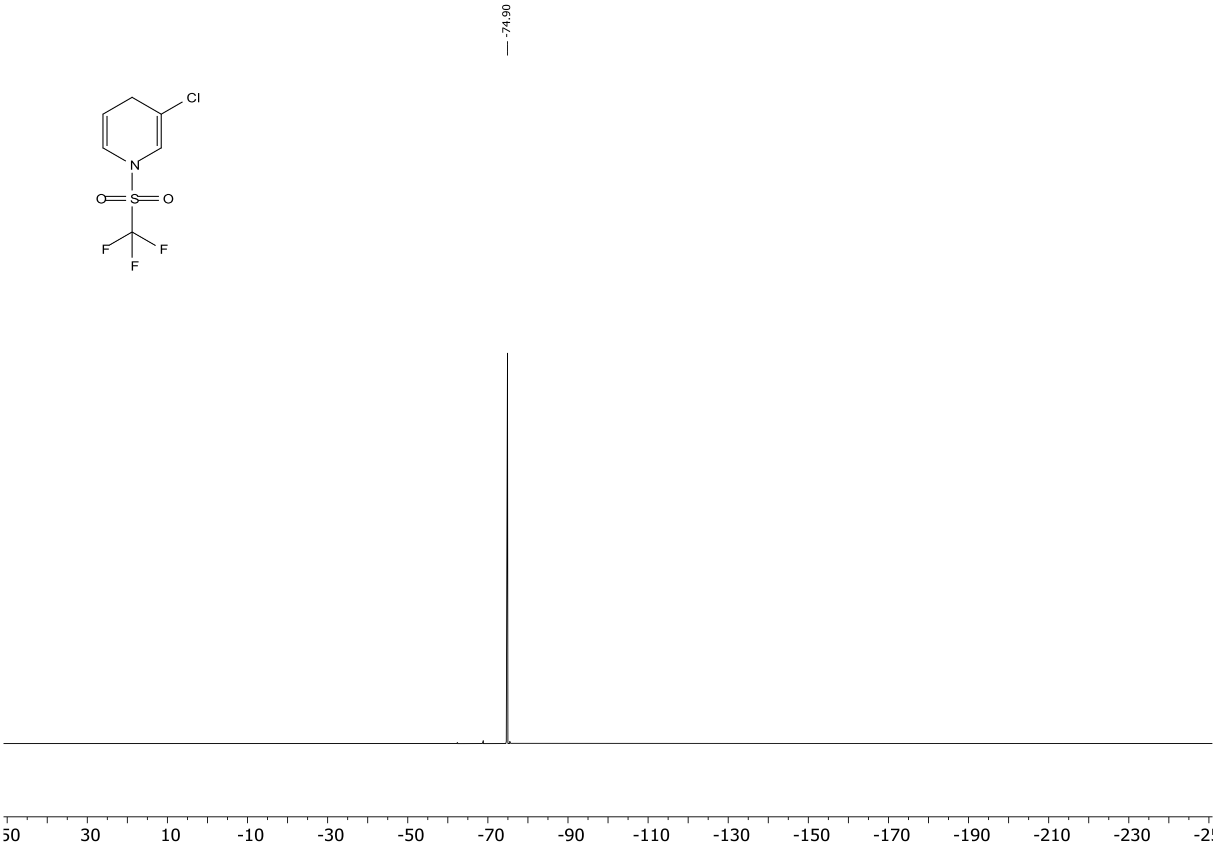

^{1}H NMR (400 MHz, benzene-d_6) – compound **9**

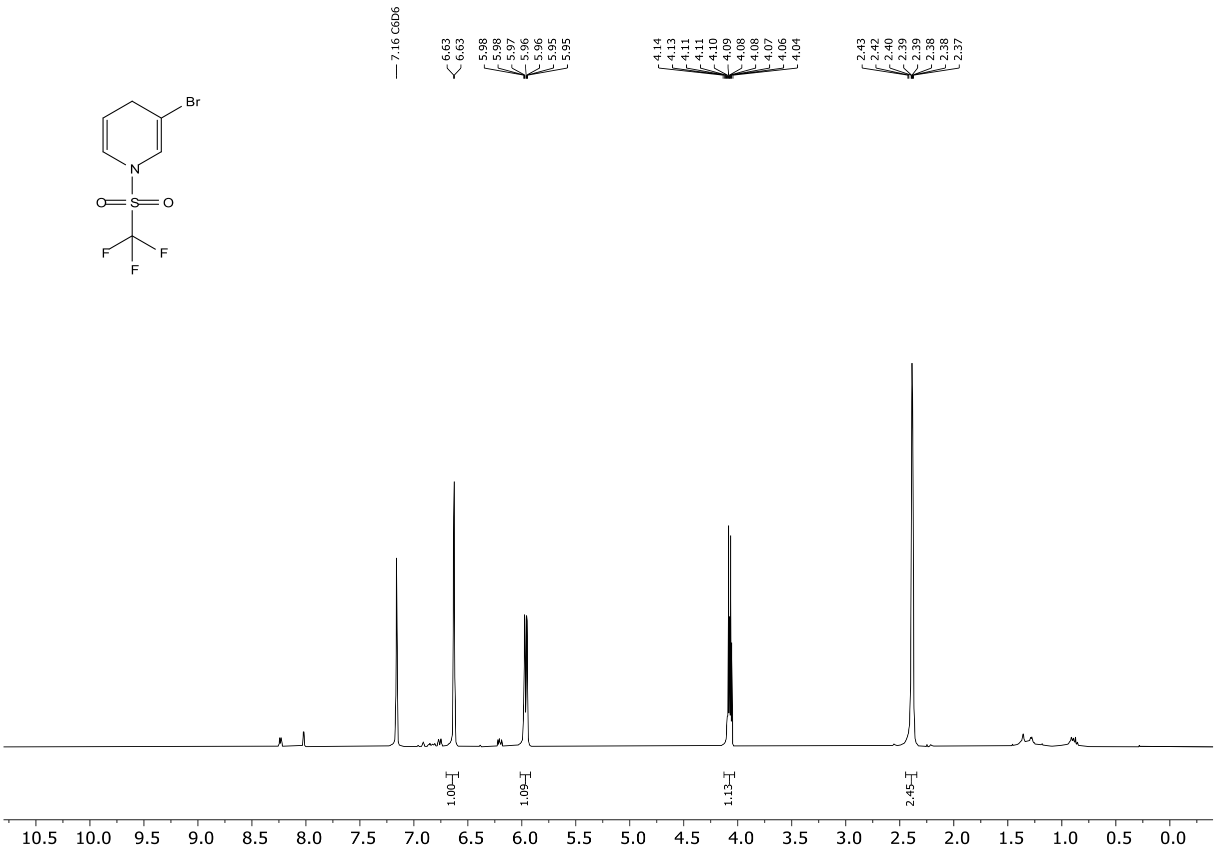

^{13}C{^{1}H} NMR (101 MHz, benzene-d_6) – compound **9**

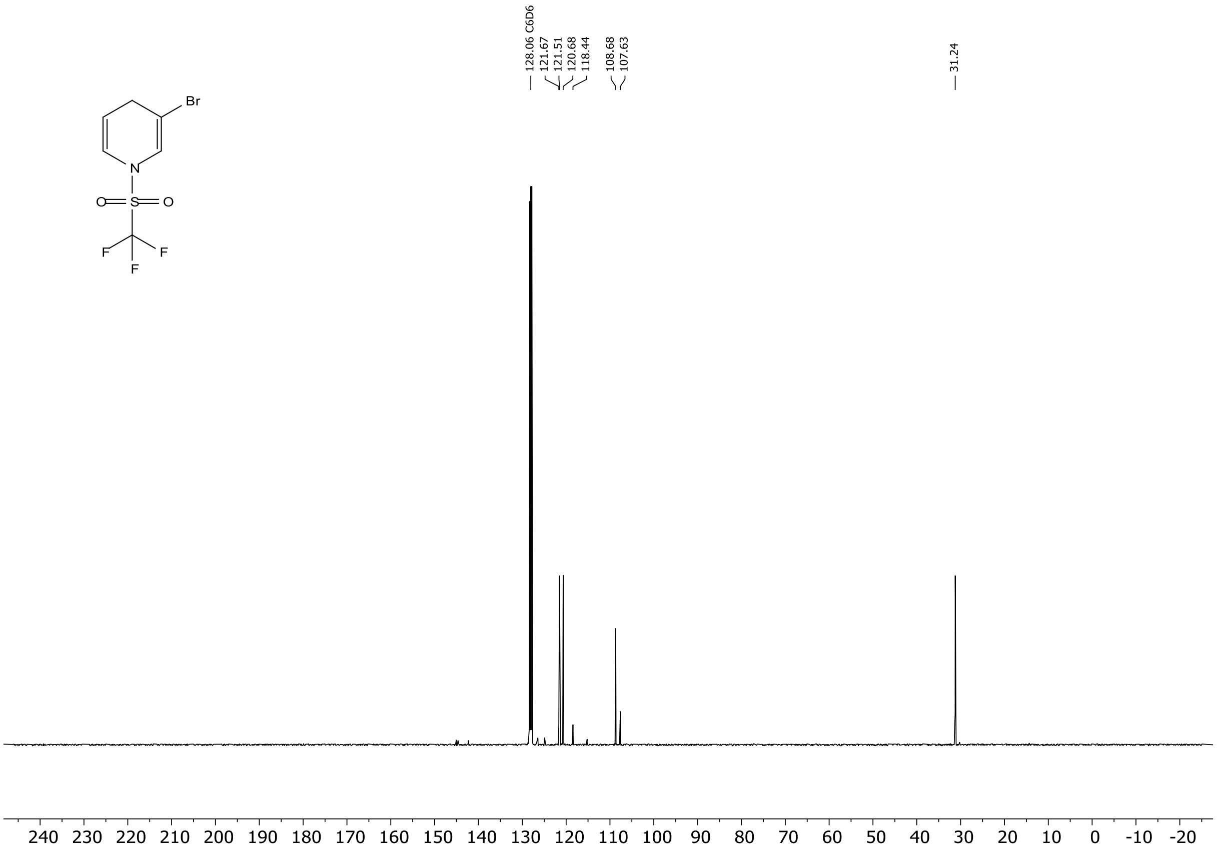

$^{19}F\{^{1}H\}$ NMR (377 MHz, benzene-d_6) – compound **9**

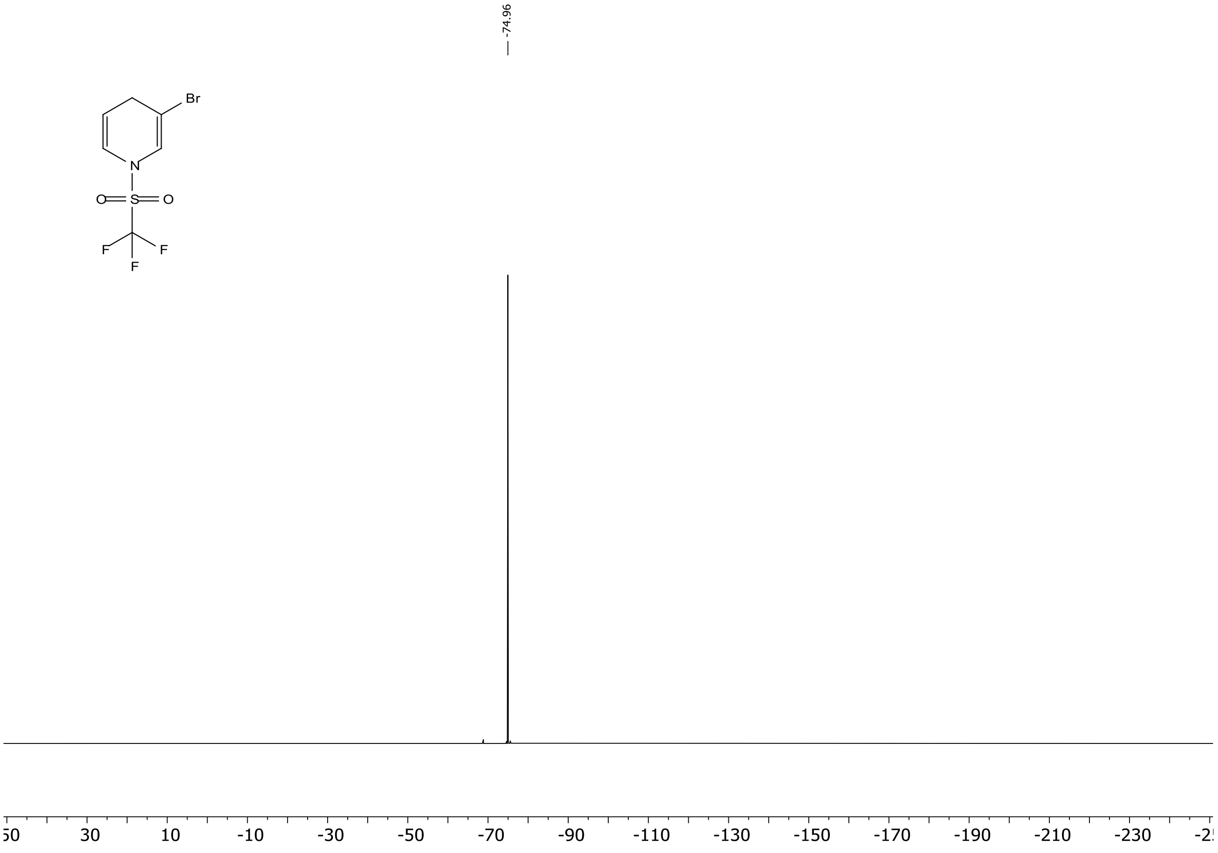

^{1}H NMR (400 MHz, benzene-d_6) – compound **10**

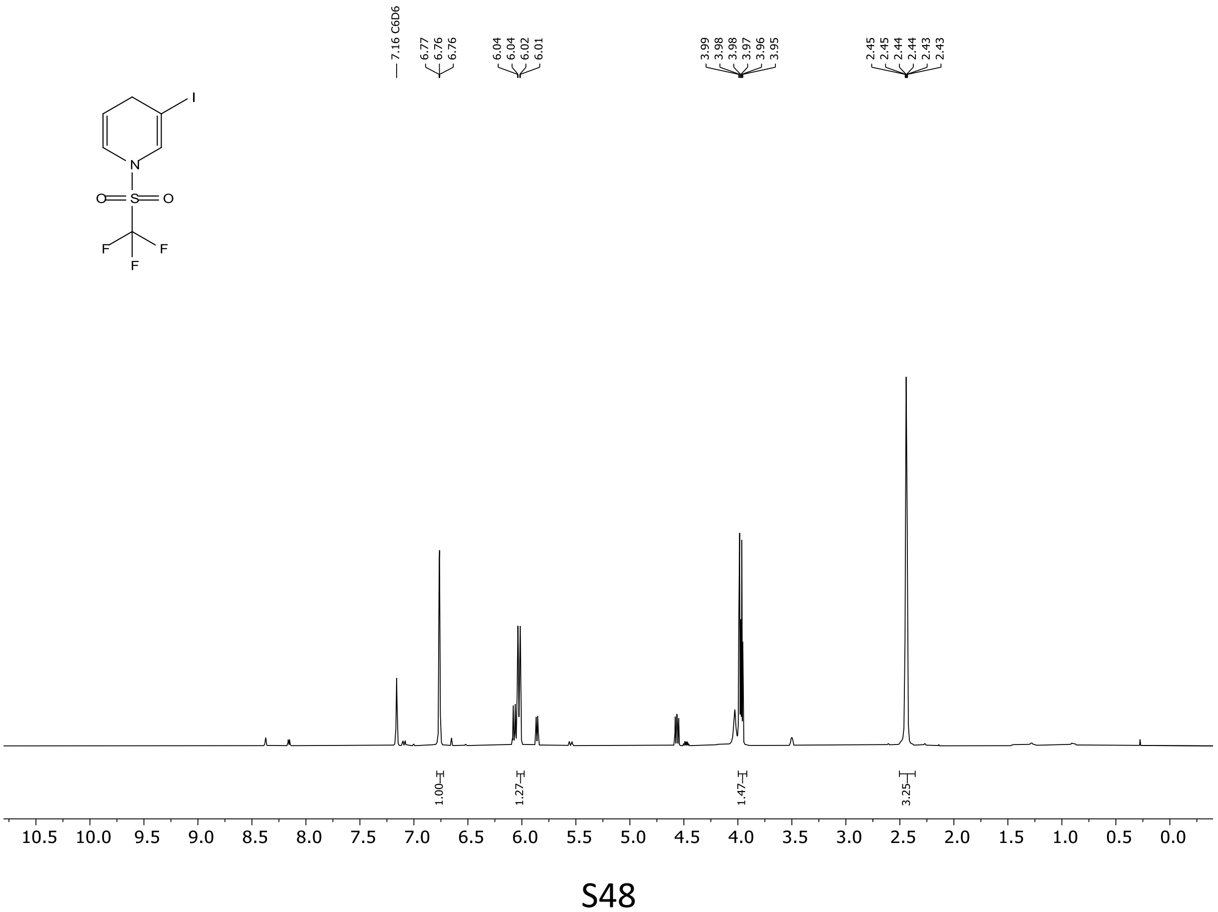

$^{13}C\{^{1}H\}$ NMR (101 MHz, benzene-d_6) – compound **10**

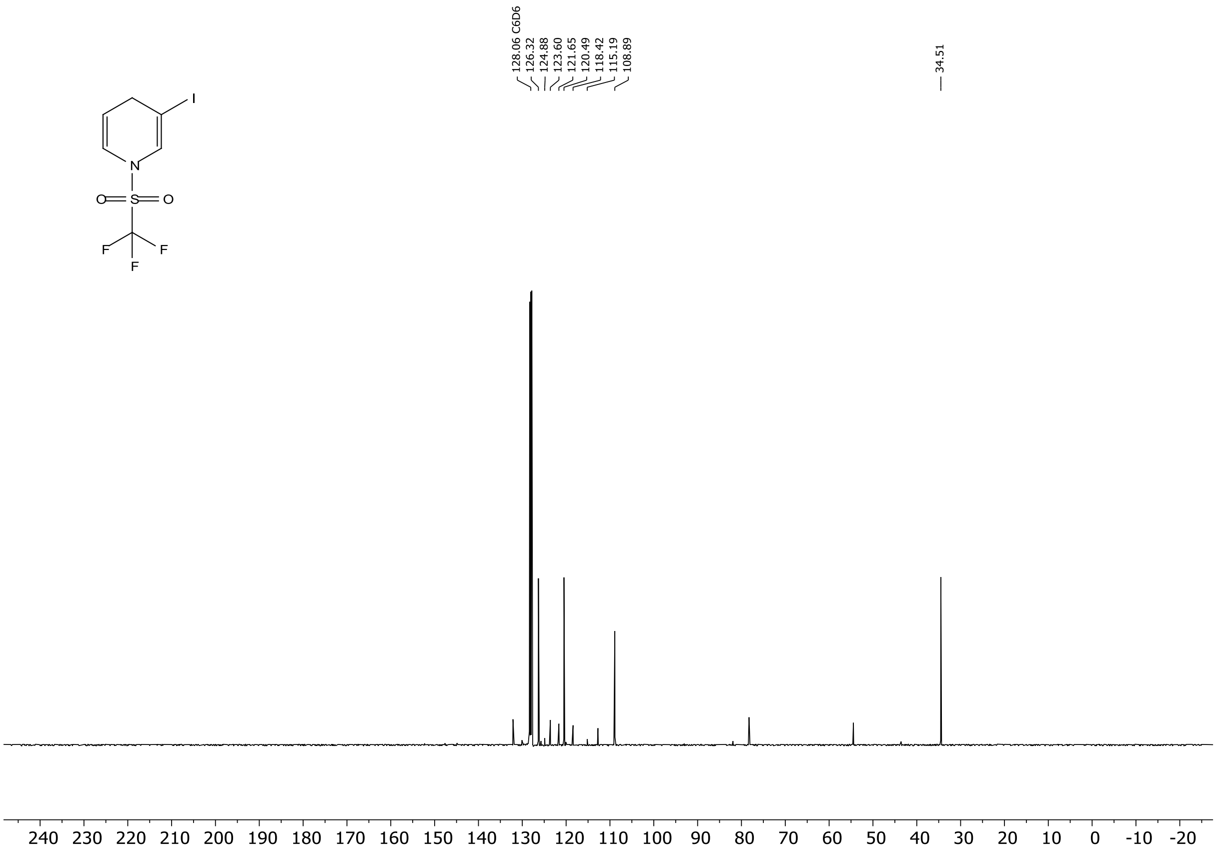

$^{19}F\{^{1}H\}$ NMR (377 MHz, benzene-d_6) – compound **10**

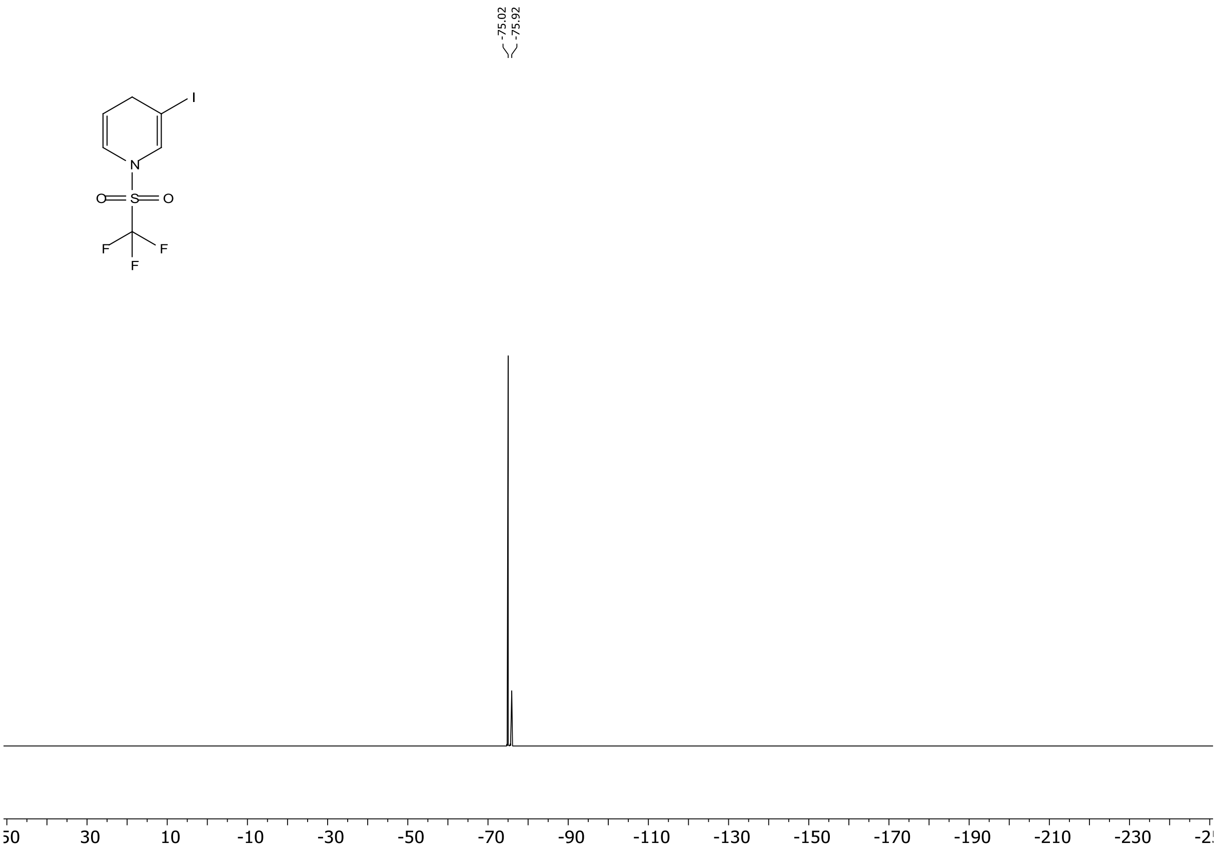

^{1}**H NMR** (400 MHz, benzene-d_6) – compound **11**

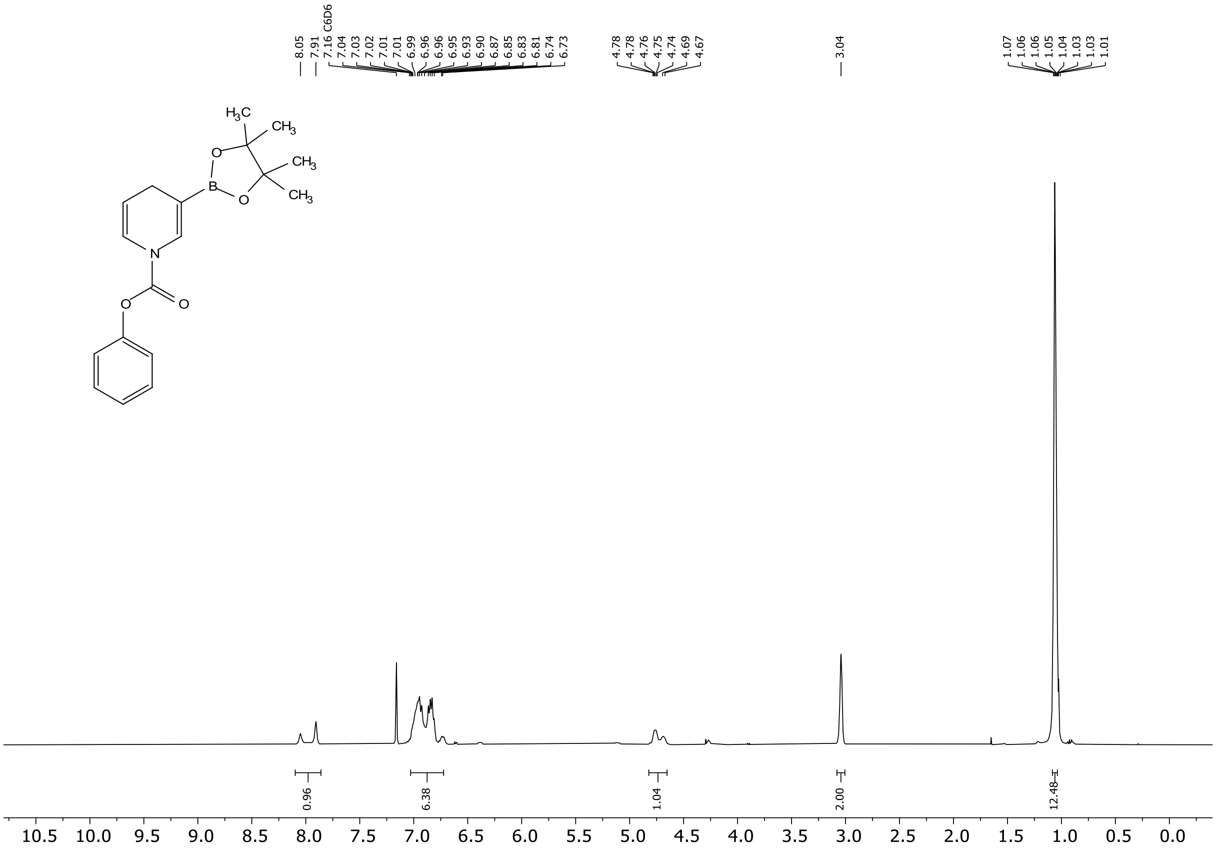

^{11}**B NMR** (128 MHz, benzene-d_6) – compound **11**

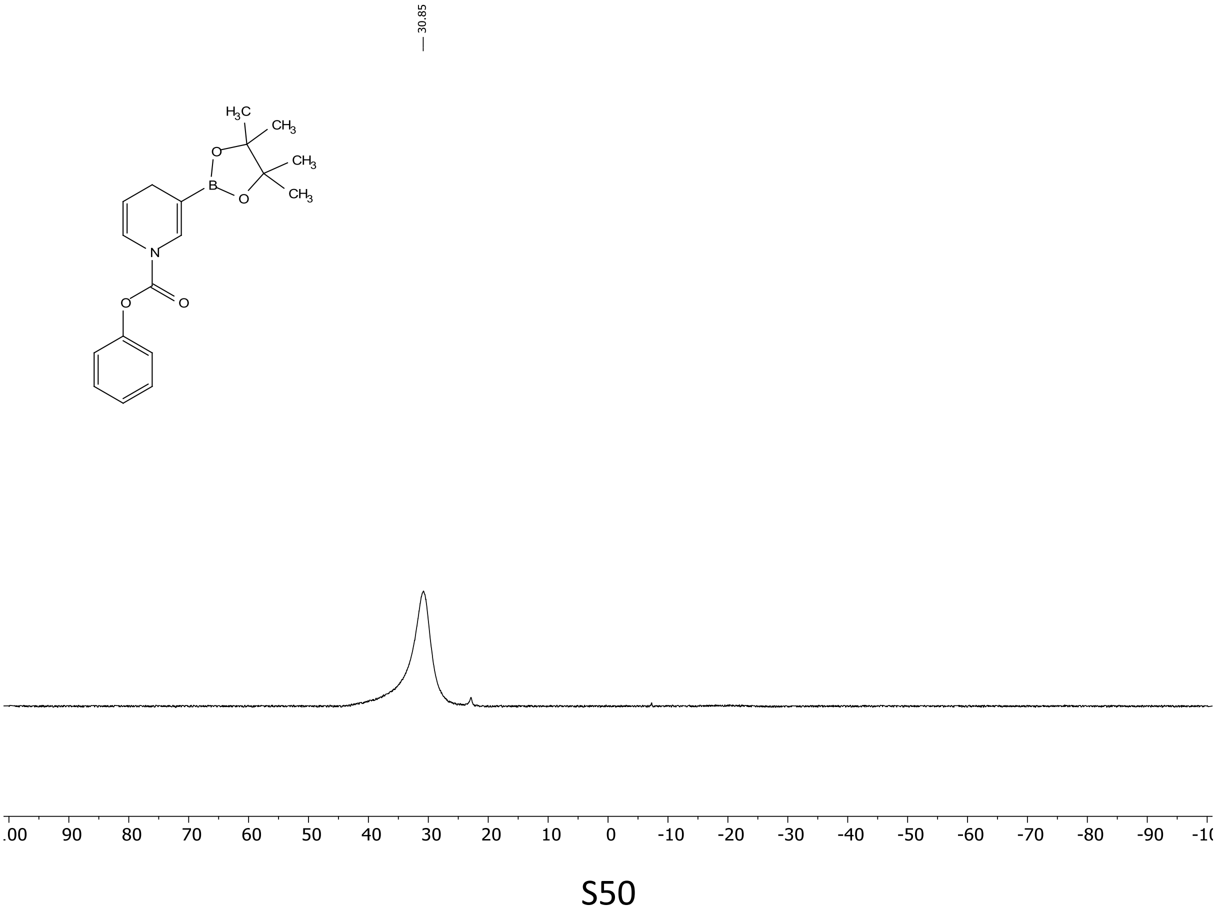

$^{13}C\{^1H\}$ NMR (101 MHz, benzene-d_6) – compound **11**

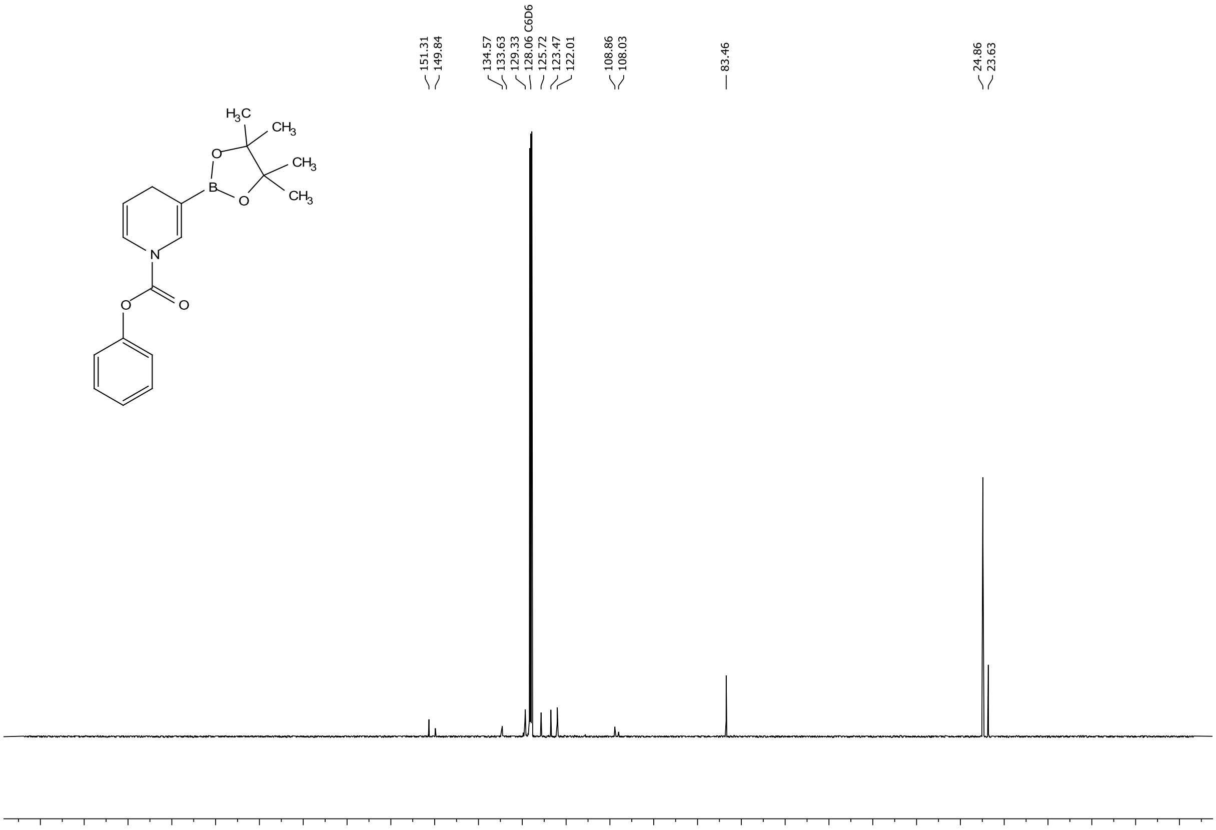

^1H NMR (400 MHz, benzene-d_6) – compound **12**

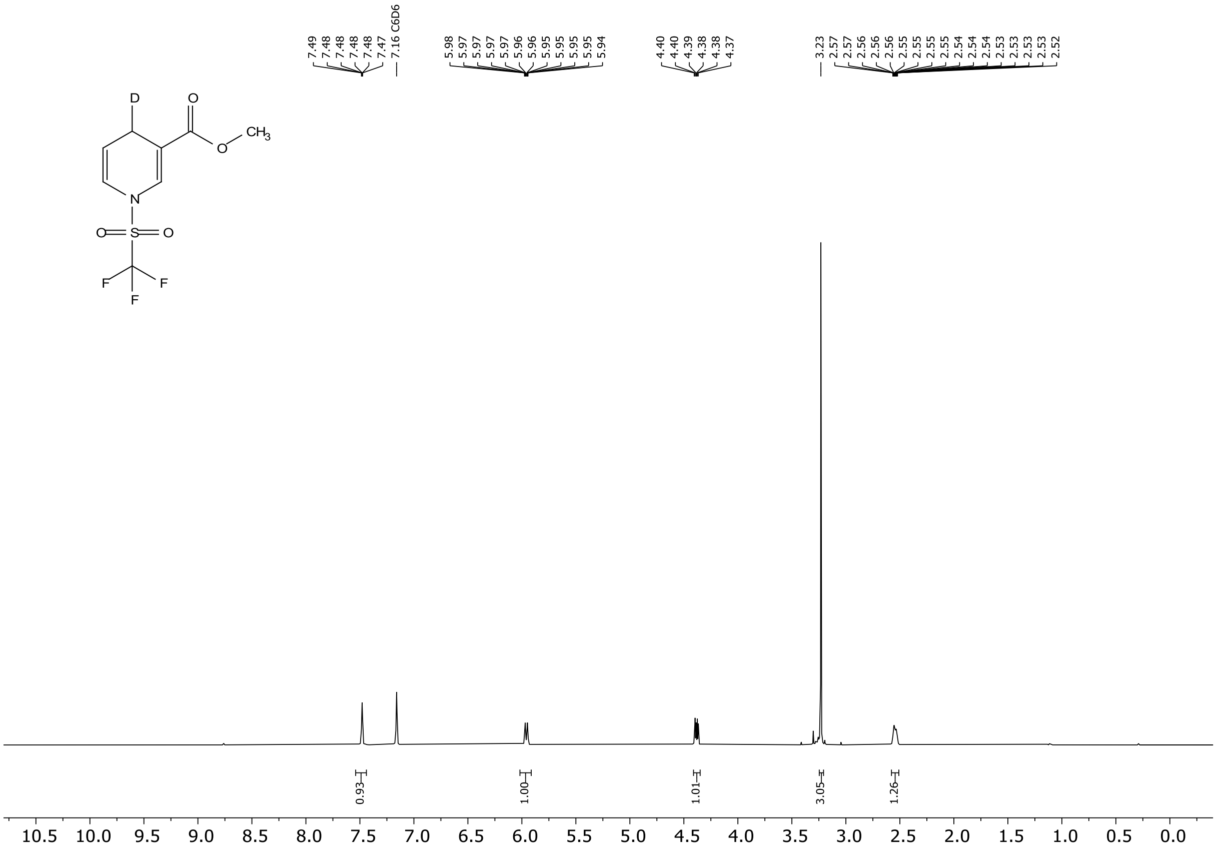

$^{13}C\{^{1}H\}$ NMR (101 MHz, benzene-d_6) – compound **12**

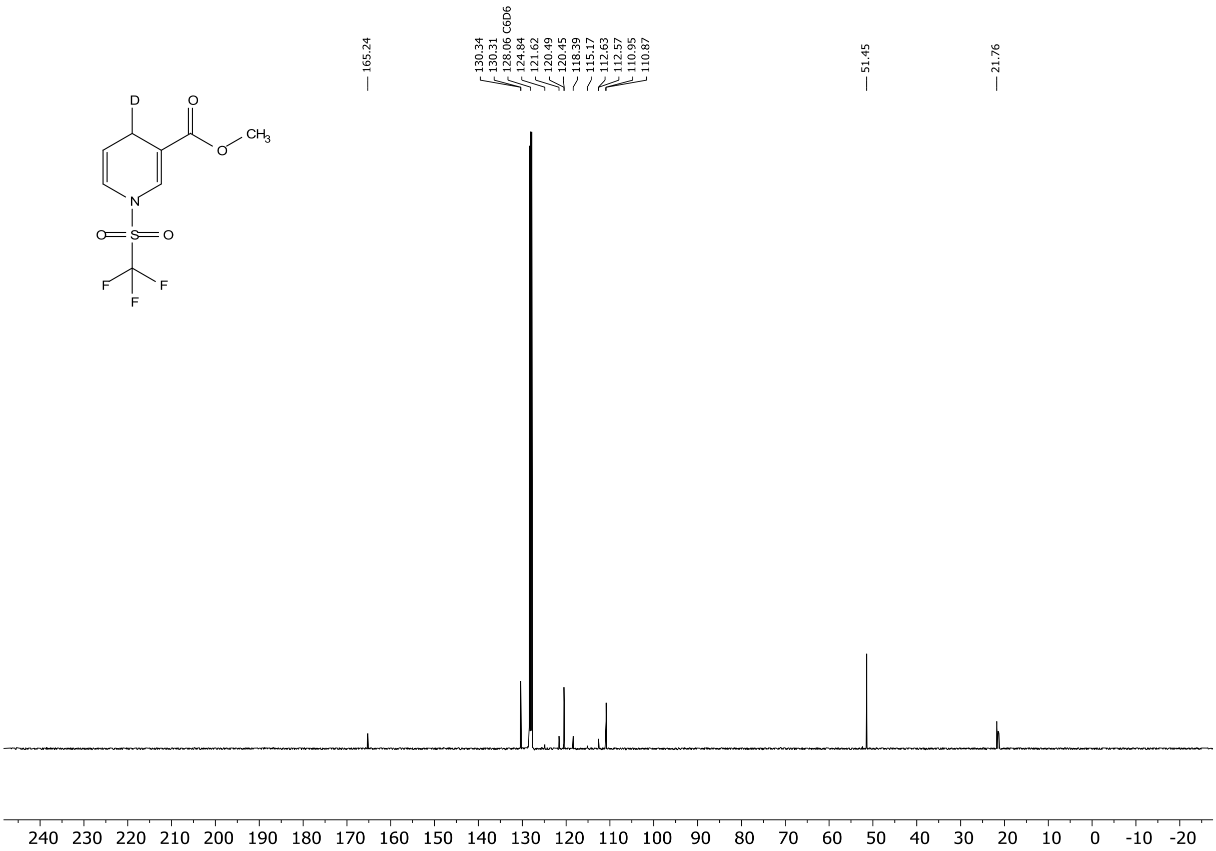

$^{19}F\{^{1}H\}$ NMR (377 MHz, benzene-d_6) – compound **12**

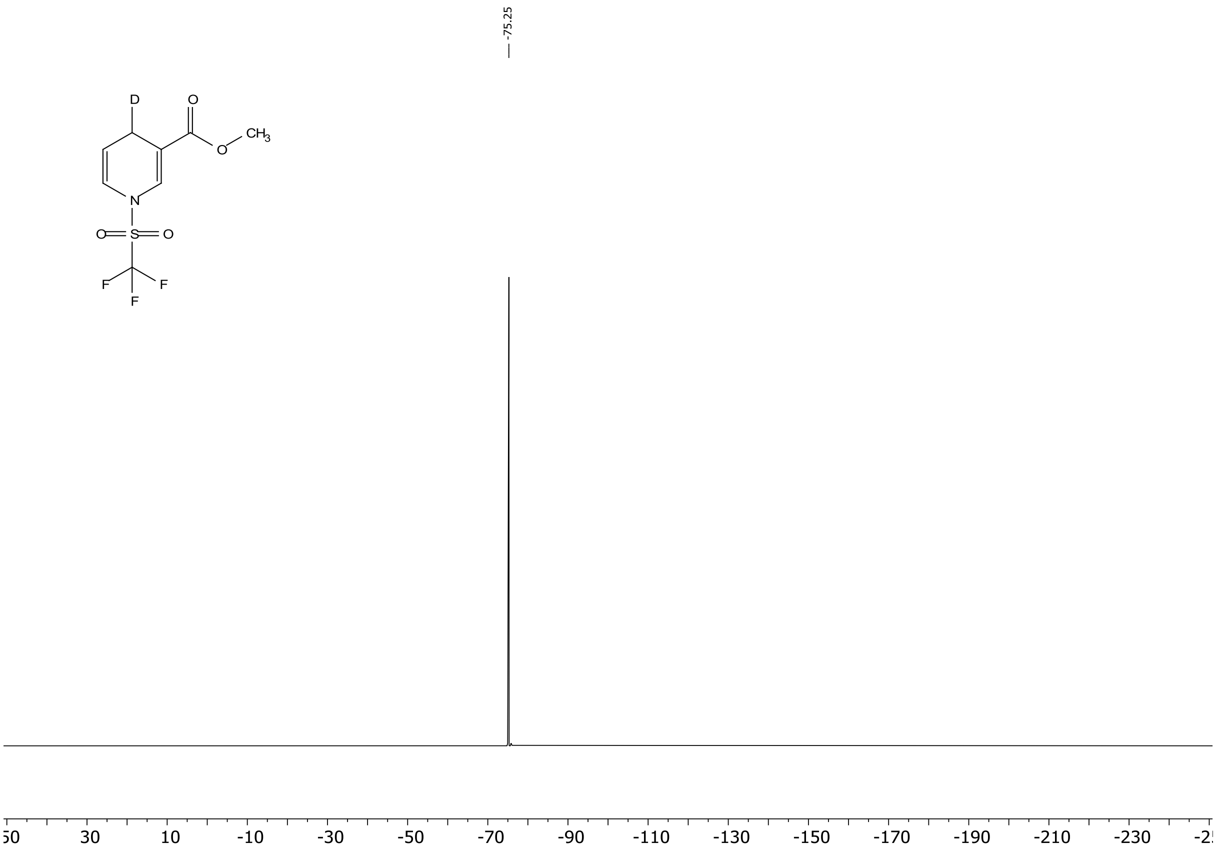

^{1}**H NMR** (400 MHz, benzene-d_6) – compound **13**

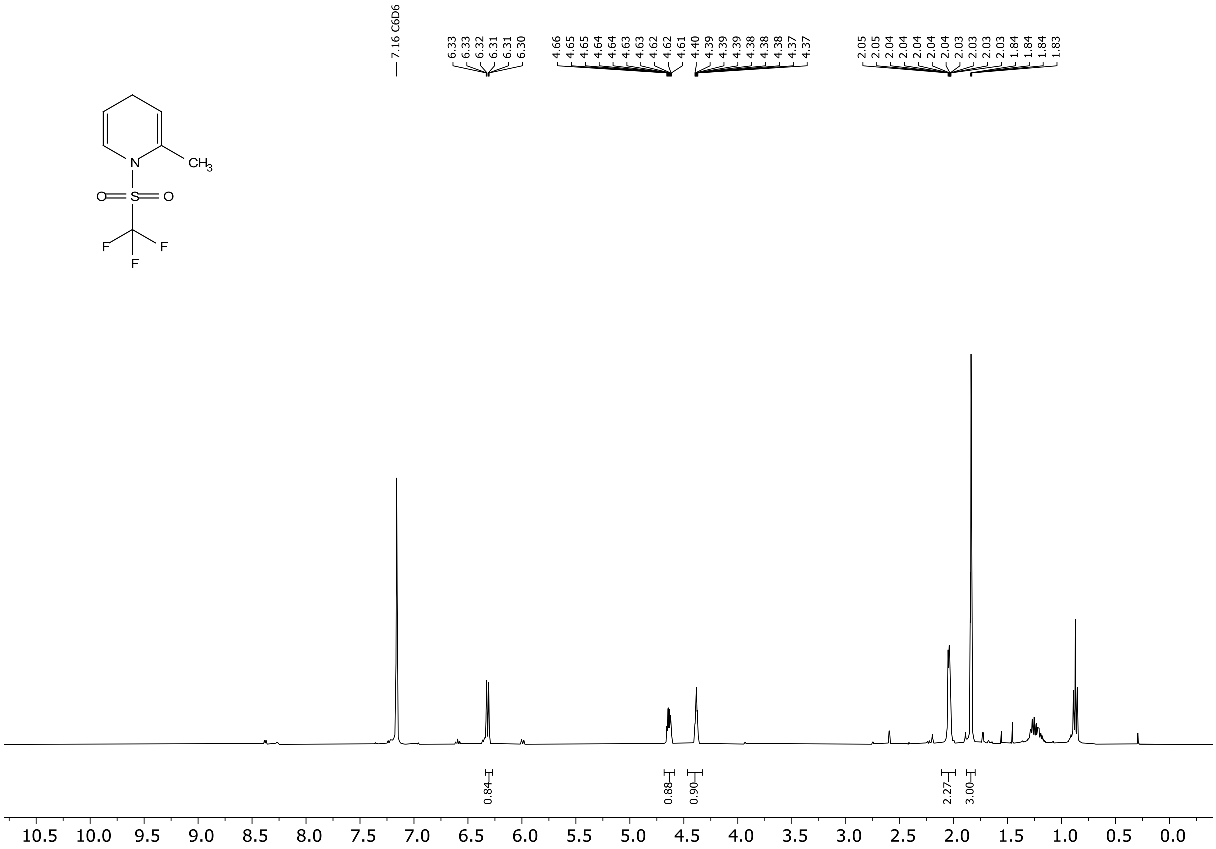

^{13}**C{^{1}H} NMR** (101 MHz, benzene-d_6) – compound **13**

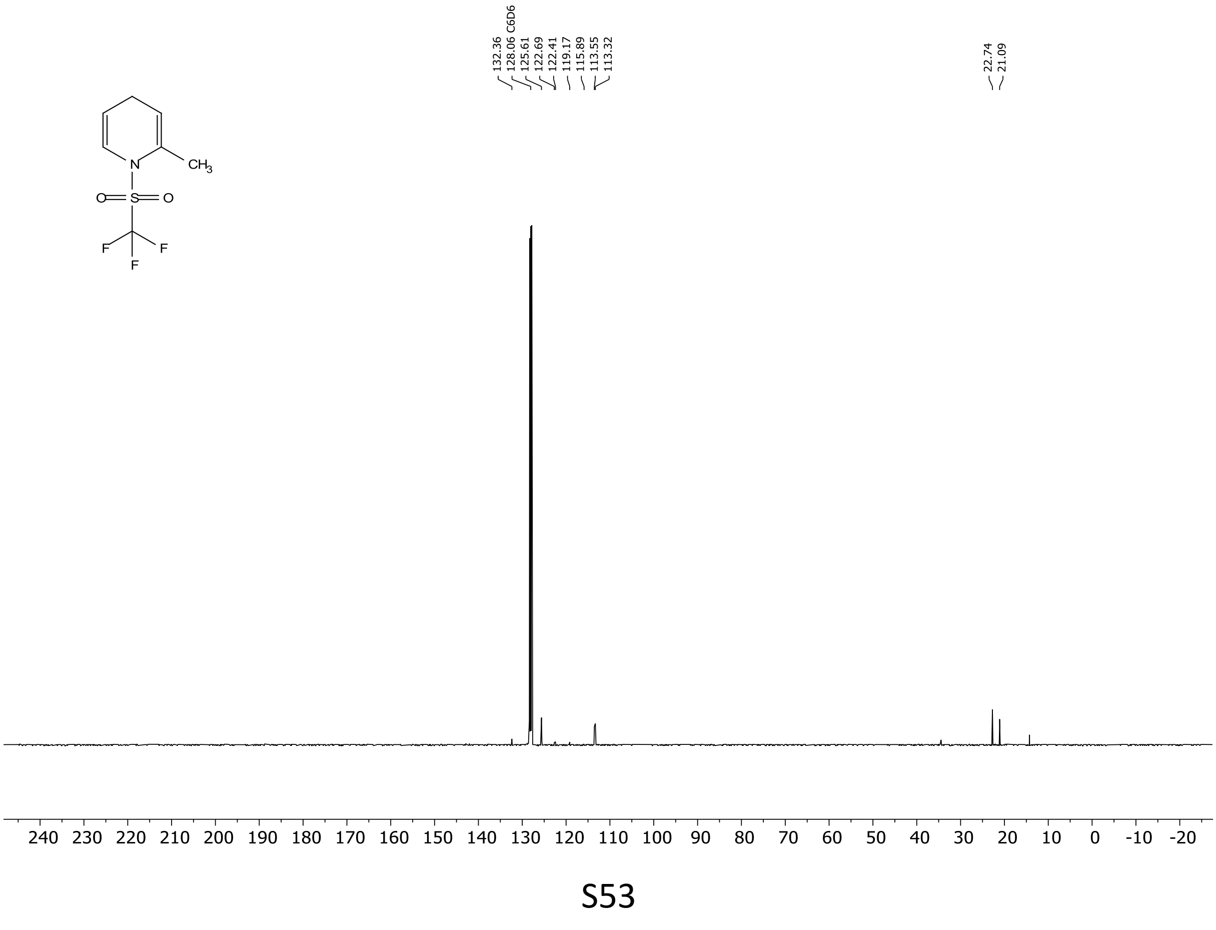

$^{19}F\{^1H\}$ NMR (377 MHz, benzene-d_6) – compound **13**

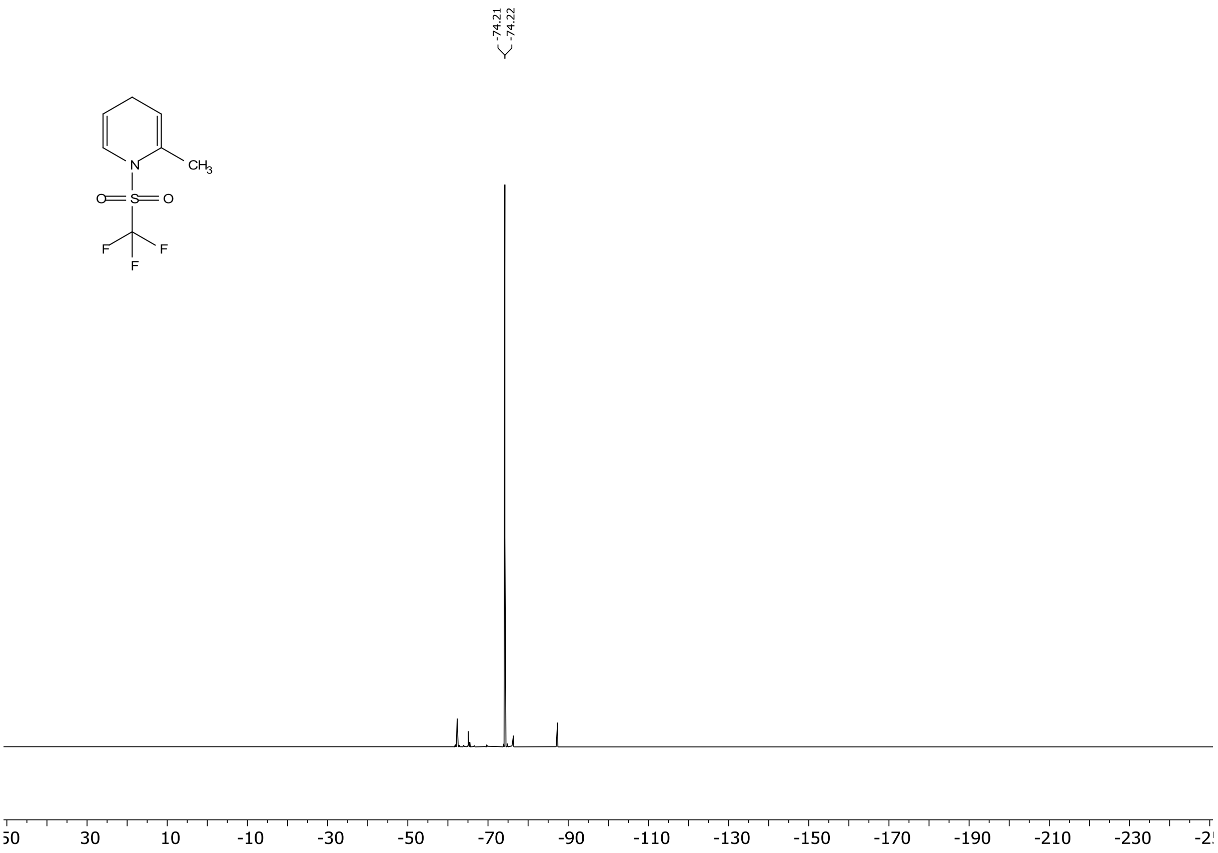

^1H NMR (400 MHz, benzene-d_6) – compound **14**

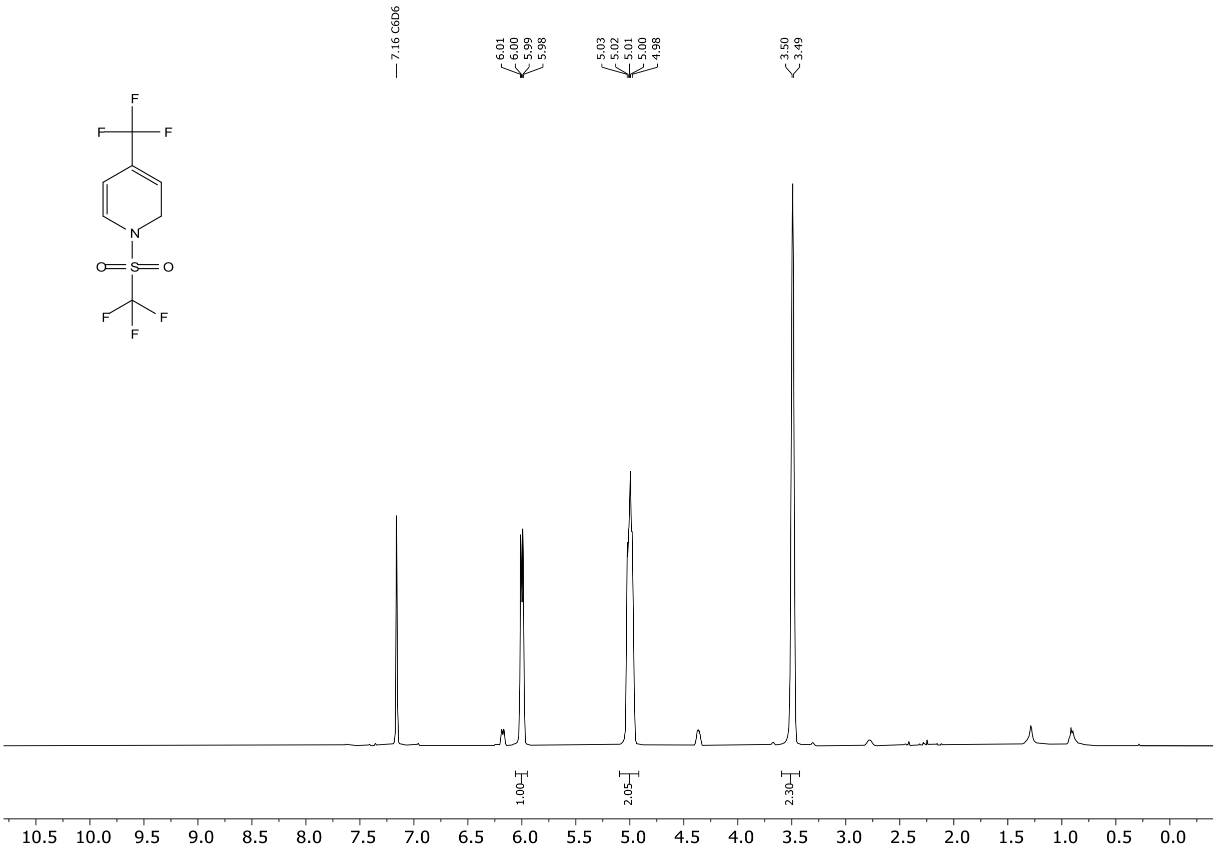

$^{13}C\{^1H\}$ NMR (101 MHz, benzene-d_6) – compound **14**

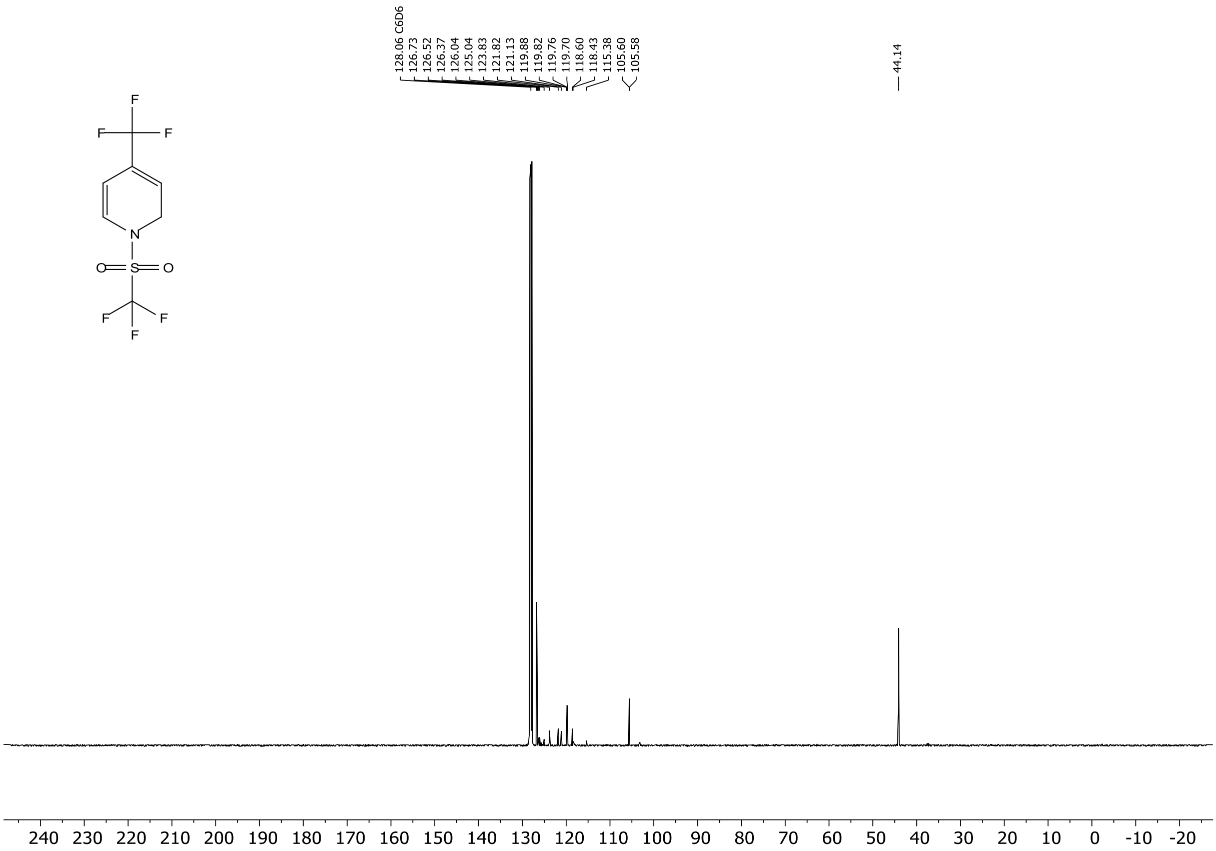

$^{19}F\{^1H\}$ NMR (377 MHz, benzene-d_6) – compound **14**

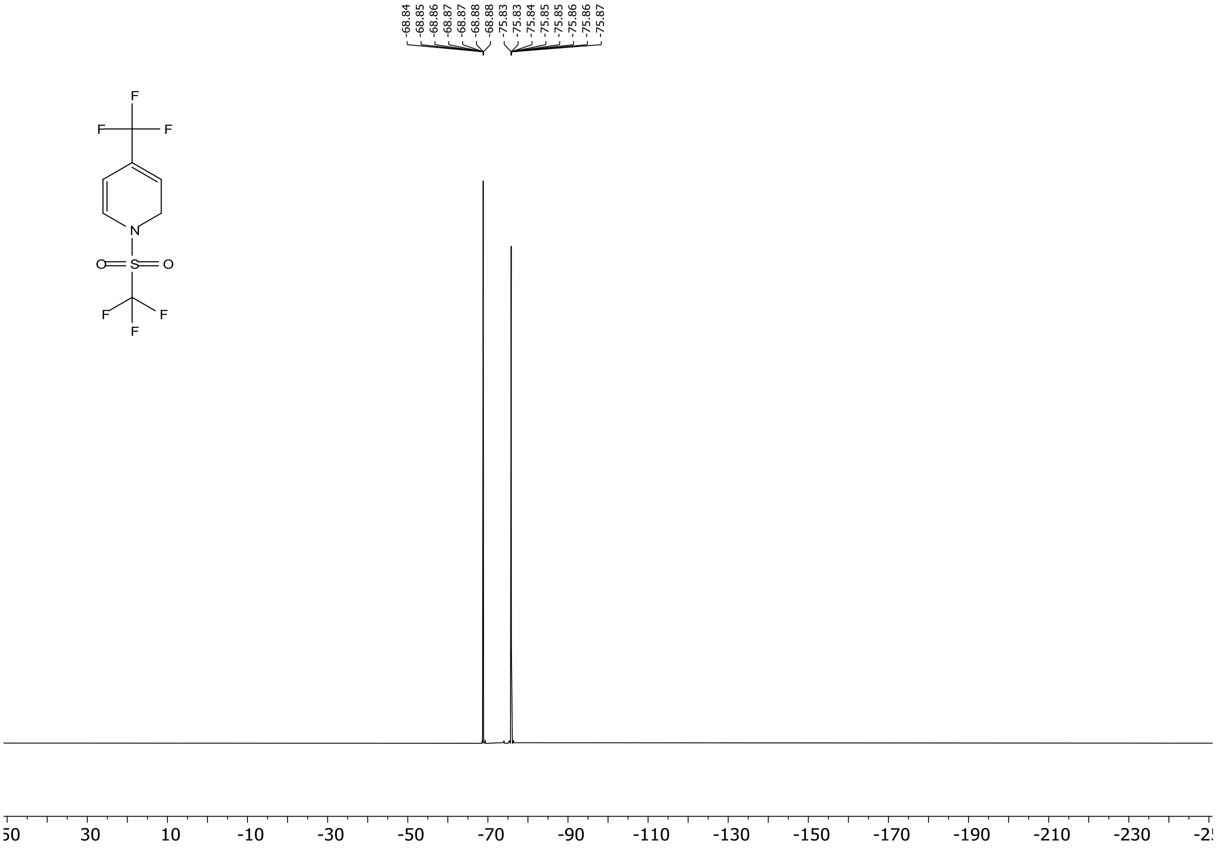

^{1}H NMR (400 MHz, benzene-d_6) – compound **15**

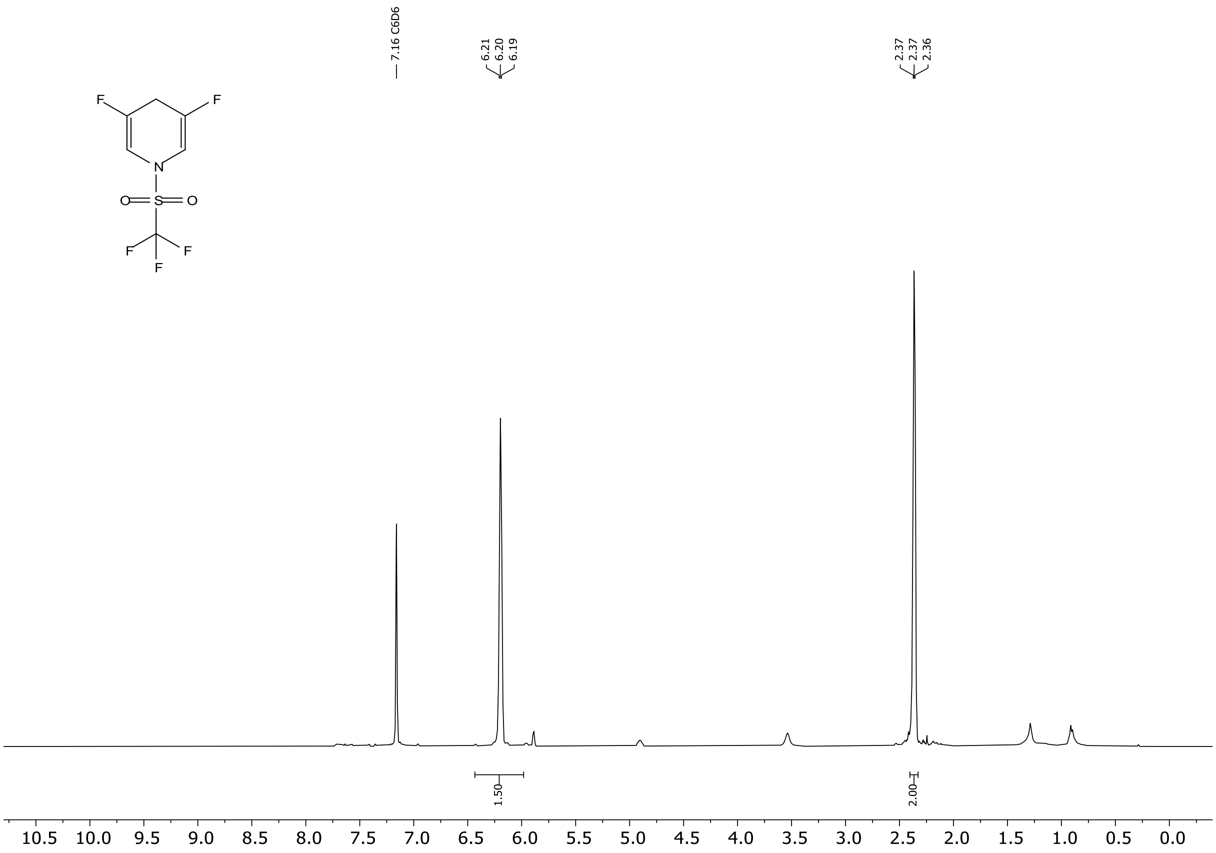

^{13}C{^{1}H} NMR (101 MHz, benzene-d_6) – compound **15**

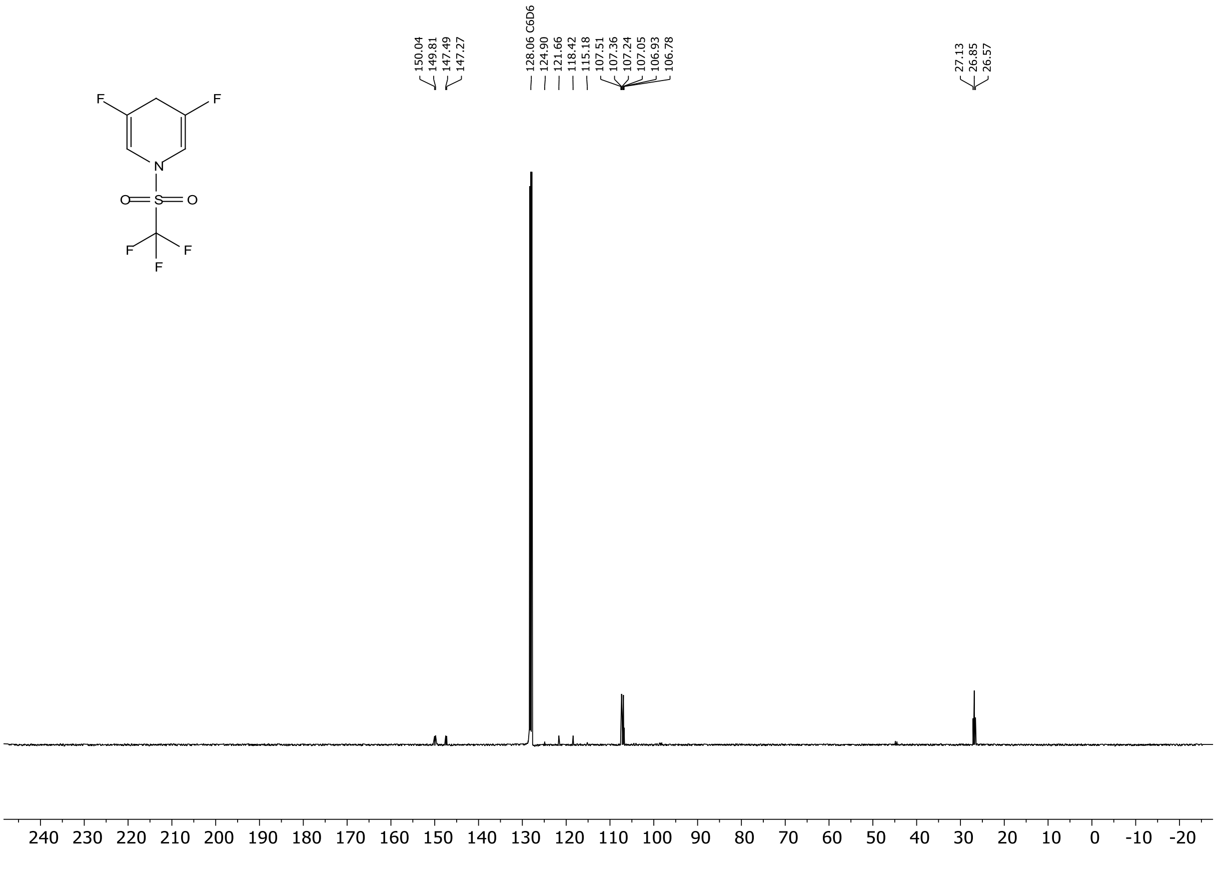

^{19}F{^{1}H} NMR (377 MHz, benzene-d_6) – compound **15**

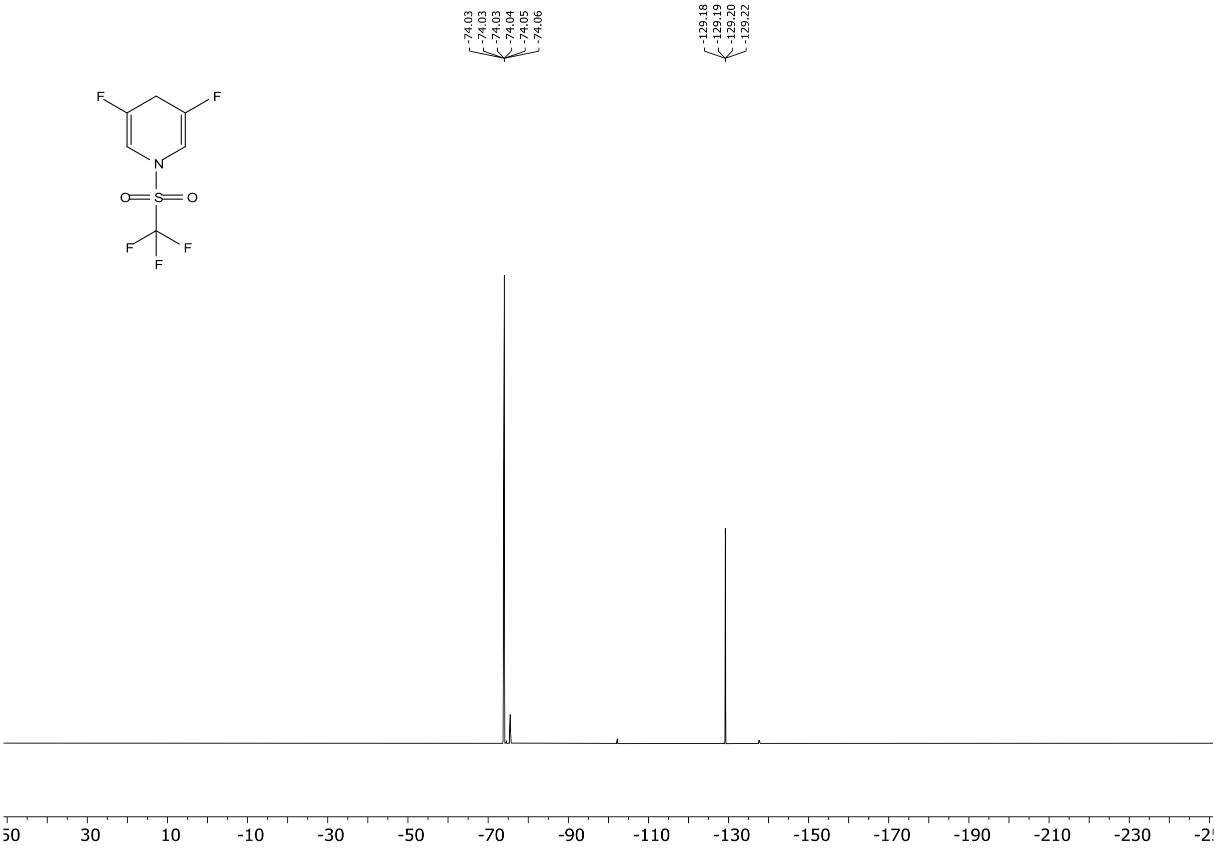

^{1}H NMR (400 MHz, benzene-d_6) – compound **16**

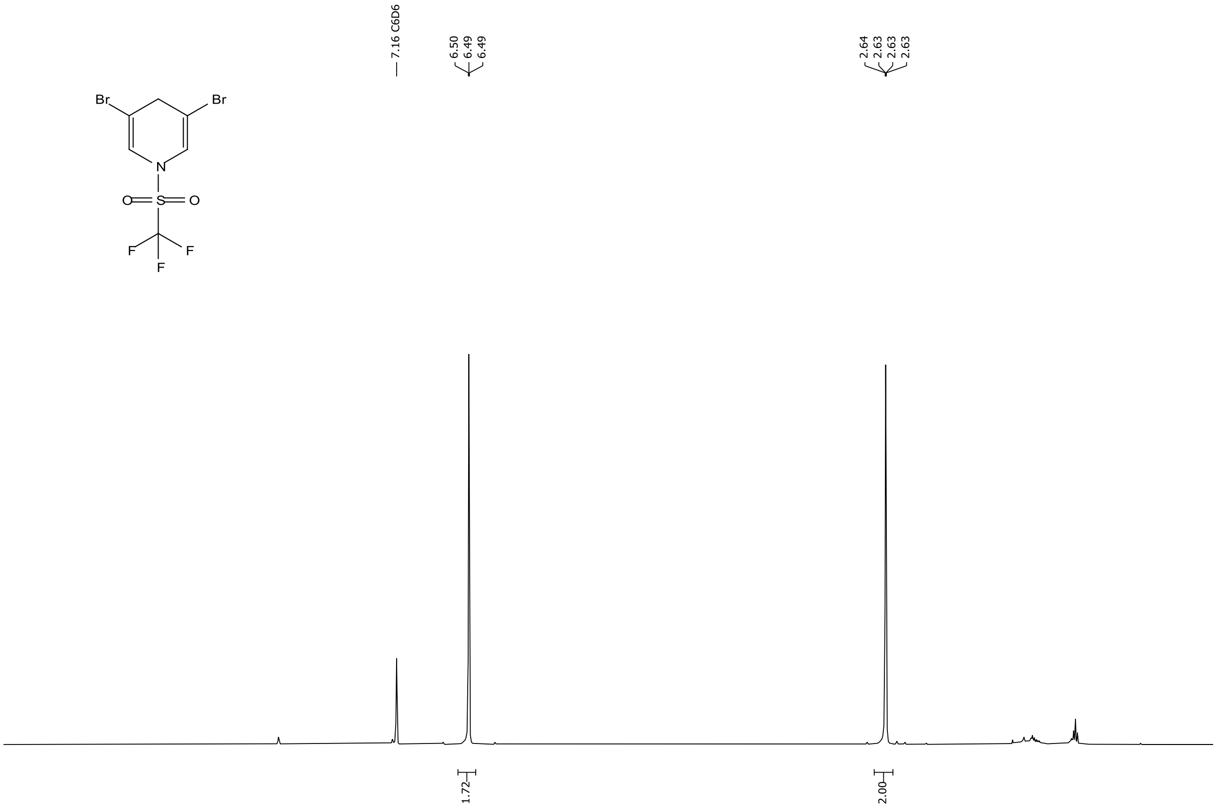

^{13}C{^{1}H} NMR (101 MHz, benzene-d_6) – compound **16**

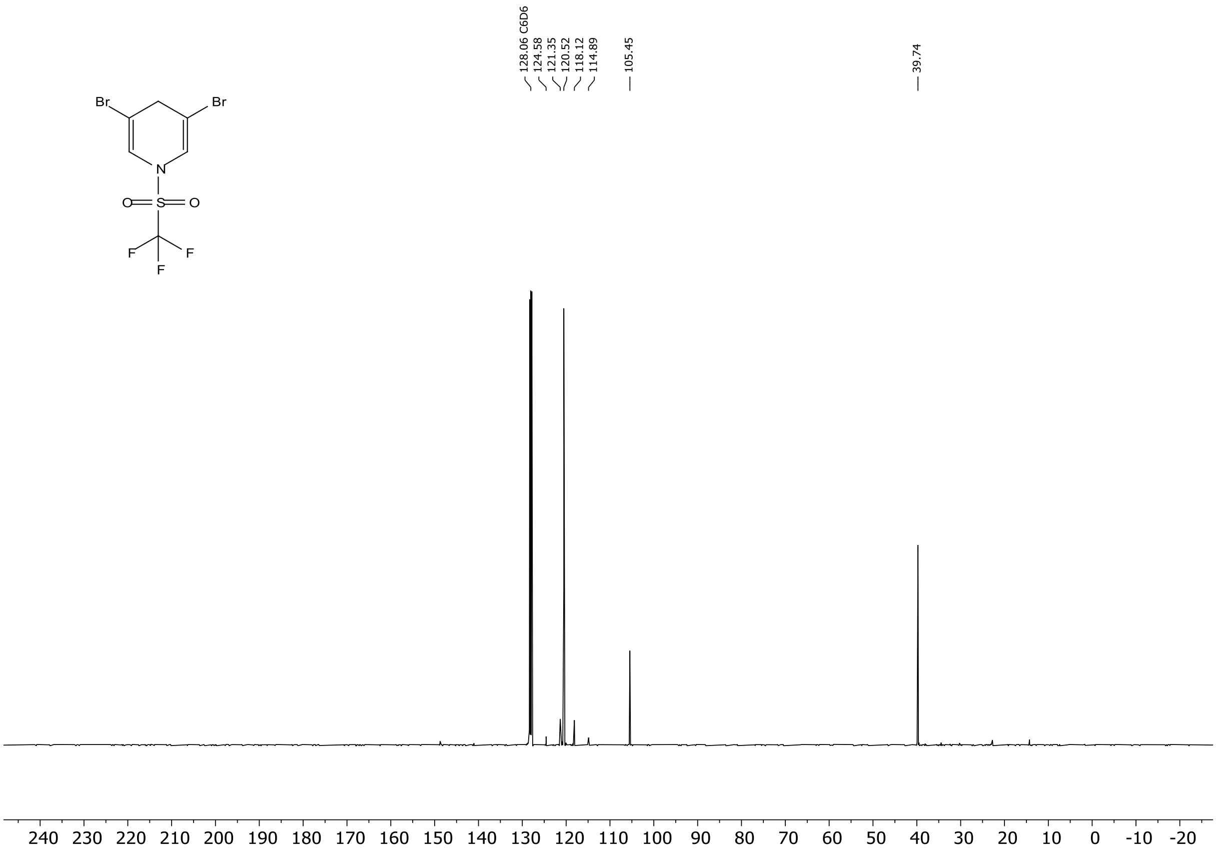

^{19}F{^{1}H} NMR (377 MHz, benzene-d_6) – compound **16**

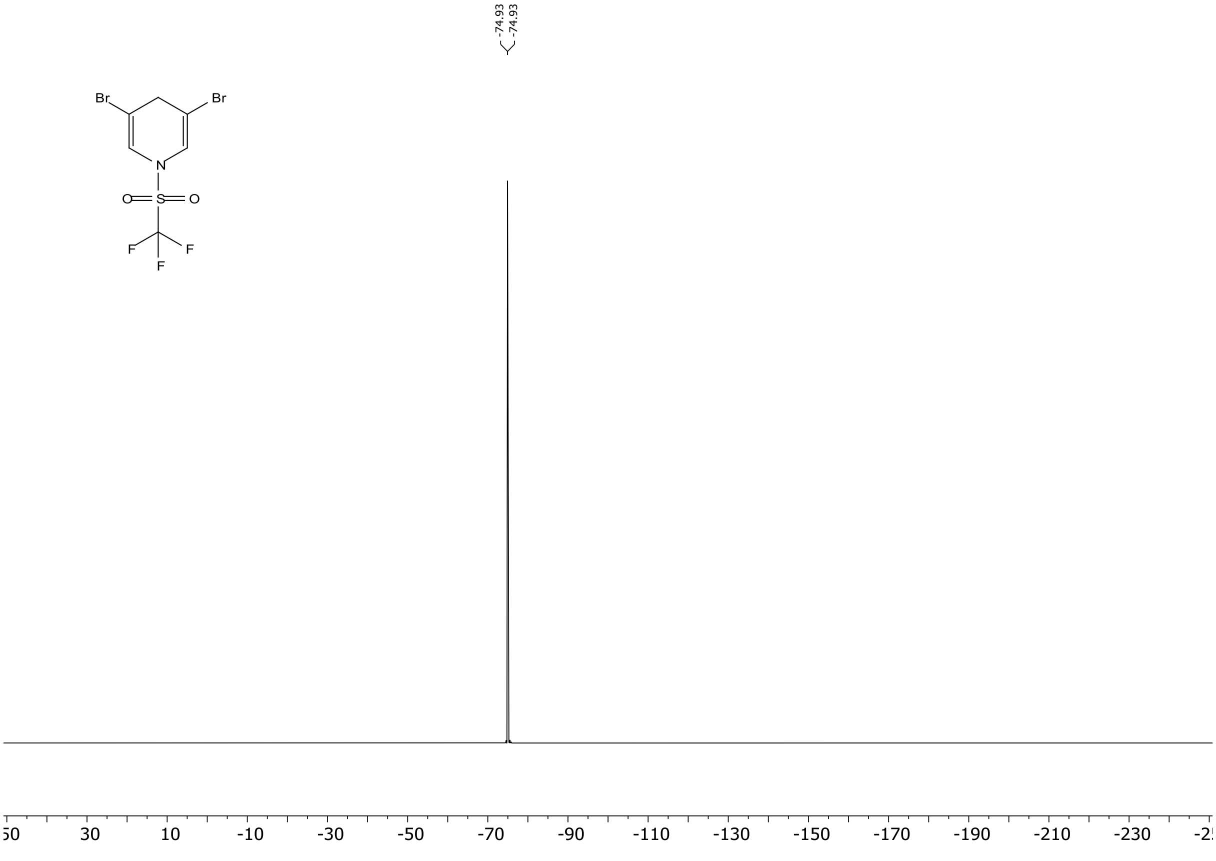

^{1}H NMR (400 MHz, benzene-d_6) – compound **17**

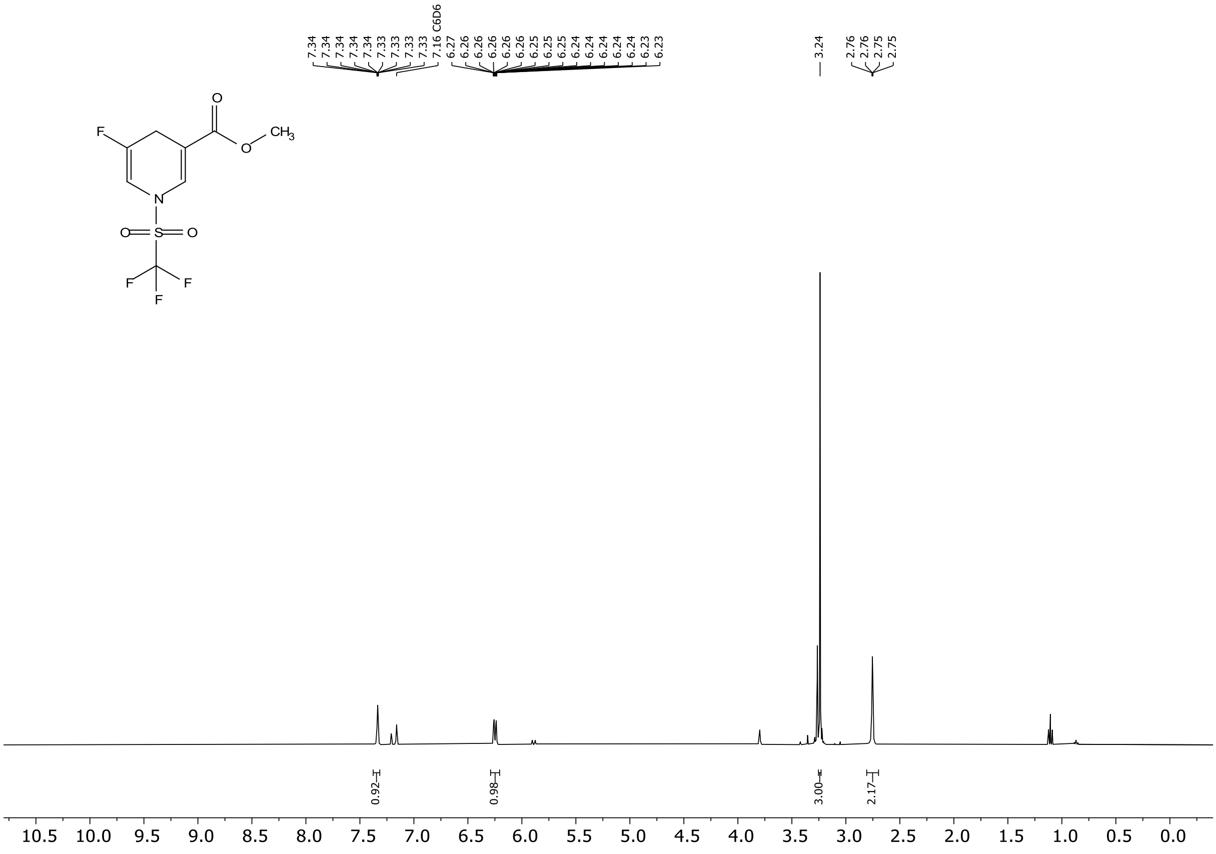

$^{13}C\{^{1}H\}$ NMR (101 MHz, benzene-d_6) – compound **17**

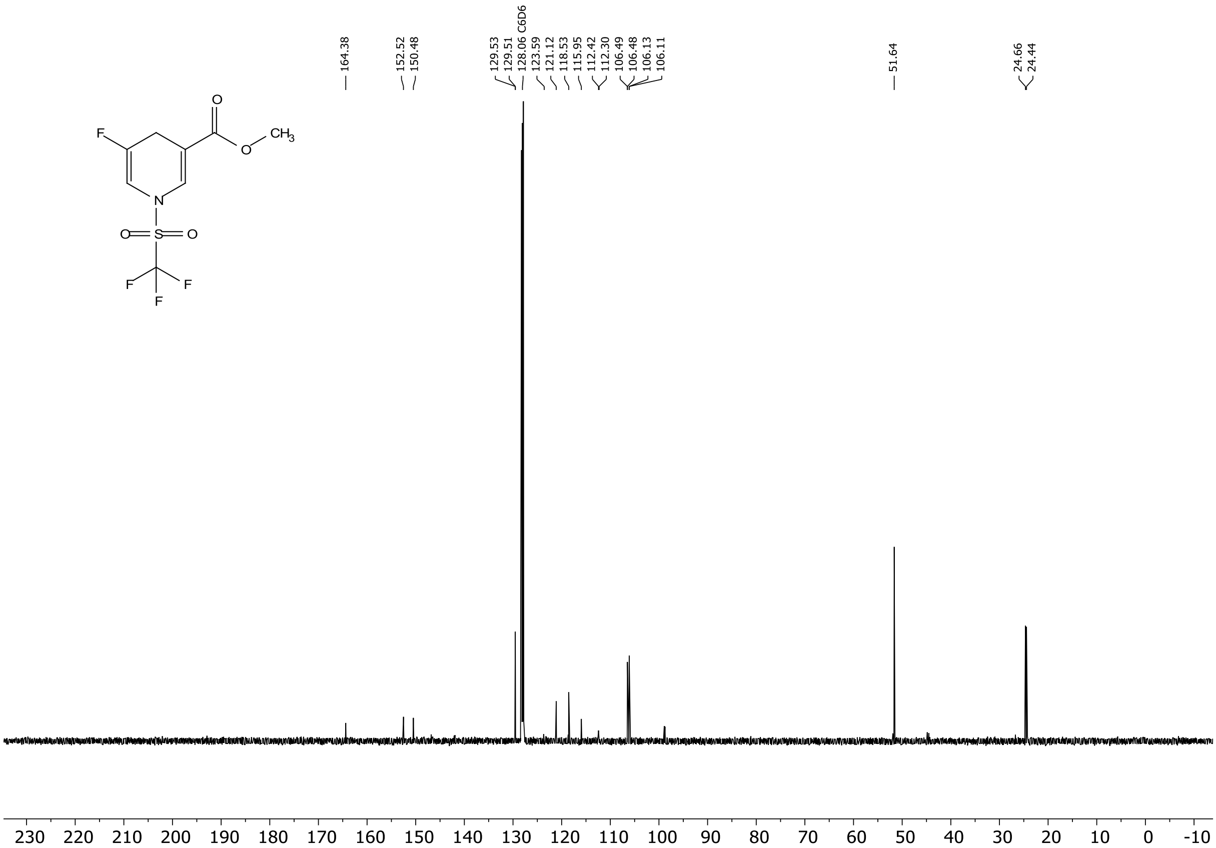

$^{19}F\{^{1}H\}$ NMR (377 MHz, benzene-d_6) – compound **17**

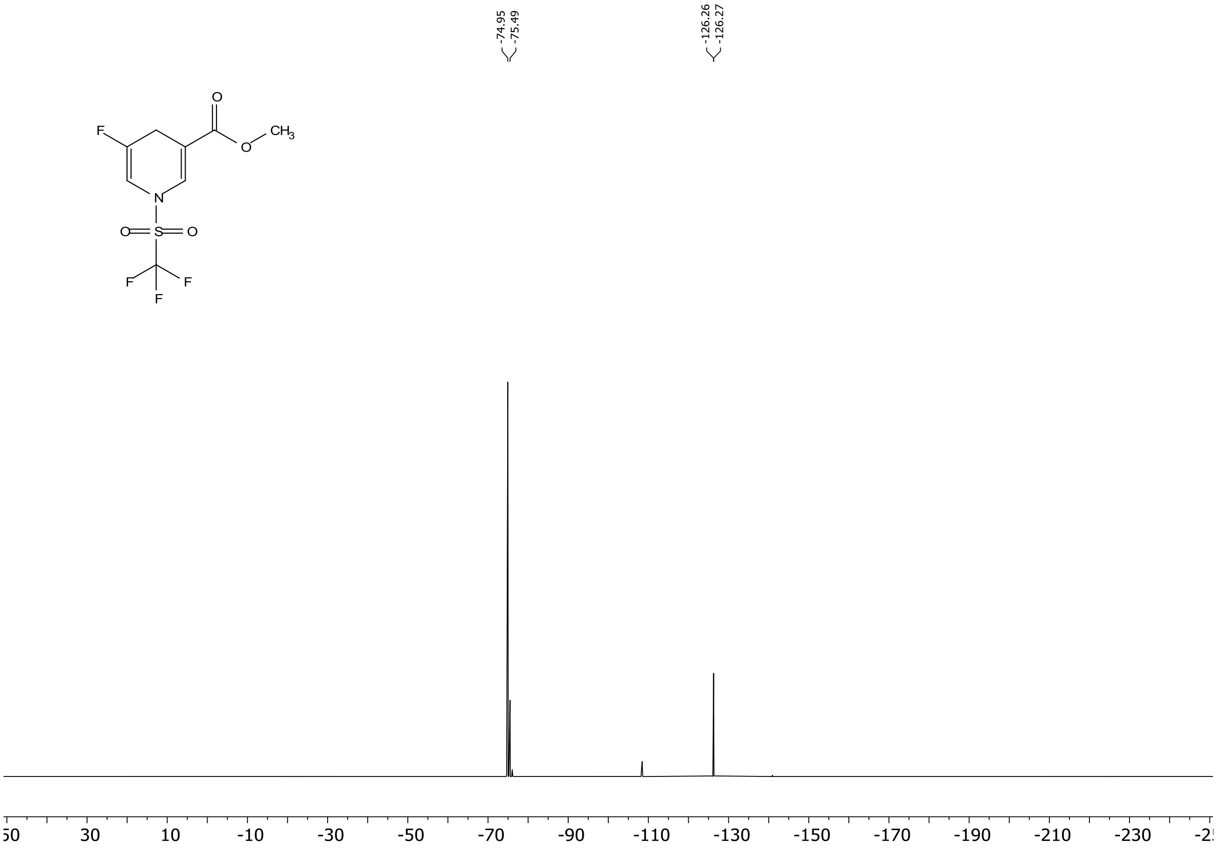

^{1}H NMR (400 MHz, benzene-d_6) – compound **18**

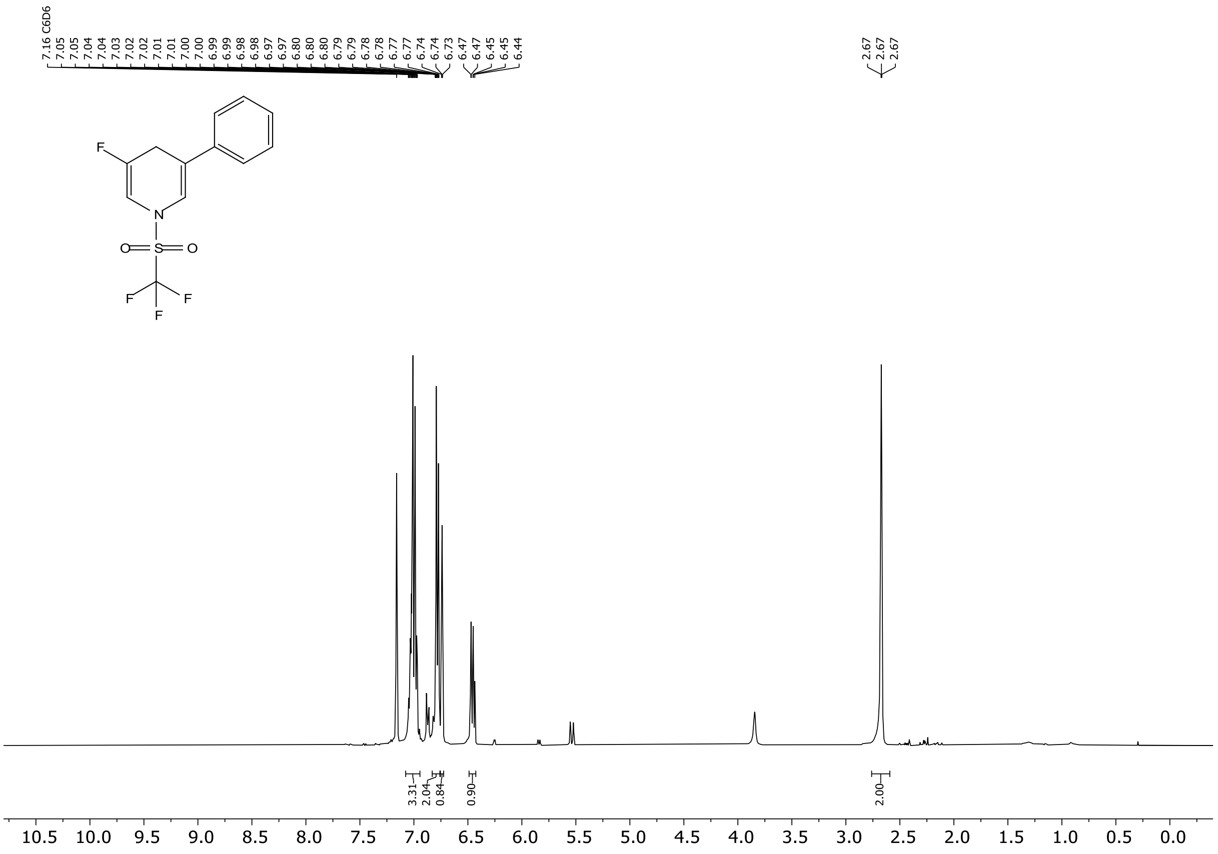

$^{13}C\{^1H\}$ NMR (101 MHz, benzene-d_6) – compound **18**

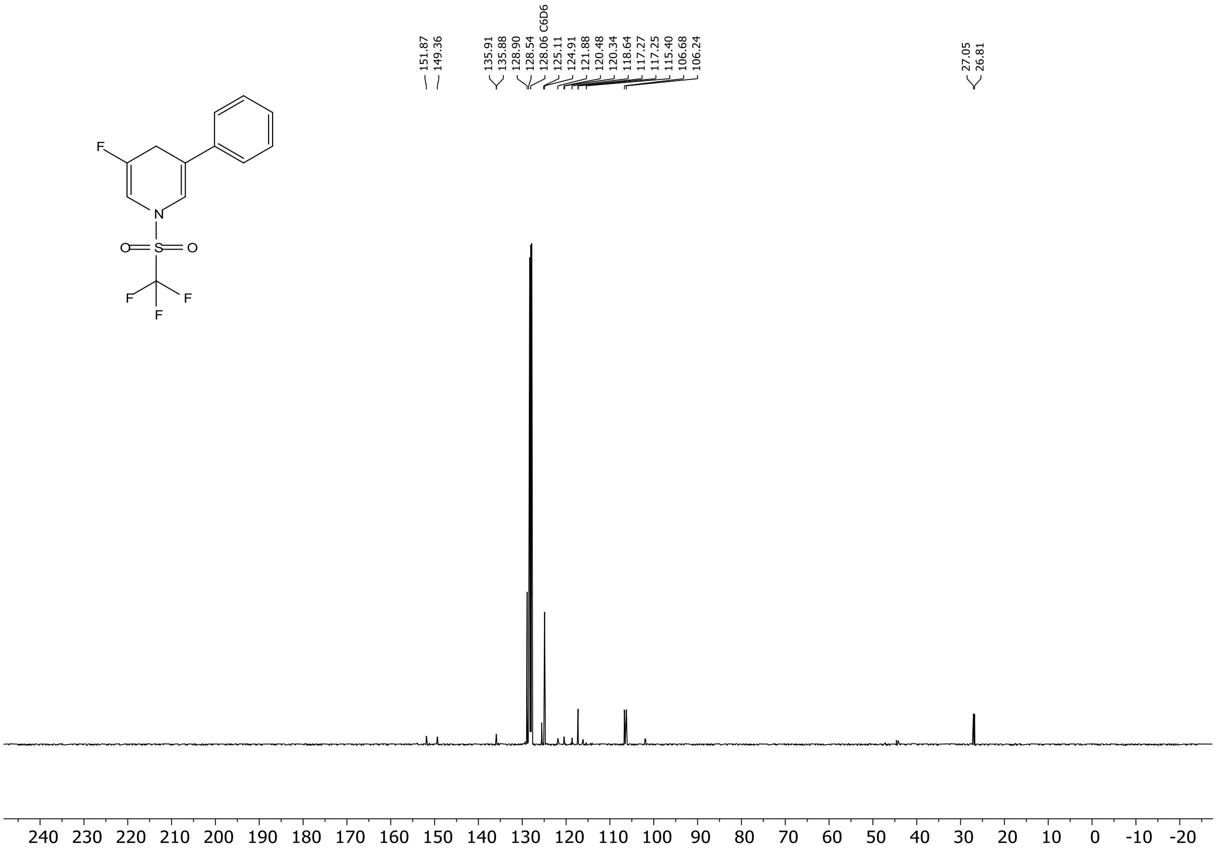

$^{19}F\{^1H\}$ NMR (377 MHz, benzene-d_6) – compound **18**

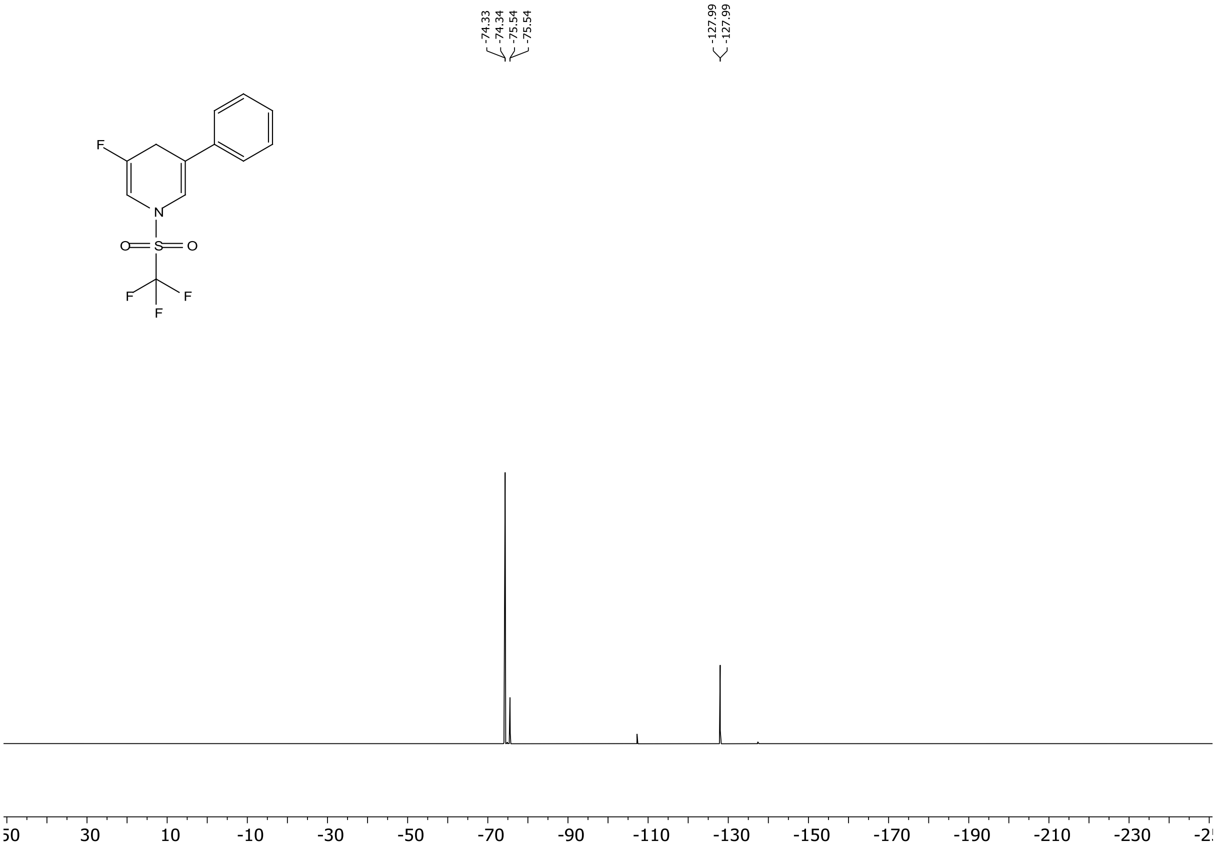

^{1}H NMR (400 MHz, benzene-d_6) – compound **19**

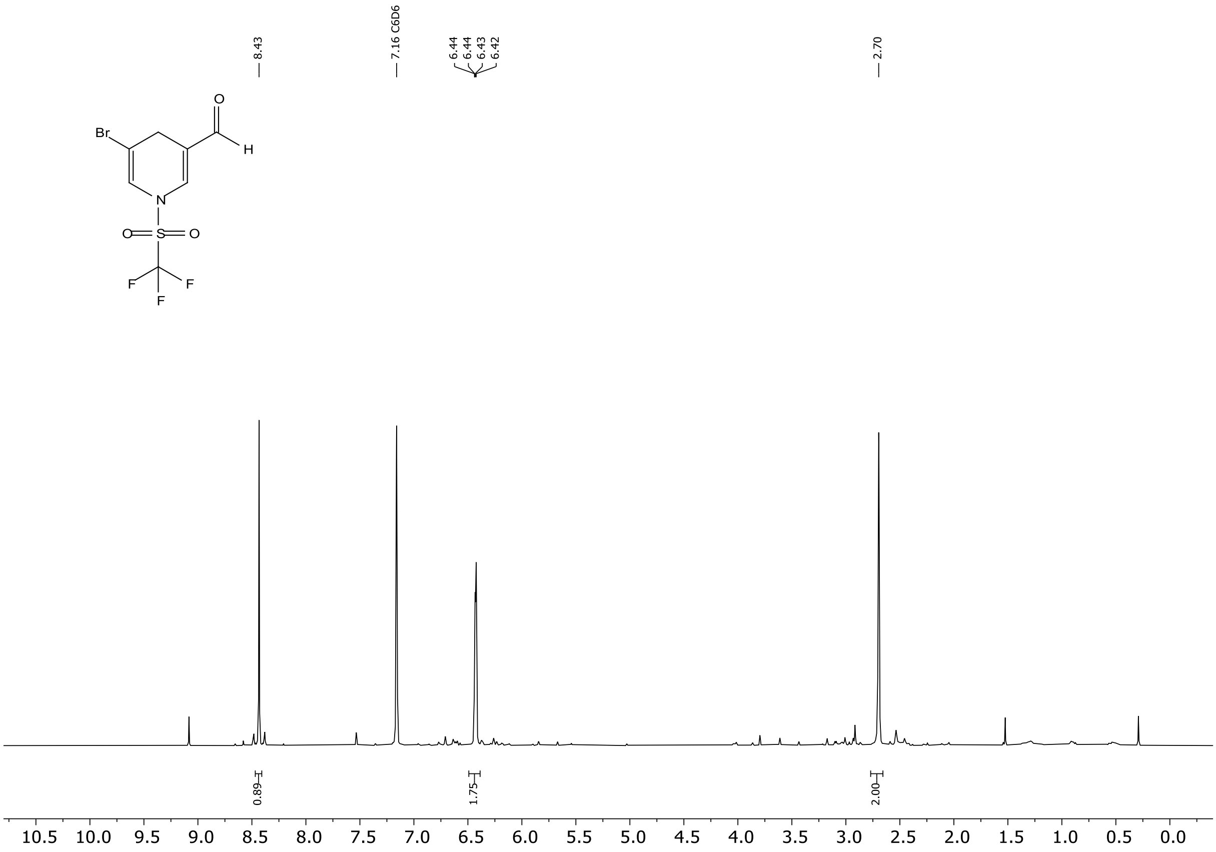

^{13}C{^{1}H} NMR (101 MHz, benzene-d_6) – compound **19**

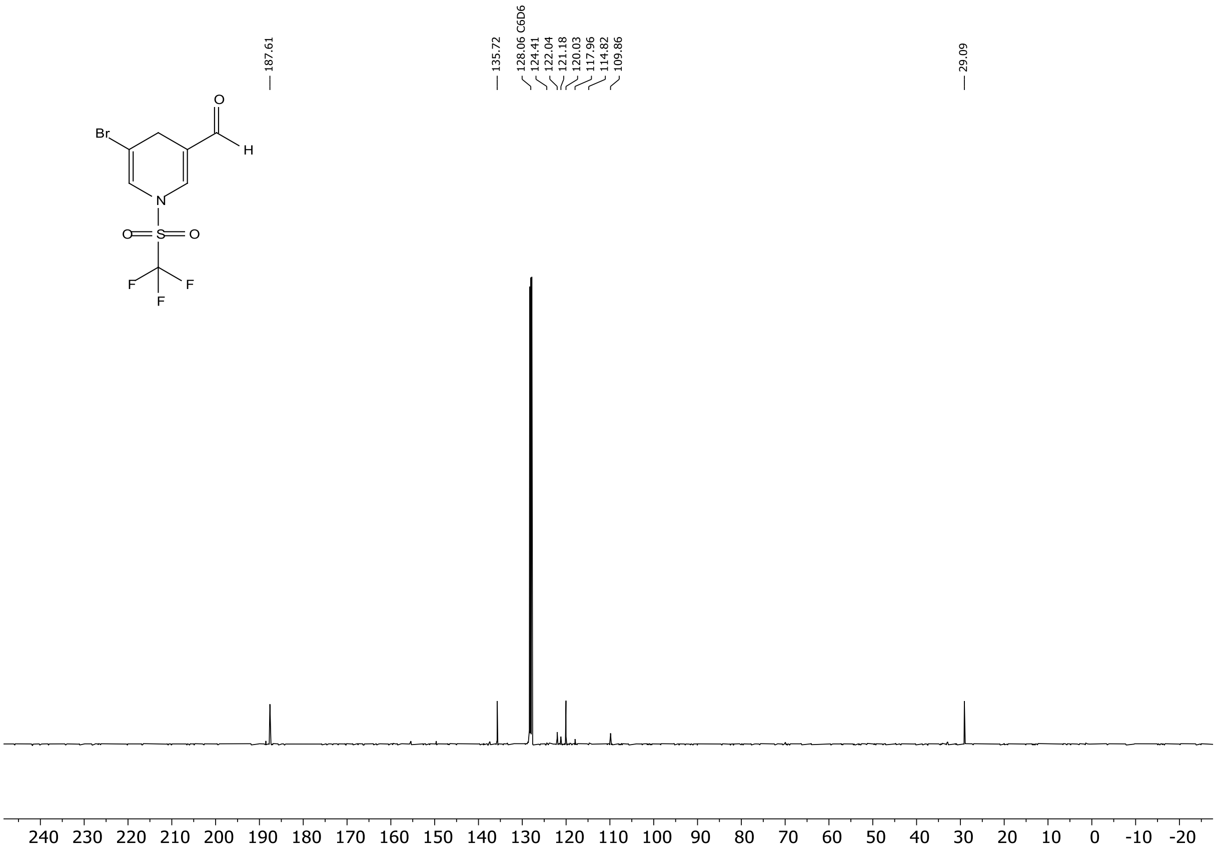

^{19}**F{^{1}H} NMR** (377 MHz, benzene-d_6) – compound **19**

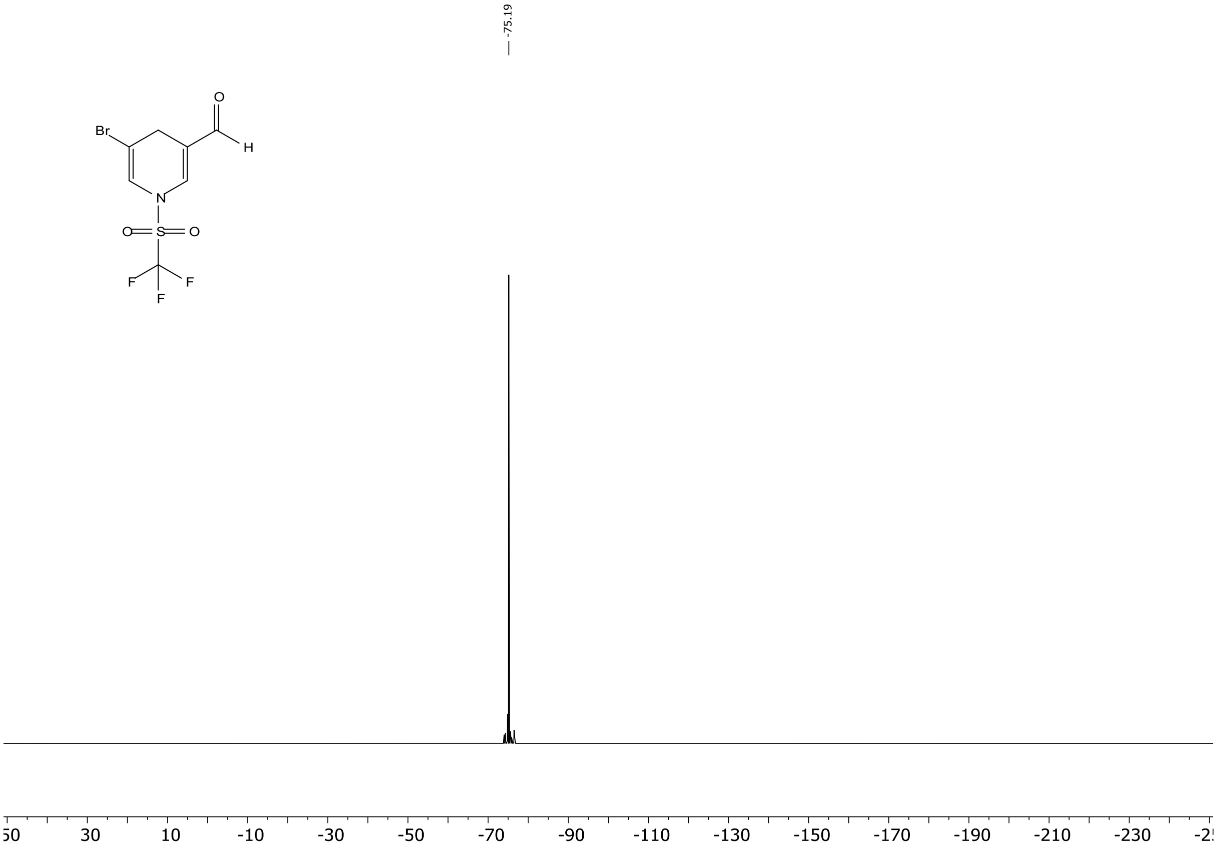

^{1}**H NMR** (400 MHz, benzene-d_6) – compound **20**

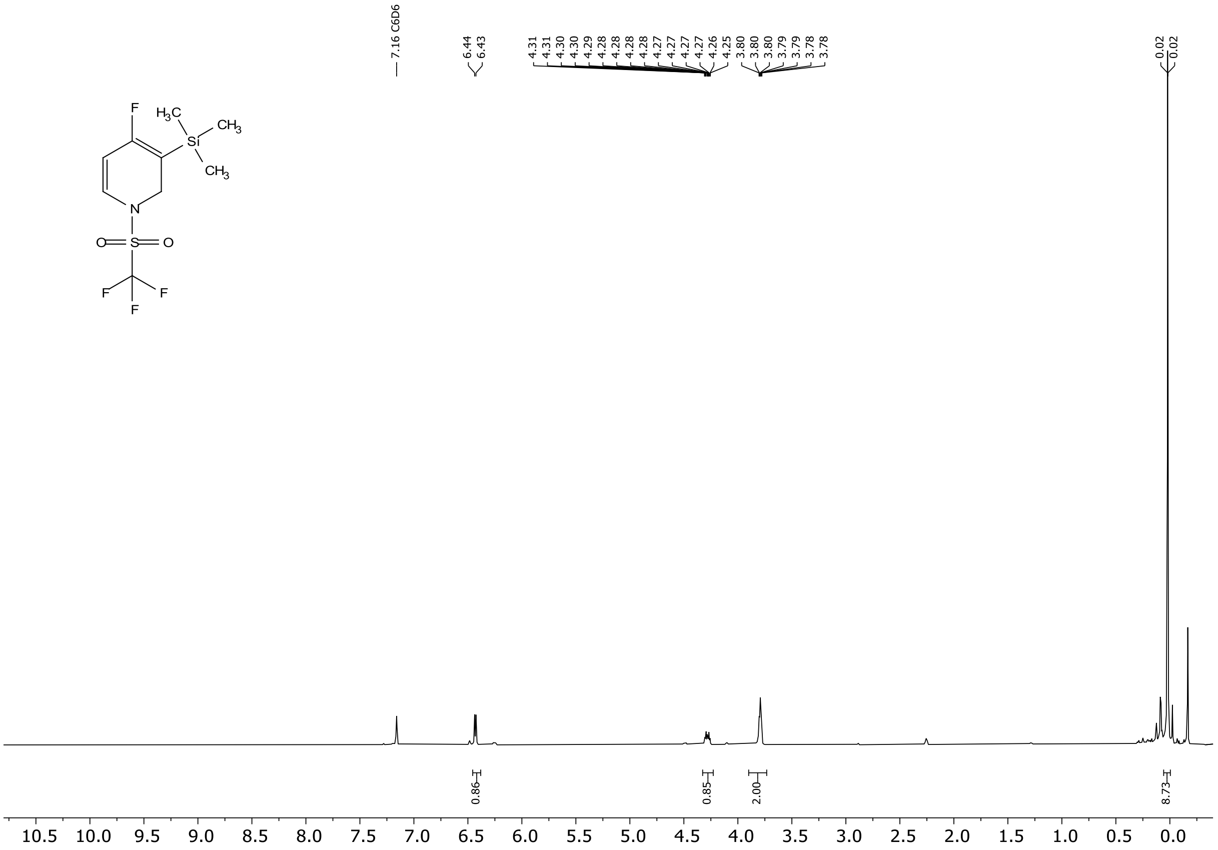

$^{13}C\{^{1}H\}$ NMR (101 MHz, benzene-d_6) – compound **20**

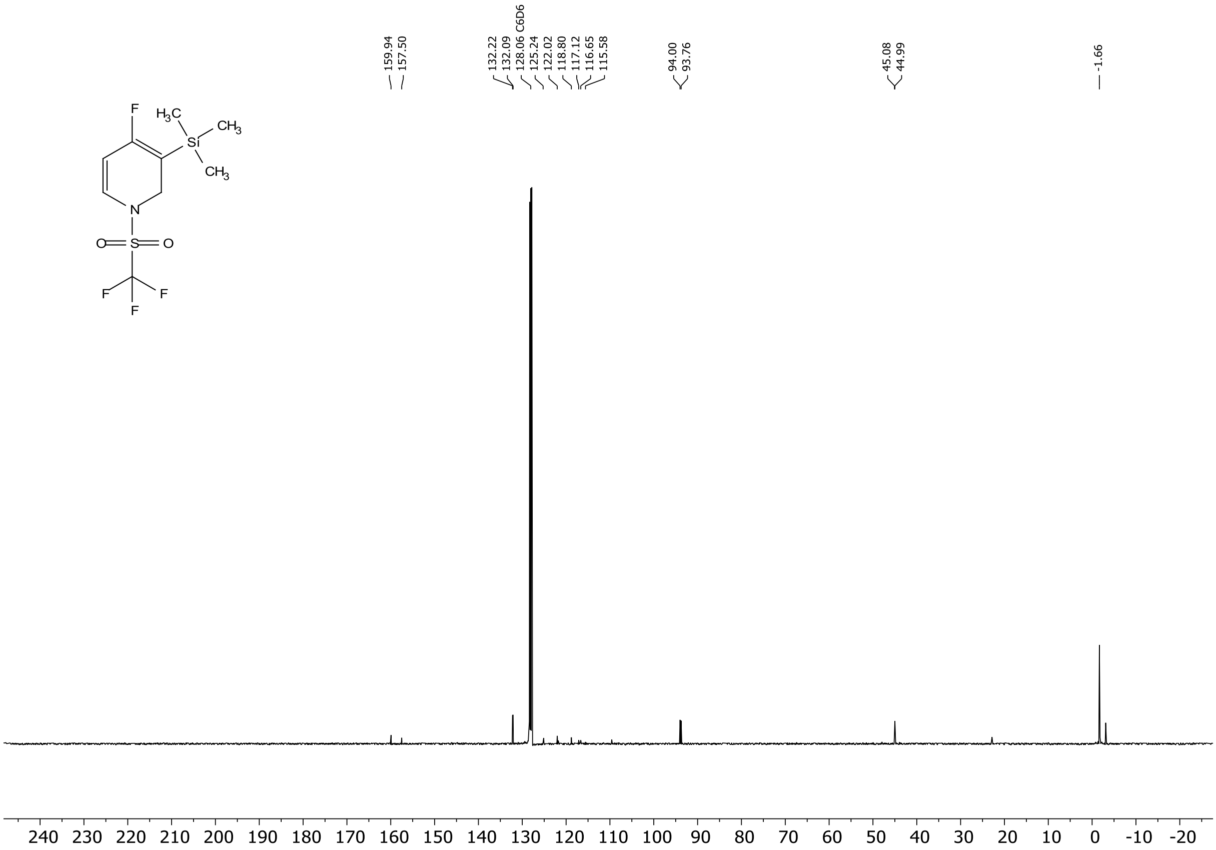

$^{19}F\{^{1}H\}$ NMR (377 MHz, benzene-d_6) – compound **20**

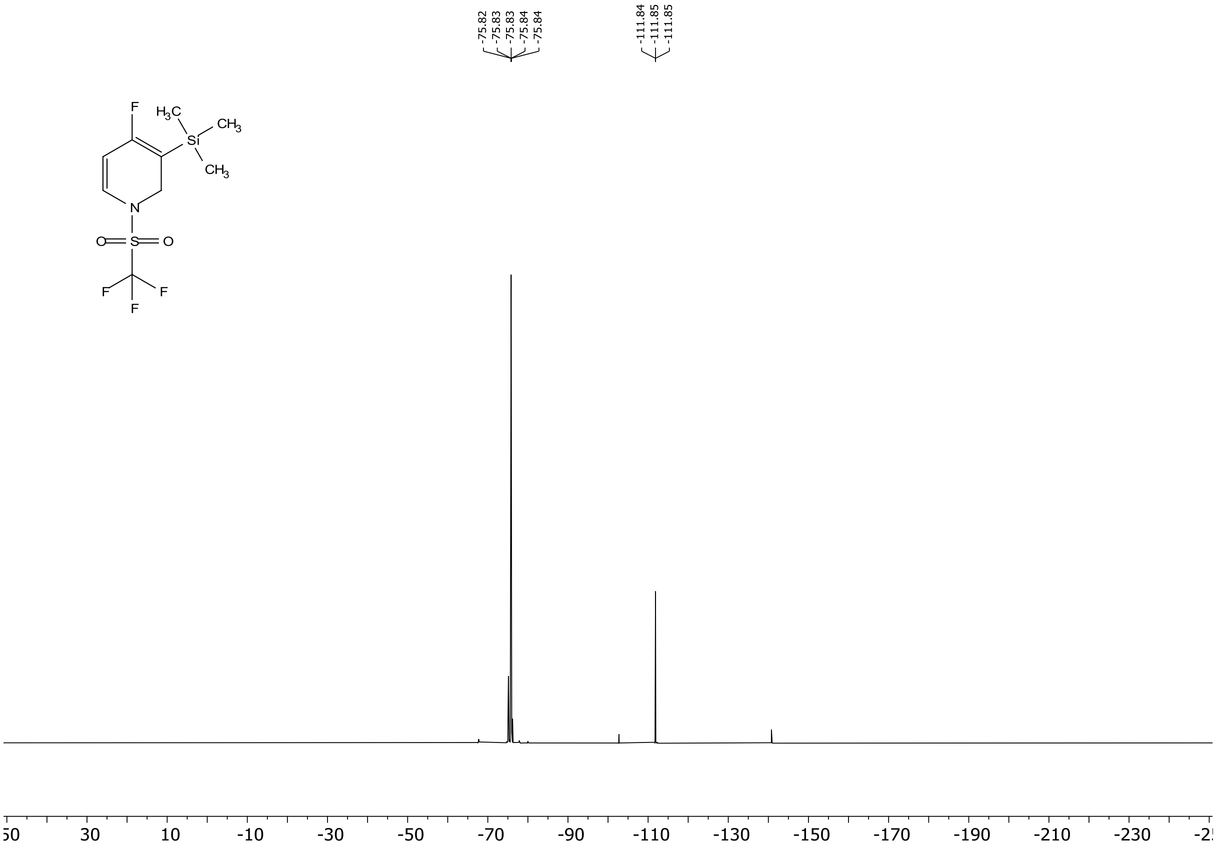

^{1}H NMR (400 MHz, benzene-d_6) – compound **21**

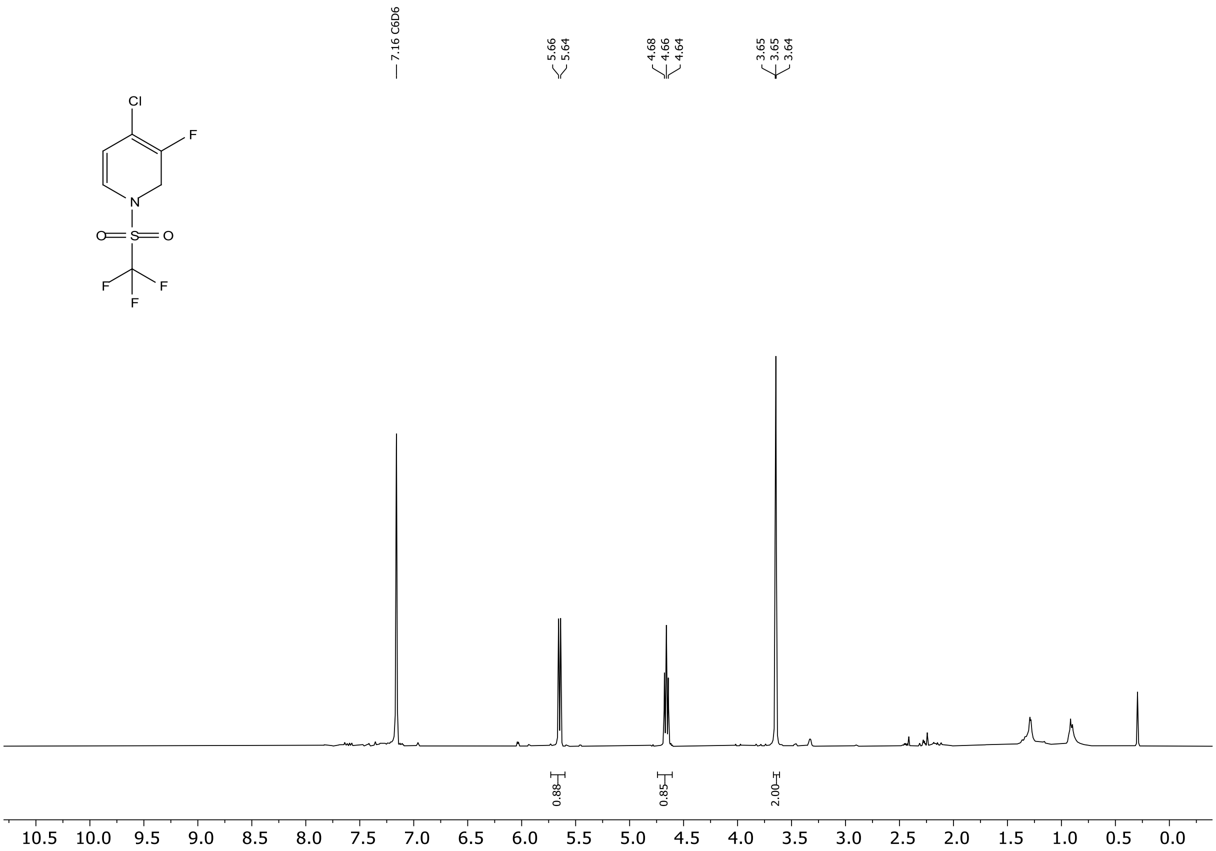

^{13}C{^{1}H} NMR (101 MHz, benzene-d_6) – compound **21**

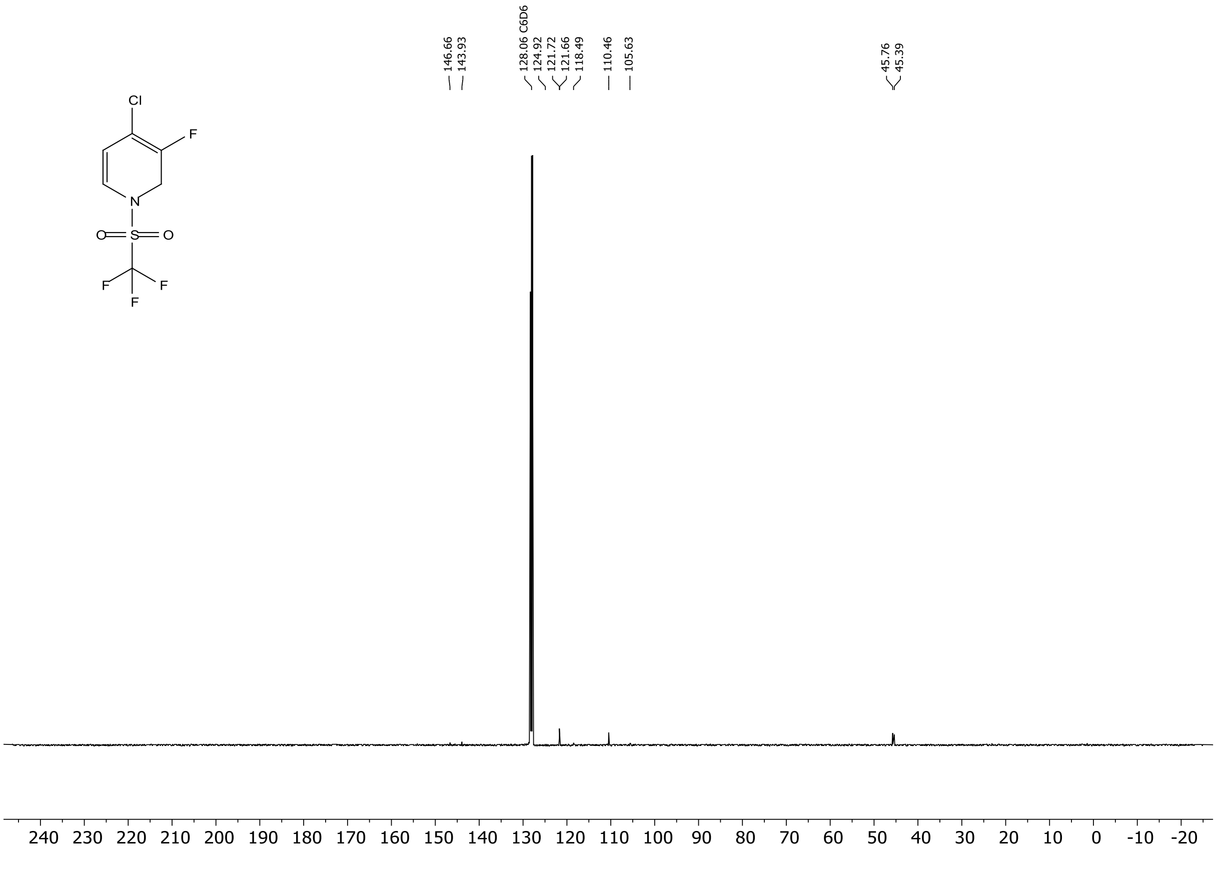

^{19}F{^{1}H} NMR (377 MHz, benzene-d_6) – compound **21**

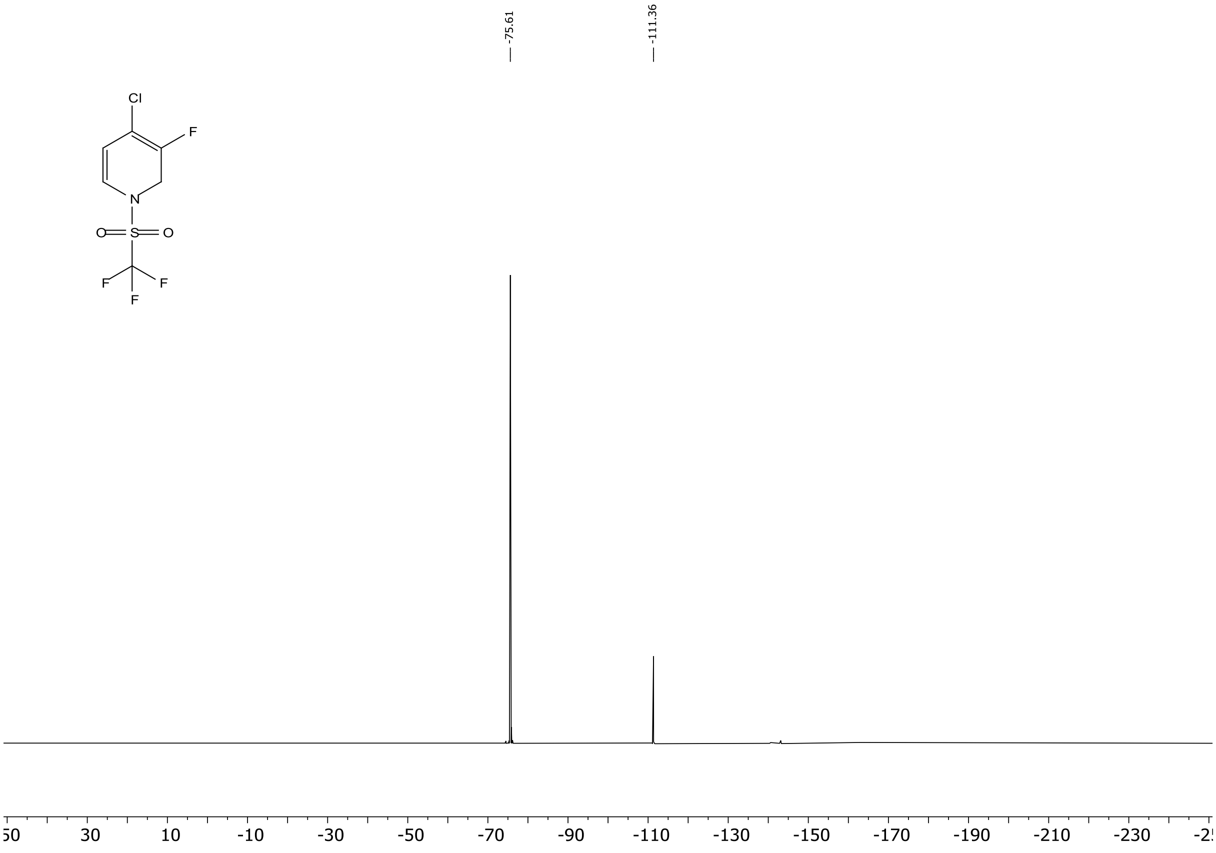

^{1}H NMR (400 MHz, benzene-d_6) – compound **22**

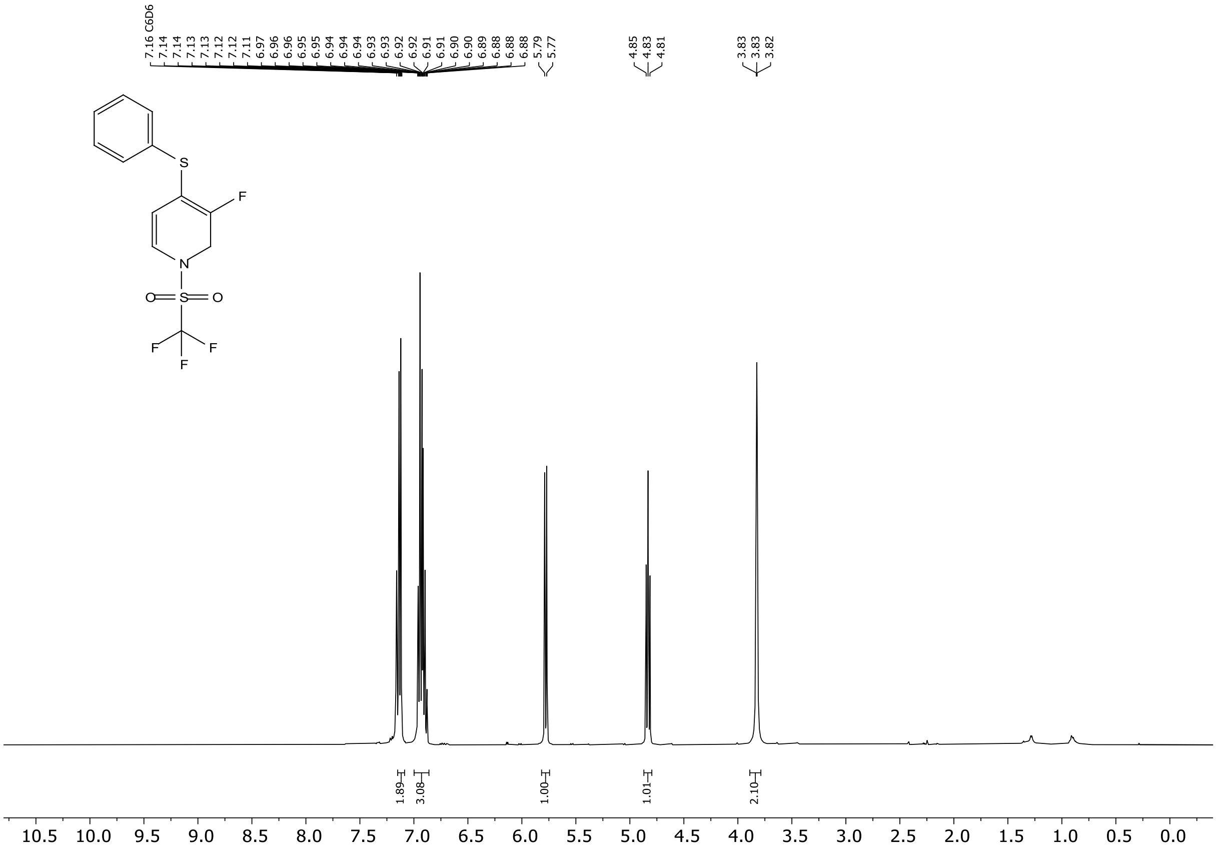

$^{13}C\{^1H\}$ NMR (101 MHz, benzene-d_6) – compound **22**

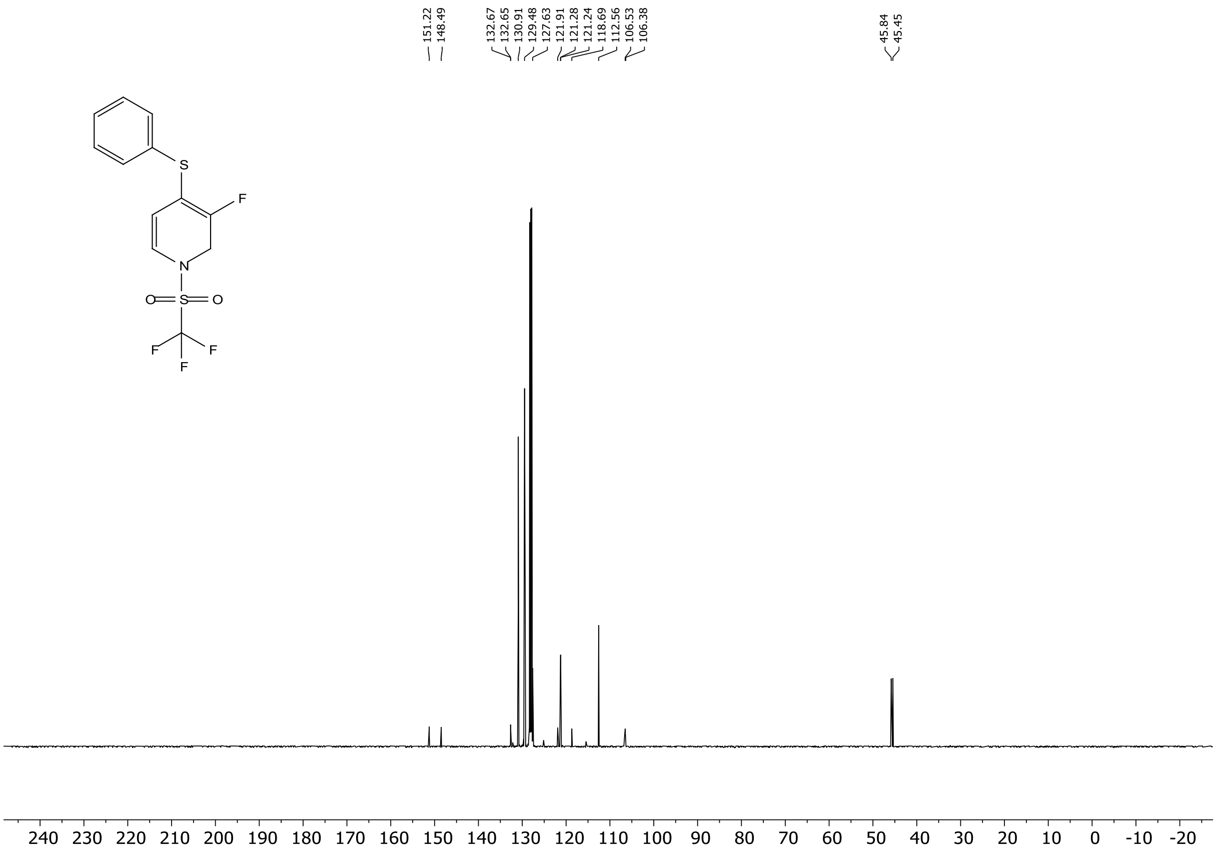

$^{19}F\{^1H\}$ NMR (377 MHz, benzene-d_6) – compound **22**

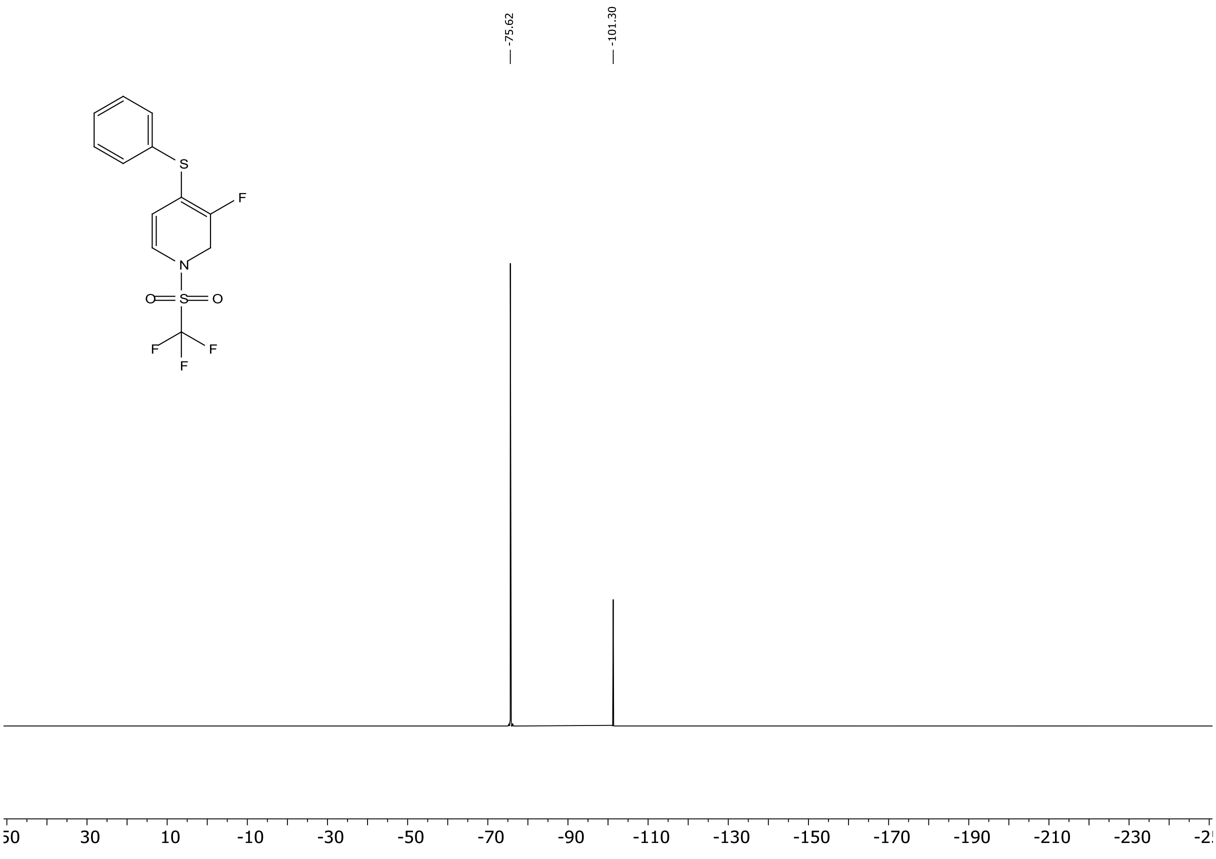

^{1}H NMR (400 MHz, benzene-d_6) – compound **23**

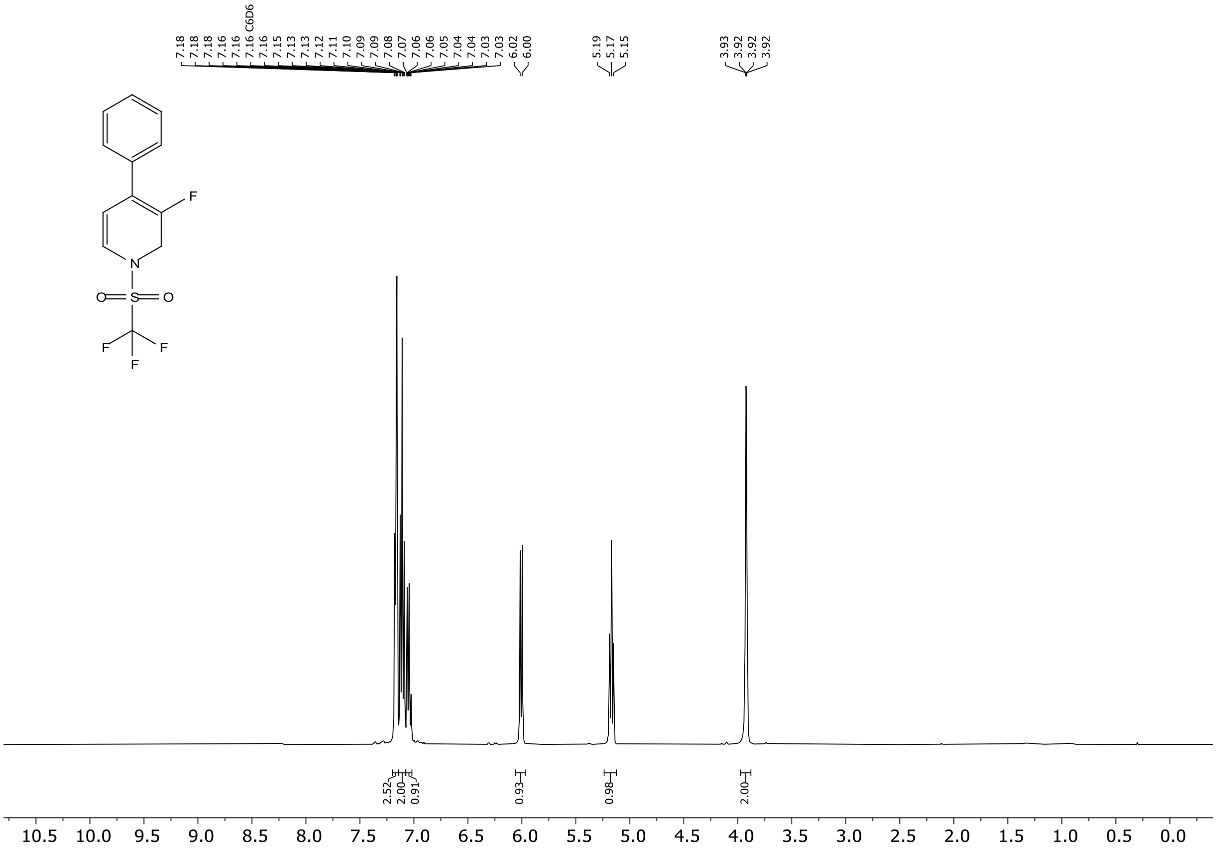

^{13}C{^{1}H} NMR (101 MHz, benzene-d_6) – compound **23**

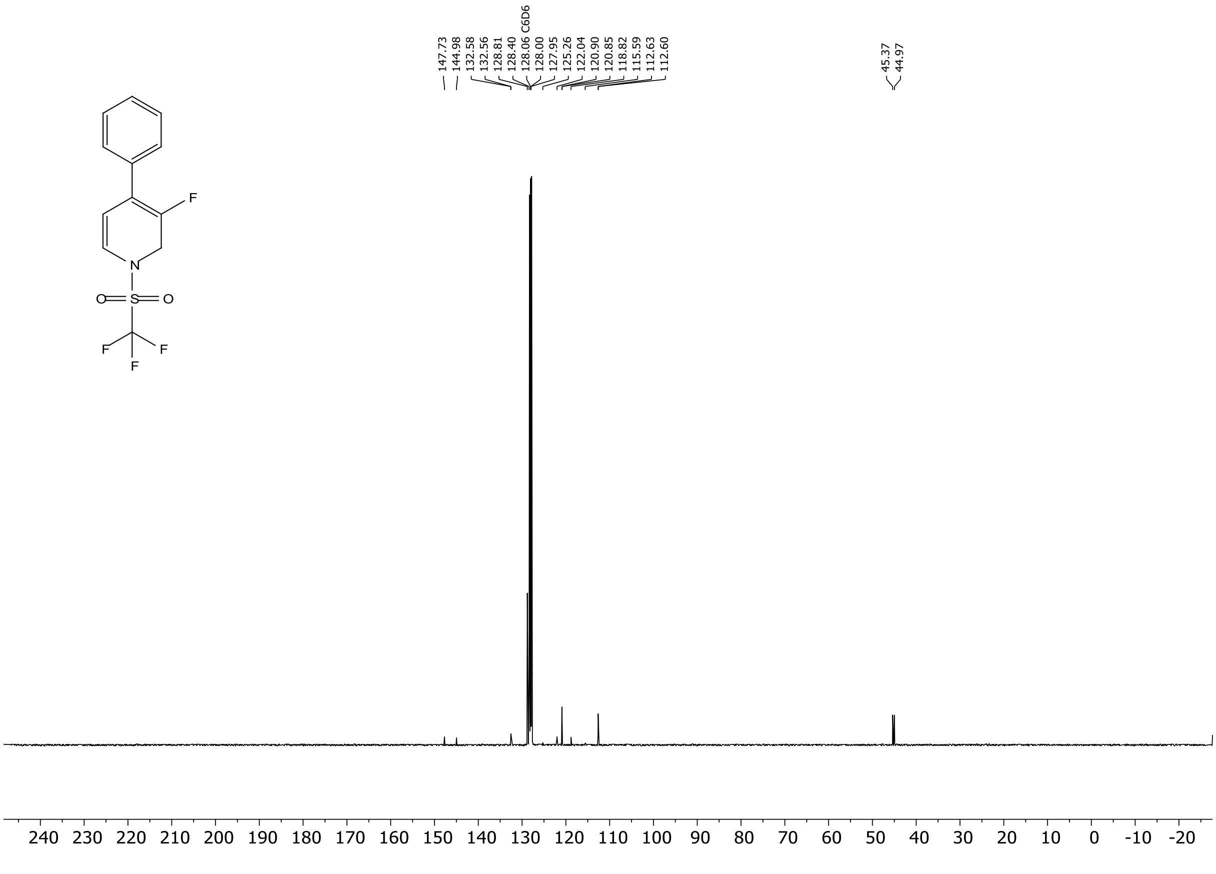

^{19}F{^{1}H} NMR (377 MHz, benzene-d_6) – compound **23**

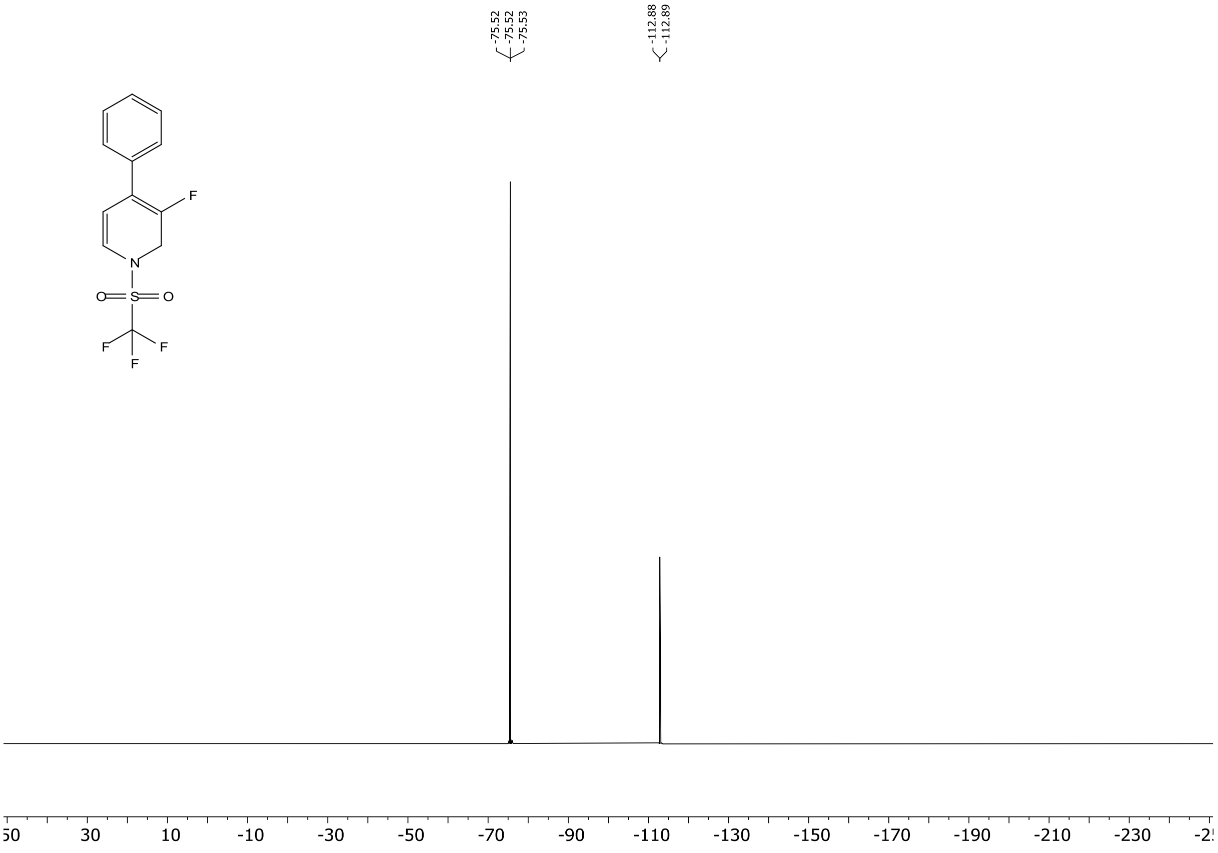

^{1}H NMR (400 MHz, chloroform-d_3) – compound **24**

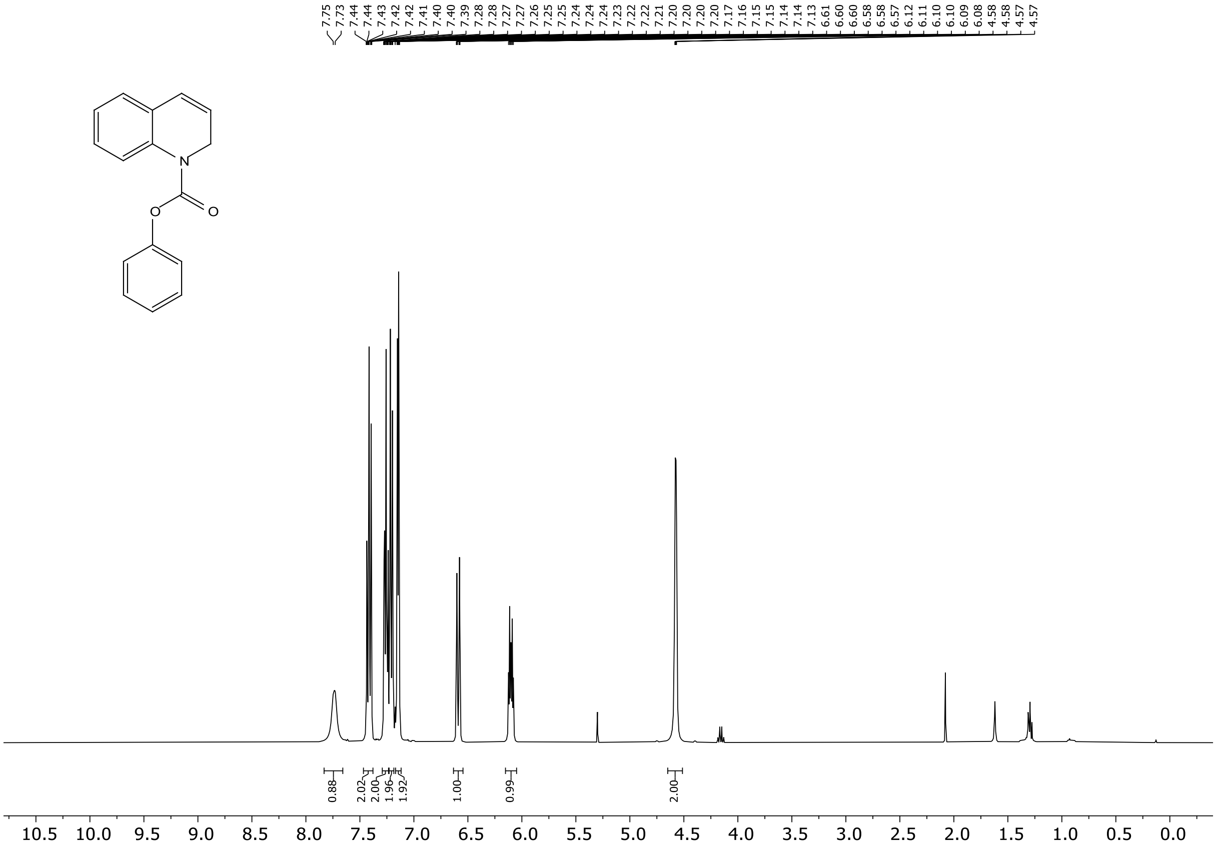

$^{13}C\{^1H\}$ NMR (101 MHz, chloroform-d_3) – compound **24**

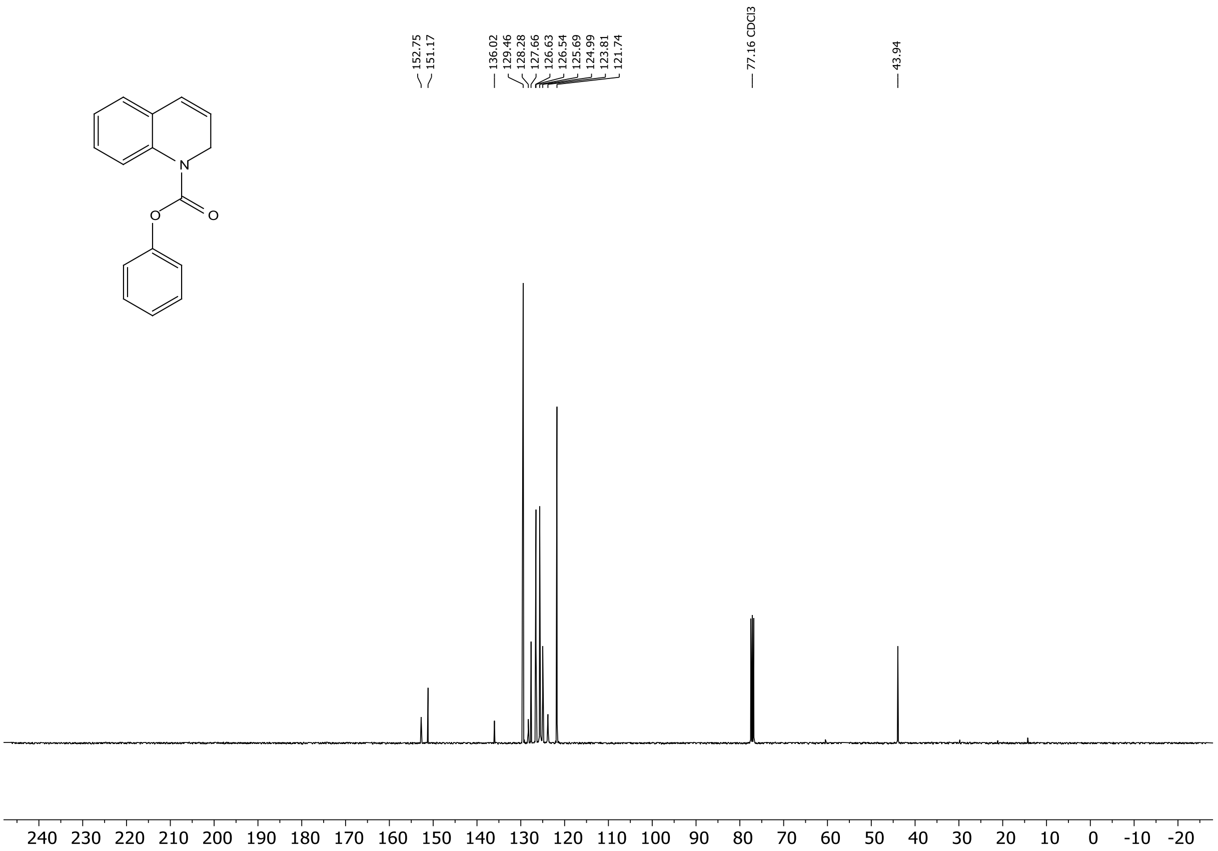

^1H NMR (400 MHz, chloroform-d_3) – compound **25**

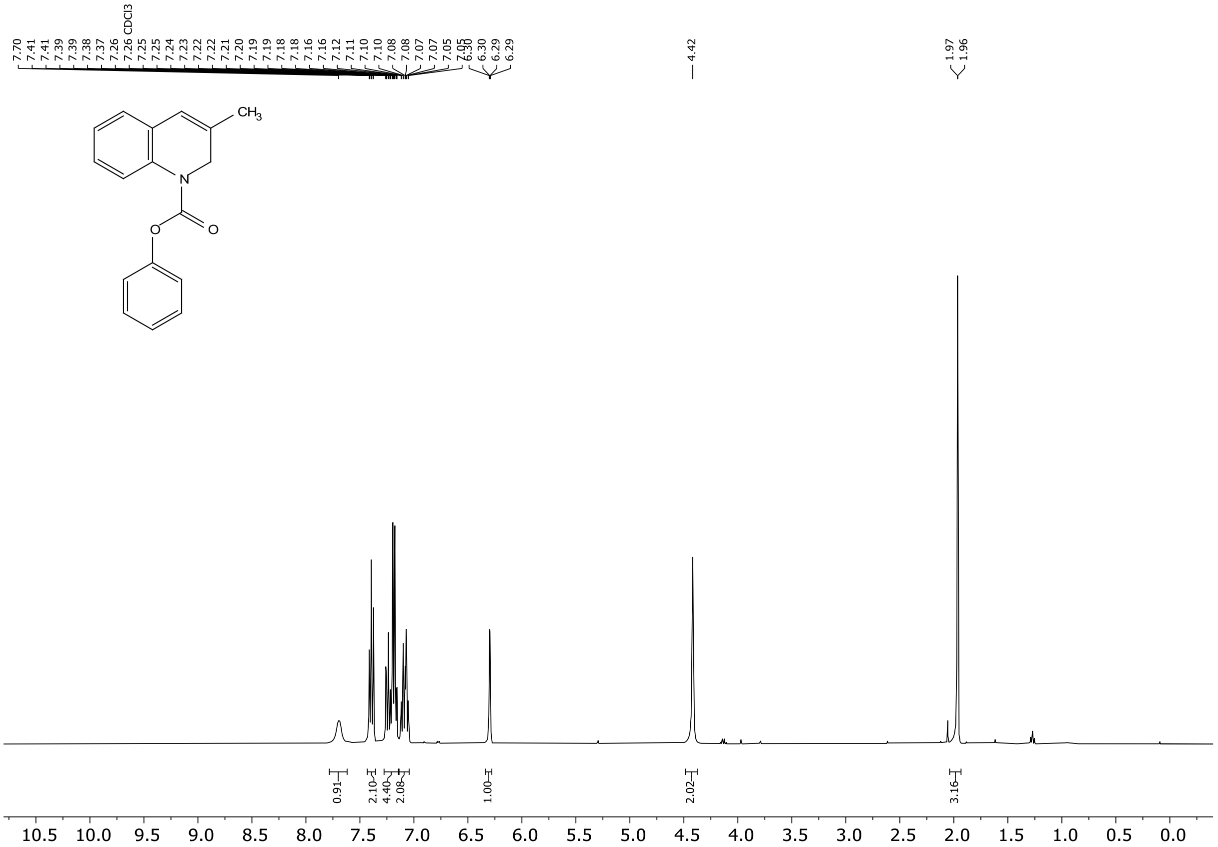

$^{13}C\{^1H\}$ NMR (101 MHz, chloroform-d_3) – compound **25**

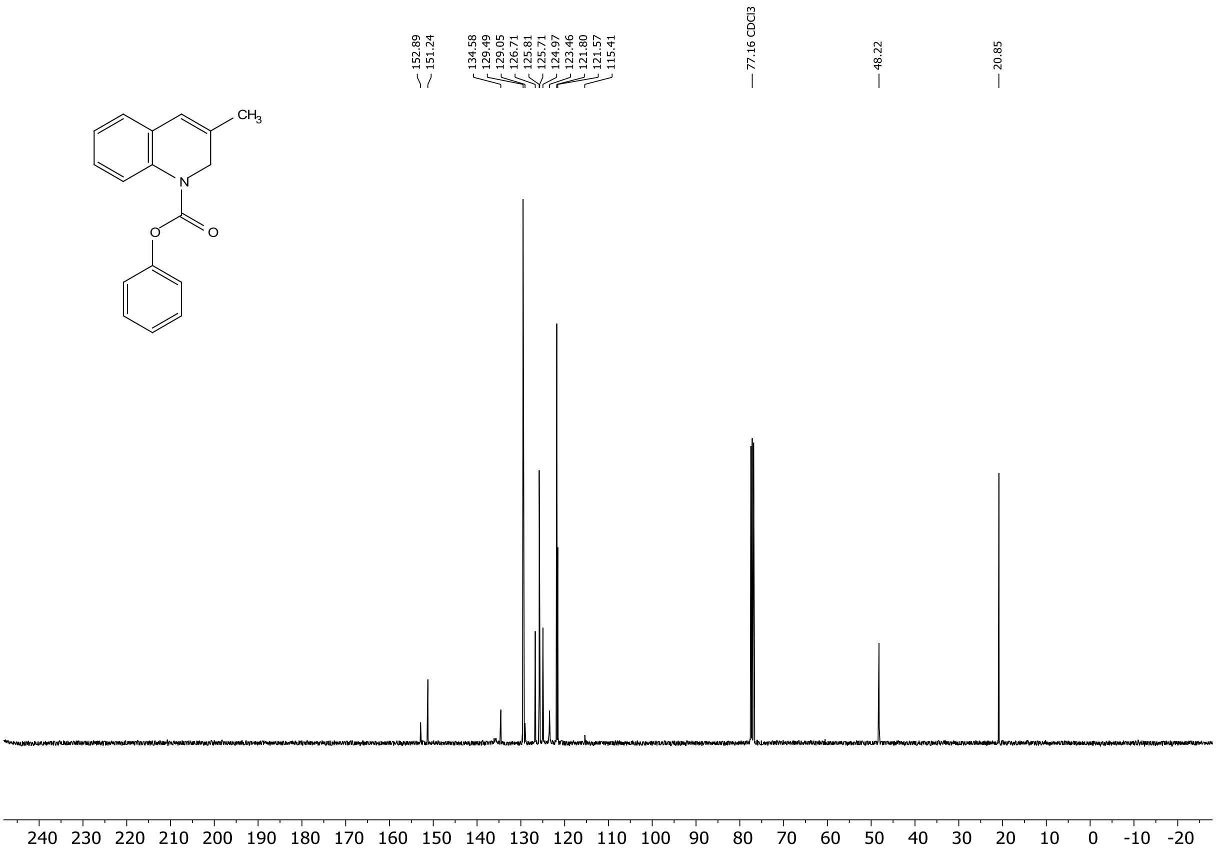

^1H NMR (400 MHz, chloroform-d_3) – compound **26**

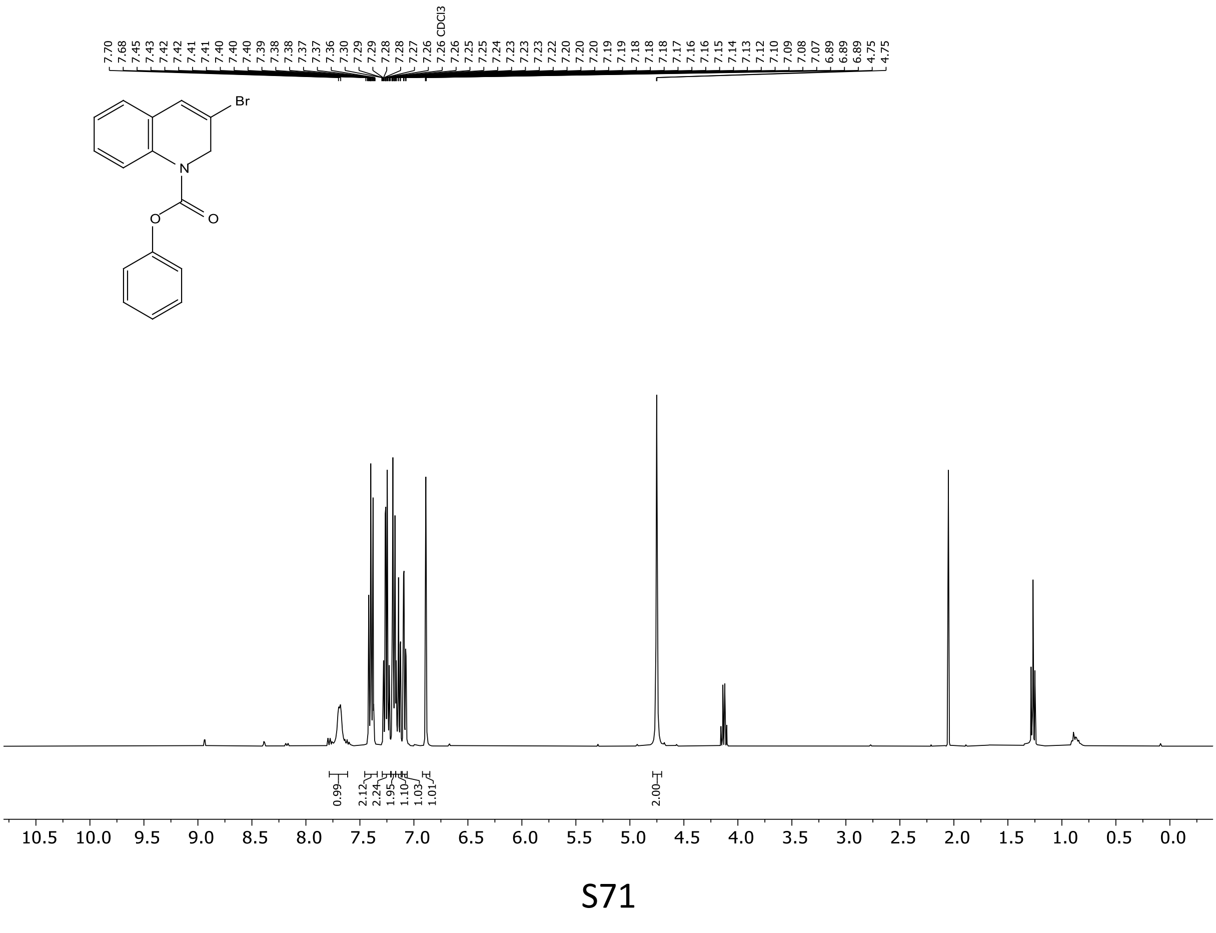

$^{13}C\{^1H\}$ NMR (101 MHz, chloroform-d_3) – compound **26**

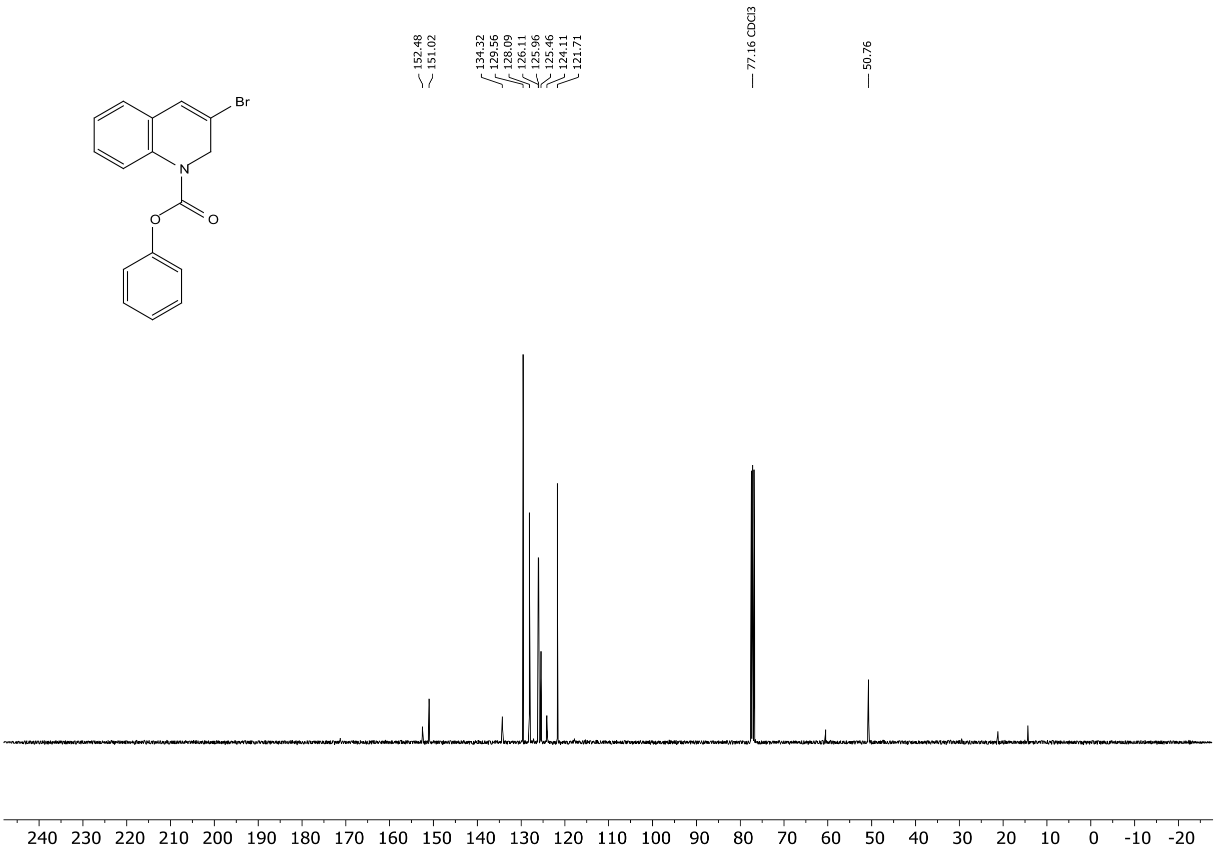

^1H NMR (400 MHz, benzene-d_6) – compound **27**

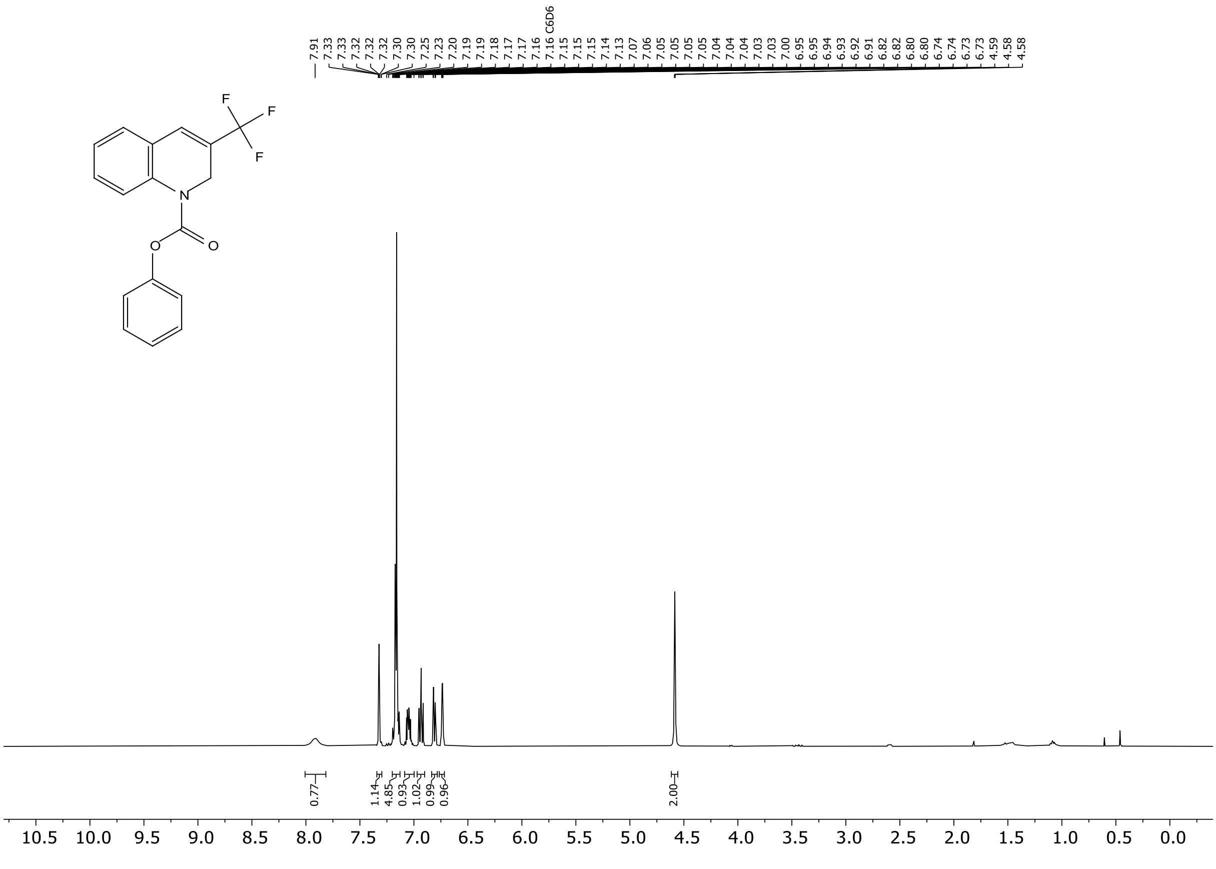

^{13}C{^{1}H} NMR (101 MHz, benzene-d_6) – compound **27**

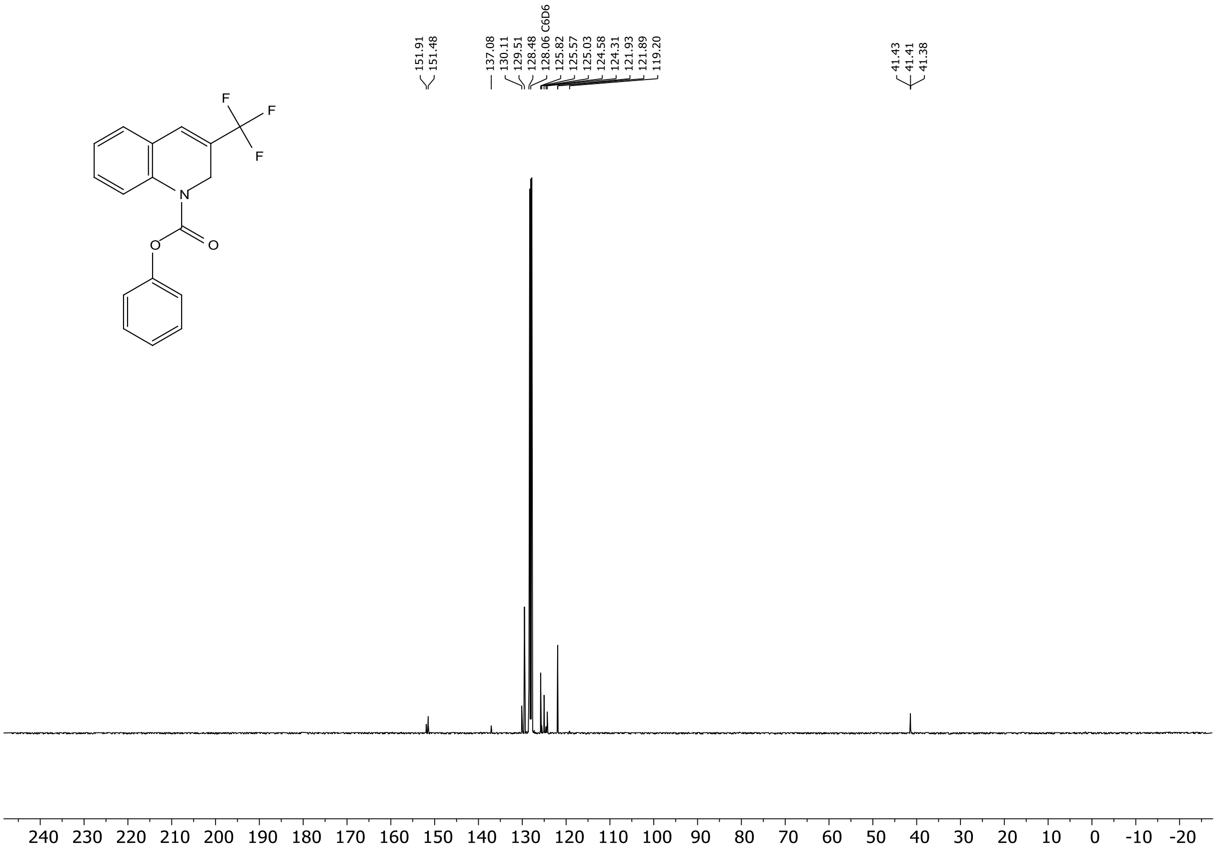

^{19}F{^{1}H} NMR (377 MHz, benzene-d_6) – compound **27**

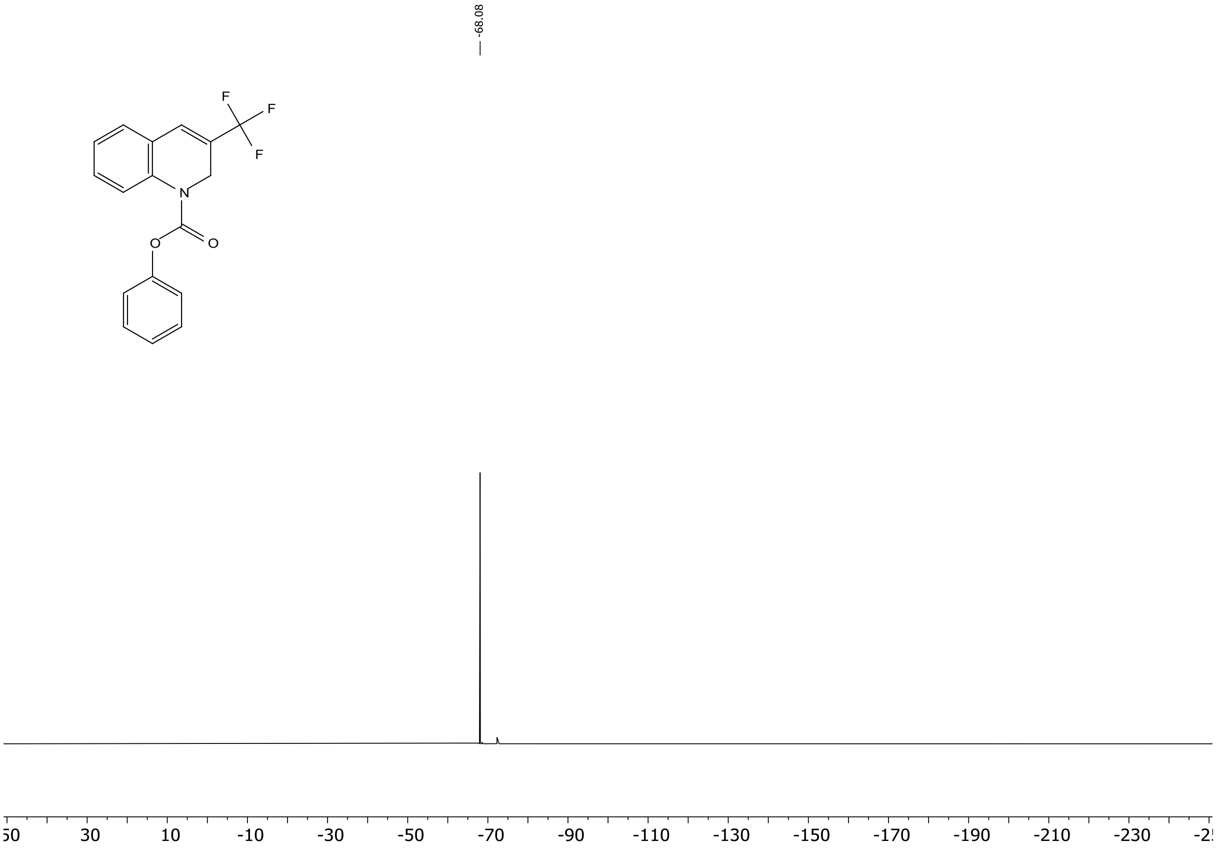

^{1}H NMR (400 MHz, benzene-d_6) – compound **28**

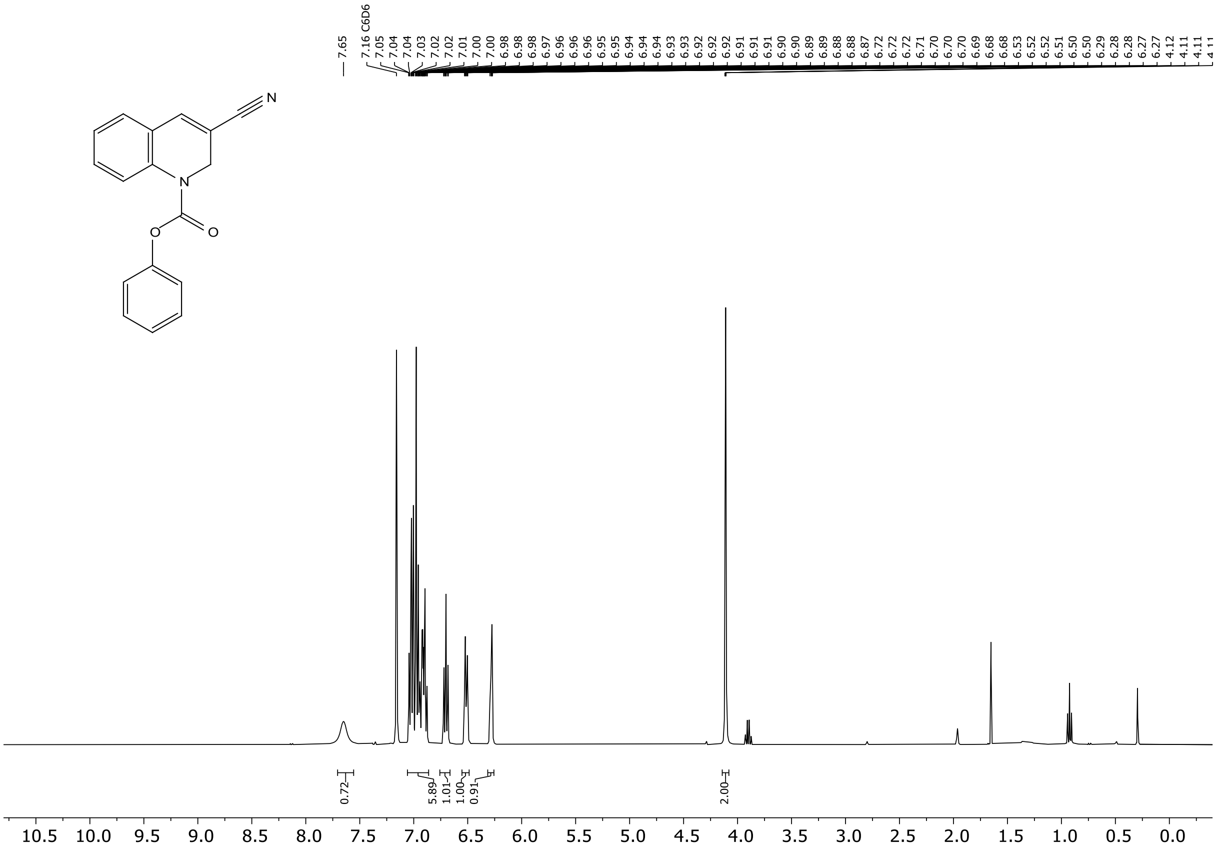

^{13}C{^{1}H} NMR (101 MHz, benzene-d_6) – compound **28**

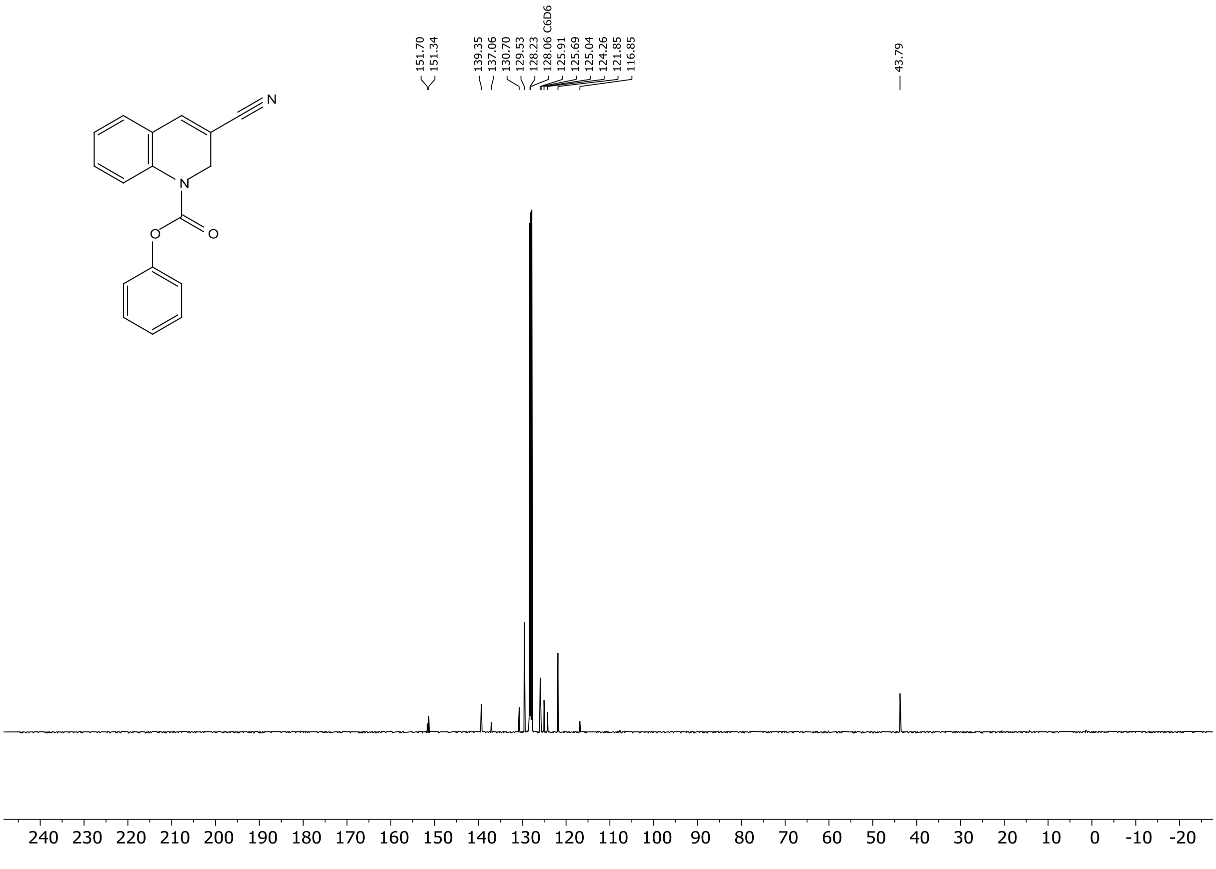

^{1}H NMR (400 MHz, chloroform-d_3) – compound **29**

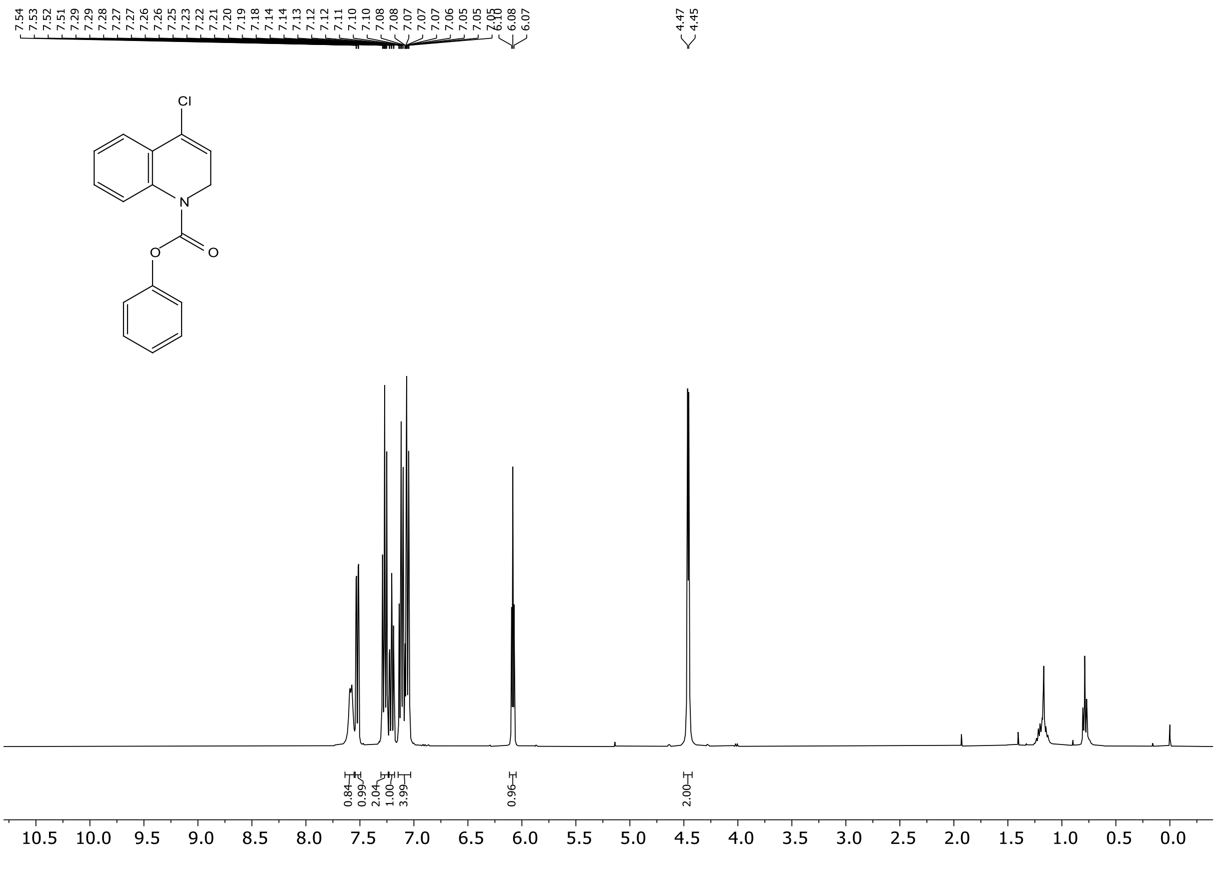

^{13}C{^{1}H} NMR (101 MHz, chloroform-d_3) – compound **29**

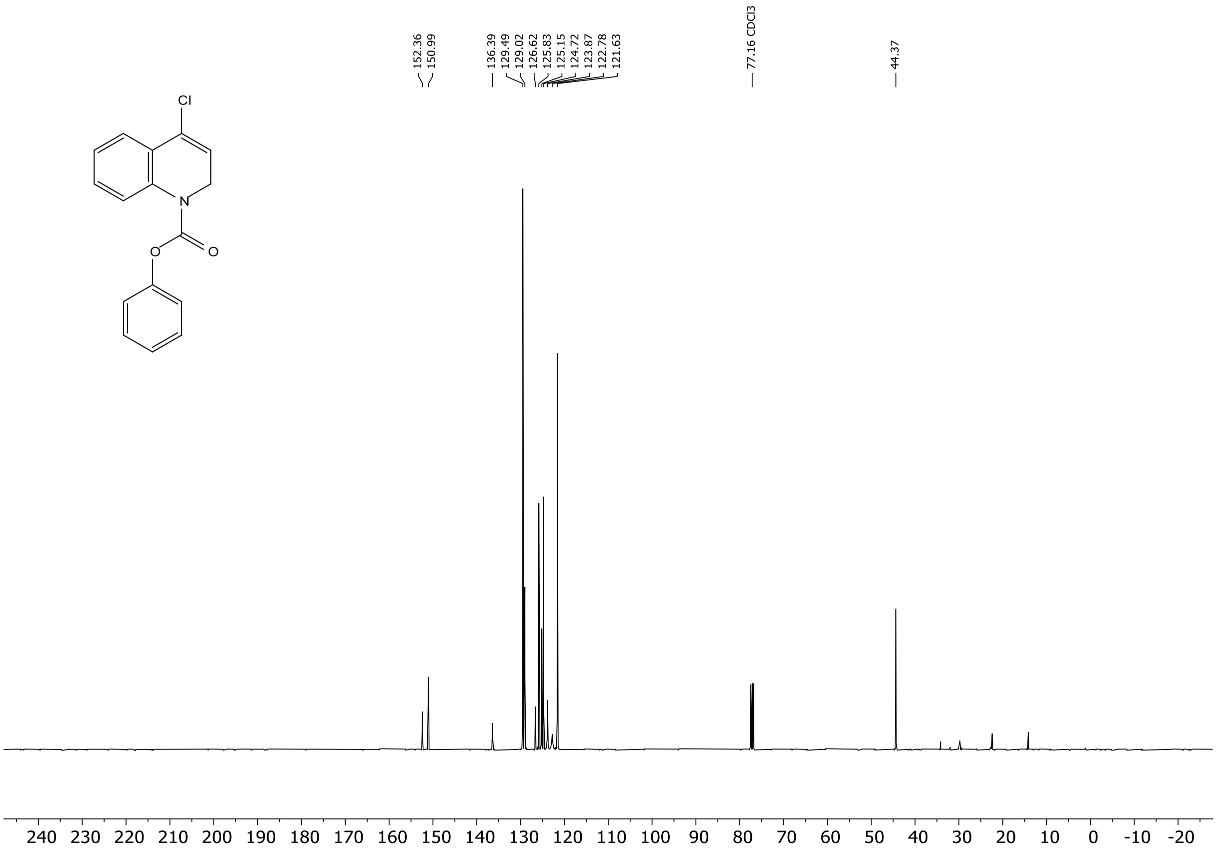

^{1}H NMR (400 MHz, chloroform-d_3) – compound **30**

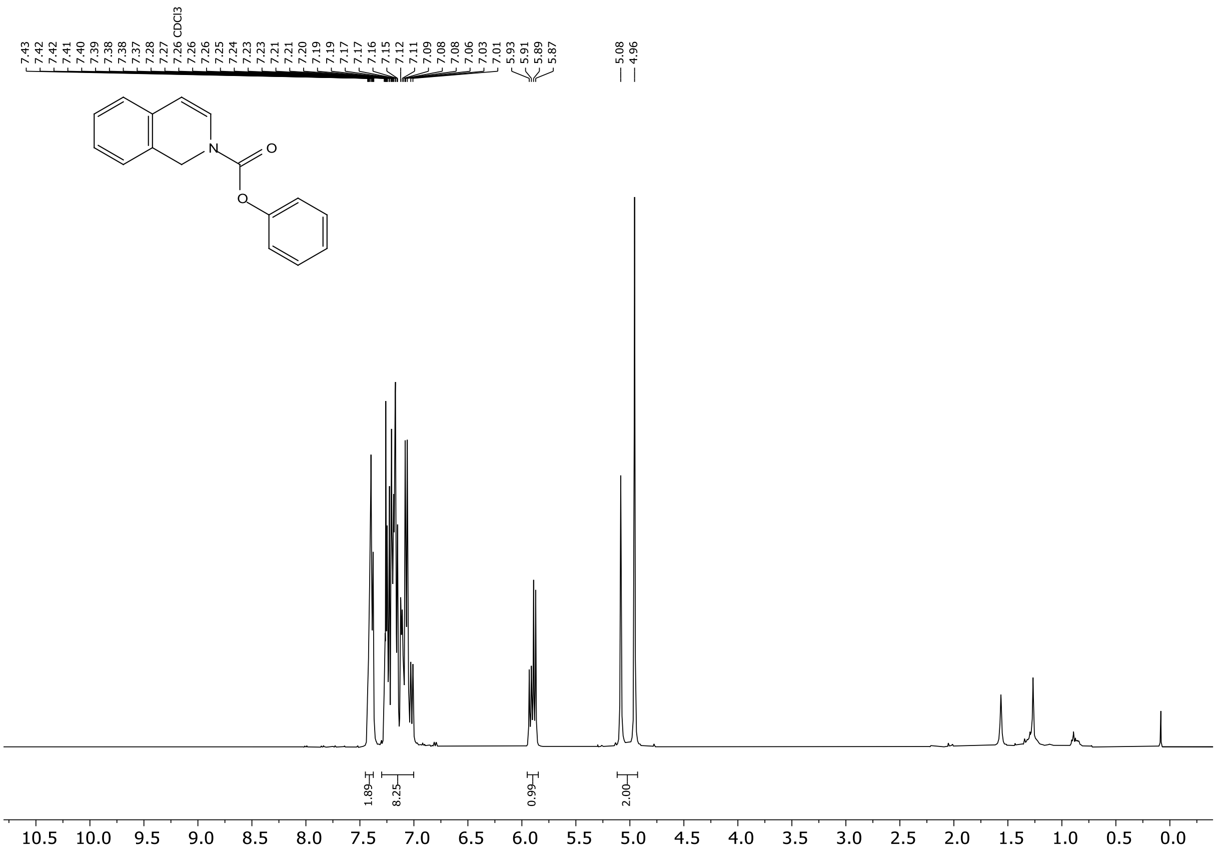

^{13}C{^{1}H} NMR (101 MHz, chloroform-d_3) – compound **30**

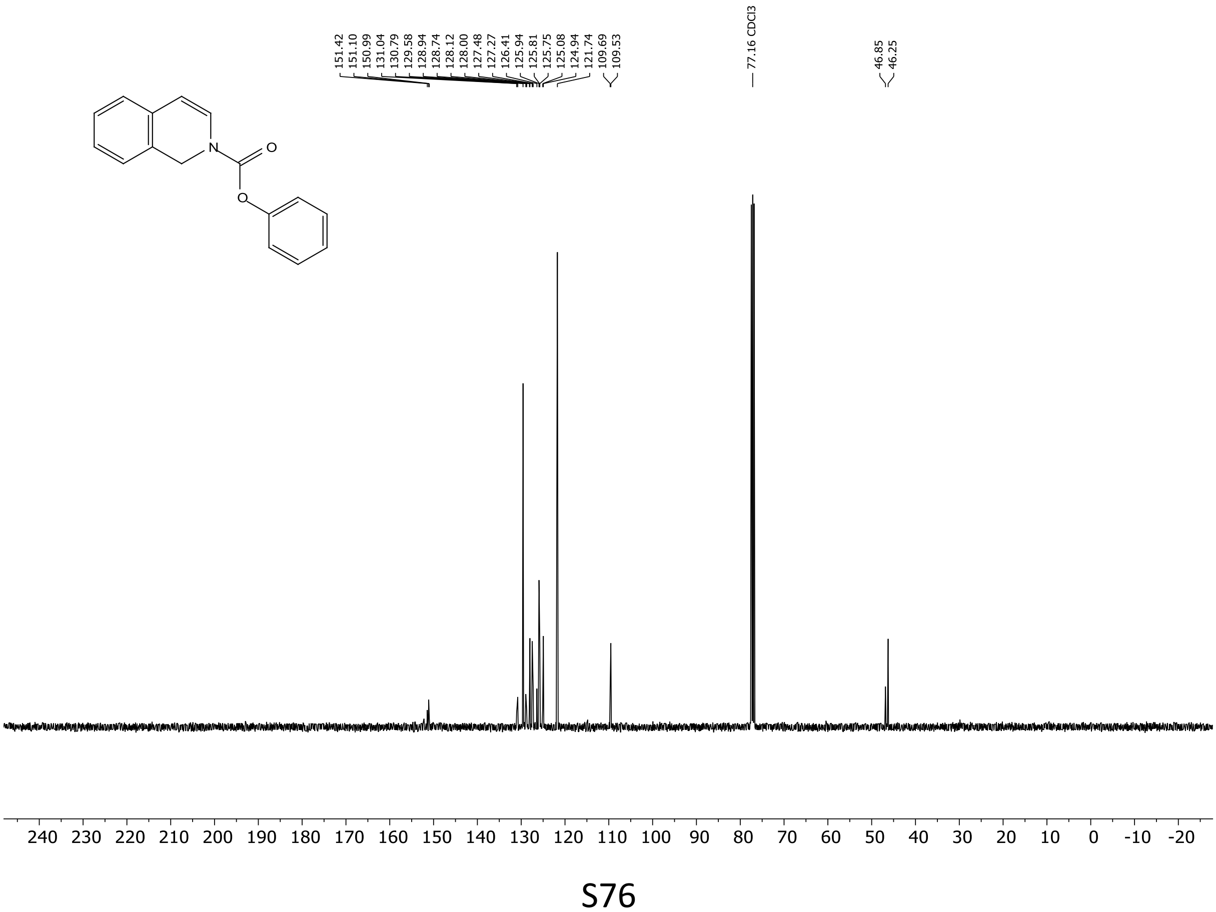

^{1}**H NMR** (599 MHz, toluene-d_8, T = 363 K) – compound **31**

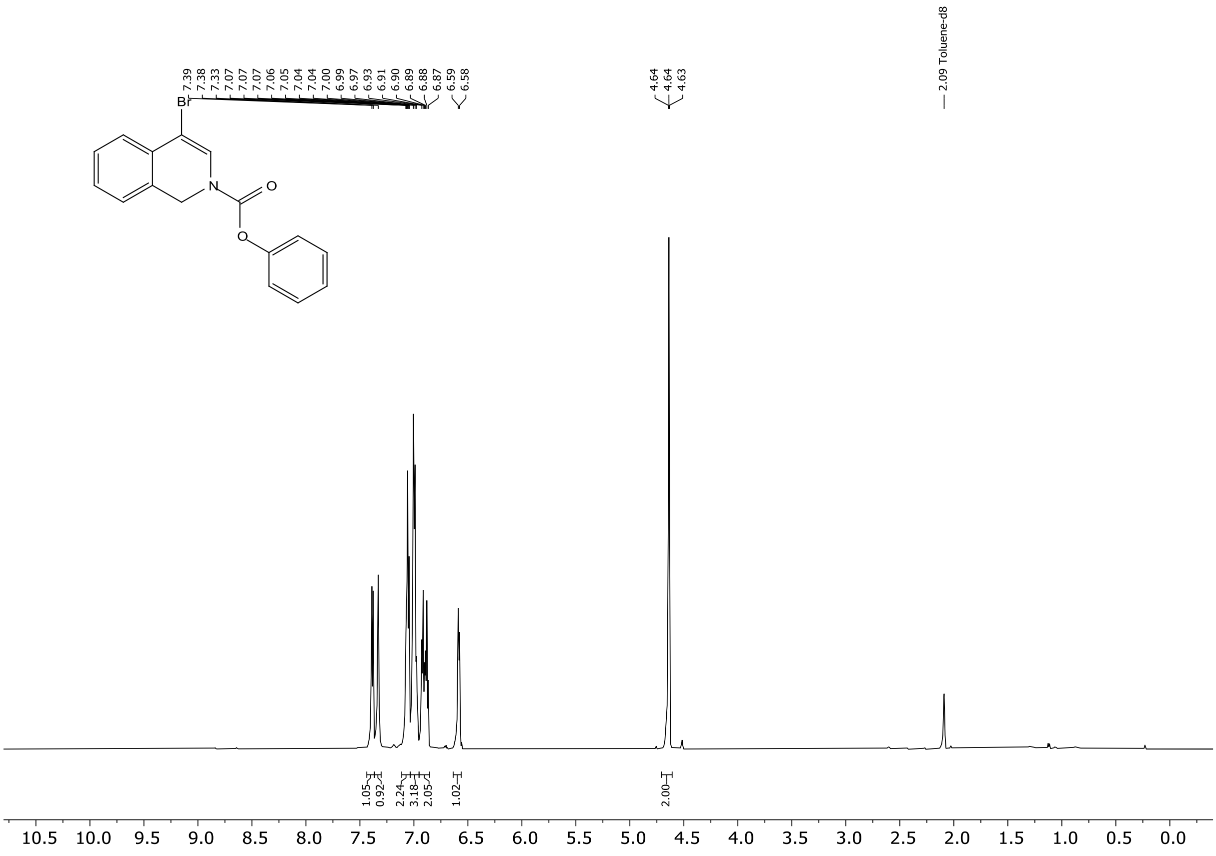

^{13}**C{^{1}H} NMR** (151 MHz, toluene-d_8, T = 363 K) – compound **31**

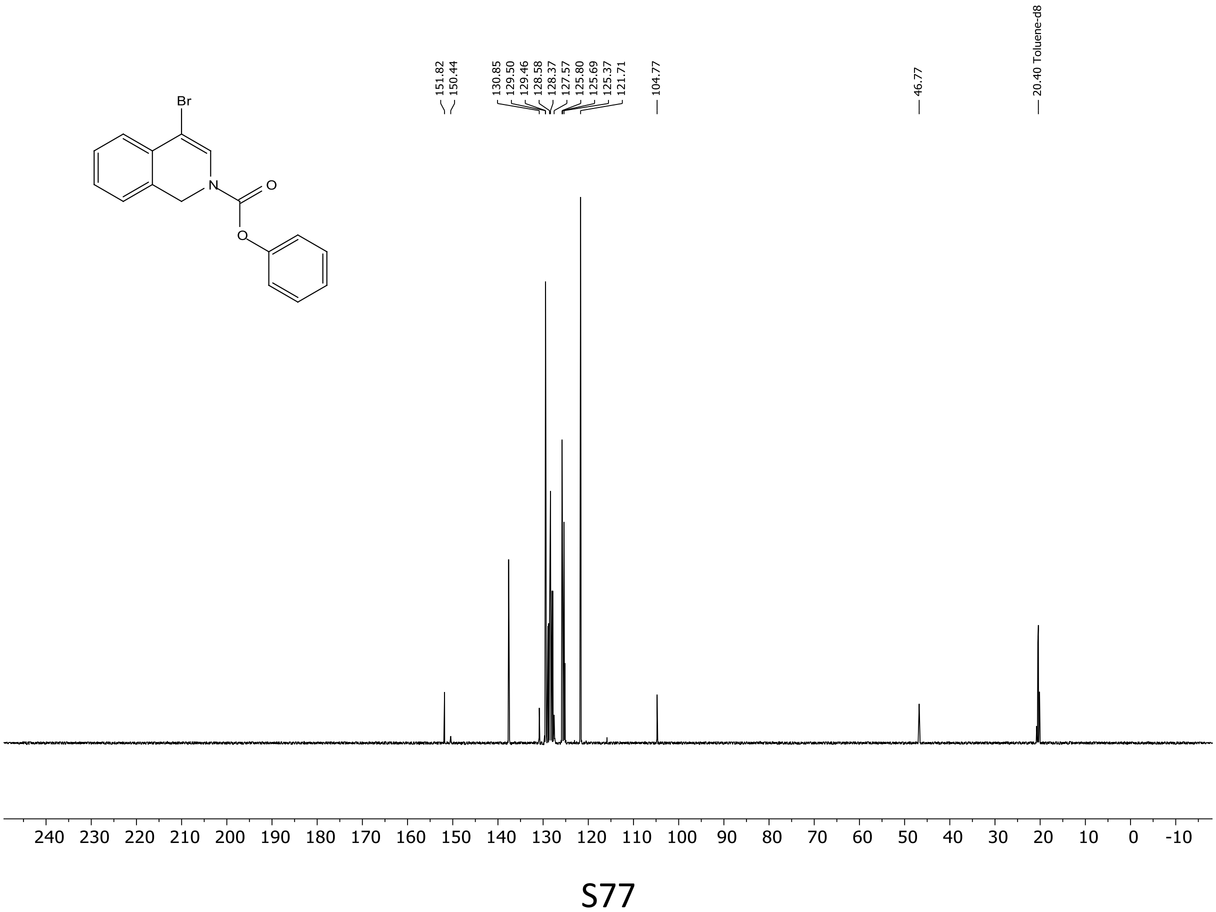

^{1}H NMR (400 MHz, chloroform-d_3) – compound **32**

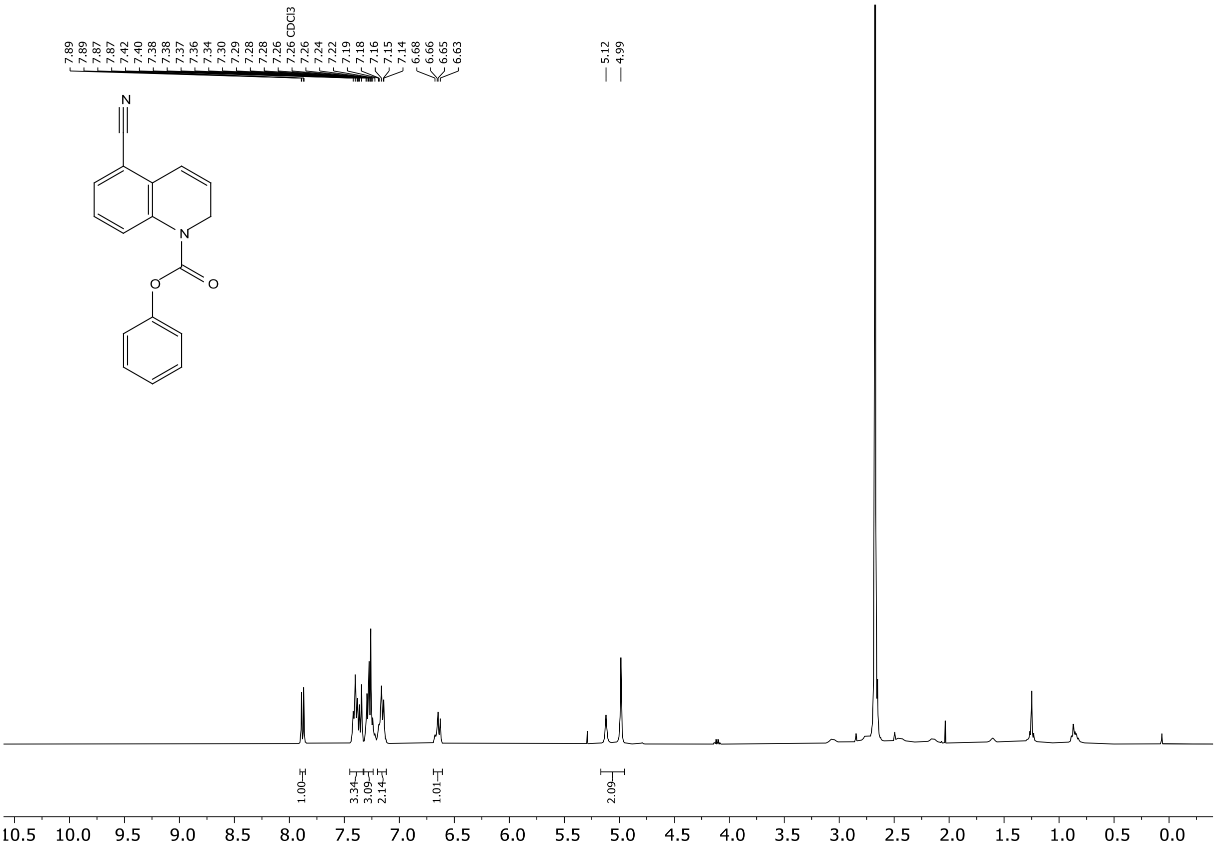

^{13}C{^{1}H} NMR (101 MHz, chloroform-d_3) – compound **32**

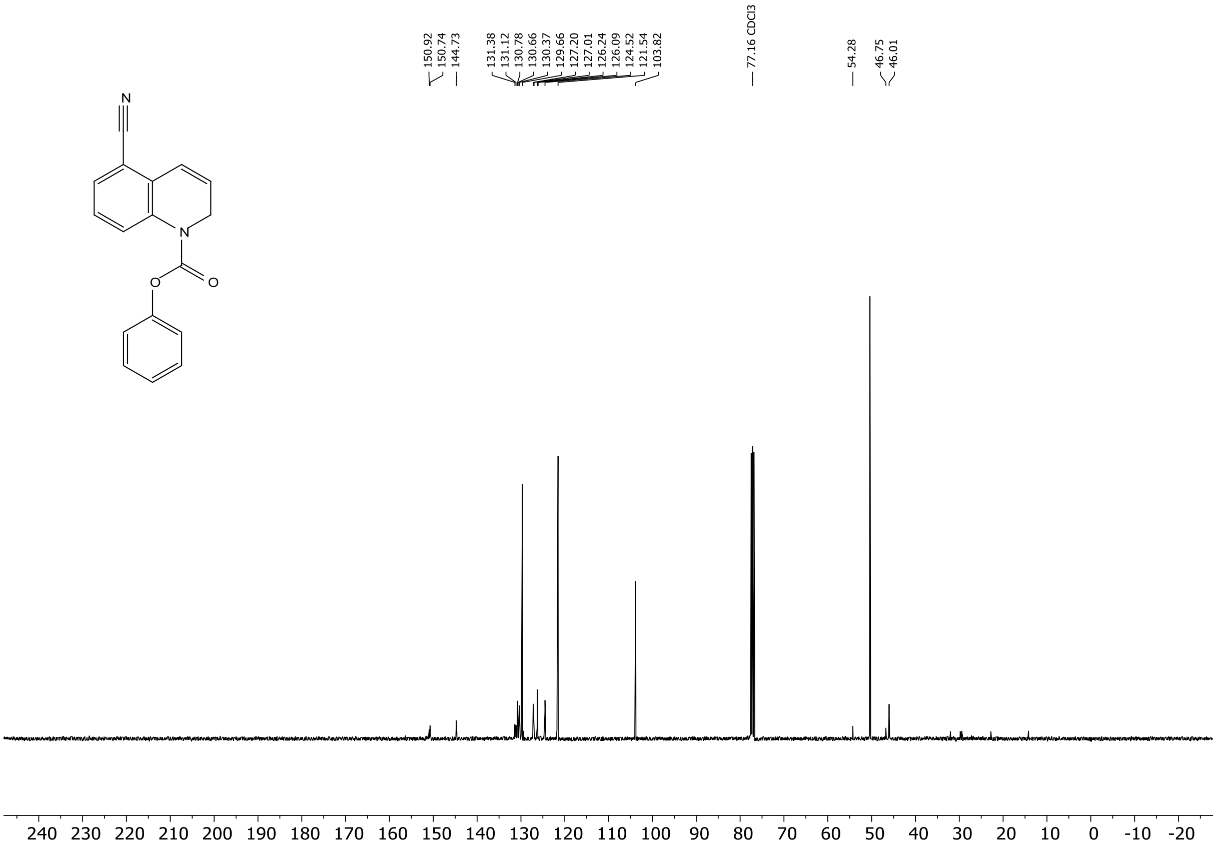

^{1}H NMR (400 MHz, chloroform-d_3) – compound **33**

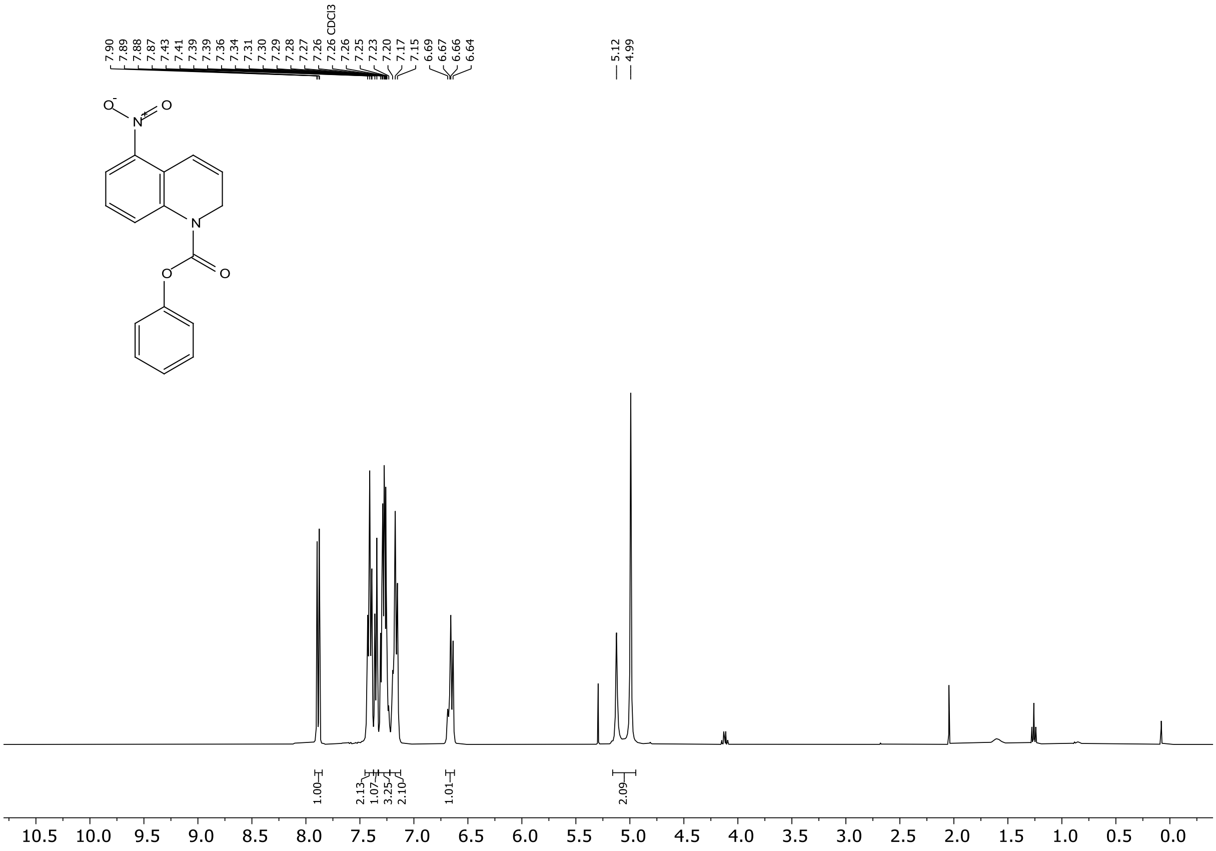

^{13}C{^{1}H} NMR (101 MHz, chloroform-d_3) – compound **33**

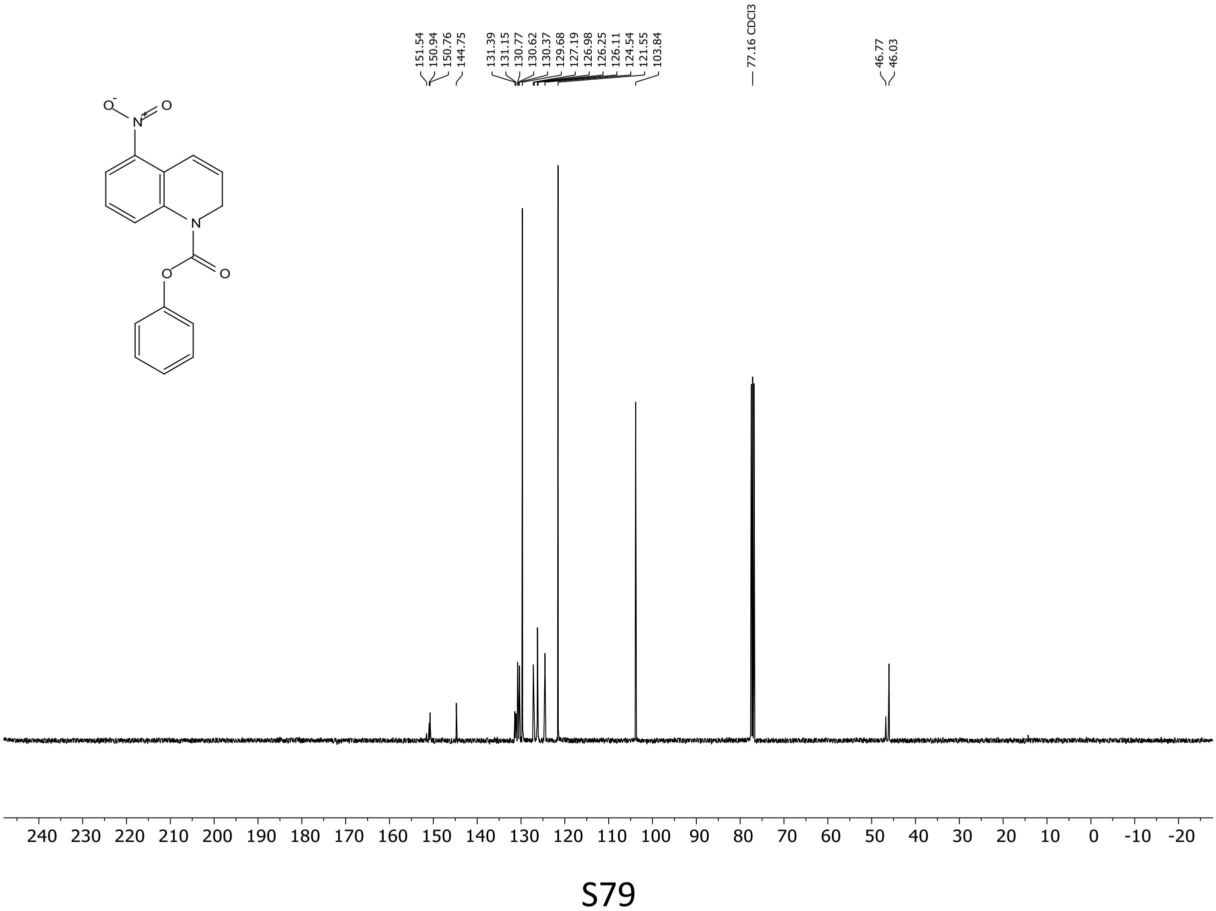

^{1}H NMR (400 MHz, benzene-d_6) – compound **34**

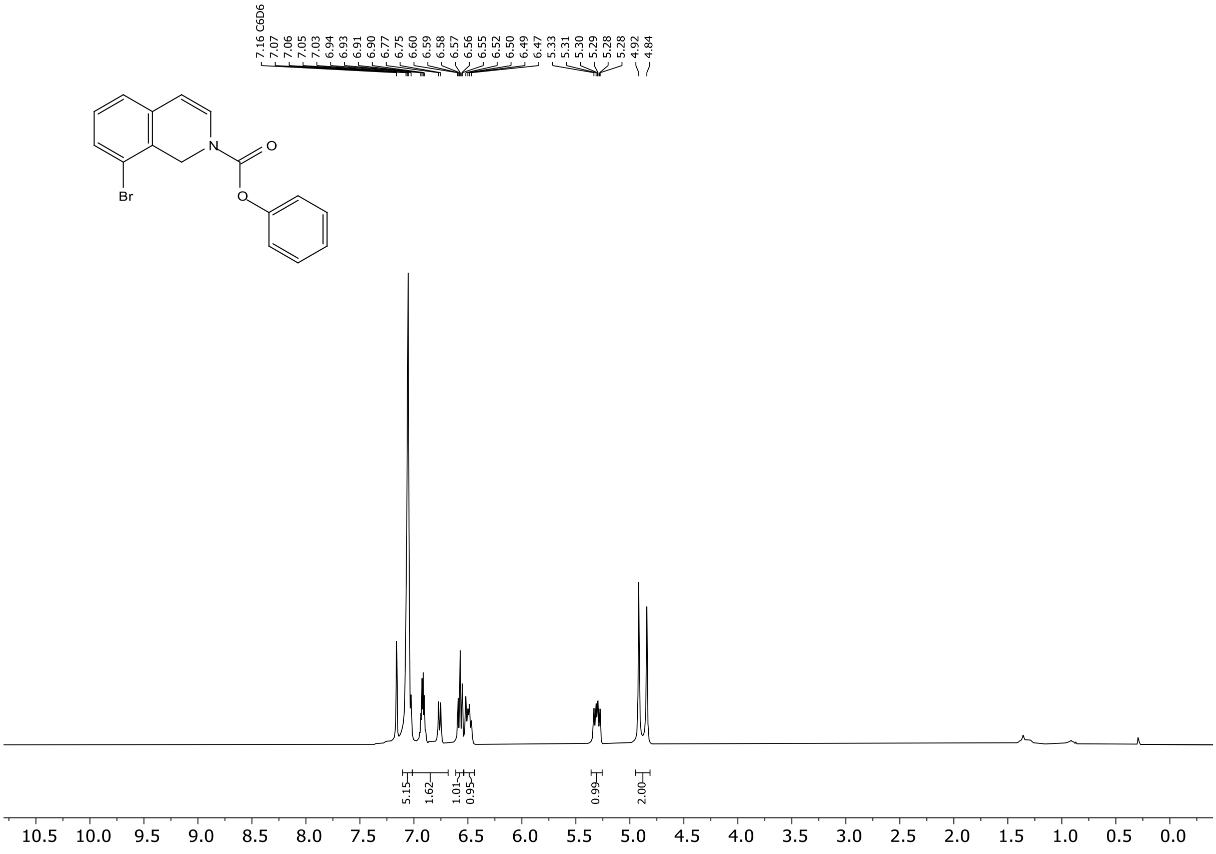

^{13}C{^{1}H} NMR (101 MHz, benzene-d_6) – compound **34**

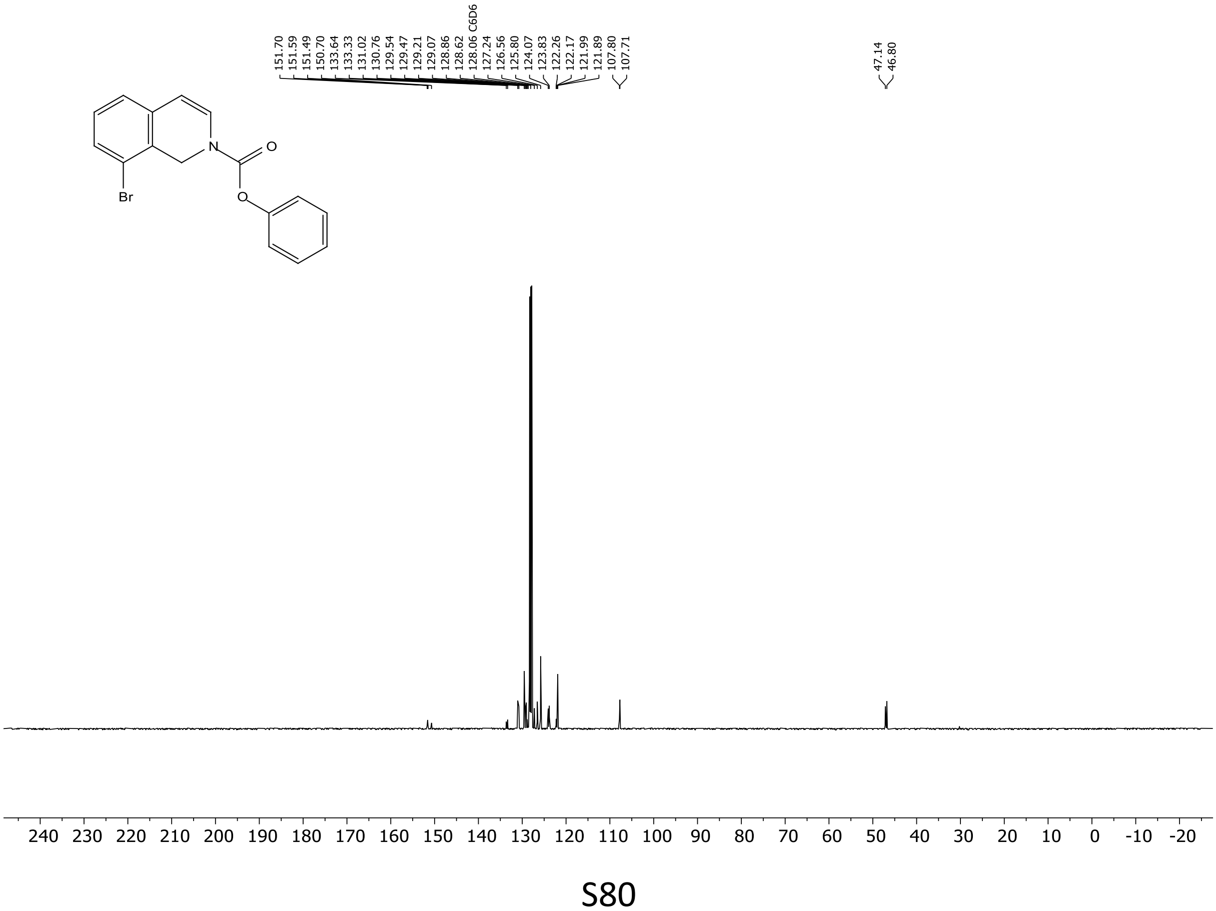

^{1}H NMR (400 MHz, benzene-d_6) – compound **35**

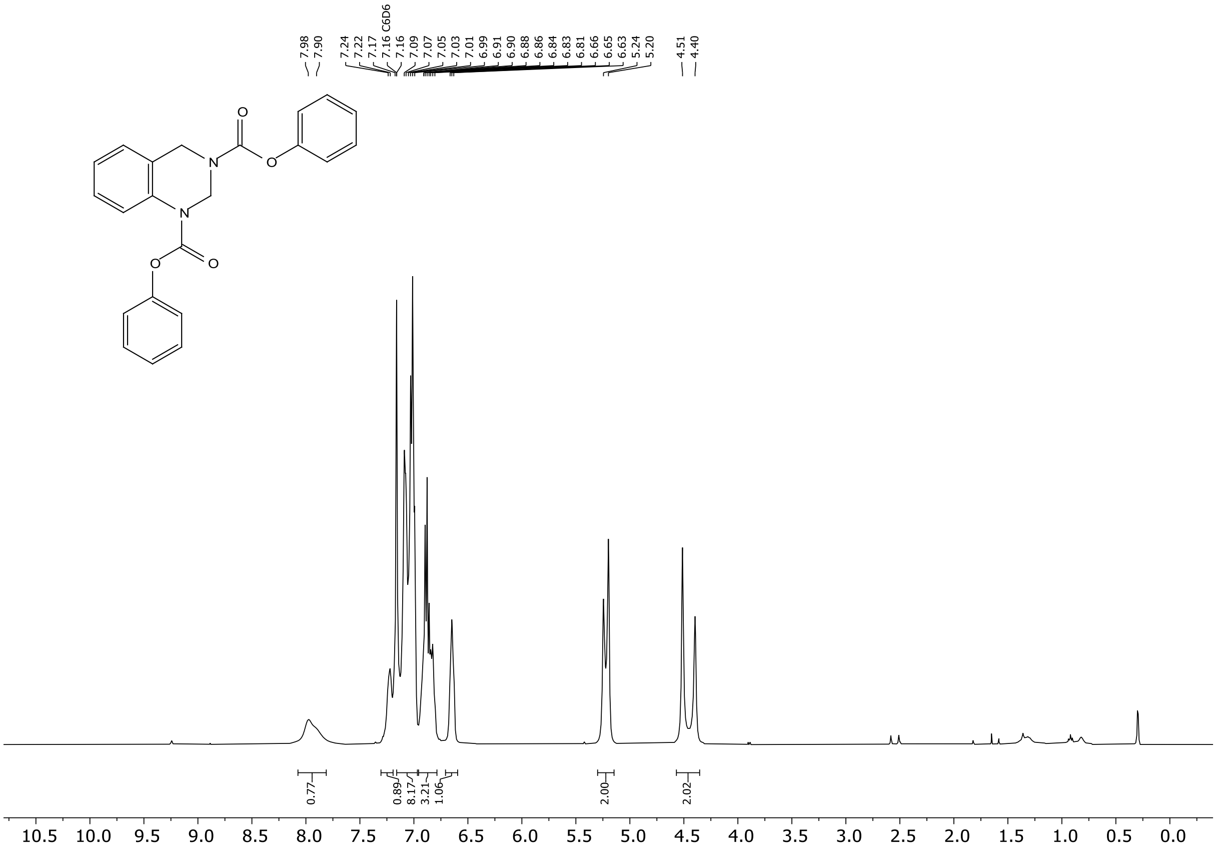

^{13}C{^{1}H} NMR (101 MHz, benzene-d_6) – compound **35**

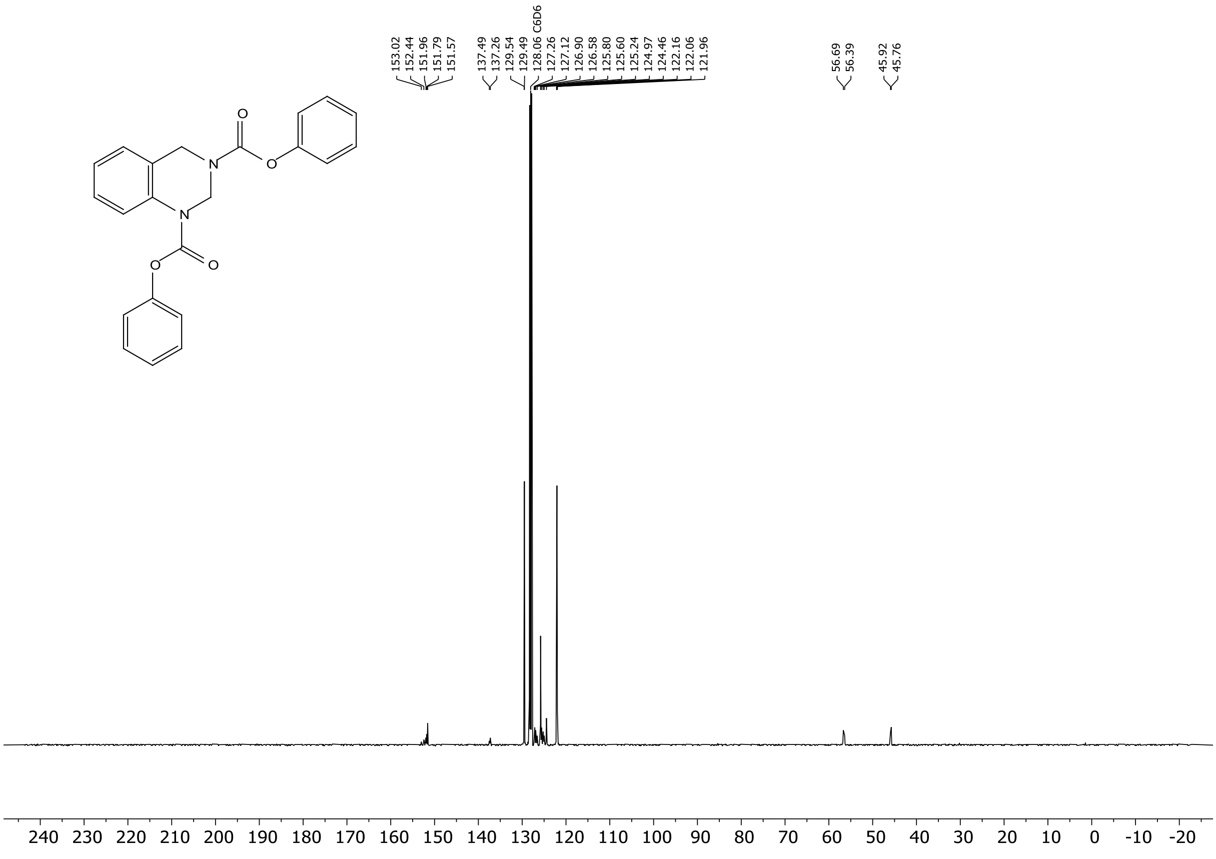

^{1}H NMR (400 MHz, benzene-d_6) – compound **36**

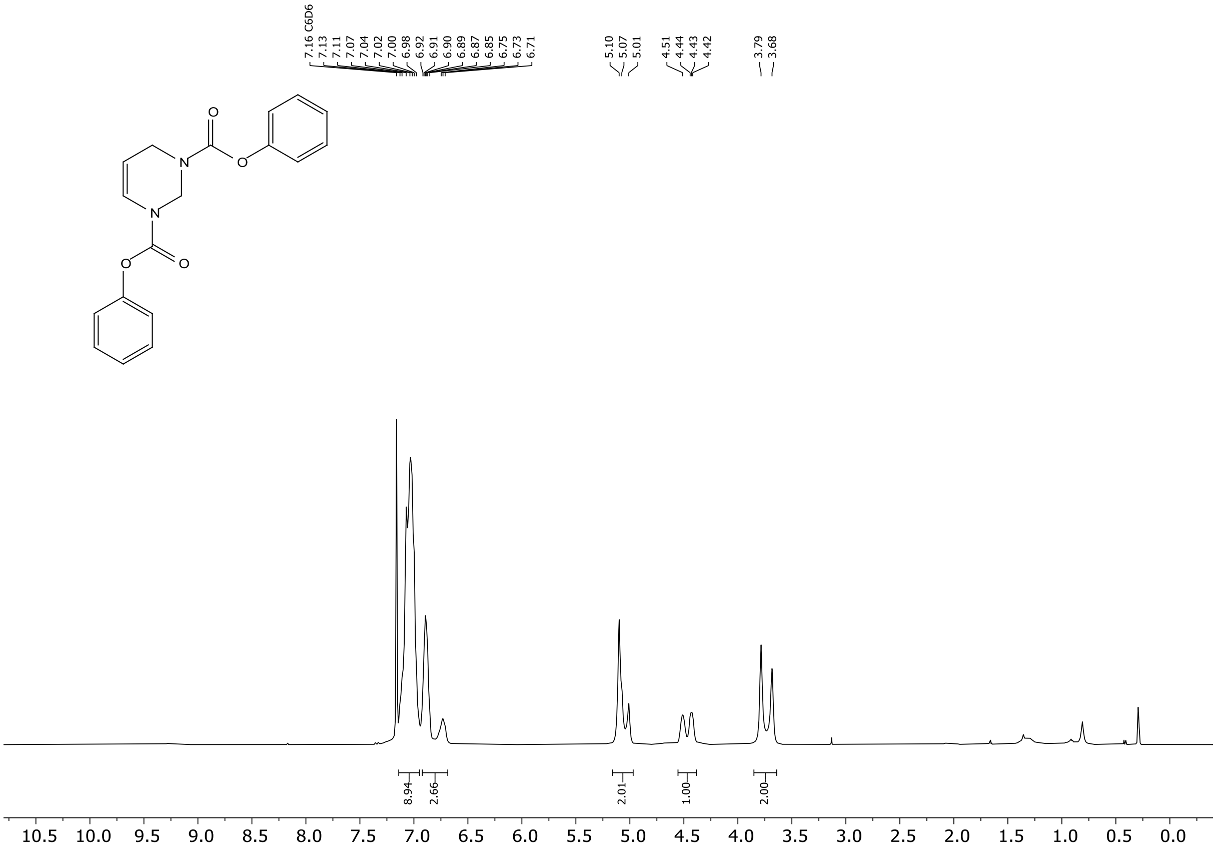

^{13}C{^{1}H} NMR (101 MHz, benzene-d_6) – compound **36**

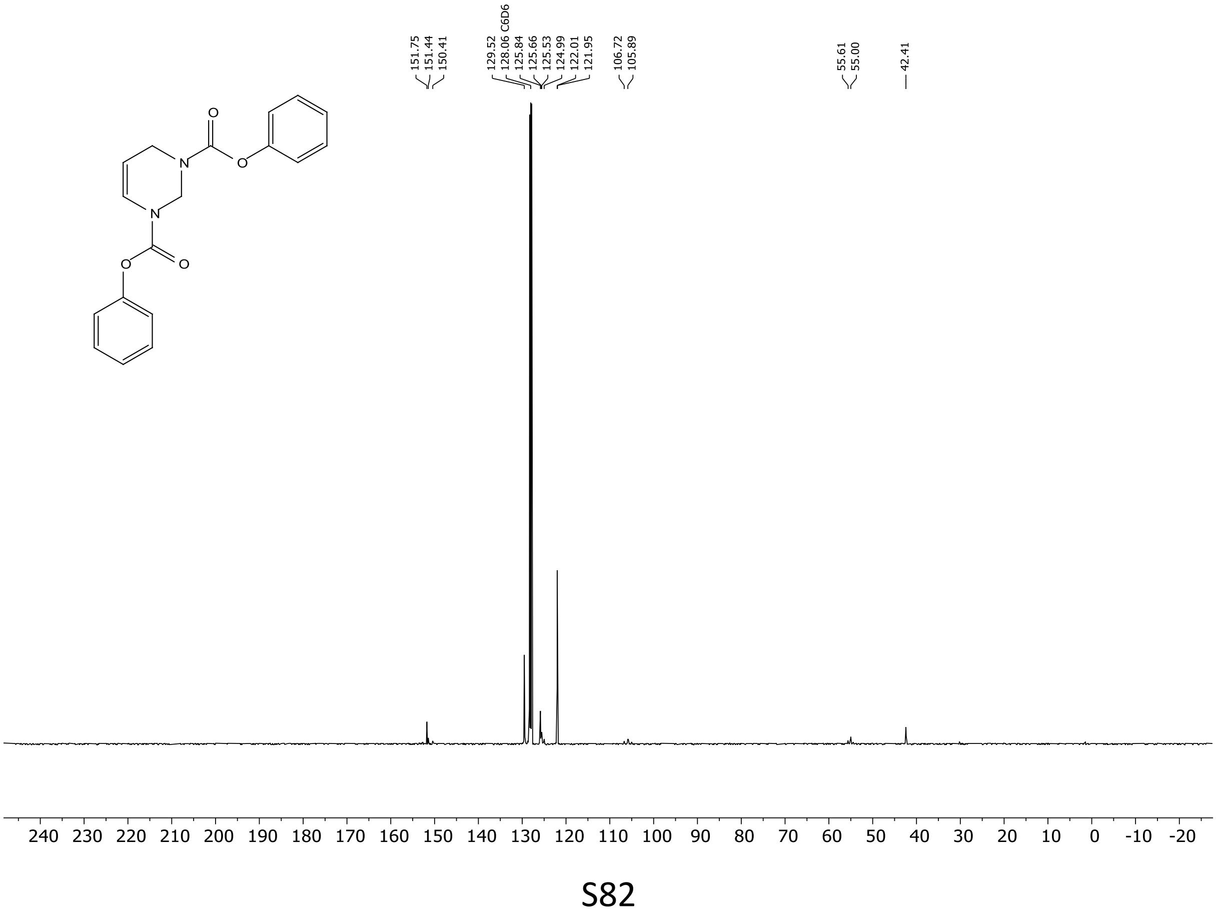

^{1}**H NMR** (400 MHz, benzene-d_6) – compound **37**

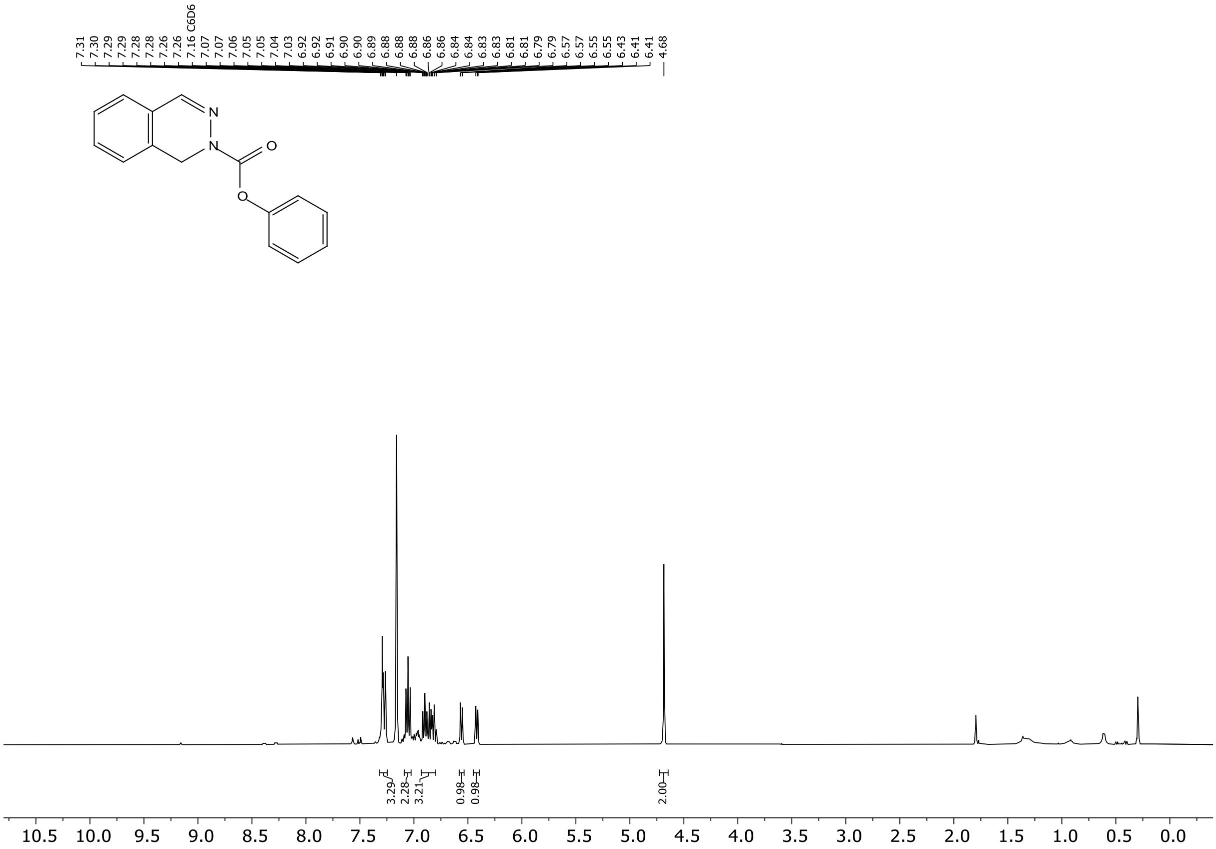

^{13}**C{^{1}H} NMR** (101 MHz, benzene-d_6) – compound **37**

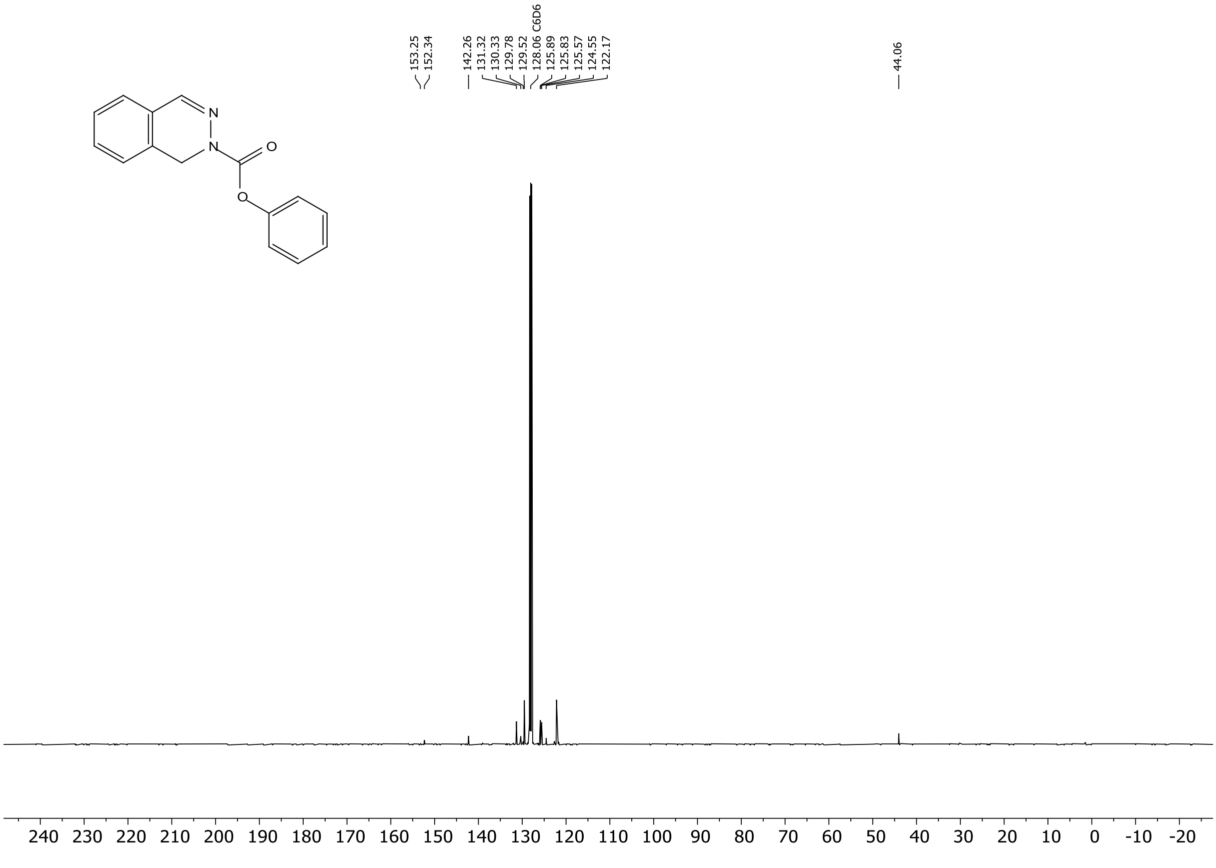

^{1}H NMR (400 MHz, chlorofrom-d_3) – compound **38**

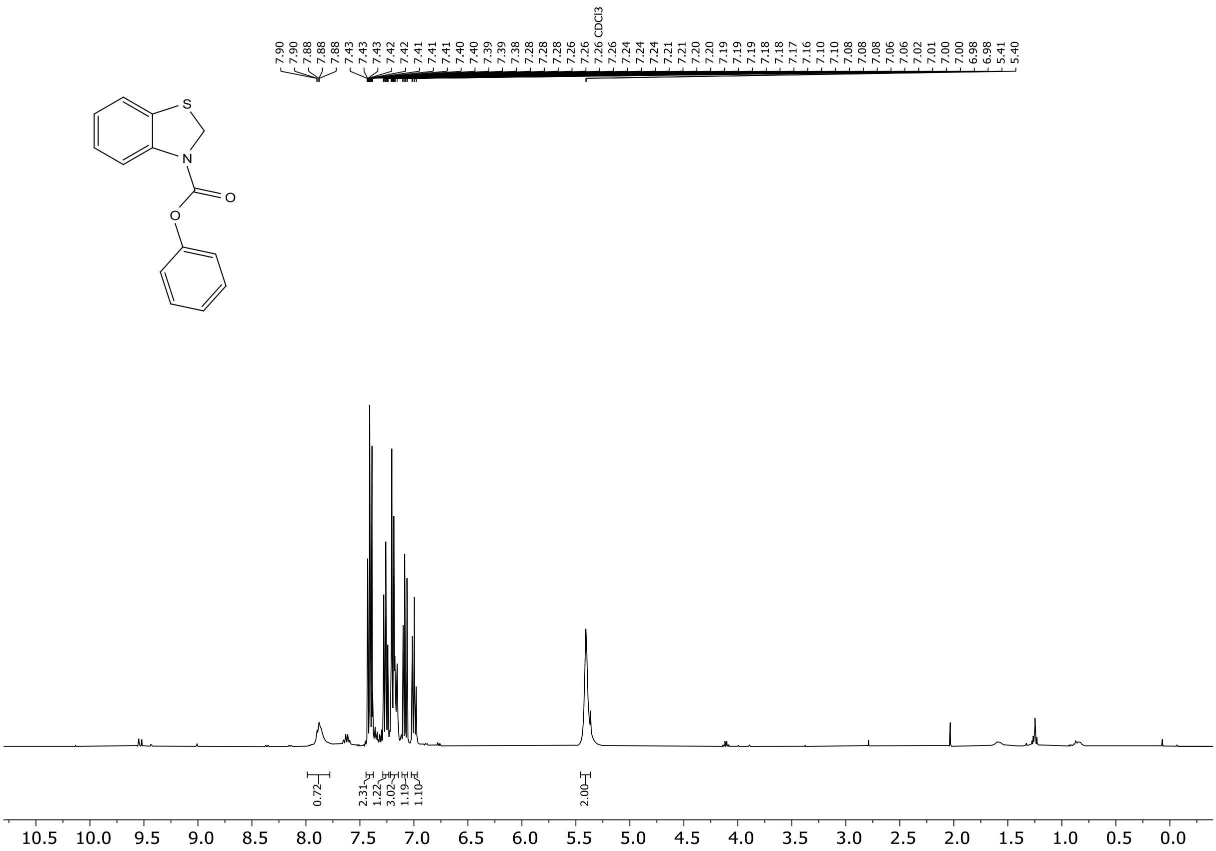

^{13}C{^{1}H} NMR (101 MHz, chlorofrom-d_3) – compound **38**

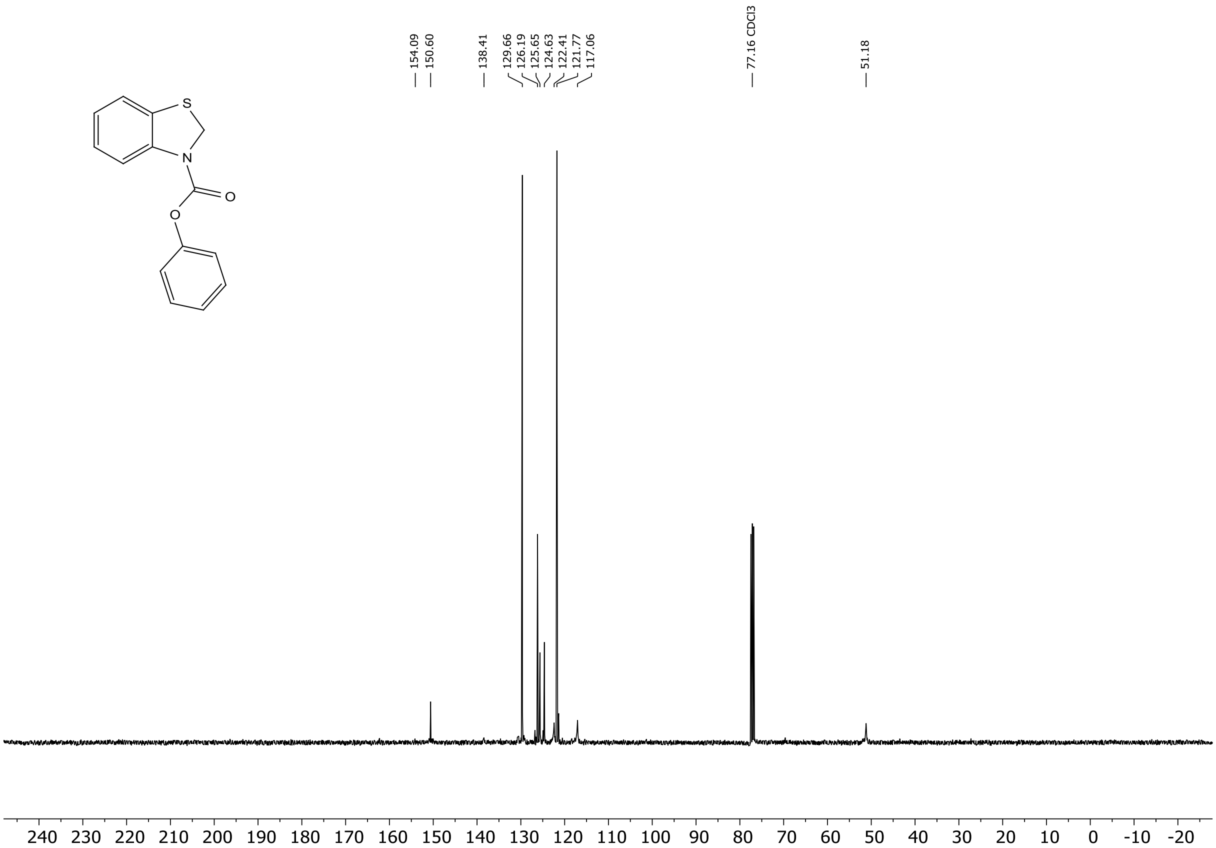

^{1}H NMR (400 MHz, chlorofrom-d_3) – compound **40**

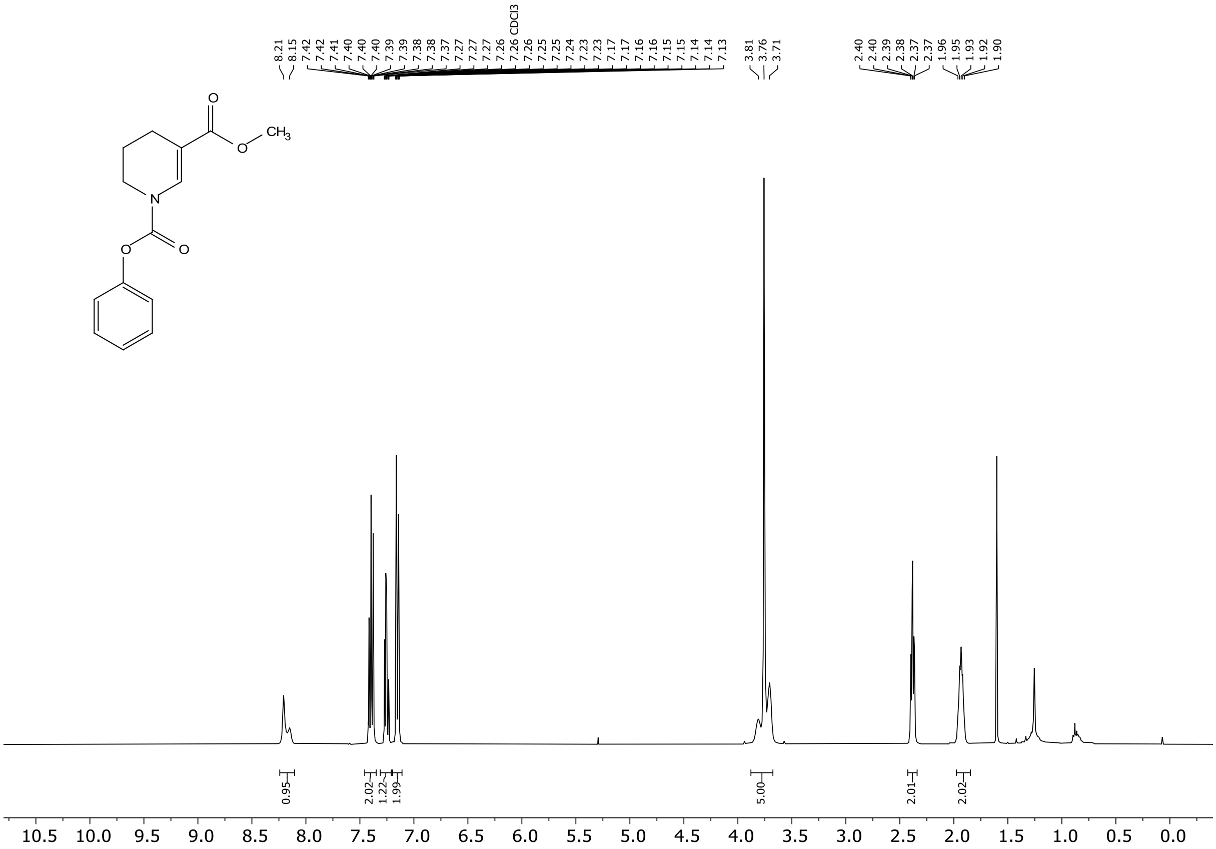

^{13}C{^{1}H} NMR (101 MHz, chlorofrom-d_3) – compound **40**

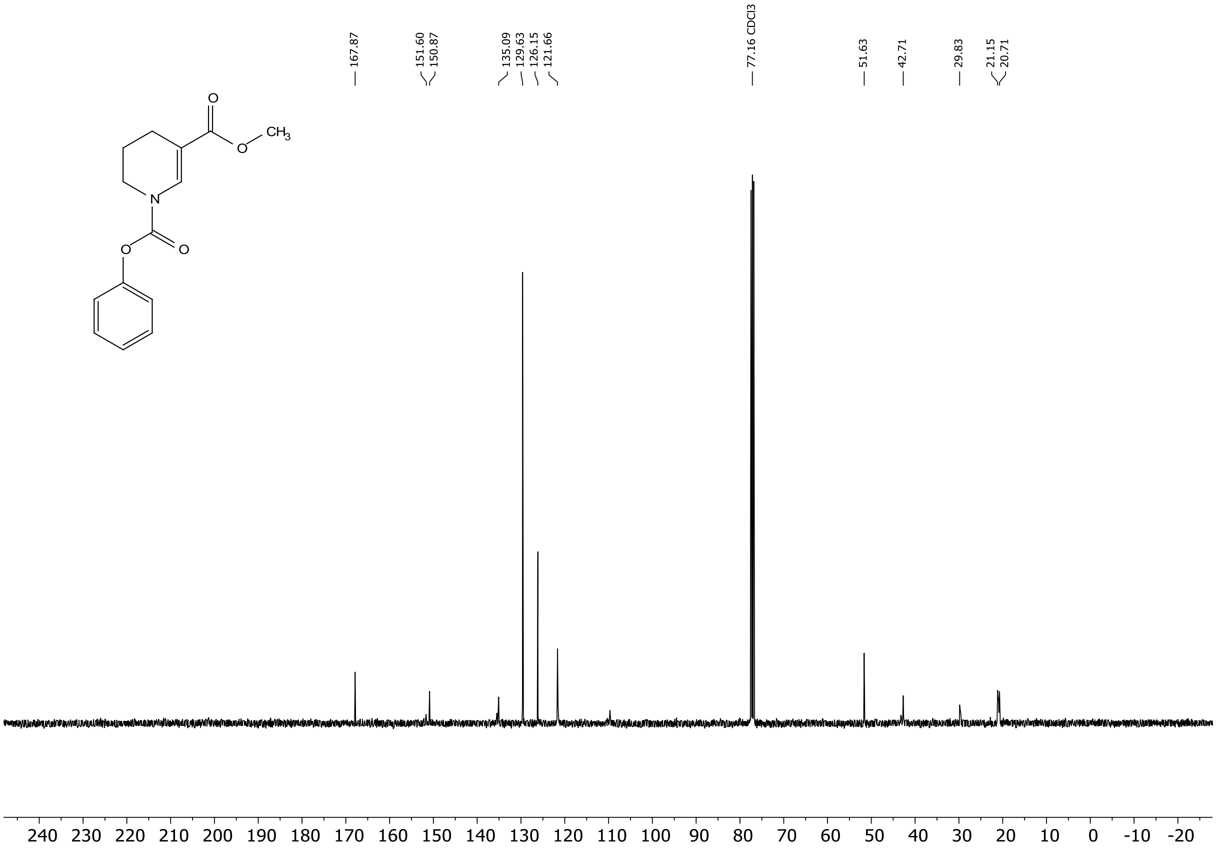

^{1}H NMR (400 MHz, chlorofrom-d_3) – compound **41**

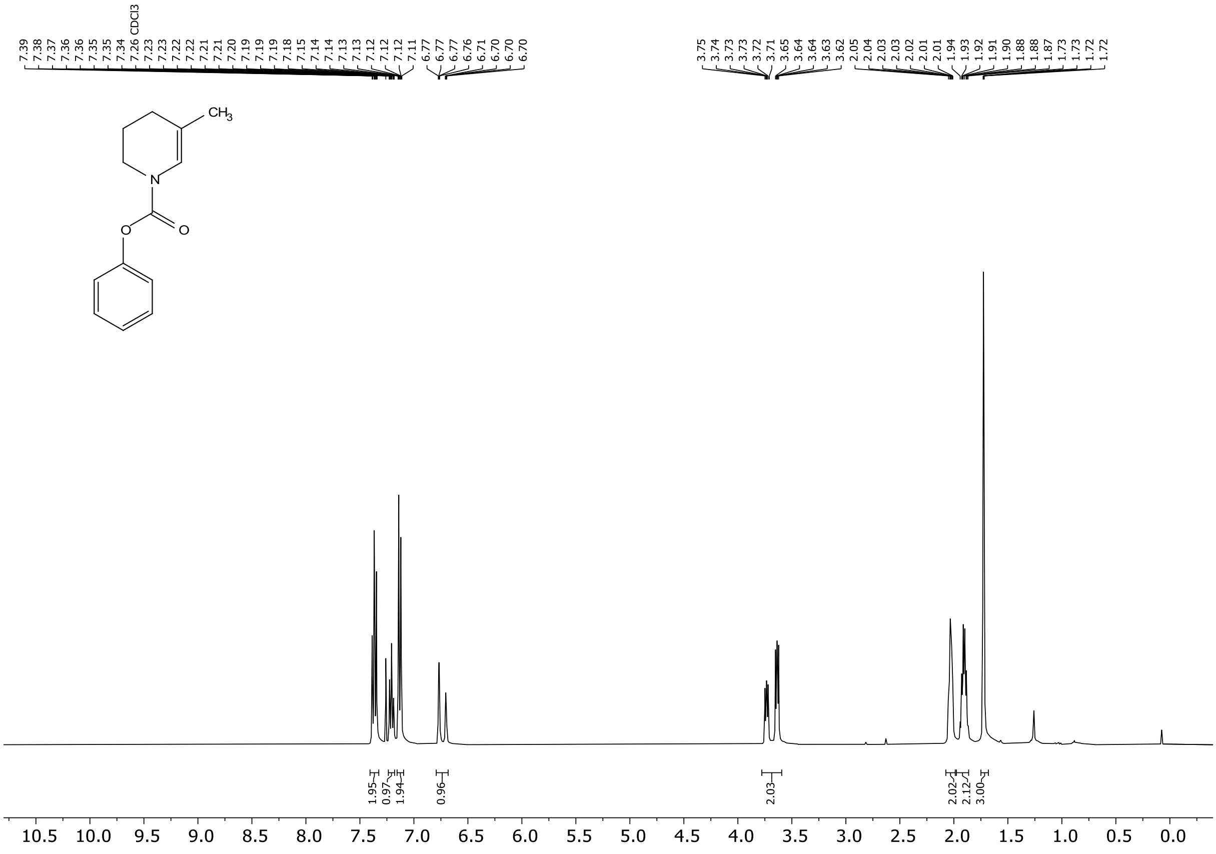

^{13}C{^{1}H} NMR (101 MHz, chlorofrom-d_3) – compound **41**

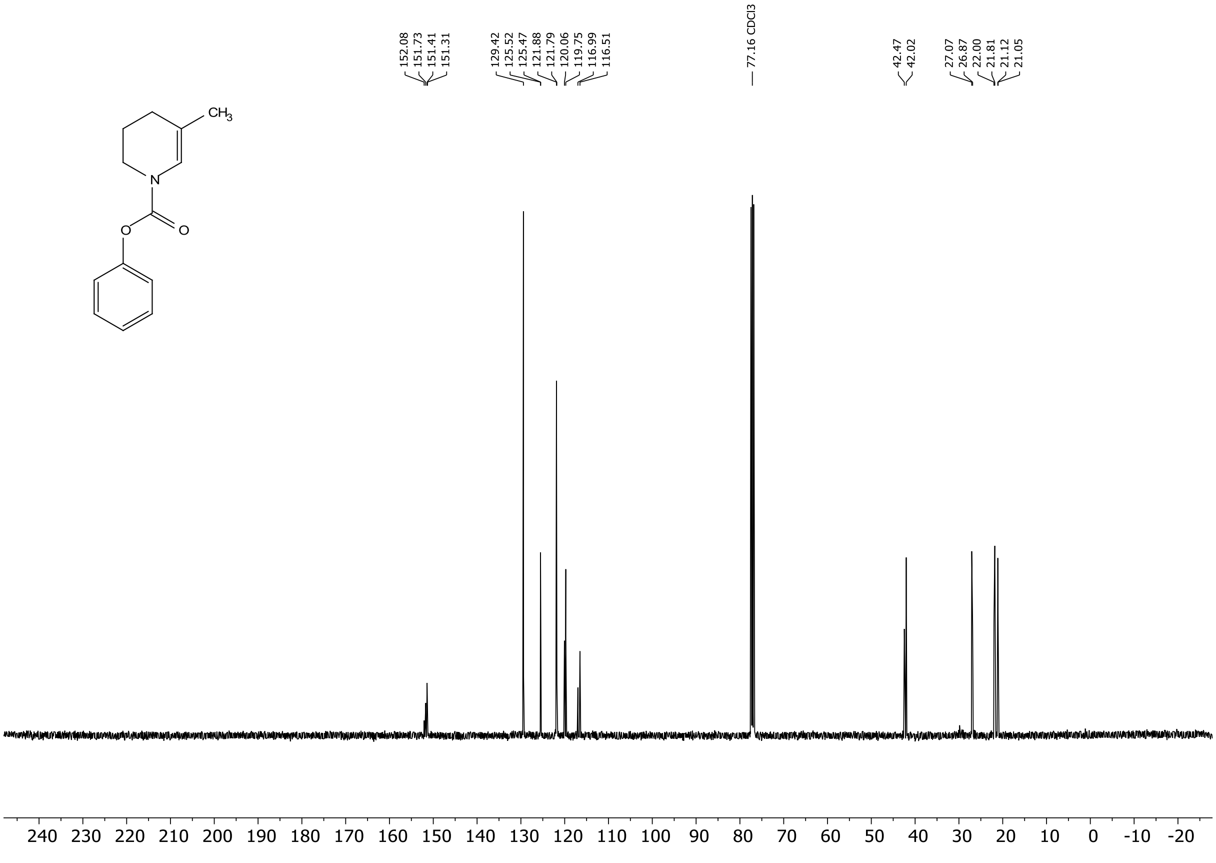

^{1}H NMR (400 MHz, chlorofrom-d_3) – compound **42**

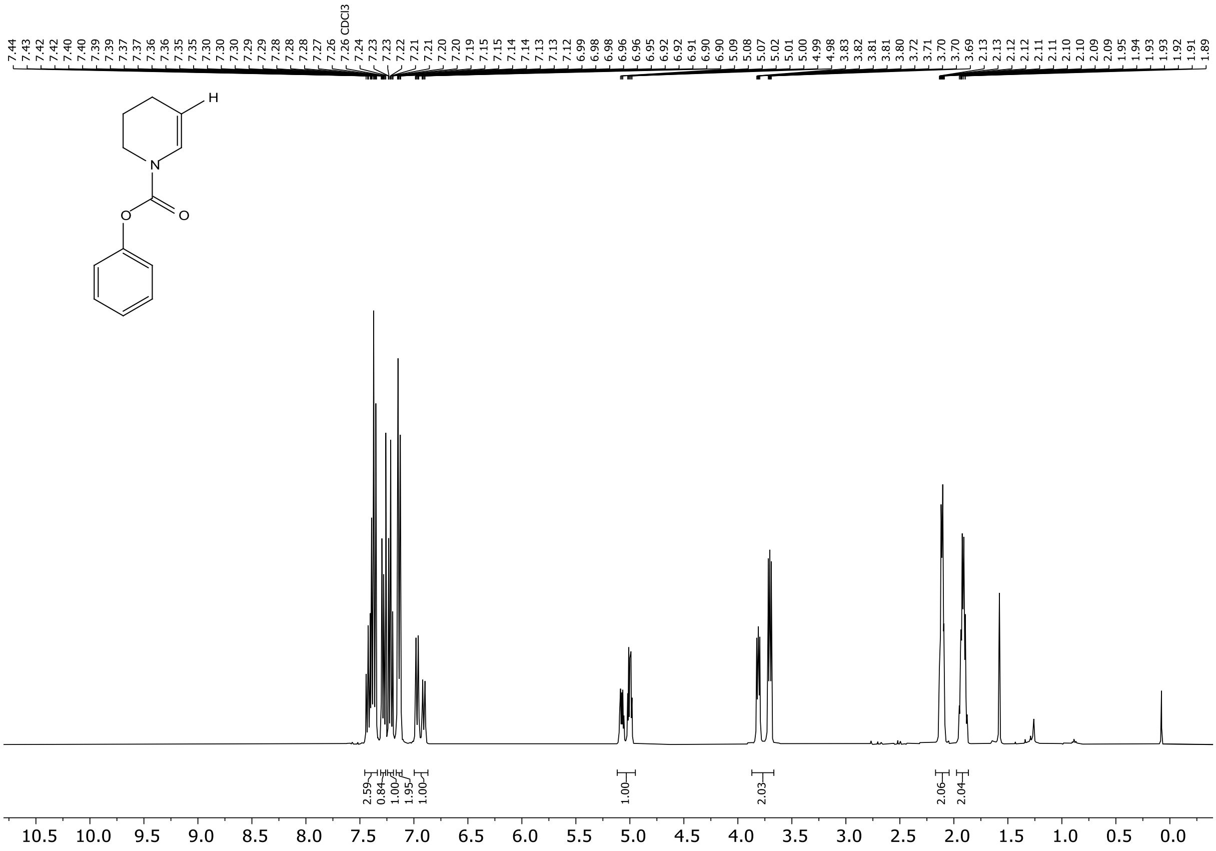

^{13}C{^{1}H} NMR (101 MHz, chlorofrom-d_3) – compound **42**

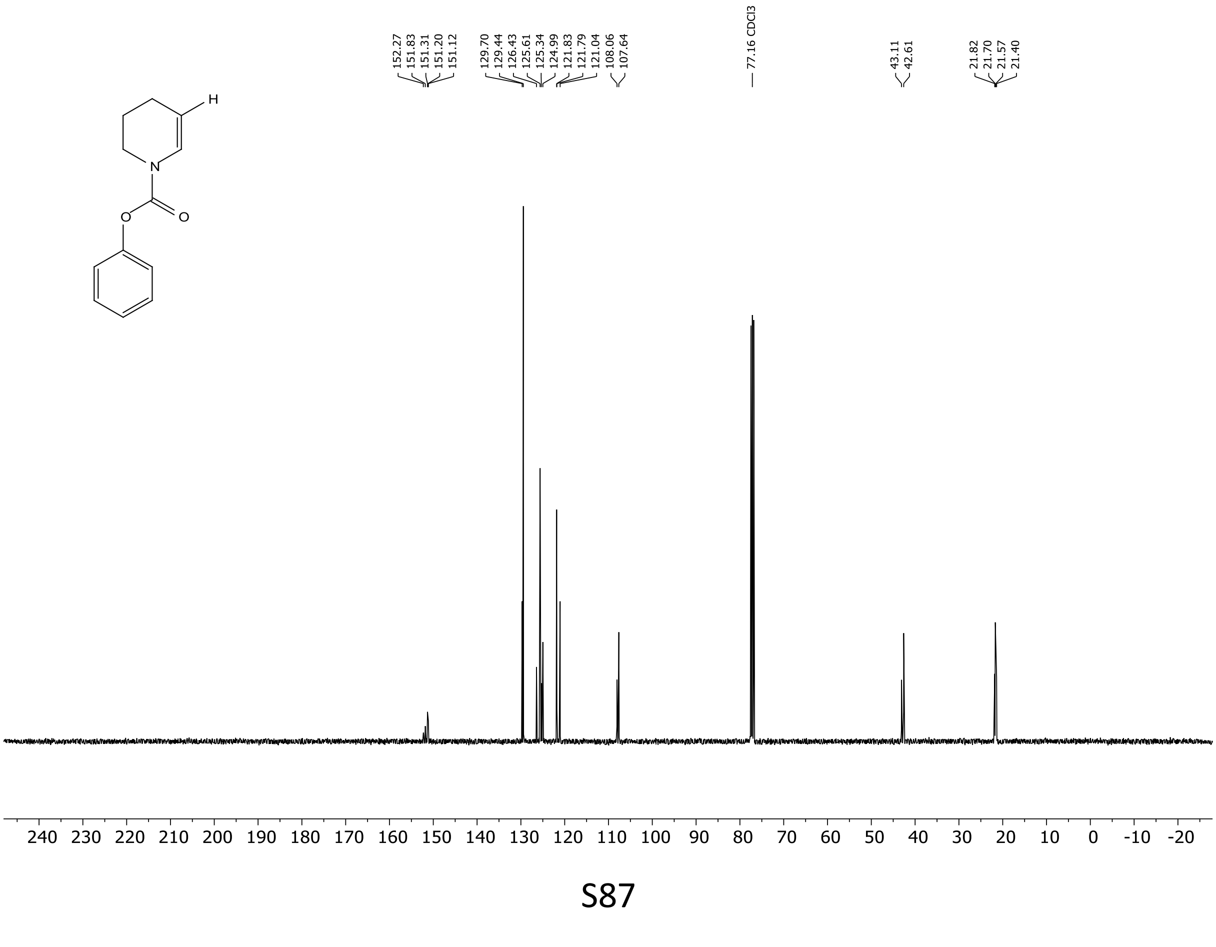

^{1}H NMR (400 MHz, chlorofrom-d_3) – compound **43**

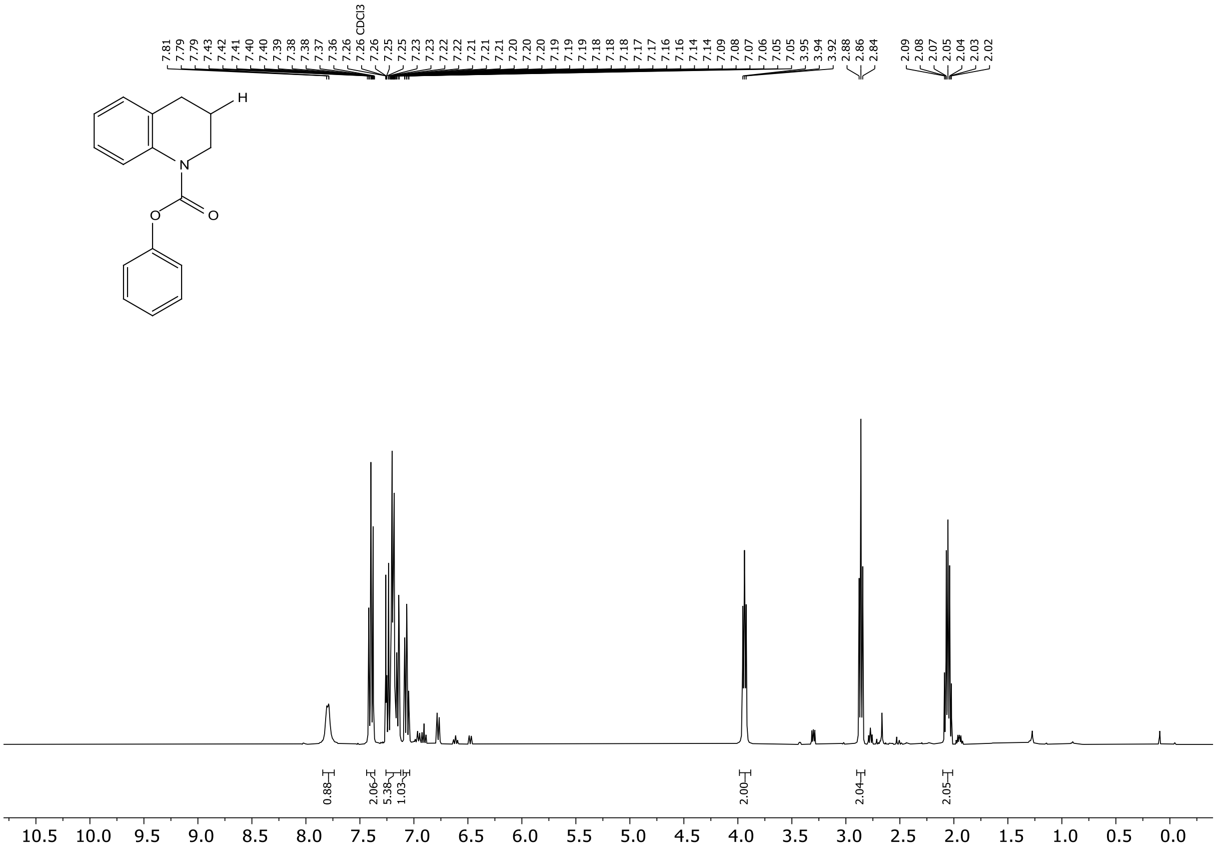

^{13}C{^{1}H} NMR (101 MHz, chlorofrom-d_3) – compound **43**

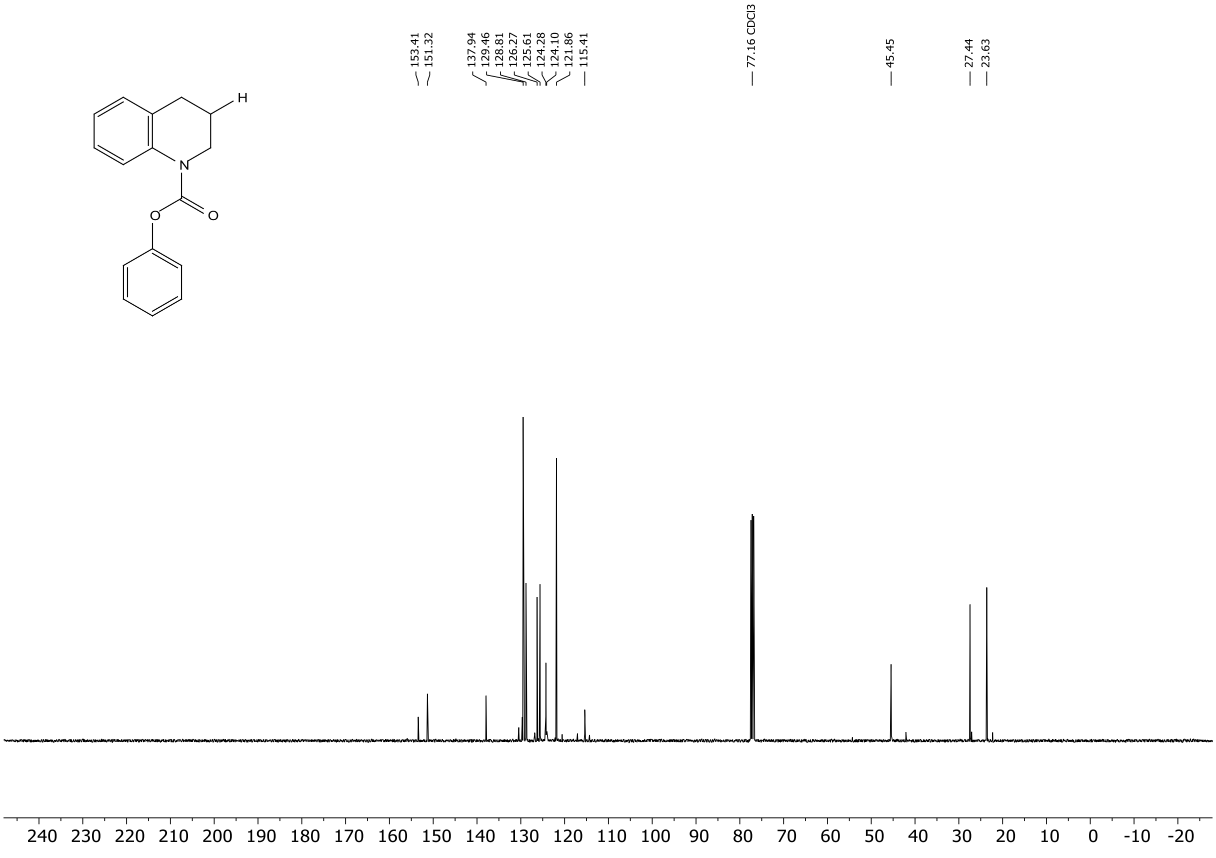

^{1}**H NMR** (400 MHz, chlorofrom-d_3) – compound **44**

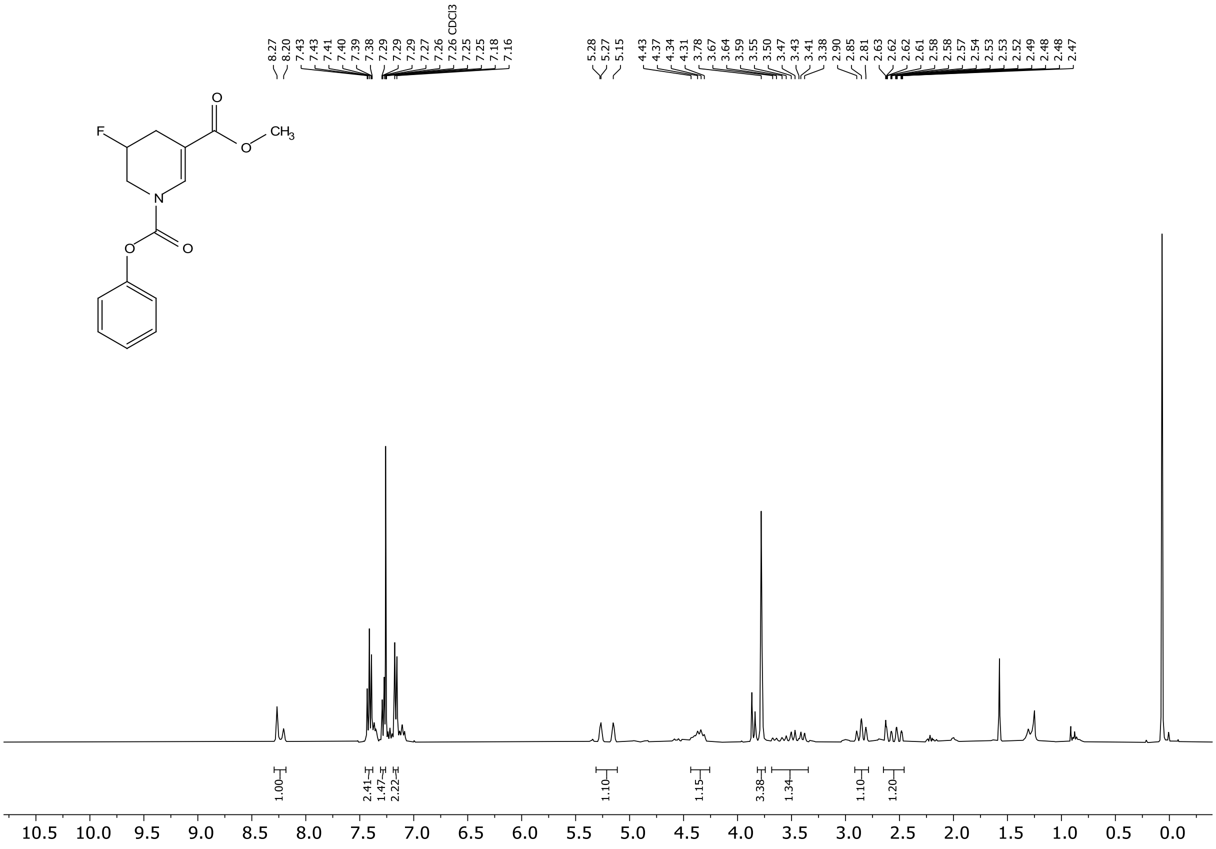

^{13}**C{^{1}H} NMR** (101 MHz, chlorofrom-d_3) – compound **44**

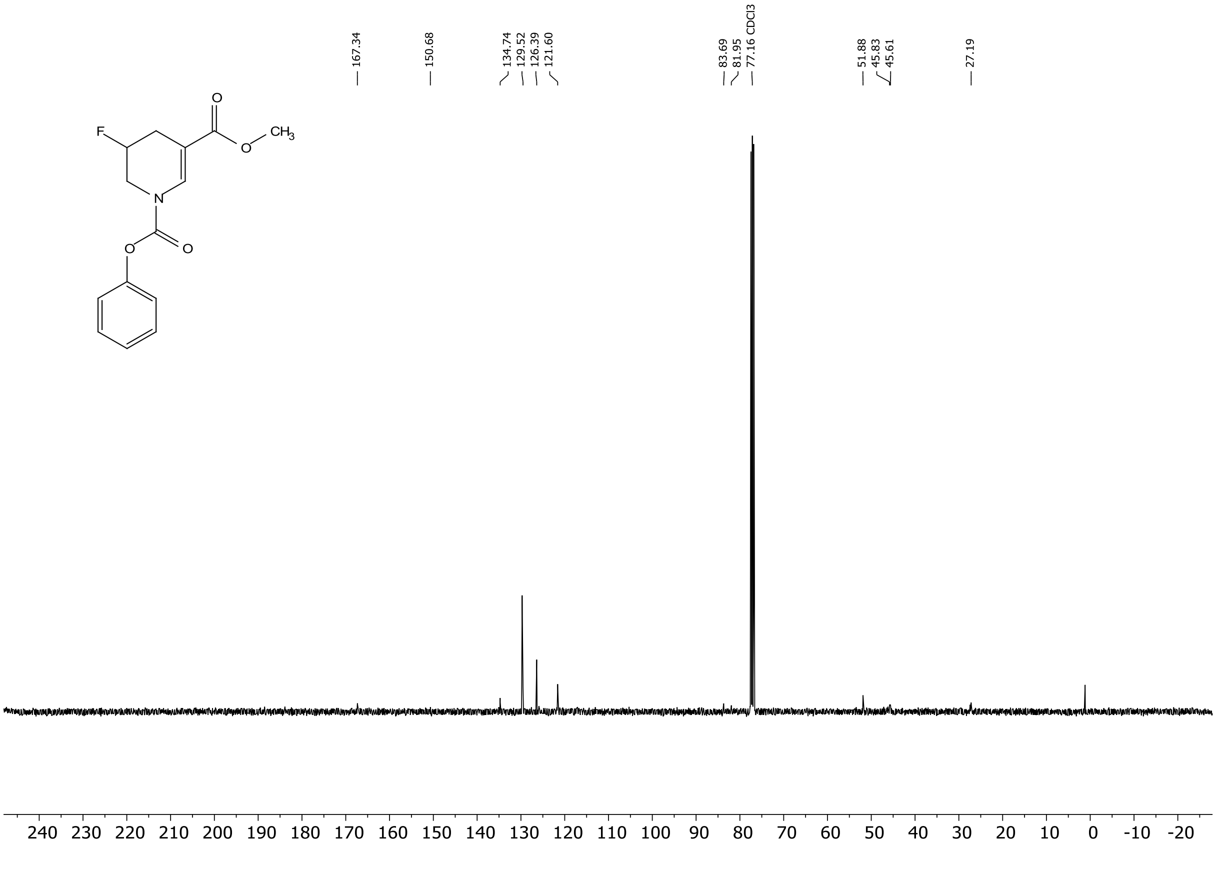

^{19}F{^{1}H} NMR (376 MHz, chloroform-*d$_3$*) – compound **44**

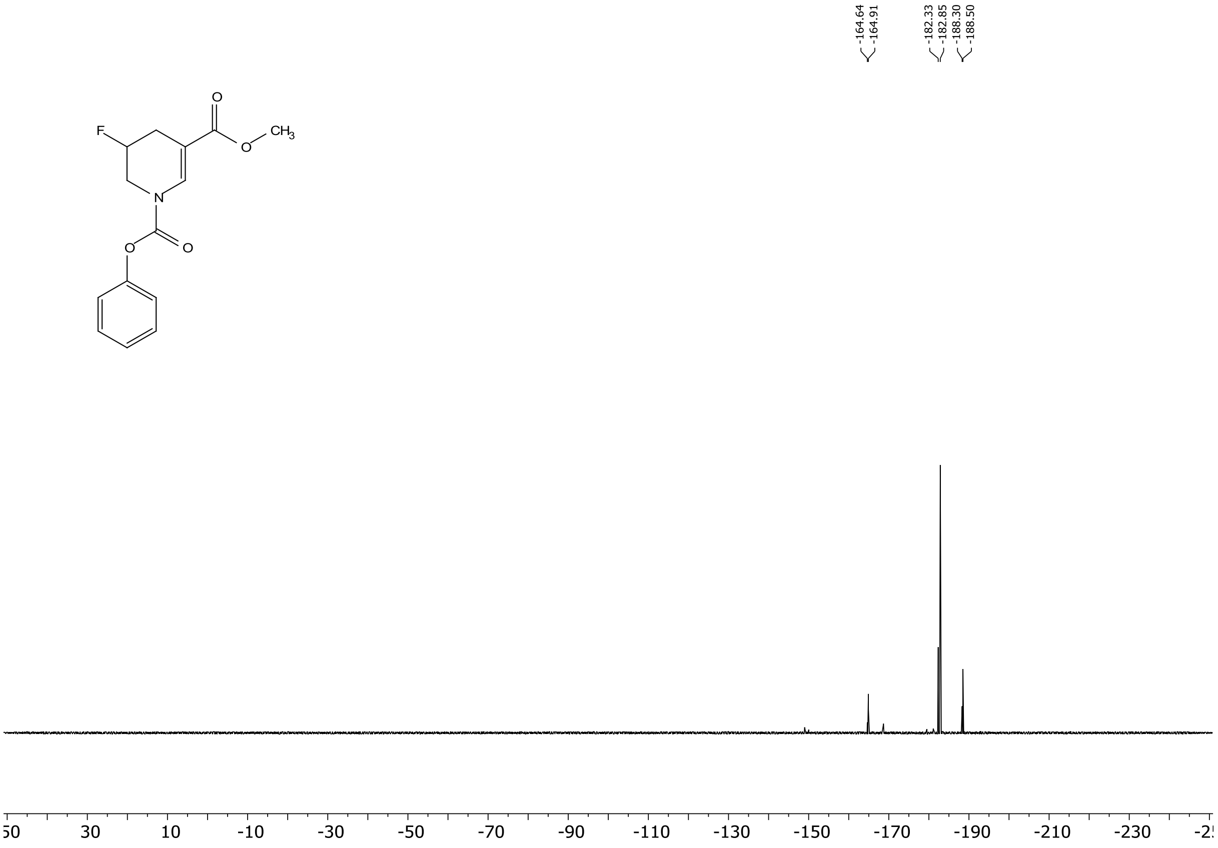

^{1}H NMR (400 MHz, chlorofrom-*d*$_3$) – compound **45**

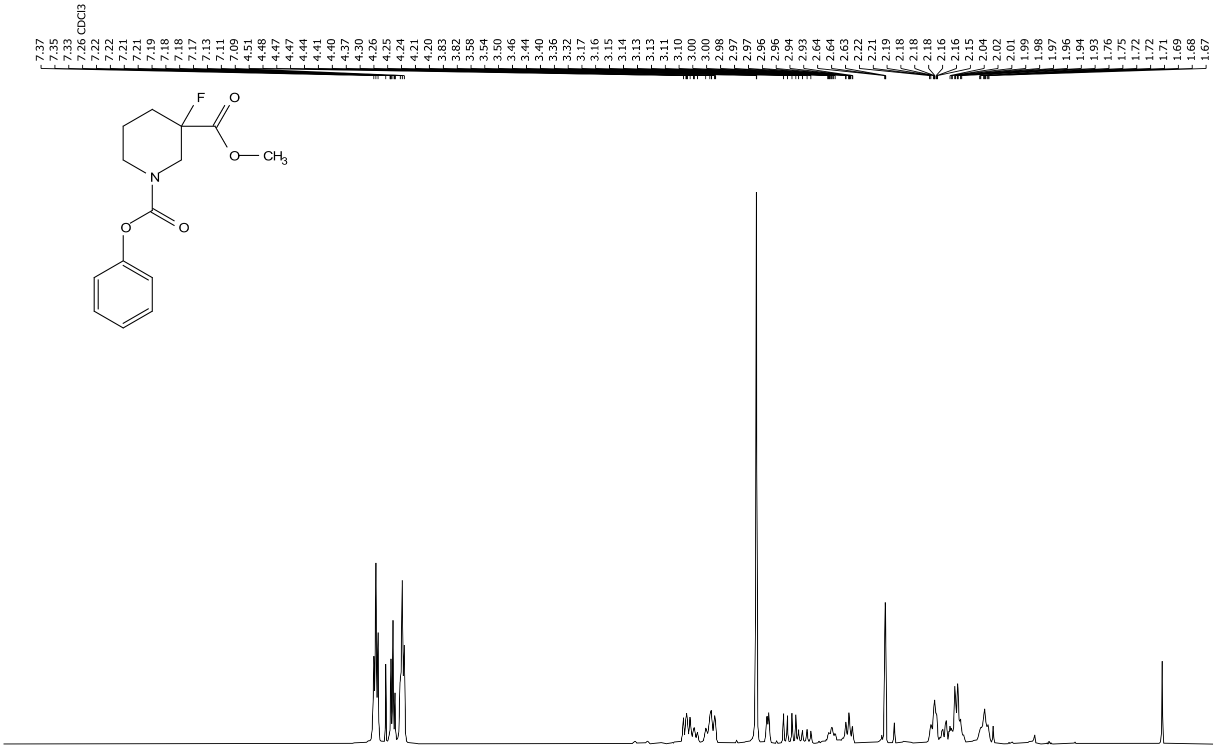

$^{13}C\{^1H\}$ NMR (101 MHz, chlorofrom-d_3) – compound **45**

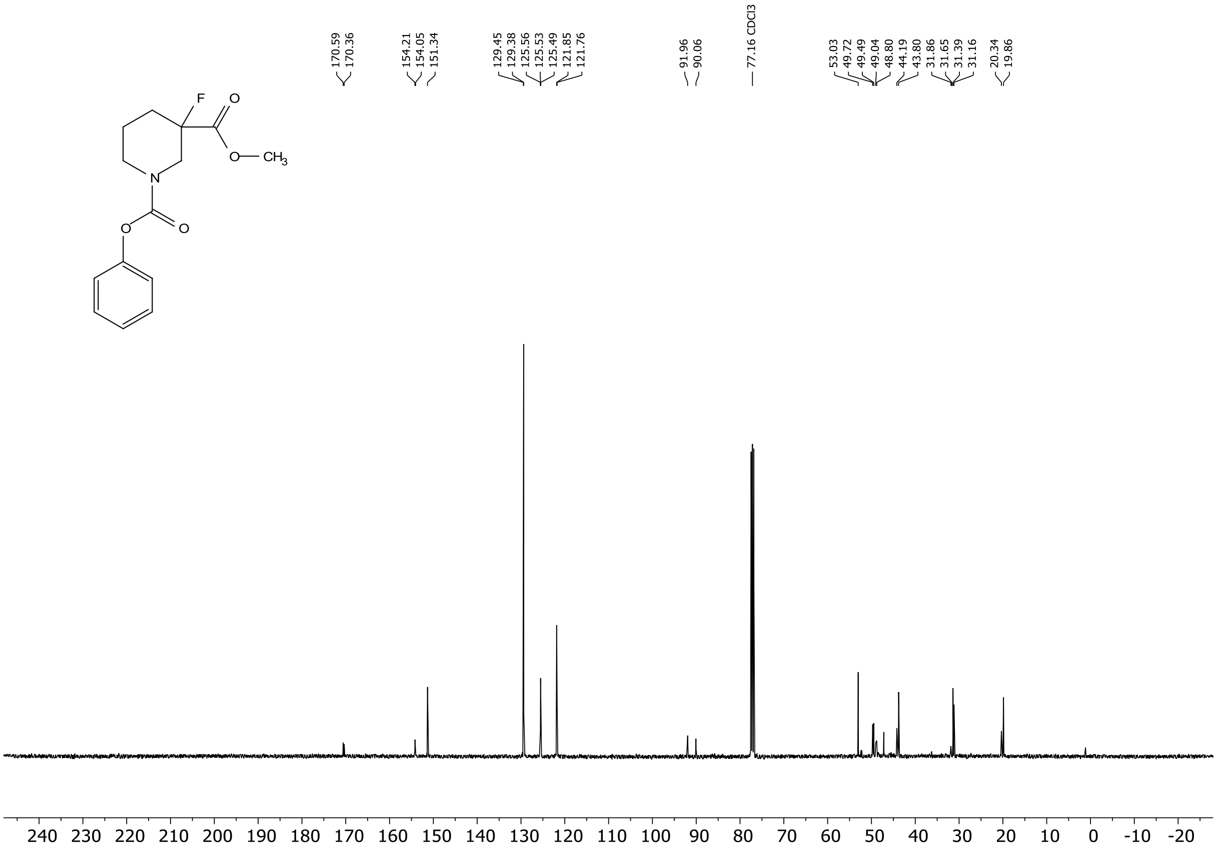

$^{19}F\{^1H\}$ NMR (376 MHz, chloroform-d_3) – compound **45**

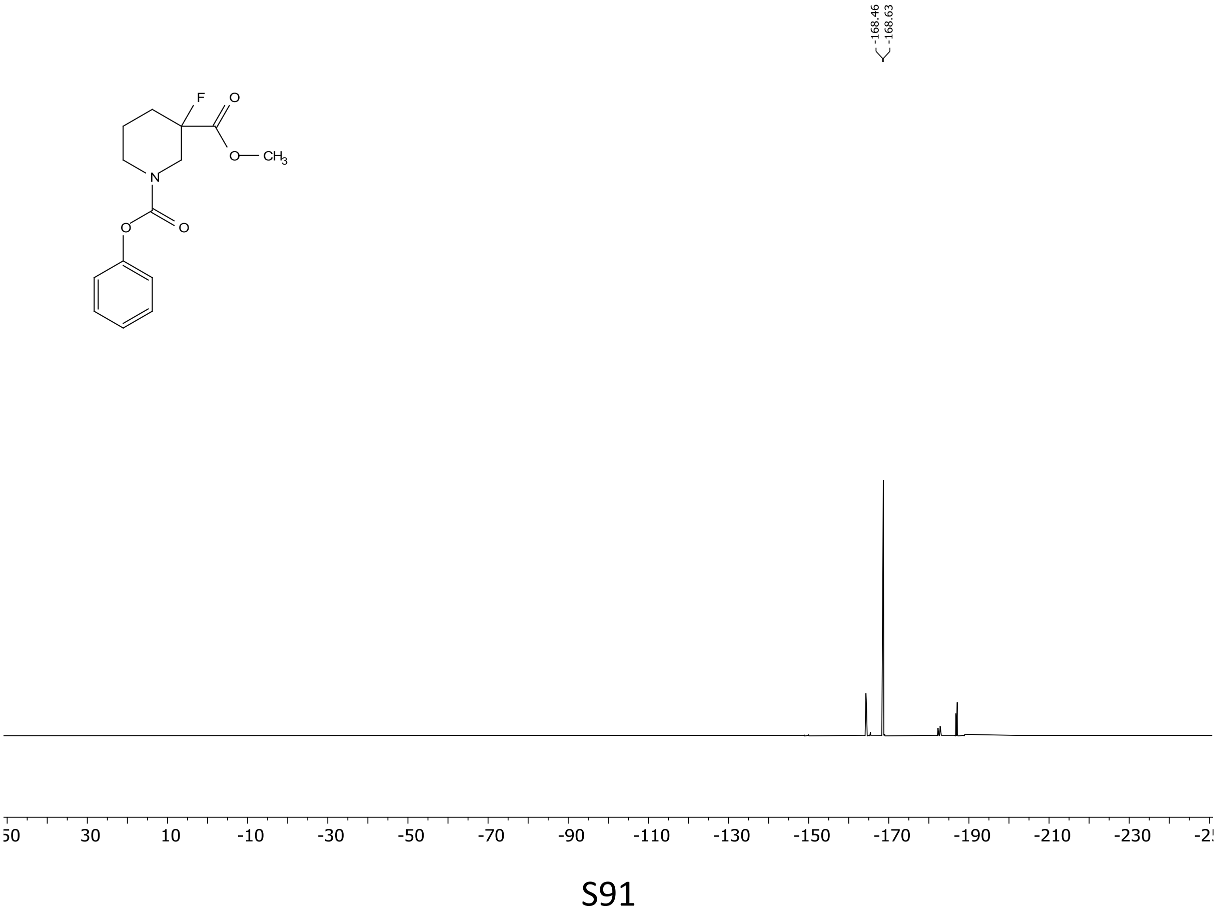

^{1}H NMR (500 MHz, toluene-d_8) – compound **46**

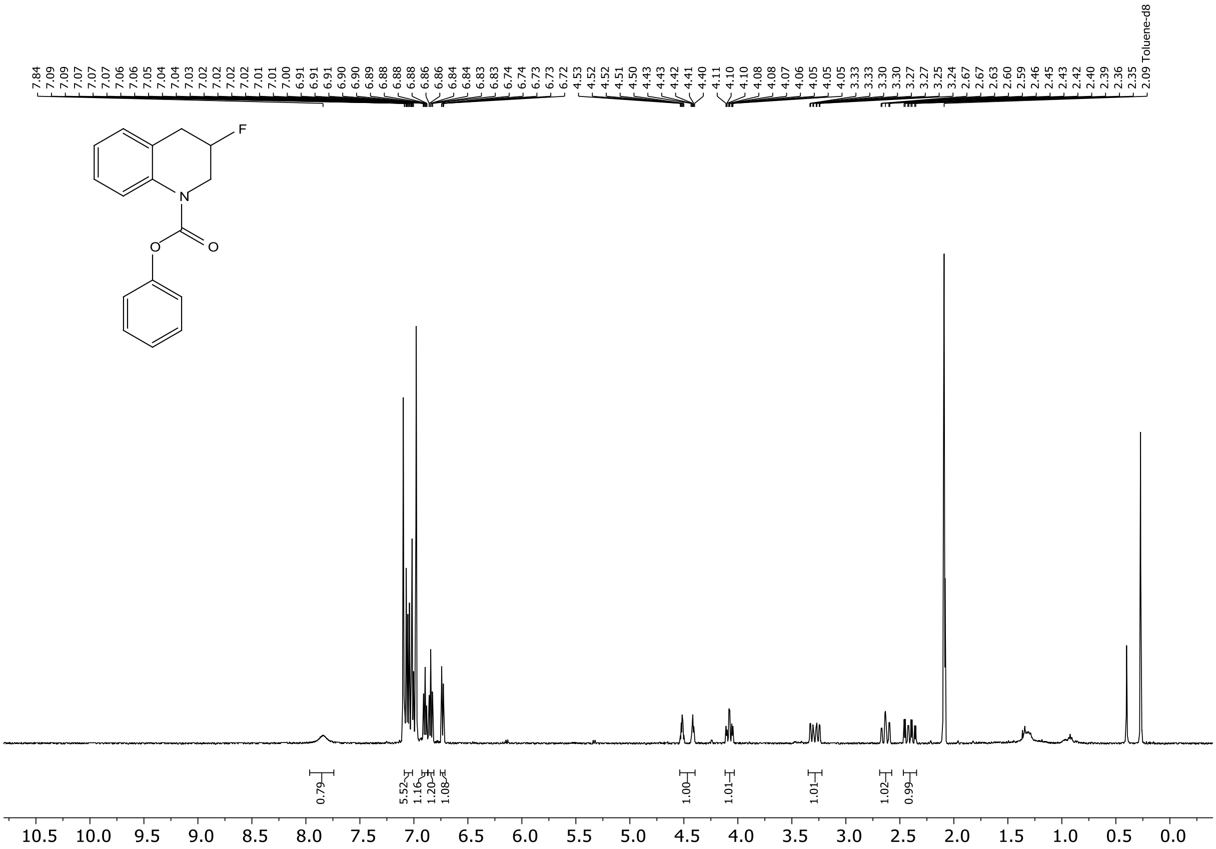

^{13}C{^{1}H} NMR (126 MHz, toluene-d_8) – compound **46**

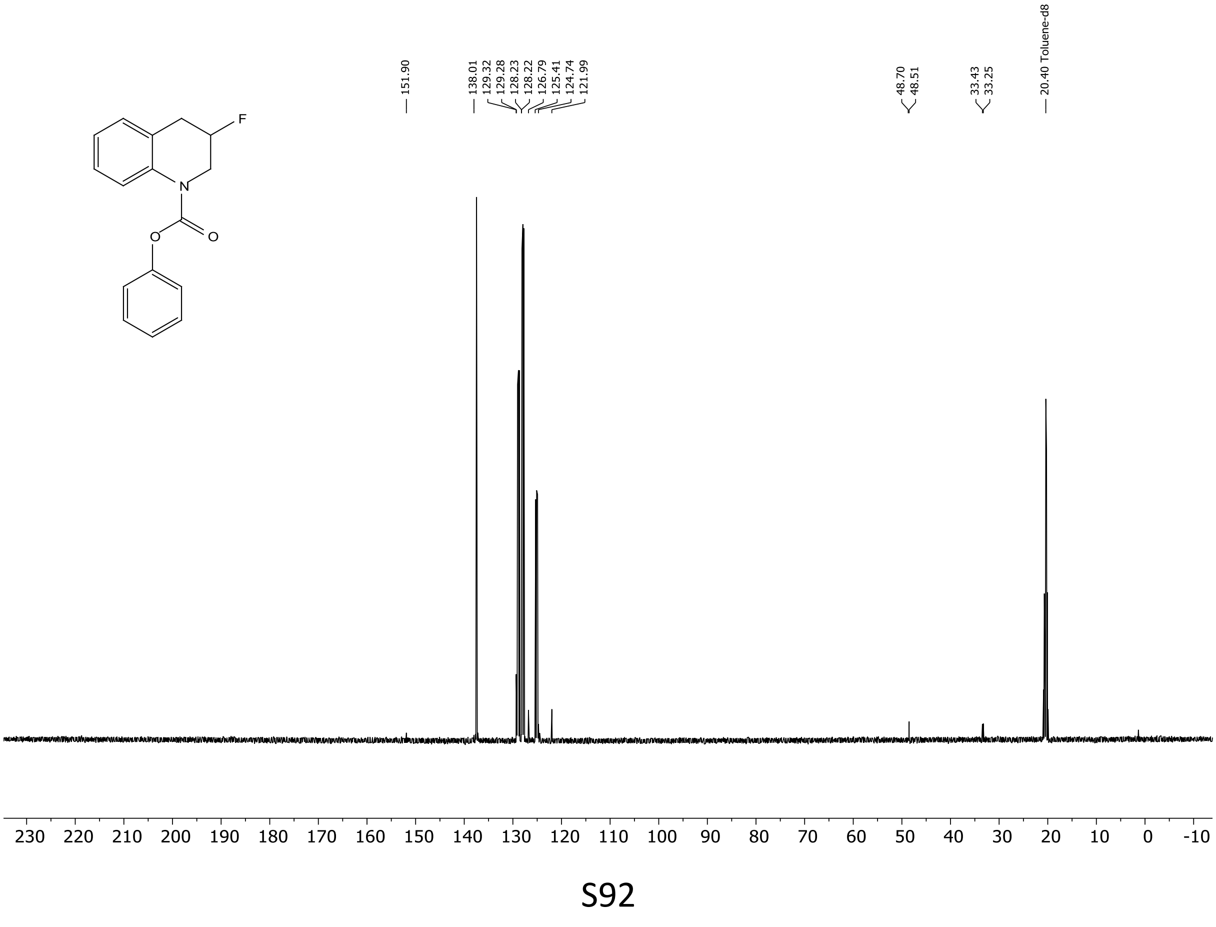

$^{19}F\{^{1}H\}$ NMR (470 MHz, toluene-d_8) – compound **46**

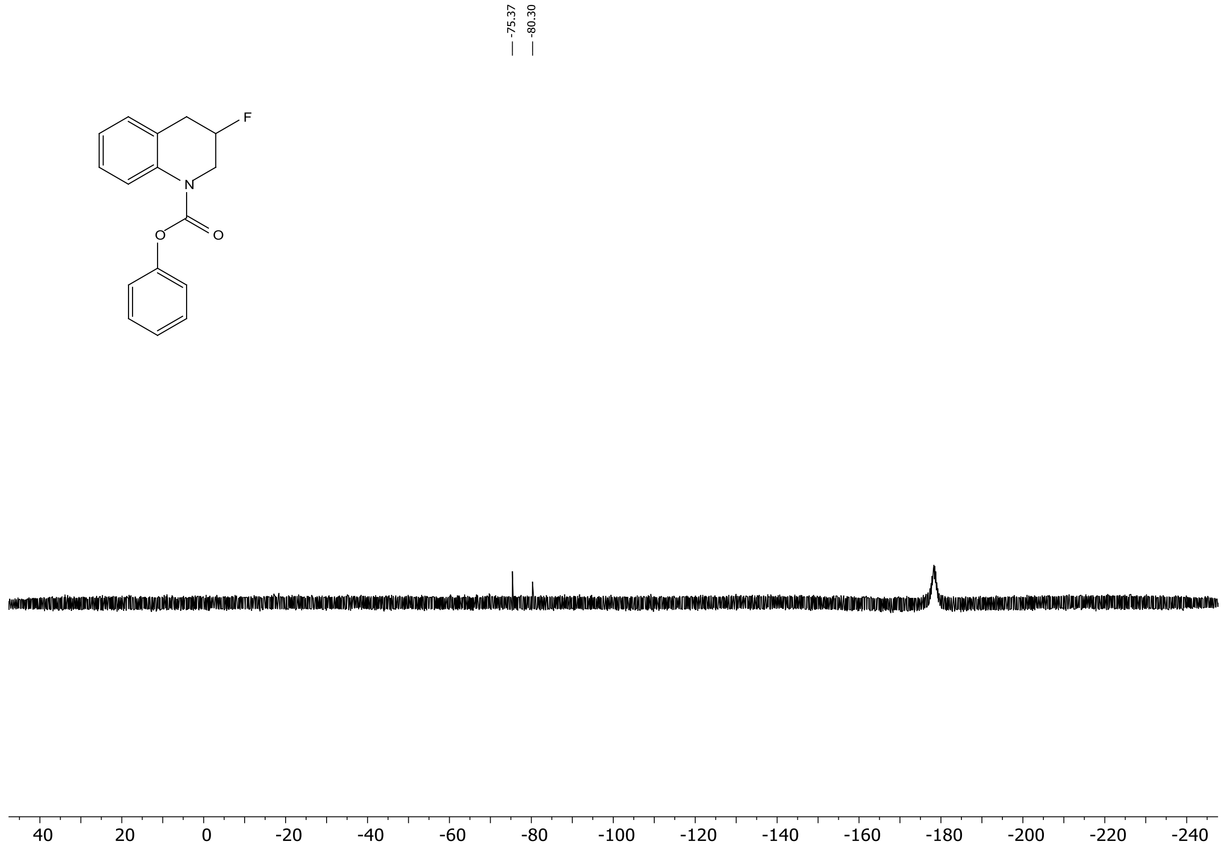

^{1}H NMR (500 MHz, toluene-d_8) – compound **47**

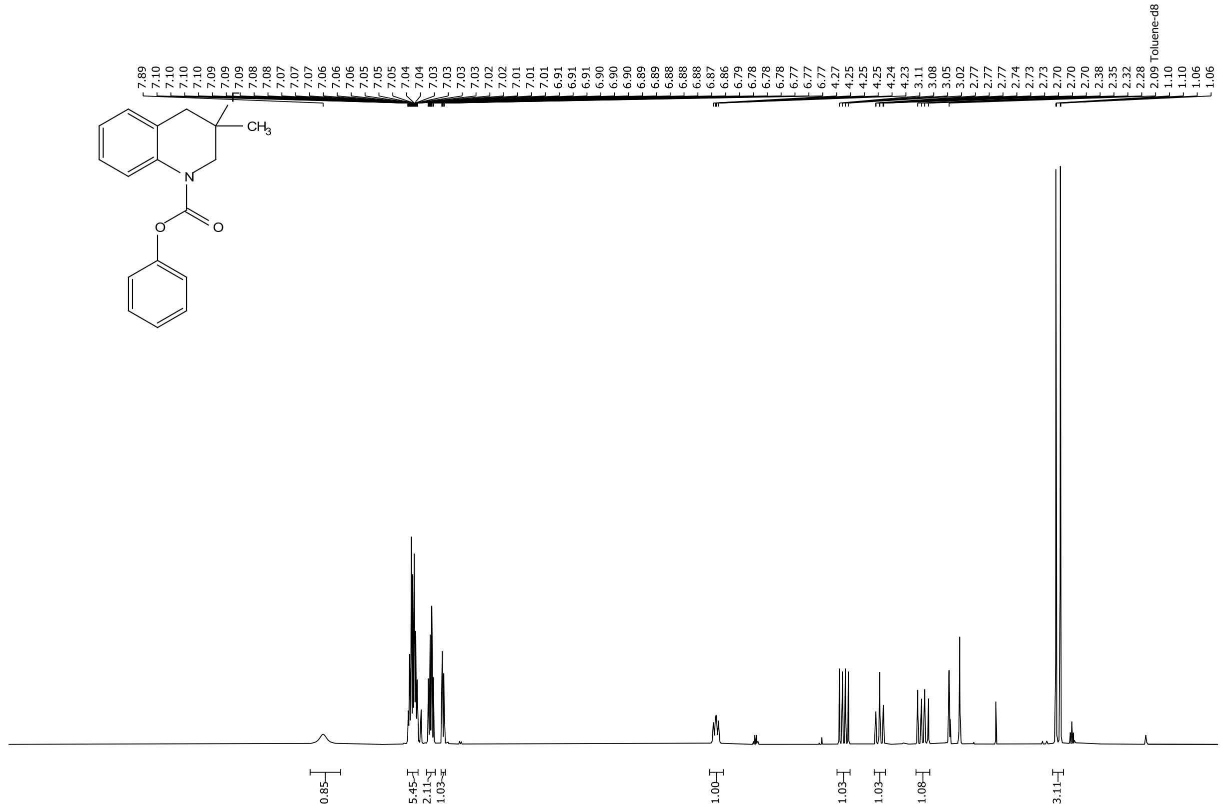

$^{13}C\{^1H\}$ NMR (126 MHz, toluene-d_8) – compound **47**

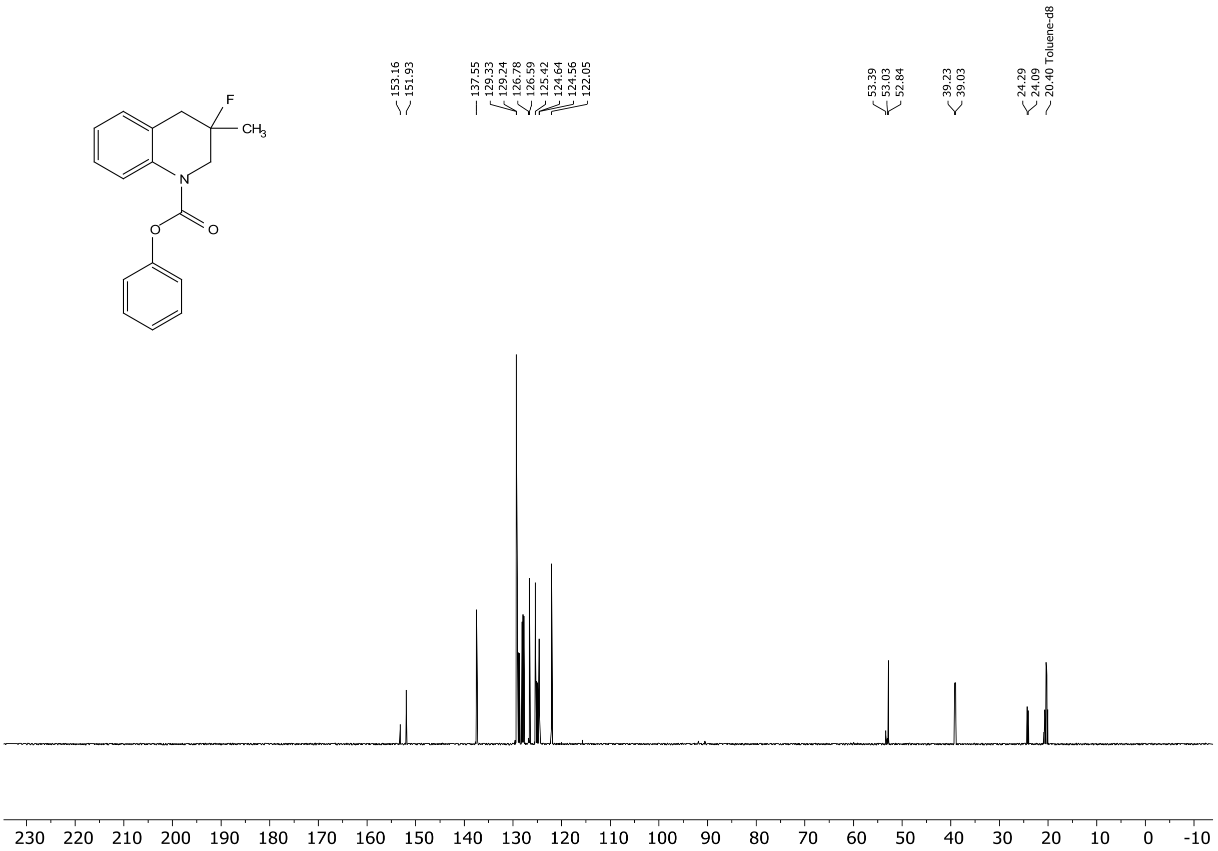

$^{19}F\{^1H\}$ NMR (470 MHz, toluene-d_8) – compound **47**

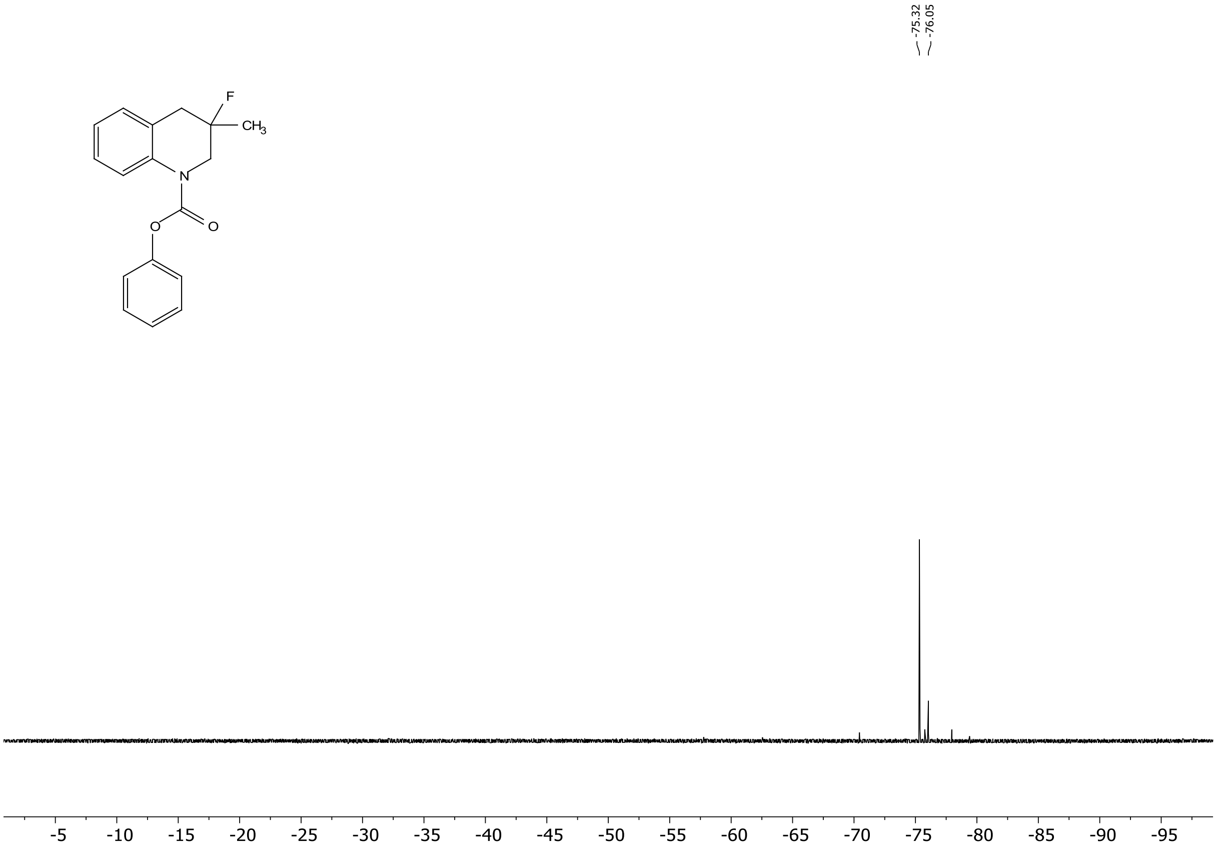

^{1}H NMR (400 MHz, chlorofrom-d_3) – compound **48**

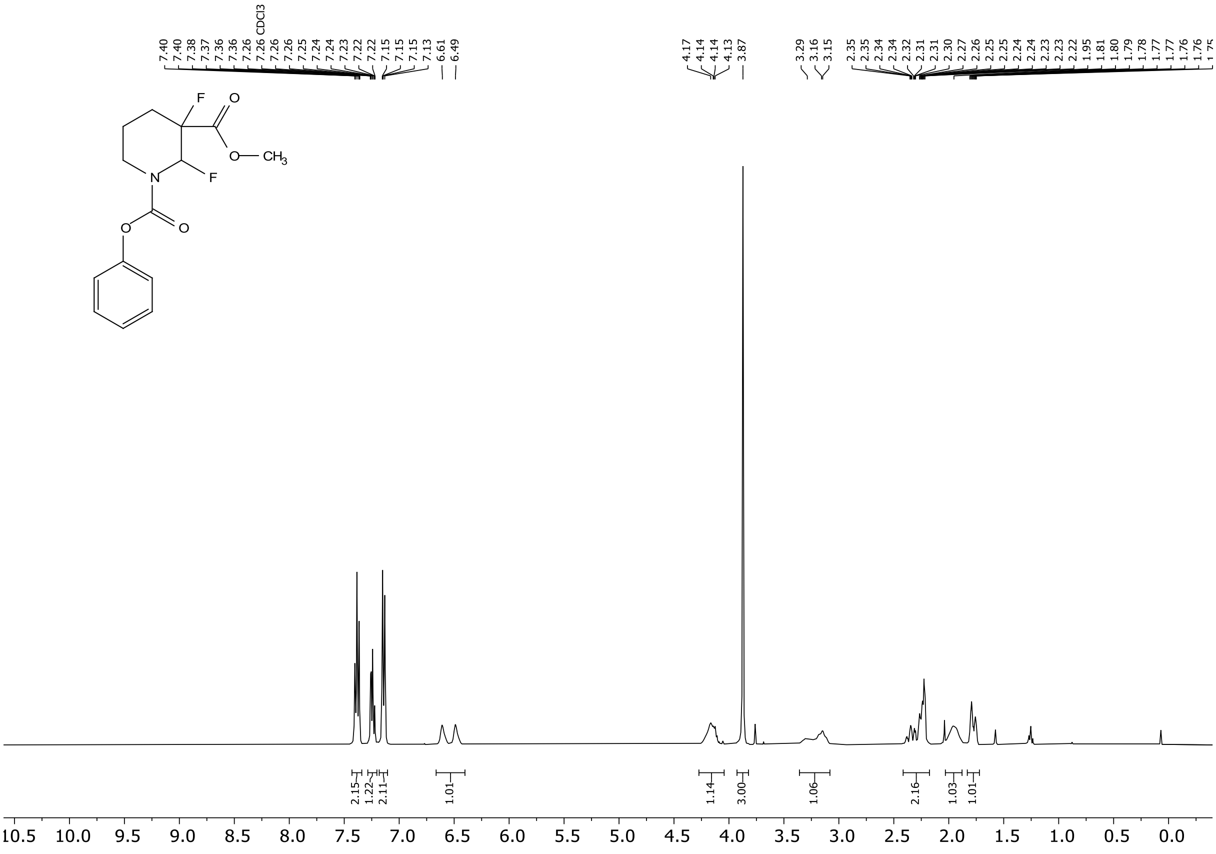

^{13}C{^{1}H} NMR (101 MHz, chlorofrom-d_3) – compound **48**

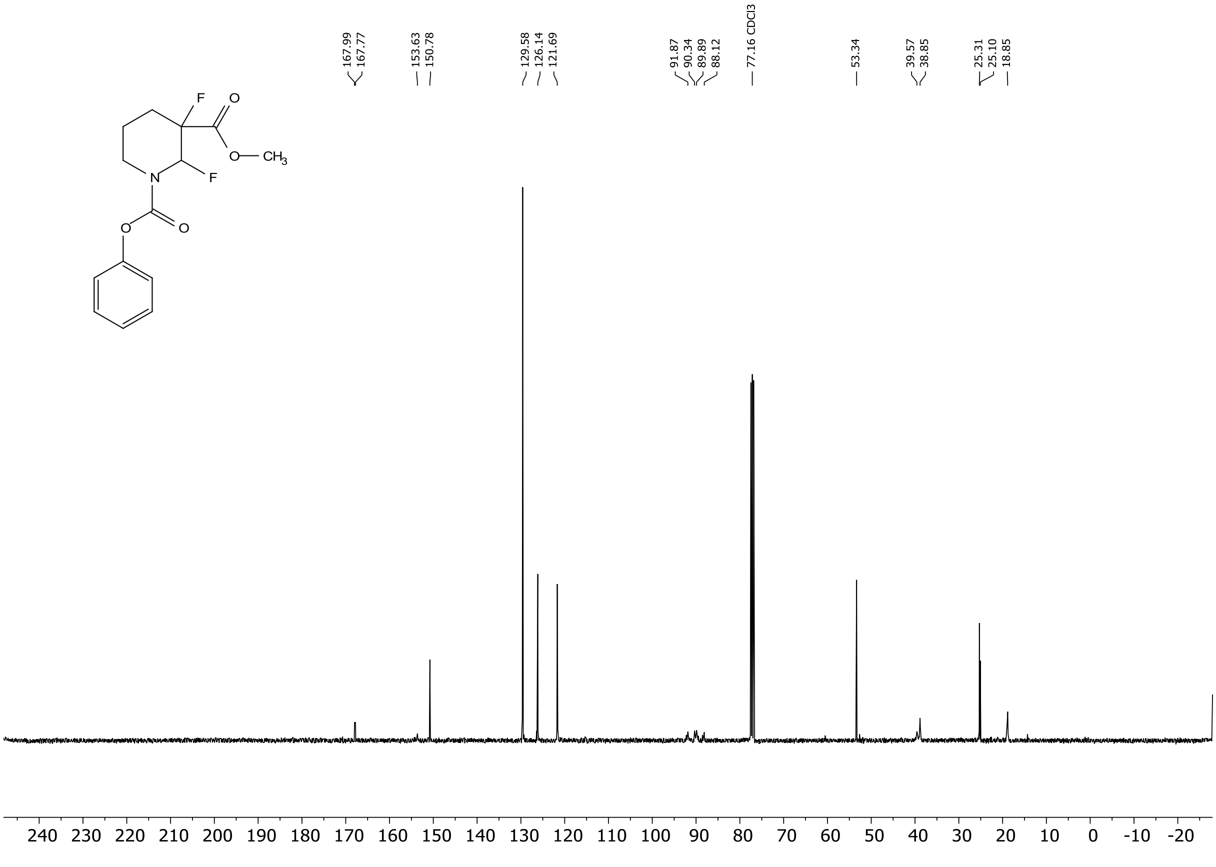

$^{19}F\{^{1}H\}$ NMR (376 MHz, chloroform-d_3) – compound **48**

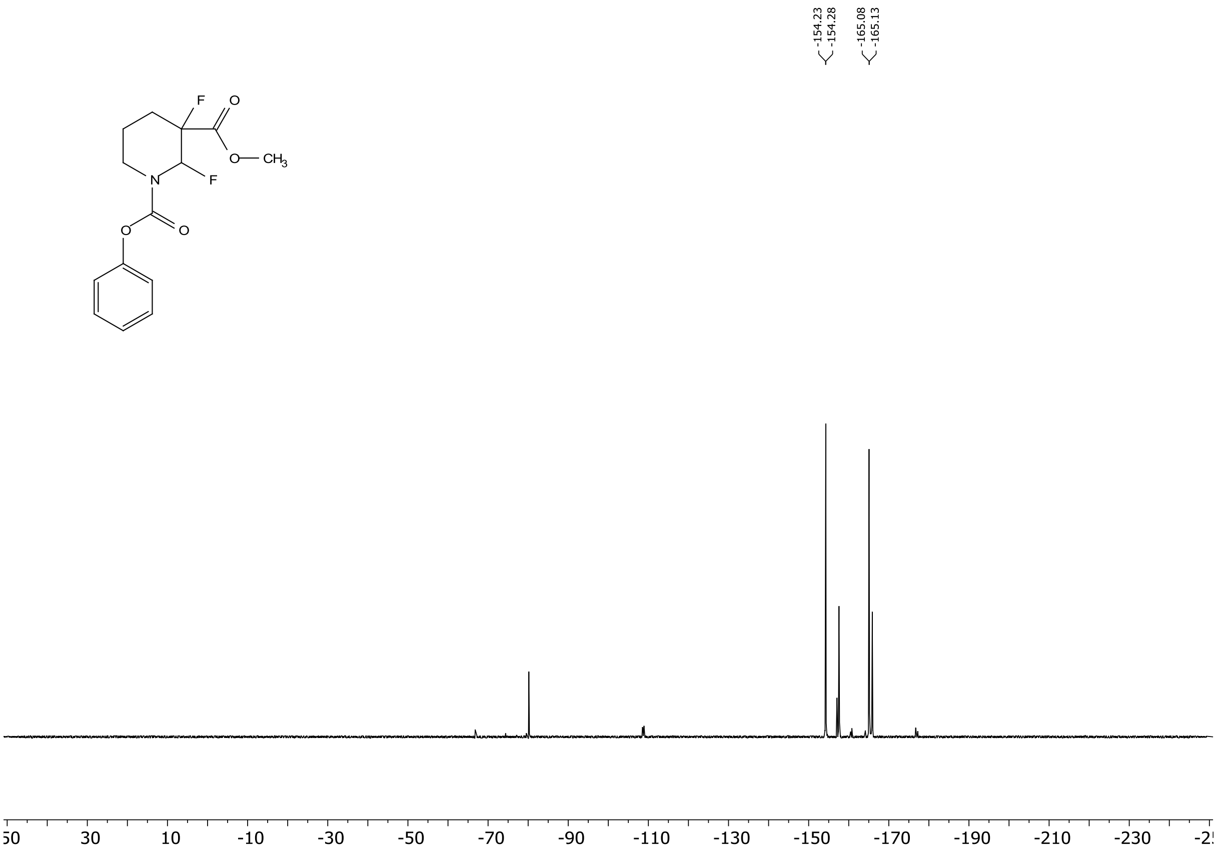

^{1}H NMR (500 MHz, chlorofrom-d_3) – compound **49**

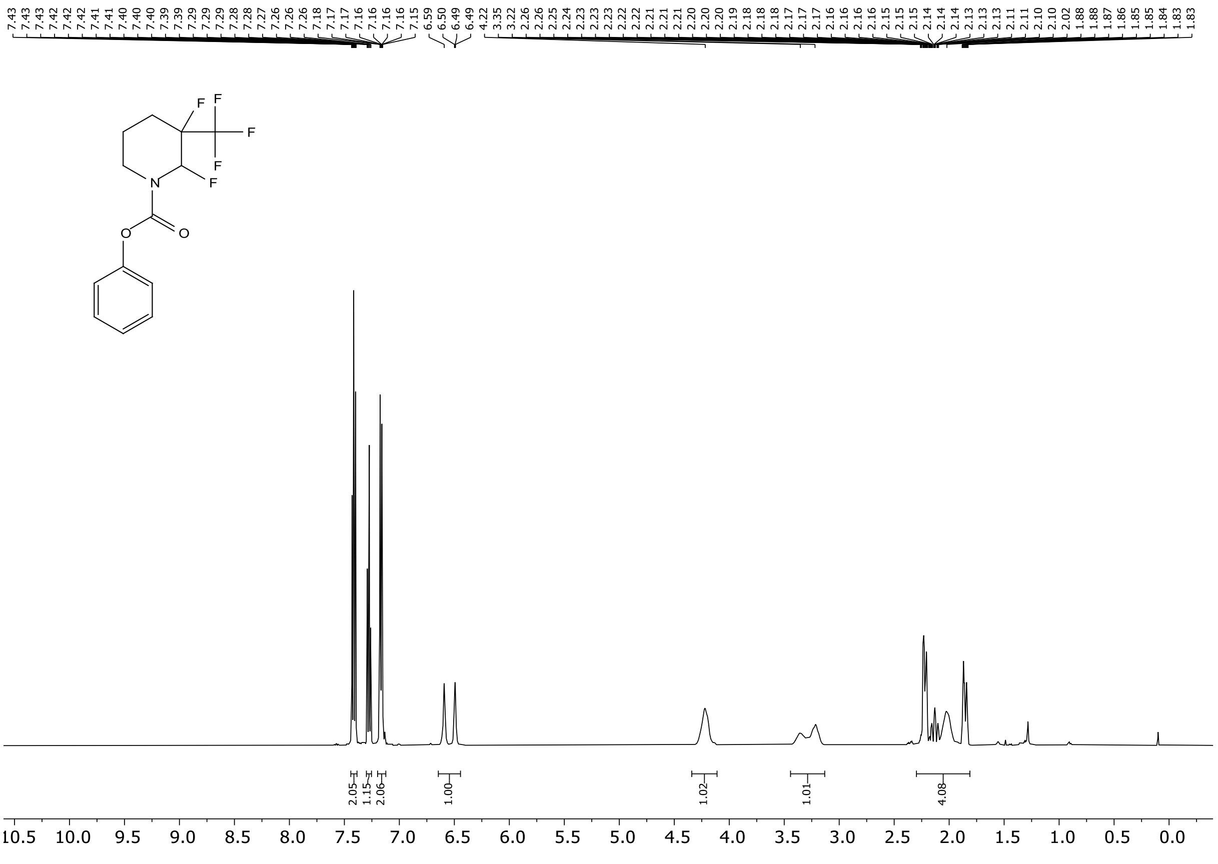

^{13}C{^{1}H, ^{19}F} NMR (126 MHz, chlorofrom-*d*$_3$) – compound **49**

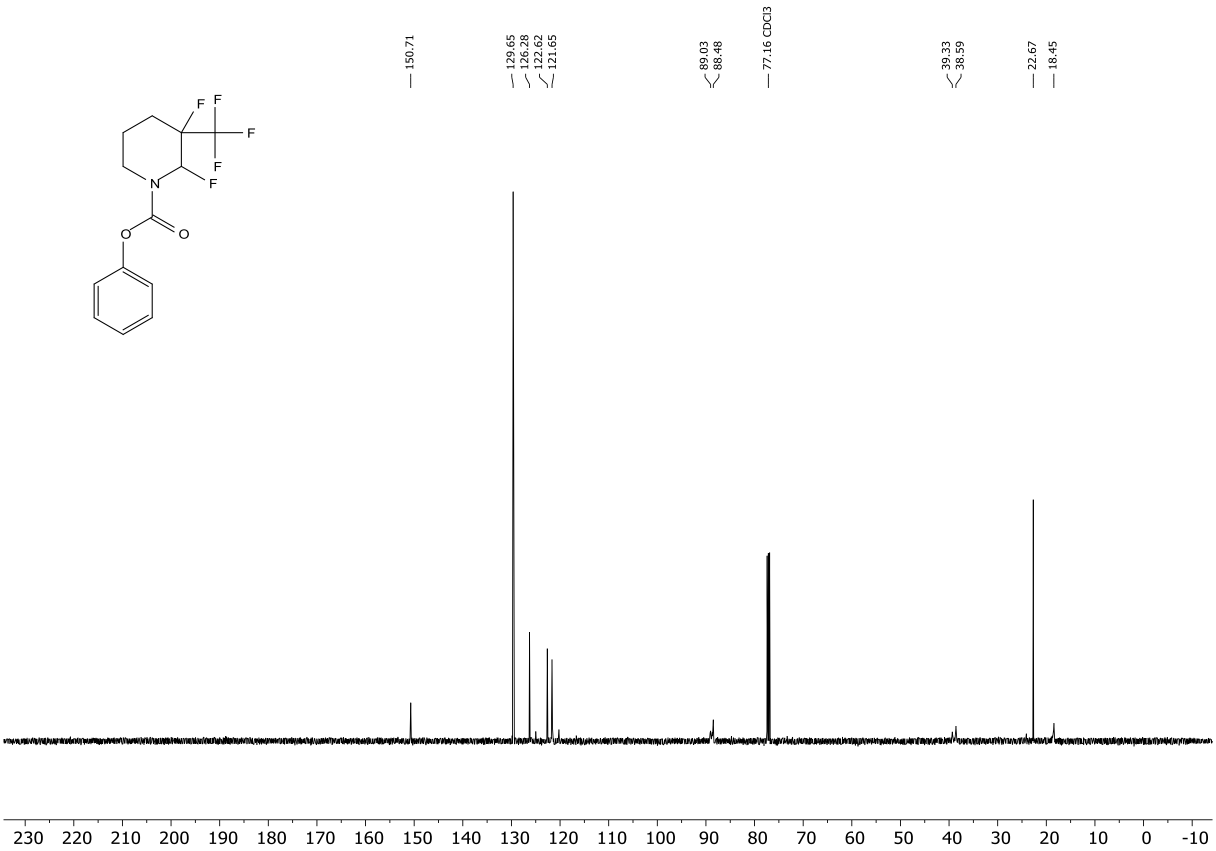

^{19}F{^{1}H} NMR (470 MHz, chloroform-*d*$_3$) – compound **49**

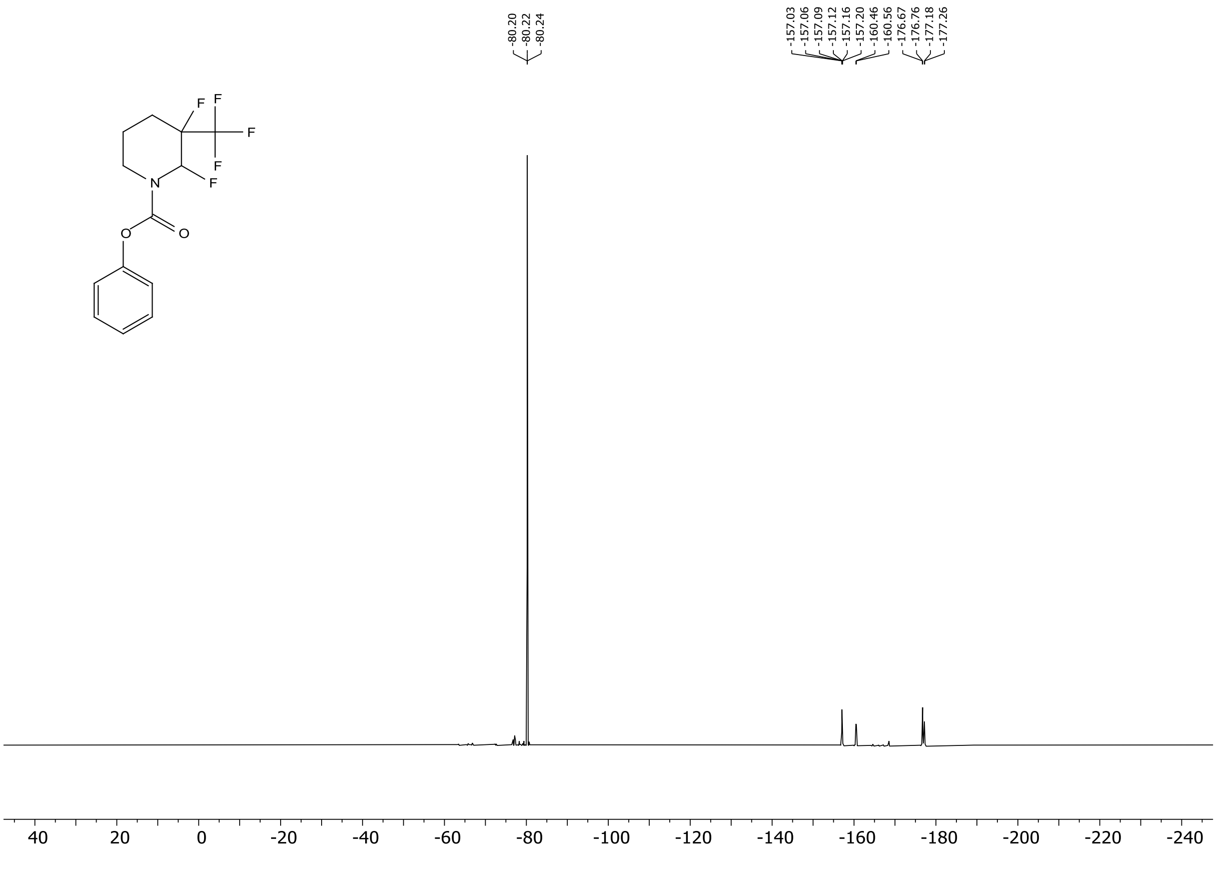

^{1}H NMR (400 MHz, chlorofrom-d_3) – compound **50**

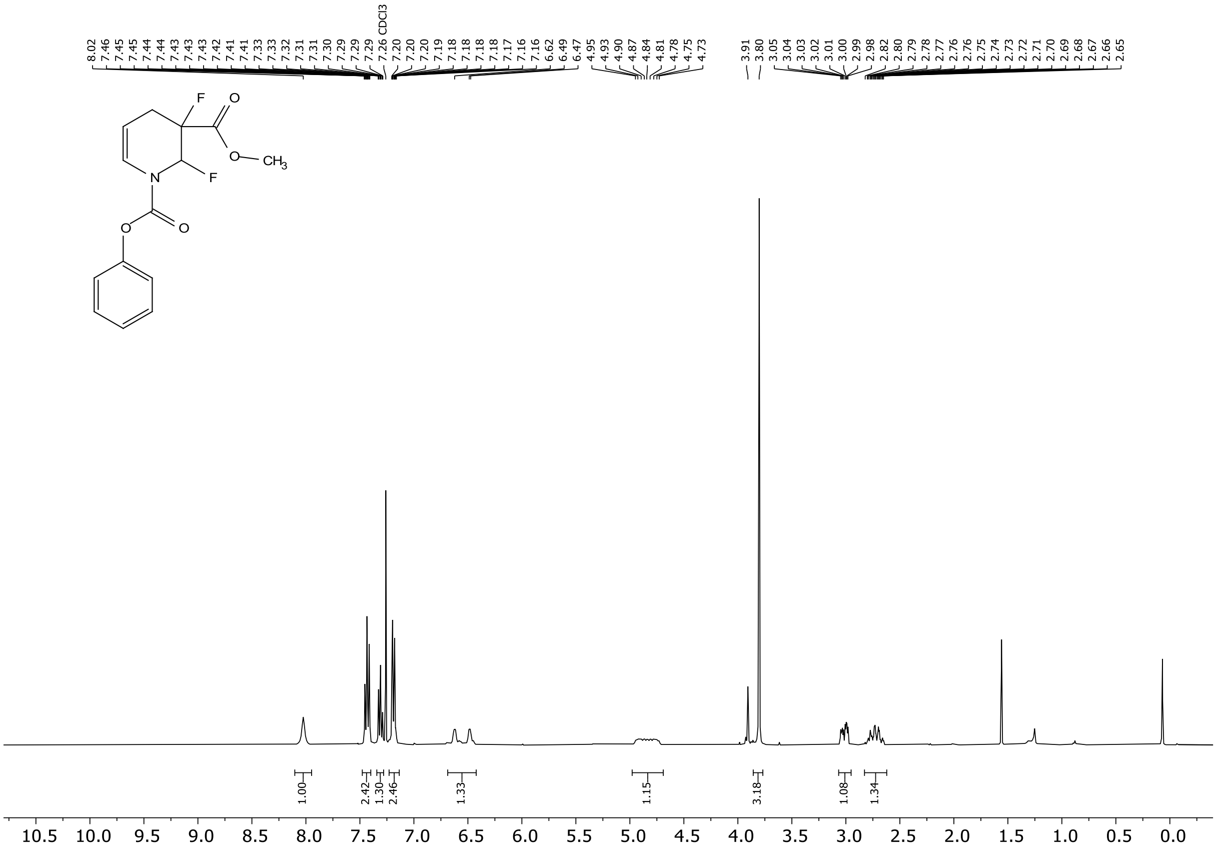

^{13}C{^{1}H} NMR (101 MHz, chlorofrom-d_3) – compound **50**

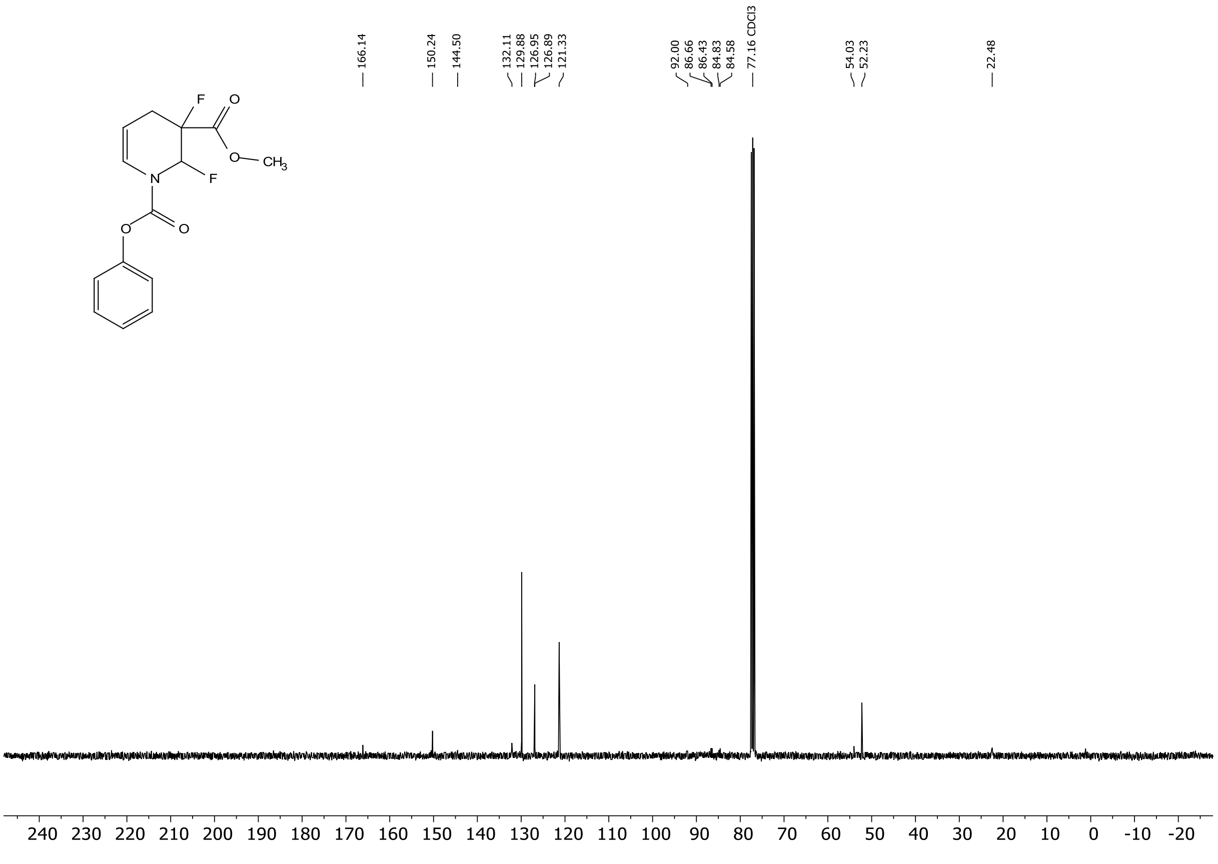

$^{19}F\{^{1}H\}$ NMR (376 MHz, chloroform-d_3) – compound **50**

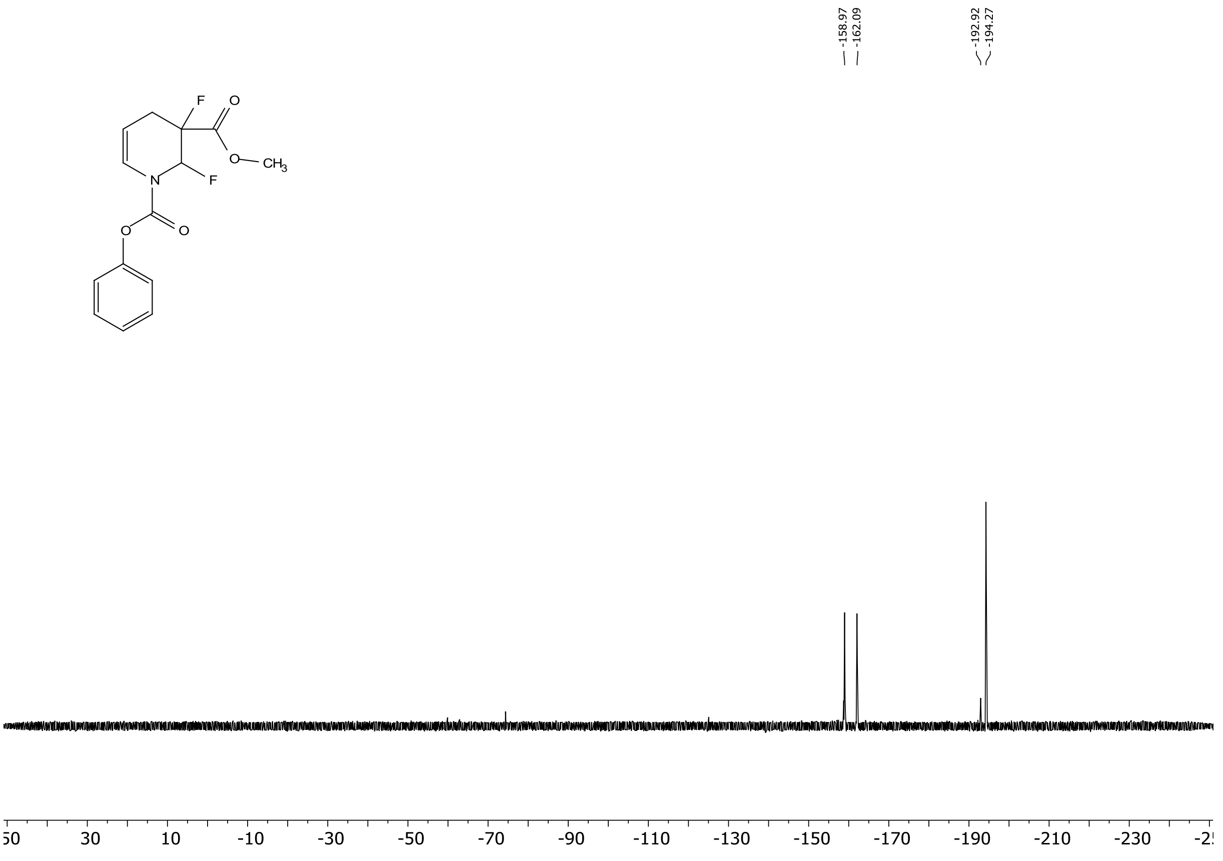

^{1}H NMR (400 MHz, chlorofrom-d_3) – compound **51**

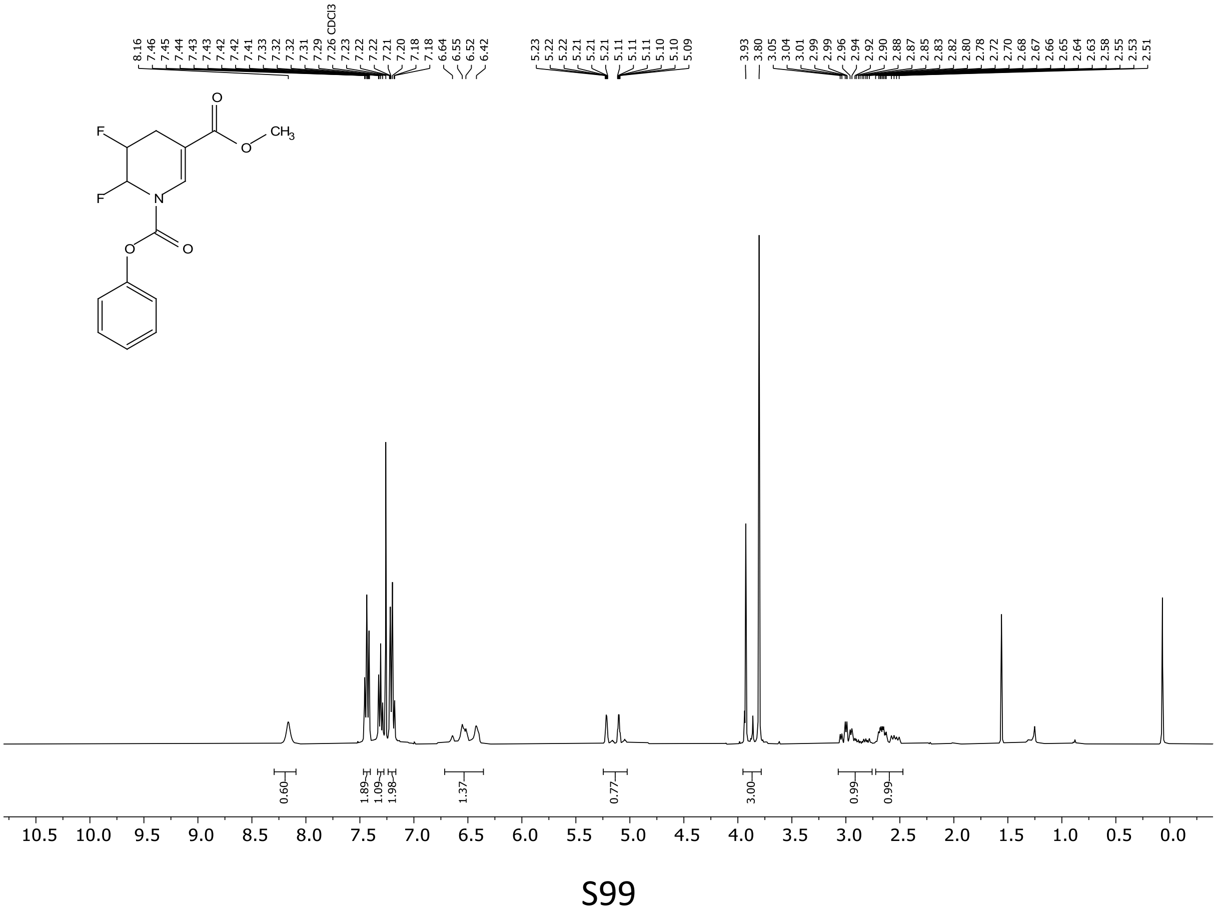

$^{13}C\{^1H\}$ NMR (101 MHz, chlorofrom-d_3) – compound **51**

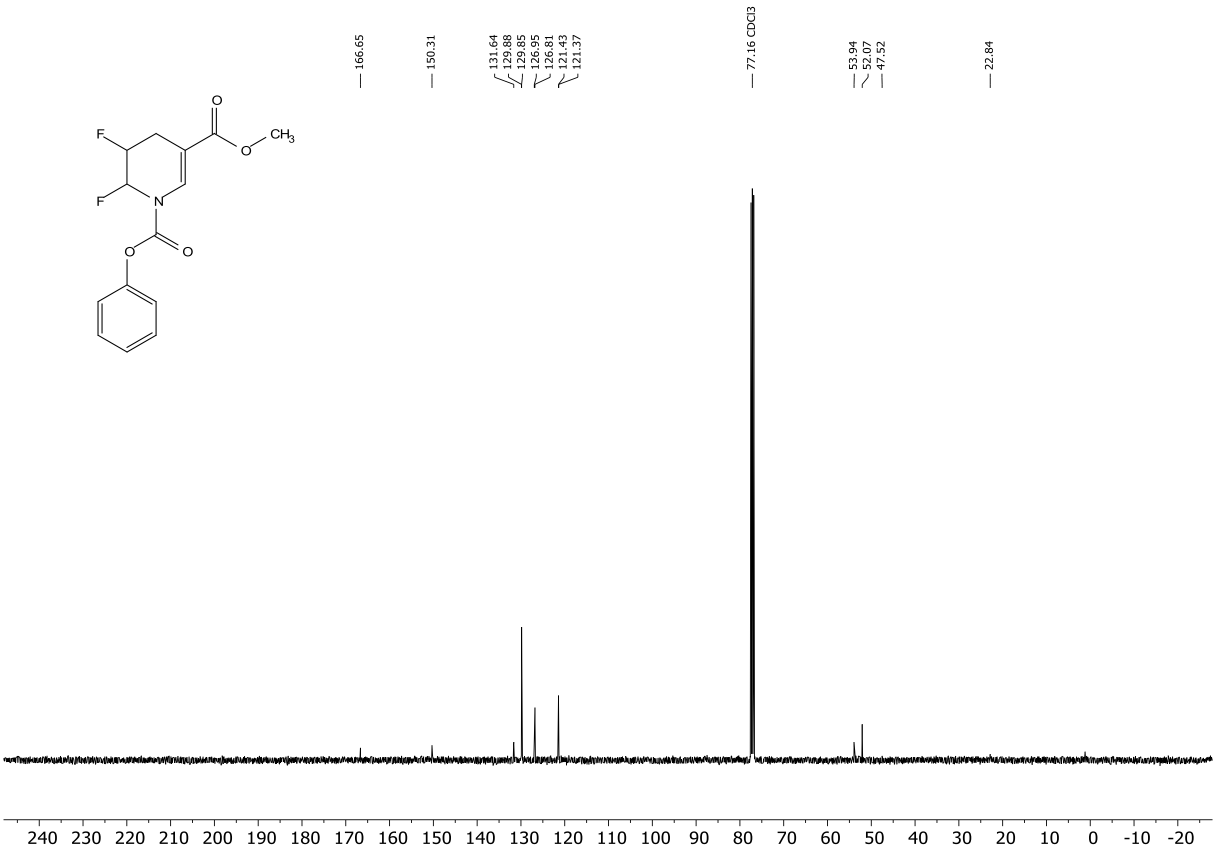

$^{19}F\{^1H\}$ NMR (376 MHz, chloroform-d_3) – compound **51**

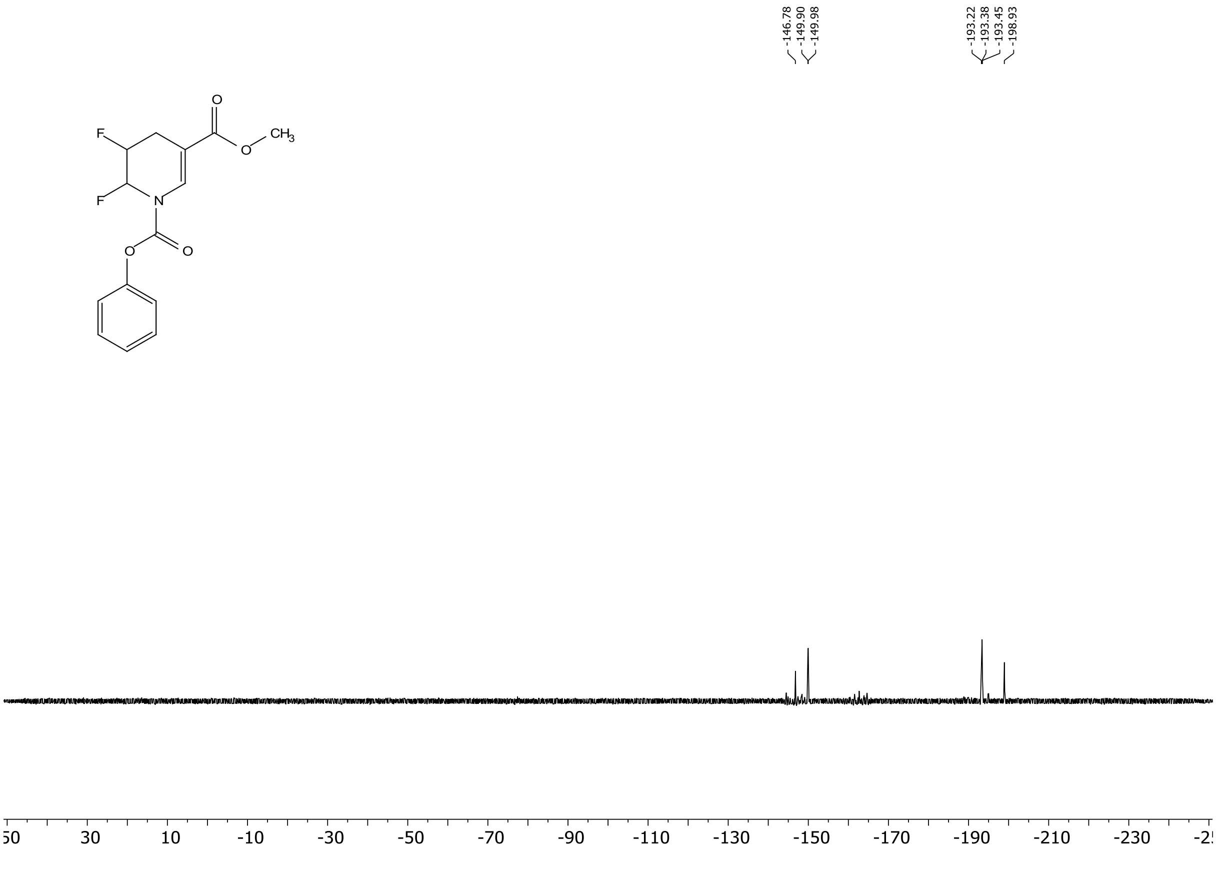

^{1}H NMR (500 MHz, toluene-d_8) – compound **S3**

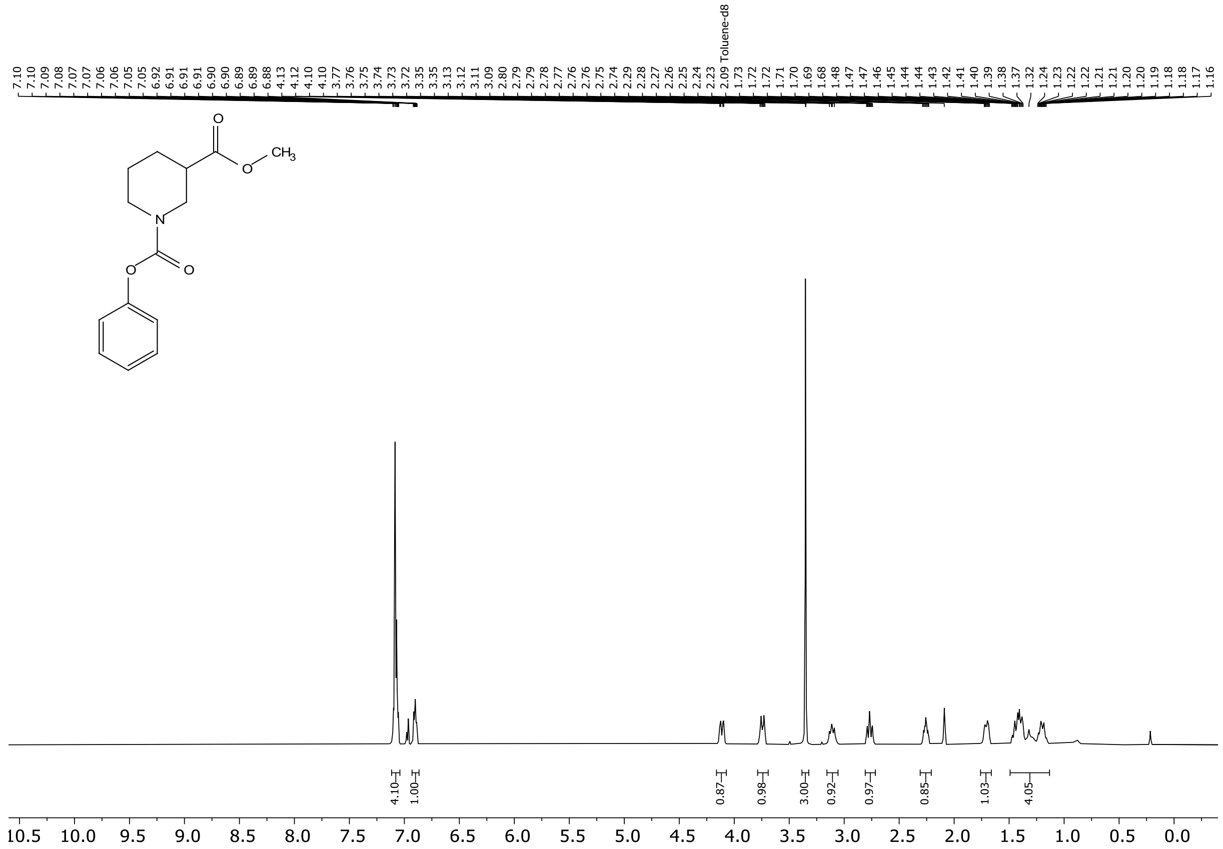

^{13}C{^{1}H} NMR (126 MHz, toluene-d_8) – compound **S3**

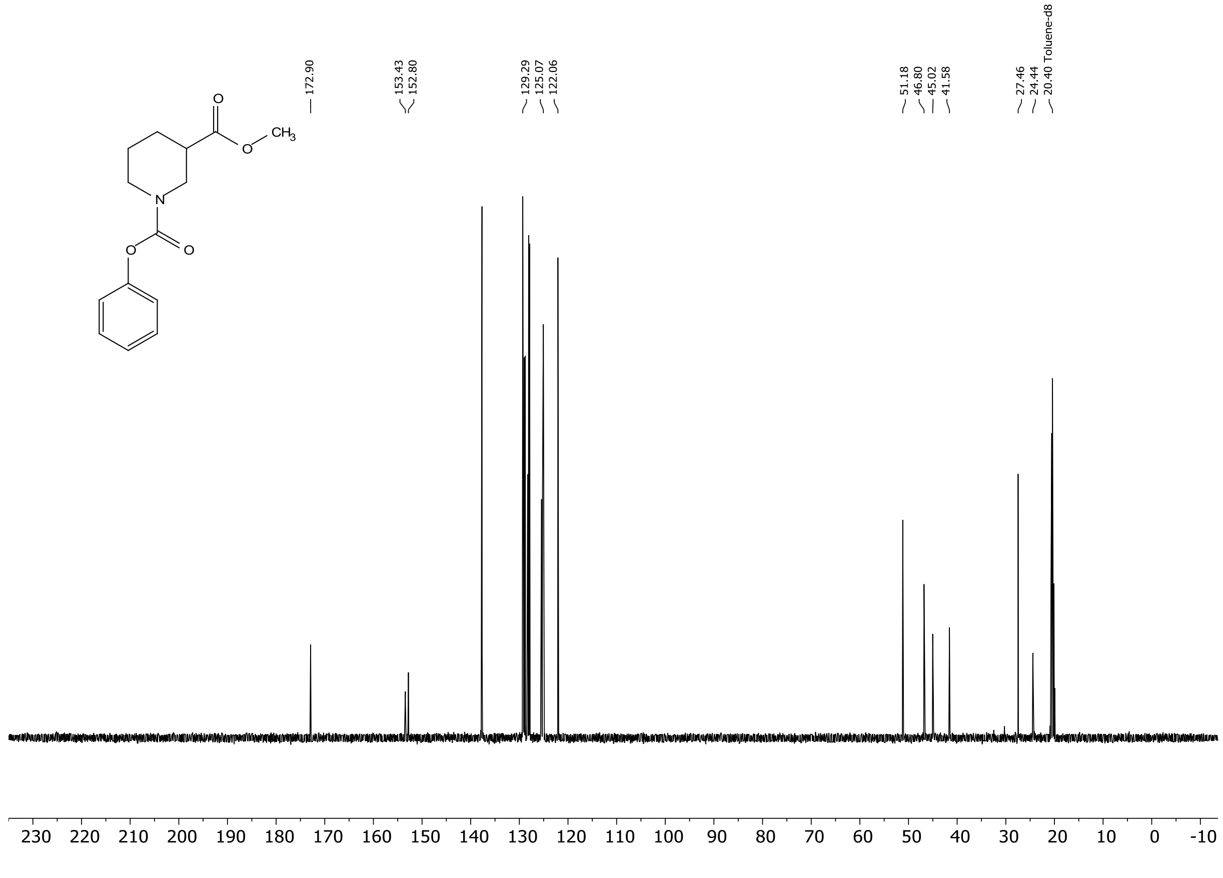

^{1}H NMR (500 MHz, toluene-d_8) – compound **52**

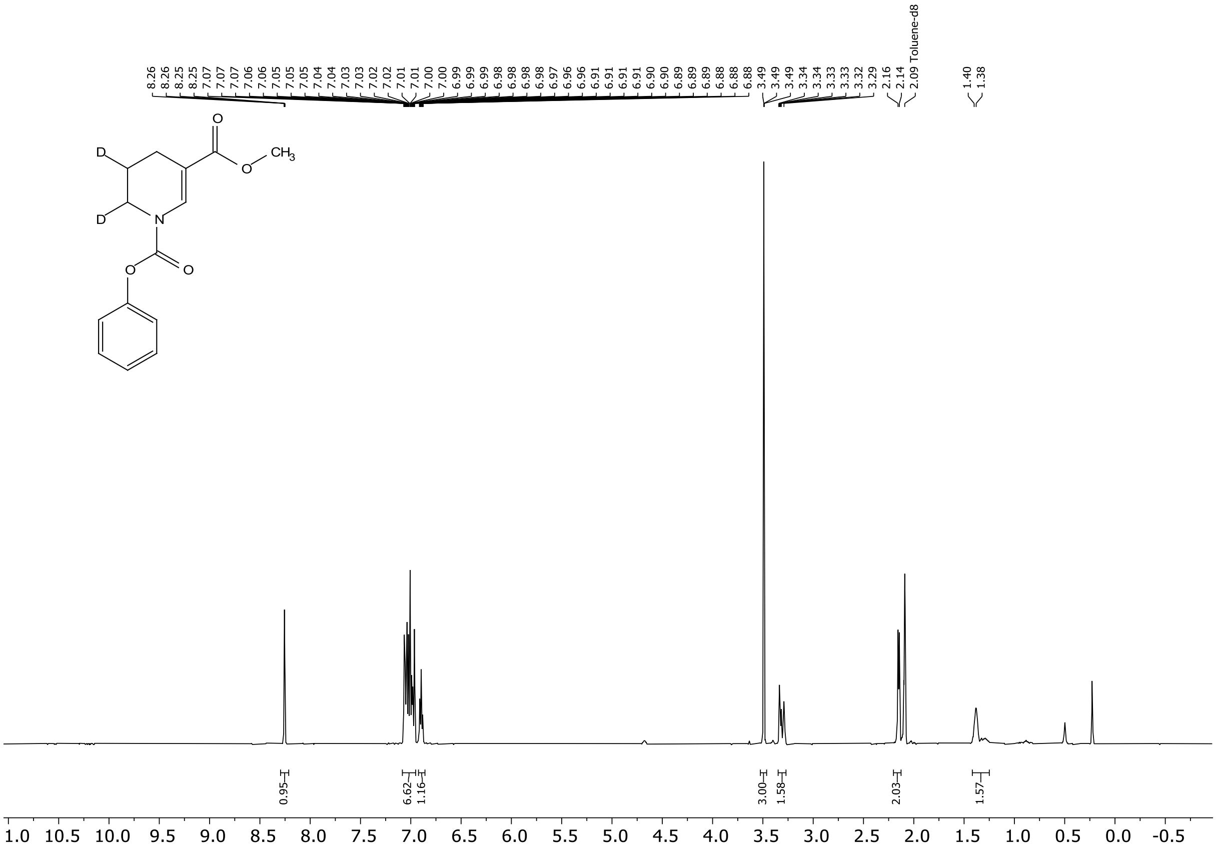

^{13}C{^{1}H} NMR (126 MHz, toluene-d_8) – compound **52**

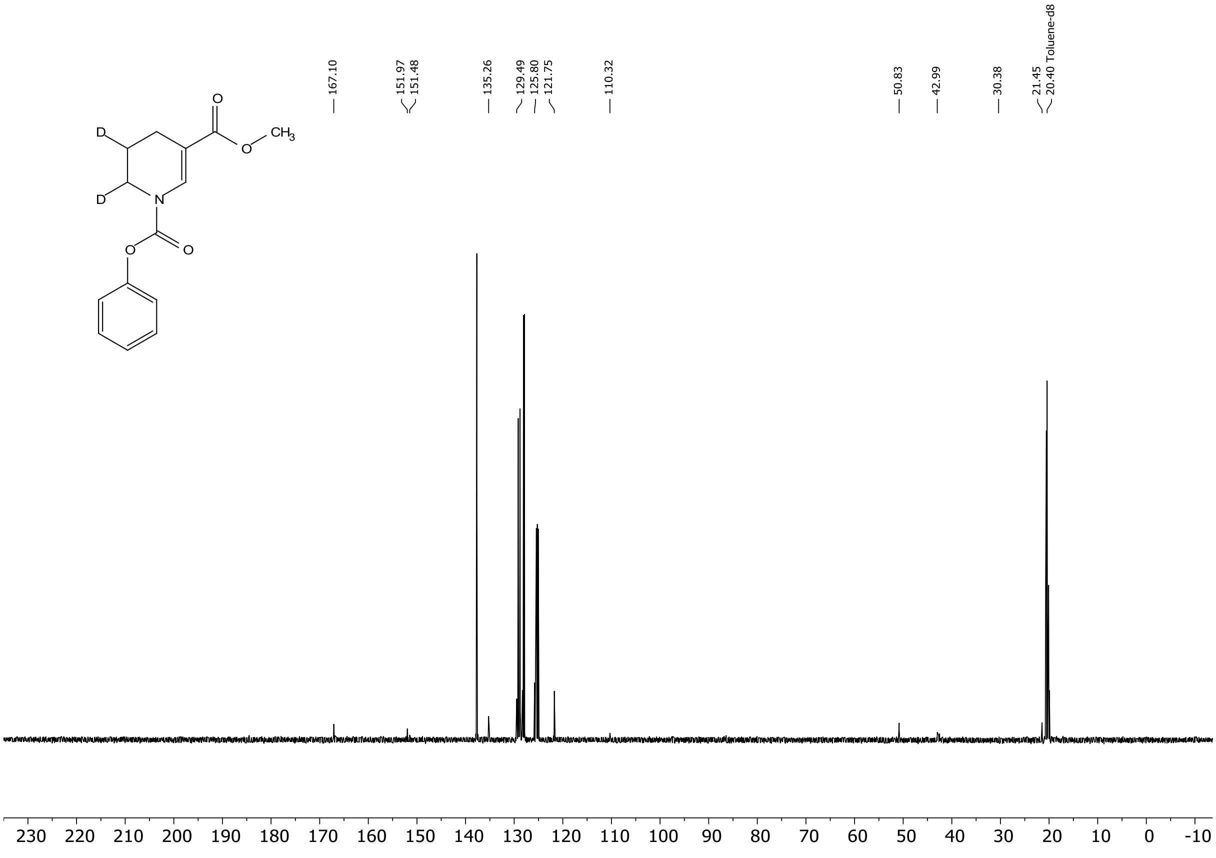

^{1}H NMR (500 MHz, toluene-d_8) – compound **53**

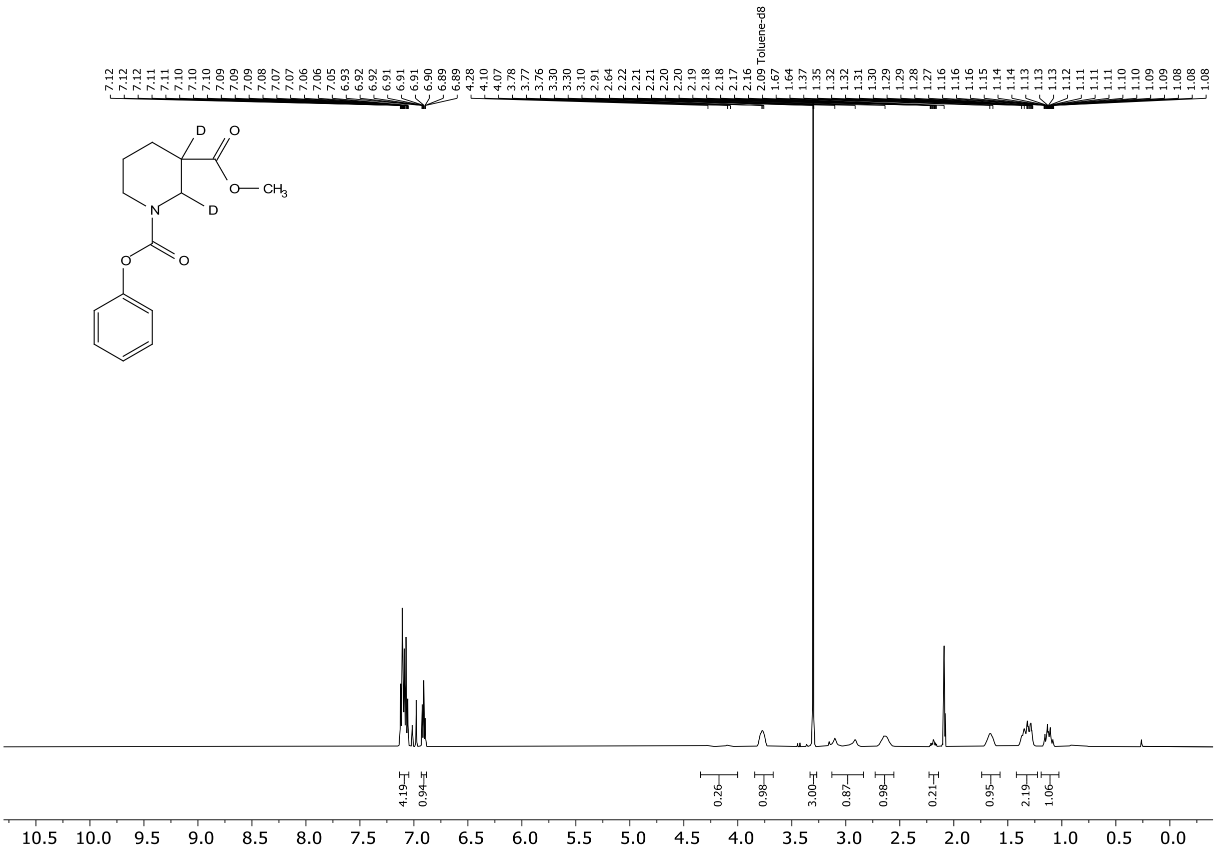

^{13}C{^{1}H} NMR (126 MHz, toluene-d_8) – compound **53**

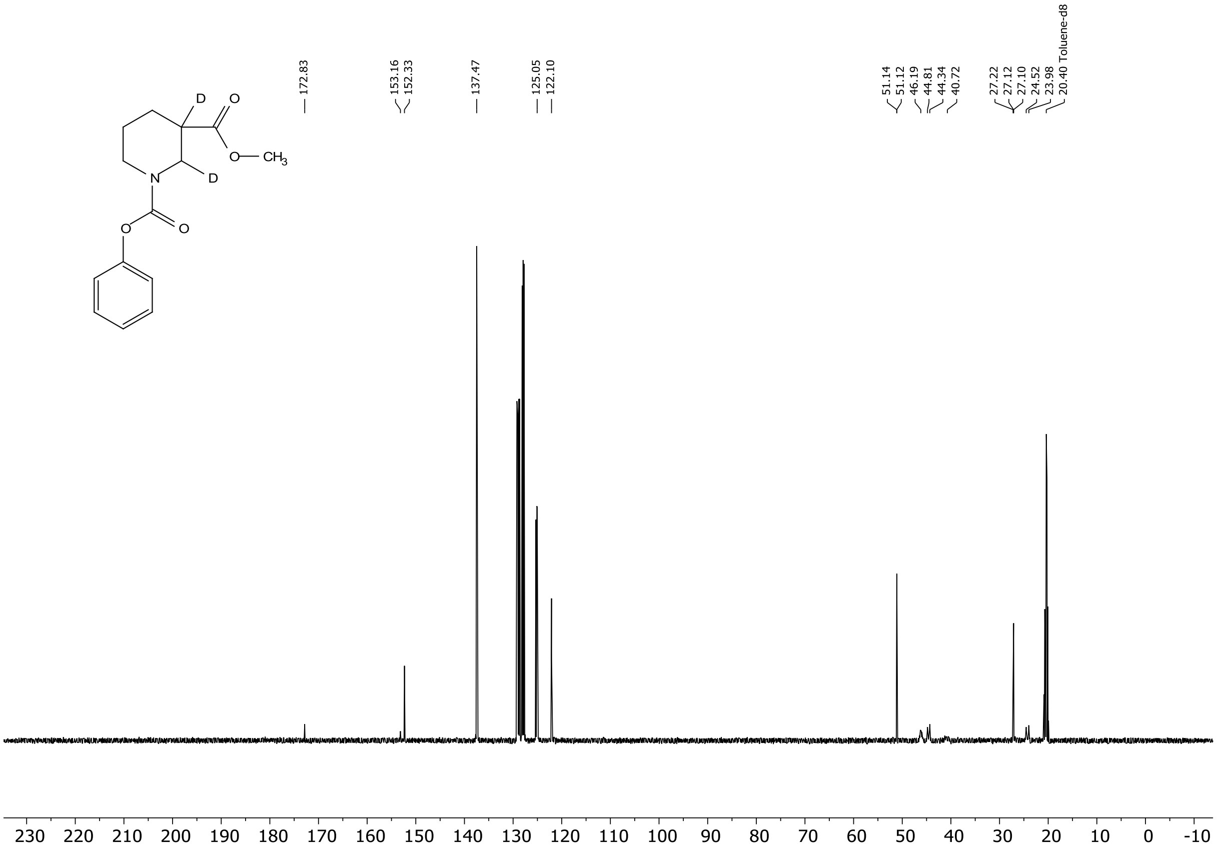

4. References

1. *APEX3* (**2016**), *SAINT* (**2015**) and *SADABS* (**2015**), Bruker AXS Inc., Madison, Wisconsin, USA.
2. G. M. Sheldrick, *SHELXT* – Integrated space-group and crystal-structure determination. *Acta Cryst.* **2015**, *A71*, 3.
3. G. M. Sheldrick, Crystal structure refinement with *SHELXL*. *Acta Cryst.* **2015**, *C71*, 3.
4. *XP* – Interactive molecular graphics*, Version 5.1,* Bruker AXS Inc., Madison, Wisconsin, USA, **1998**.
5. L. Pitzer, F. Schäfers, F. Glorius, *Angew. Chem. Int. Ed.* **2019**, *58*, 8572.
6. T. Wagener, A. Heusler, Z. Nairoukh, K. Bergander, C. G. Daniliuc, F. Glorius, *ACS Catal.* **2020**, *10*, 12052.
7. F. Marsais, F. Trécourt, P. Bréant, G. Quéguiner, *J. Heterocycl. Chem.* **1988**, *25*, 81.
8. Z. Nairoukh, M. Wollenburg, C. Schlepphorst, K. Bergander, F. Glorius, *Nat. Chem.* **2019**, *11*, 264.
9. J. G. Atkinson, D. W. MacDonald, R. S. Stuart, P. H. Tremaine, *Can. J. Chem.* **1967**, *45*, 2583.
